# 8th IAS Conference on HIV Pathogenesis, Treatment & Prevention 19–22 July 2015, Vancouver, Canada

**DOI:** 10.7448/IAS.18.5.20479

**Published:** 2015-07-22

**Abstract MOAA0101–Figure 1:**
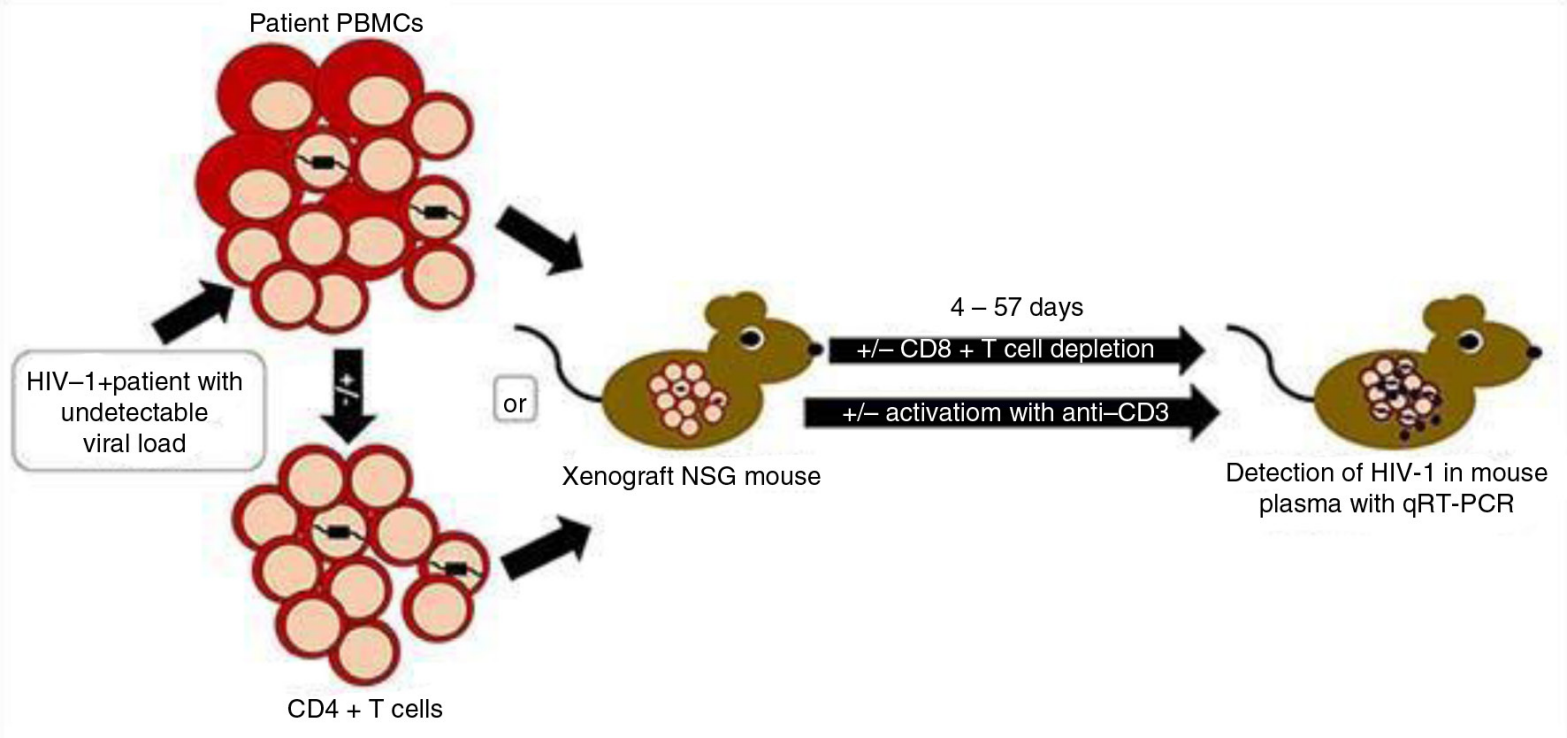
MVOA for detection of residual virus.

