# Supplementary material for: 8th IAS Conference on HIV Pathogenesis, Treatment & Prevention 19–22 July 2015, Vancouver, Canada
Source: J Int AIDS Soc. 2015 Jul 22;18(5Suppl 4):20479. doi: 10.7448/IAS.18.5.20479 (PMC4513179; doi:10.7448/IAS.18.5.20479)
Supplement: Related texts

# Sub article: A murine viral outgrowth assay to detect HIV in patients with undetectable viral loads

**DOI:** 10.7448/IAS.18.5.20320

**Published:** 2015-07-22

**Authors:** Kelly Metcalf Pate, Chris Pohlmeyer, Victoria Walker-Sperling, Jeremy Foote, Kevin Najarro, Catherine Cryer, Maria Salgado, Lucio Gama, Elizabeth Engle, Erin Shirk, Suzanne Queen, Stanley Chioma, Meghan Vermillion, Brandon Bullock, Ming Li, Claire Lyons, Robert Adams, Chris Zink, Janice Clements, Joseph Mankowski, Joel Blankson

**Affiliations:** 1 Department of Molecular and Comparative Pathobiology, Johns Hopkins University School of Medicine, Baltimore, United States; 2 Department of Medicine, Johns Hopkins University School of Medicine, Baltimore, United States; 3 School of Veterinary Medicine, University of Pennsylvania, Philadelphia, United States; 4 Institue IrsiCaixa, Universitat Autònoma de Barcelona, Badalona, Spain; 5 Cummings School of Veterinary Medicine, Tufts University, North Grafton, United States

## Abstract

**Introduction:**

Sensitive assays are needed for detection of residual HIV in patients with undetectable plasma viral loads to determine if eradication strategies are effective. The gold standard quantitative viral outgrowth assay (QVOA) underestimates the magnitude of the viral reservoir, while sensitive PCR-based assays lack the ability to distinguish replication competent from defective virus. We sought to determine whether xenograft of leukocytes from HIV-1 infected patients with undetectable plasma viral loads into severely immunocompromised mice would result in viral amplification and measurable viral loads within the aberrant murine host.

**Methods:**

We evaluated whether xenograft of 1) peripheral blood mononuclear cells (PBMCs) from five HIV-1+ patients on suppressive antiretroviral therapy (ART), 2) PBMCs or purified resting CD4+ T cells from 5 HIV-1+ elite suppressors (ES), or 3) PBMCs from a Simian Immunodeficiency Virus (SIV)+ pigtailed macaque on suppressive ART, all with undetectable plasma viral loads, into NOD. Cg-PrkdcscidIl2rgtm1Wjl/SzJ (NSG) mice resulted in viral amplification in the mouse. Successful xenograft of mice was confirmed by flow cytometry. Human CD8+ T cells were depleted in humanized mice with depleting antibody, and CD4+ T cells were activated in a subset of mice with activating anti-CD3. Plasma viral loads in xenografted mice were quantified using qRT-PCR, and compared to plasma viral load and QVOA results from the human or macaque donor.

**Results:**

With this murine viral outgrowth assay (MVOA), we amplified HIV-1 from all 10 HIV+ subjects with undetectable plasma viral load, including an ES from whom we were unable to recover virus by QVOA. We detected HIV in mice an average of 20 days after xenograft with PBMCs from patients on suppressive ART, and an average of 28 days after xenograft with PBMCs or resting CD4+ T cells from ES. For two of the mice xenografted with CD4+ T cells from ES, we detected HIV only after activation with anti-CD3. We similarly detected SIV in macaquized mice by seven days post-xenograft.

**Conclusions:**

The MVOA has the potential to serve as a powerful tool to identify residual HIV-1 in patients with undetectable viral loads, such as those who have undergone promising cure therapies.

# Sub article: Virologic and immunologic correlates of viral control post-ART interruption in SIV-infected rhesus macaques

**DOI:** 10.7448/IAS.18.5.20321

**Published:** 2015-07-22

**Authors:** Luca Micci, Emily Ryan, Rémi Fromentin, Clarisse Benne, Nicolas Chomont, Jeffrey Lifson, Mirko Paiardini

**Affiliations:** 1 YNPRC, Emory University, Atlanta, United States; 2 Département de microbiologie, infectiologie et immunologie, Université de Montréal, Montreal, Canada; 3 Case Western Reserve University, Cleveland, United States; 4 NCI/NIH, Frederick, United States

## Abstract

**Introduction:**

Antiretroviral therapy (ART) does not eradicate HIV and the virus rebounds upon treatment interruption. Recently, a sustained control of HIV replication in the absence of ART has been achieved in a subset of patients starting ART early after infection, defined as post-ART treatment controllers (PTC). Unfortunately, the virologic and immunologic determinants of post-ART control of HIV replication are still unclear, particularly in tissues. Here, we used the well-established model of SIV-infection in rhesus macaques (RMs) to investigate the existence of PTC in this model and the features associated with post-ART SIV control.

**Methods:**

Fifteen RMs (B*08^-^ and B*17) were infected (i.v.) with SIV_mac239_. All 15 animals initiated a five-drug ART regimen 60 days after infection, which was maintained for seven months. ART was then interrupted and RMs monitored for eight additional months. Blood (PB), lymph node (LN) and colorectal (RB) biopsies were collected throughout the study. Quantitative assessment of total SIV-DNA and RNA was performed on purified blood CD4 T cells and mucosal tissues by quantitative PCR; immunological parameters were determined by flow cytometry.

**Results:**

ART suppressed SIV-RNA to <60 copies/mL in all RMs. After ART interruption, six RMs controlled SIV viremia at <10^3^ copies/mL up to eight months off-ART (PTC), while nine RMs rebounded to pre-ART levels (non-controllers, NC). At pre-ART, PTC had significantly lower plasma viremia and SIV-DNA content, as well as higher CD4 T cell counts as compared to NC. Levels of intestinal CD4 T cells were similar, but PTC had higher frequencies of Th17 cells than NC. On-ART, PTC had significantly lower levels of residual plasma viremia (3 copies/mL, limit of detection) and SIV-DNA content (both in blood and colorectum). After ART interruption, SIV-DNA content rapidly increased in NC while it progressively decreased in PTC. Finally, in PTC control of SIV rebound associated with higher CD4 T cell levels and reduced immune activation in PB and RB during the entire off-ART period.

**Conclusions:**

Lower set point viremia, reduced cell-associated SIV-DNA and preserved Th17 cell homeostasis associate with improved virologic response to ART and sustained viral control post-ART interruption in SIV-infected RMs.

# Sub article: Anti-HIV antibody responses reflect the quantifiable HIV reservoir size

**DOI:** 10.7448/IAS.18.5.20322

**Published:** 2015-07-22

**Authors:** Sulggi Lee, Nicolas Chomont, Remi Fromentin, Robert Silicano, Janet Silicano, Douglas Richman, Una O'Doherty, Sarah Palmer, Peter Burbelo, Steven Deeks

**Affiliations:** 1 Department of Medicine, University of California San Francisco, San Francisco, United States; 2 Department of Immunology, University of Montreal, Montreal, Canada; 3 Department of Medicine, Johns Hopkins University, Baltimore, United States; 4 Department of Medicine, University of California San Diego, La Jolla, United States; 5 Department of Pathology and Laboratory Medicine, University of Pennsylvania, Philadelphia, United States; 6 Department of Medicine, University of Sydney, Sydney, Australia; 7 National Institute of Dental and Craniofacial Research, Clinical Dental Research Core, Bethesda, United States

## Abstract

**Introduction:**

A major challenge to HIV eradication strategies is accurate measurement of the latent HIV reservoir. We assessed whether the host response to residual virus may be a sensitive measure of reservoir size by comparing anti-HIV antibody profiles in relation to several HIV reservoir assays.

**Methods:**

Using a luciferase immunoprecipitation systems (LIPS) assay, we quantitatively analyzed seven anti-HIV antibody profiles from 61 patients who initiated long-term (≥3 years) antiretroviral therapy (ART) during chronic HIV infection. HIV antibody levels were evaluated in relation to 12 HIV reservoir measures: total, integrated and 2-LTR DNA (rtPCR, n=48); unspliced RNA (rtPCR, n=44), total and 2-LTR DNA (droplet digital PCR, n=27); integrated DNA (*alu*PCR, n=16); viral outgrowth assay (VOA, n=27) and plasma HIV RNA (single copy assay, SCA, n=27). Summary estimates of the overall association between HIV reservoir measures and HIV antibody levels adjusted for multiple comparisons were obtained using permutation testing.

**Results:**

Participants were mostly male (96%) with a median age of 56, median nadir and proximal CD4+ T cell counts of 210 and 670 cells/mm^3^, respectively, and ART-suppression for a median of 11 years. Individual correlations showed that integrated and total HIV DNA levels by *alu*PCR and ddPCR were significantly associated with all antibody levels except p24 (nor matrix, for ddPCR, Figure 1). HIV reservoir size measured by viral outgrowth assay (VOA) was associated with gp120 and gp41 levels (r=0.45, p=0.02; r=0.43, p=0.02) while HIV RNA by SCA and HIV DNA by rtPCR were not correlated with any HIV antibody responses. Permutation testing demonstrated a strong overall association between HIV reservoir size and anti-HIV antibody responses (r=0.82, p=0.04, Table 1), in particular with gp120 (r=0.80, p=0.009), gp41 (r=0.73, p=0.04) and reverse transcriptase (r=0.82, p=0.007). Further adjustment for age, proximal CD4+ T cell count and years of ART suppression did not significantly alter these results.

**Conclusions:**

Anti-HIV antibody responses correlate with quantifiable reservoir size during chronic ART-mediated suppression. Epitope location (envelope proteins and reverse transcriptase, an enzyme involved in the early steps of viral replication) may determine the strength of this association. Future studies are needed to evaluate whether viral RNA or proteins are produced in cells with defective proviruses.

**Abstract MOAA0103–Figure 1:** Individual correlations matrix.

**Abstract MOAA0103–Table 1:** Adjusted summary correlations

Anti-HIV antibody response	R	P
loggp120	0.80	0.009
loggp41	0.73	0.042
logrt	0.82	0.007
logintegrase	0.70	0.053
logpr	0.60	0.199
logma	0.54	0.340
logp24	0.41	0.679
All	0.82	0.039

# Sub article: Trancriptomics and metabolomics identify inflammatory profiles that segregate subjects with high and low inducible HIV reservoir

**DOI:** 10.7448/IAS.18.5.20323

**Published:** 2015-07-22

**Authors:** Khader Ghneim, Jeff Ahlers, Slim Fourati, Carey Shive, Mark Cameron, Pranab Mukerjee, Mahmoud Ghannoum, Benigno Rodriguez, Steven Deeks, Michael Lederman, Rafick Sekaly

**Affiliations:** 1 Department of Pathology, Case Western Reserve University, Cleveland, United States; 2 VGTI-FL, Port Saint Lucie, United States; 3 Department of Medicine, University of California San Francisco, San Francisco, United States

## Abstract

**Introduction:**

To identify mechanisms that control immune reconstitution and the size of the inducible HIV reservoir, we performed whole blood transcriptional and metabolic profiling of subjects from the CLIF and UCSF SCOPE cohorts. These cohorts included subjects who increased CD4 counts post cART (IR) or stayed <350/mm^3^ after three years of cART (INR).

**Methods:**

We performed unsupervised analysis of gene expression data using hierarchical clustering to identify class and supervised analysis using statistical filtering to identify gene signatures and pathway activity differentially expressed between classes. Multivariate analysis based on Sparse Partial Least Regression was used to determine if Group membership correlated with plasma metabolites measured by LC-MS/GC-MS. A gene-based classifier was developed to identify INR groups using the pamr package.

**Results:**

Two groups of INR subjects were identified by whole blood gene expression and pathway analysis. INR-A had the highest levels of IL-6, sCD14, FOXO3 and STAT1 expression, and highest levels of oxidative stress and mitochondrial dysfunction. Pathway analysis showed that INR-A failed to activate the NF-κB pathway, TLR-MyD88 signalling and proinflammatory modules yet upregulated expression of the p38 MAPK pathway, IRF-3, IRF-4 and IL-10 associated with a tolerogenic myeloid response. In contrast, INR-B was characterized by an unrestrained proinflammatory response including the upregulation of multiple TLRs, STAT1, IRF1 and IRF8 associated with Type I/II IFN responses. Plasma metabolites including carnitines, bacterial metabolites and cholesterol also segregated between the two INR groups and correlated with gene expression including FOXO3A and STAT-1. TILDA, a measure of the inducible HIV reservoir; revealed that INR-A subjects had higher levels than INR-B and IR's. As CD4 counts and plasma biomarkers of inflammation/immune activation fail to distinguish the two INR groups, we developed a 352 gene-based classifier that accurately identified patient groups (AUC of 0.81 by ROC analysis) in an independent test cohort (UCSF SCOPE) including those that had the highest levels of HIV reservoir.

**Conclusions:**

Identifying pathways that control immune reconstitution and the size of the inducible HIV reservoir paves the way to the development of therapeutic strategies that can lead to eradication of HIV.

# Sub article: HIV-1 virological remission for more than 11 years after interruption of early initiated antiretroviral therapy in a perinatally infected child

**DOI:** 10.7448/IAS.18.5.20566

**Published:** 2015-07-22

**Authors:** Pierre Frange, Albert Faye, Veronique Avettand-Fenoel, Erainna Bellaton, Diane Deschamps, Mathieu Angin, Sophie Caillat-Zucman, Gilles Peytavin, Jerome Le Chenadec, Josiane Warszawski, Christine Rouzioux, Asier Saez-Cirion, ANRS Epf-Co10 Pediatric Cohort

**Affiliations:** 1 Assistance Publique – Hôpitaux de Paris (AP-HP), Laboratoire de Microbiologie Clinique, Hôpital Necker – Enfants malades, Paris, France; 2 EA7327, Université Paris Descartes, Paris, France; 3 AP-HP, Unité d'Immunologie, Hématologie et Rhumatologie Pédiatriques, Hôpital Necker – Enfants Malades, Paris, France; 4 AP-HP, Service de Pédiatrie générale, Hôpital Robert Debré, Paris, France; 5 Université Paris 7 Denis Diderot, Paris, France; 6 AP-HP, Service d'Hématologie pédiatrique, Hôpital Robert Debré, Paris, France; 7 AP-HP, Hôpital Bichat – Claude Bernard, Paris, France; 8 INSERM UMR1137 IAME Université Paris Diderot, Paris, France; 9 Unite de HIV Inflammation et Persistance, Institut Pasteur, Paris, France; 10 AP-HP, Laboratoire d'Immunologie, Hôpital Robert Debré, Paris, France; 11 INSERM UMR1149, Université Paris Diderot, Paris, France; 12 AP-HP, Laboratoire de Pharma-Toxicologie, Hôpital Bichat, Paris, France; 13 IAME, INSERM UMR 1137, Université Paris Diderot, Paris, France; 14 AP-HP, Service d'Epidémiologie et de Santé publique, Hôpital Bicêtre, Le Kremlin-Bicetre, France; 15 INSERM U1018, Université Paris Sud, Le Kremlin-Bicetre, France

## Abstract

**Introduction:**

Durable HIV-1 remission after interruption of combined antiretroviral therapy (cART) has been reported in some adults who started cART during primary HIV-1 infection. The *in utero* HIV-1-infected “Mississippi child” exhibited transient viral control after interrupting very early-initiated cART. However, viraemia rebounded 27 months later, leaving unclear the possibility of obtaining long-term post-treatment remission in vertically infected children. Here, we report the case of a perinatally HIV-1-infected adolescent who shows unprecedented virological remission more than 11 years after cART discontinuation.

**Methods:**

HIV-RNA and CD4+ T-cell counts have been monitored since birth. Ultrasensitive HIV-RNA, peripheral blood mononuclear cell (PBMC)-associated HIV-DNA, flow-cytometry-assessed frequency of HIV-specific CD8+ T cells, CD8+ T-cell-mediated HIV suppression, reactivation of the CD4+ T-cell reservoir were evaluated after 10 and 11 years of control off therapy. Plasma concentrations of antiretrovirals were determined by tandem mass spectrometry.

**Results:**

One infant born for a woman with uncontrolled HIV-1 viraemia received zidovudine-based prophylaxis during six weeks. HIV-RNA and DNA were not detected 3 and 14 days after birth. HIV-DNA was detected at four weeks of age. HIV-RNA reached a peak of 2.1×10^6^ copies/mL at three months of age when cART (zidovudine, lamivudine, didanosine and ritonavir) was initiated. HIV-RNA was undetectable one month later and remained below assay-detection limits while on cART, except at 15 and 21 months of age. Between 5.8 and 6.8 years of age, cART was discontinued by the family. HIV-RNA was undetectable at 6.8 years of age and cART was not resumed. HIV-RNA has remained <50 copies/mL through 18.3 years of age, except for one blip (515 copies/mL). CD4+ T-cell counts remained stable. After 11 years of control off therapy (confirmed by undetectable plasma concentrations of antiretrovirals), HIV-RNA was below four copies/mL and HIV-DNA was 2.2 log copies/10^6^ PBMC. Low levels of HIV-RNA and p24 were detected upon the activation of CD4+ T cells with PHA. HLA genotype showed homozygosity at several loci (A*2301-;B*1503/4101;C*0210/0802;DRB1*1101-;DQB1*0602-). HIV-specific CD8+ T-cell responses and T-cell activation were very weak. HIV-1 western blot was positive with the absence of antibodies against gp110 and p18.

**Conclusions:**

This case provides first-time evidence that very long-term HIV-1 remission is possible in perinatally infected early-treated children, with similar characteristics as reported in adult post-treatment controllers.

# Sub article: Time-associated changes in cell-associated HIV RNA in HIV-infected subjects on suppressive antiretroviral therapy – implications for clinical trials of cure interventions

**DOI:** 10.7448/IAS.18.5.20567

**Published:** 2015-07-22

**Authors:** Christina Chang, Paul Cameron, Julian Elliott, Alan Perelson, Michael Roche, Ashanti Dantanarayana, Ajantha Solomon, Vivek Naranbhai, Surekha Tenakoon, Rebecca Hoh, James McMahon, Ken Sikaris, Wendy Hartogensis, Peter Bacchetti, Frederick Hecht, Jeffrey Lifson, Steve Deeks, Sharon Lewin

**Affiliations:** 1 Doherty Institute, The University of Melbourne, Melbourne, Australia; 2 Department of Infectious Diseases, Alfred Hospital, Melbourne, Australia; 3 Los Alamos National Laboratory, University of California, Los Alamos, United States; 4 Department of Medicine, The University of Oxford, Oxford, United Kingdom; 5 School of Medicine, University of California, San Francisco, San Francisco, United States; 6 Department of Pathology, Melbourne, Australia; 7 Division of Biostatistics, University of California, San Francisco, United States; 8 Center for Integrative Medicine, University of California, San Francisco, United States; 9 National Laboratory for Cancer Research, National Cancer Institute, Frederick, United States

## Abstract

**Introduction:**

Cell-associated unspliced (CA-US) HIV RNA is an important marker of the HIV reservoir and a common primary endpoint in clinical trials of latency reversing agents in HIV-infected subjects on antiretroviral therapy (ART). We observed large baseline variation in CA-US HIV RNA in a recent clinical trial of disulphiram and hypothesized that these changes were due to circadian-related alterations in CD4+ T-cell composition, gene regulation or anticipatory stress.

**Methods:**

Blood was collected on three occasions (B1, B2 and B3) from HIV-infected subjects (n=30) on suppressive ART prior to any intervention. B3 was collected immediately prior to administration of disulphiram. We measured CA-US HIV RNA and DNA by real-time PCR and plasma HIV RNA (using a single copy assay) by droplet digital PCR. Plasma cortisol and thyroid-stimulating hormone (TSH) levels were quantified by ELISA. PBMC were stained with live-dead dye and antibodies to CD3, CD4, CD8, CD45RA, CCR7, CD27, CD38, HLA-DR, acetylated lysine and acetylated histone-3 and were analyzed by flow cytometry. Data were assessed for normality and then analyzed with Wilcoxon matched-pairs signed rank tests and paired *t*-tests.

**Results:**

CA-US RNA was higher in blood collected at B3 compared to B1 and B2 (median 85.63 vs. 28.14 and 34.87 copies/million CD4+ T-cell equivalents; both, p<0.001). There were little differences in HIV DNA or plasma HIV RNA at these times. B3 was collected earlier in the day compared to B1 and B2 (mean 8.28 am vs. 11.38 am and 10.21 am; both, p<0.001). Other parameters that were significantly higher at B3 compared to B1 and B2 were cortisol (p=0.001 and 0.011); TSH (p=0.023 and 0.004); CD8+CD38+HLA-DR– T cells (both, p<0.001) and CD4+CD38+HLA-DR– T cells, which were elevated at B3 compared to B2 (p=0.012). There were no significant differences in the percentage of T-cell subsets or histone acetylation in the blood collected at these time points.

**Conclusions:**

Time-associated variation in CA-US HIV RNA seen in HIV-infected subjects on suppressive ART was not associated with significant alterations in CD4+ T-cell subset composition and was suggestive of circadian changes in HIV RNA transcription. Diurnal changes in CA-US HIV RNA may need to be considered in the design of future cure intervention trials.

# Sub article: Treatment with anti-α4β7 integrin antibody reduces virus-mediated gastrointestinal pathology by targeting distinct mucosal tissues

**DOI:** 10.7448/IAS.18.5.20324

**Published:** 2015-07-22

**Authors:** Siddappa Byrareddy, James Arthos, Claudia Cicala, Keith Reimann, Tristram Parslow, Philip Santangelo, Francois Villinger, Anthony Fauci, Aftab Ansari

**Affiliations:** 1 Department of Pathology & Laboratory Medicine, Emory University, Atlanta, United States; 2 Laboratory of Immunoregulation, National Institute of Allergy & Infectious Diseases, National Institutes of Health (NIH), Bethesda, United States; 3 Department of Mass Biologics, University of Massachusetts Medical School, Boston, United States; 4 Wallace H. Coulter Department of Biomedical Engineering, Georgia Institute of Technology and Emory University, Atlanta, United States

## Abstract

**Introduction:**

Our laboratory has recently demonstrated that *in vivo* administration of a monoclonal anti-α4β7 antibody (α4β7-mAb) during acute SIV infection following

(1) intravenous,

(2) intra-rectal or

(3) repeated low-dose intra-vaginal SIV challenge lead to markedly lower gastro-intestinal tissue viral loads compared to rhesus macaques (RM) treated with a control mAb.

The purpose of the present study was to compare the tissues that served as primary targets of viral infection in the α4β7-mAb versus control mAb-treated RM, in order to identify mechanisms by which α4β7-mAb antibody reduces virus-mediated gastrointestinal pathology.

**Methods:**

Groups of 12–16 RM were administered a rhesus α4β7-mAb monoclonal antibody or an isotype-matched control rhesus IgG mAb (50 mg/kg) intravenously (i.v.) starting on day -1 and then every three weeks after infection. Each monkey was then repeatedly challenged with a low-dose SIVmac251 intra-vaginally or a single high-dose intrarectally.

**Results:**

Intravenous administration of α4β7-mAb blocked the detection of α4β7 on CD4+ T cells in the blood, cervicovaginal tissue and gut-associated lymphoid tissue (GALT) throughout the period of mAb administration. Viral DNA was reduced in GALT biopsies of the α4β7-mAb treated RMs compared to those treated with control mAb treated (median 3.5 vs. 12.8 copies/ng DNA respectively, p=0.006). Furthermore, in-depth analysis performed on a subset of animals (n=4/group) indicated that proviral DNA was 5 to 25 fold more abundant in jejunum, ileum or colon of control-treated RMs compared to those treated with α4β7-mAb. In contrast, no difference in proviral loads in the spleen and lymph nodes from various sites was noted in the two groups. Immuno-PET/CT assisted analysis revealed that for animals with comparable plasma viral loads, the α4β7-mAb treated monkeys showed a lower signal in the large intestine. In addition, only the control treated monkeys showed a clear PET/CT signal in lymph nodes surrounding the genital tract suggesting that treatment with α4β7-mAb prevents viral replication in this tissue, leading to different patterns of tissue localization of the virus between the two groups.

**Conclusions:**

The α4β7-mAb either protects or delays intravaginal SIV transmission, reduces gastrointestinal pathology following infection and results in both quantitative and qualitative differences in the level of viremia and tissue localization of virus.

# Sub article: Oral microbiome in HIV-infected women: ageing, disease progression and opportunistic infections increase the pathogenic profile

**DOI:** 10.7448/IAS.18.5.20325

**Published:** 2015-07-22

**Authors:** Michael George, Barbara Weiser, Harold Burger, Tyler Lewy, Kathryn Anastos

**Affiliations:** 1 Department of Medical Microbiology and Immunology, University of California Davis School of Medicine, Davis, United States; 2 School of Medicine, University of California Davis, Davis, United States; 3 Sacramento Veterans Administration Medical Center, Sacramento, United States; 4 Department of Medicine, Albert Einstein College of Medicine, Yeshiva University, Bronx, United States

## Abstract

**Introduction:**

A recent marked increase in the proportion of HIV-infected individuals older than 50 highlights the need to study the impact of ageing on HIV pathogenesis. HIV-associated non-AIDS (HANA) conditions, such as cardiovascular disease, diabetes, osteoporosis and dementia are more prevalent in older HIV-infected populations than young adults. The microbiome in saliva and the oral cavity has been studied as a window into pathogenesis in ageing populations. Although disruption of the oral microbiome (dysbiosis) has been linked to various human conditions and diseases associated with ageing, the role of age-related dysbiosis in the development of opportunistic infections and HANA conditions in HIV patients is not well understood.

**Methods:**

We utilize 16S rRNA-based pyrosequencing to compare the salivary microbiome in three groups: chronically HIV-infected women enrolled in the Women's Interagency HIV Study who are

(1) >50 years old (ageing), or

(2) <35 years old (young adult) and

(3) healthy age-matched uninfected women.

We also examine correlations between dysbiosis of the salivary microbiome, disease progression and opportunistic oral infections.

**Results:**

HIV infection results in dysbiosis of the salivary microbiome that is enhanced in ageing individuals and characterized by increased abundance of pathogenic bacteria and a decline in healthy probiotic microbes. Higher proportions of *Prevotella*, *Staphylococcus, Moryella, Peptostreptococcus, Ruminococcus* and *Oribacterium* were detected in both ageing and young adult HIV infected women than in uninfected controls. *Prevotella*, *Moryella* and *Oribacterium* increases were higher in ageing than in young HIV patients. HIV infection in older patients was associated with greater salivary shedding of Epstein Barr Virus (EBV). Increased EBV shedding, higher peripheral HIV burden and reduced CD4+ T cell counts correlated with increases in *Prevotella* and decreases in probiotic *Lactobacillus*. Patients with opportunistic oral infections also showed enhanced salivary levels of *Porphyromonas, Lachnospira* and *Actinobacillus*, and reduced *Streptococcus*.

**Conclusions:**

Age, severity of disease progression and emergence of opportunistic infections all contribute to various degrees in increasing the pathogenic footprint of the oral microbiome during chronic HIV infection. The study findings provide new insights into age-related dysbiosis of the salivary microbiome and its role in HIV pathogenesis and lay critical groundwork for future expanded investigations.

# Sub article: Serum-derived bovine immunoglobulin isolate increases peripheral and mucosal CD4 T cell count in patients with HIV enteropathy

**DOI:** 10.7448/IAS.18.5.20326

**Published:** 2015-07-22

**Authors:** David Asmuth, Ma Somsouk, Peter Hunt, Zhong Min Ma, Christopher Miller, Xiao Dong Li, John Hinkle, Audrey Shaw, Eric Weaver, Gerald Klein

**Affiliations:** 1 Internal Medicine, University of California Davis Medical Center, Sacramento, United States; 2 Department of Medicine, University of California San Francisco, San Francisco, United States; 3 Center for Comparative Medicine, University of California Davis, Davis, United States; 4 Center for Comparative Medicine, Davis, United States; 5 California National Primate Research Center, Davis, United States; 6 EarlyPhase Sciences Inc., Cary, United States; 7 Entera Health Inc., Cary, United States; 8 Entera Health, Inc., Ankeny, United States; 9 Entera Health, Inc., Cary, United States

## Abstract

**Introduction:**

A multi-centre trial in HIV-enteropathy was conducted to evaluate the impact of serum-derived bovine immunoglobulin isolate (SBI) on markers of peripheral and mucosal immunity and gastrointestinal (GI) symptoms as previously reported.

**Methods:**

Patients (pts) on long-term suppressive antiretroviral treatment (ART) with HIV-enteropathy were randomized to receive SBI 2.5 vs. 5.0 g BID or placebo (PBO) during a four-week lead-in phase followed by SBI 2.5 vs. 5.0 g BID for 20 weeks. Evaluations included plasma biomarkers for inflammation, peripheral CD4 counts and pt-reported surveys on GI symptoms. Eight pts underwent duodenal biopsies to examine mucosal immunity.

**Results:**

A total of 103 pts (SBI 2.5 g; n=34; SBI 5.0 g; n=33; PBO: n=36 continued 2.5 vs. 5.0 g (n=18 each)) were enrolled (31% female; 61% black; mean age 51 years). Mean duration of HIV, ART and enteropathy was over 15, 5 and 5 years, respectively. All cohorts showed a reduction in abnormal stool frequency (p=0.0001) from baseline (BL) to week 4; however between group analysis was not significant. This reduction was maintained for pts receiving SBI through 24 weeks. The 2.5 and 5.0 g cohorts were combined for zonulin and CD4 analysis. The mean plasma zonulin levels significantly increased (p<0.0001) for pts receiving SBI through 24 weeks. Median peripheral CD4 counts increased significantly from BL to week 24 in patients in the lowest baseline CD4 quartile (308 to 386 cells/mL, p=0.002), while no significant change was observed among subjects in the combined SBI cohorts during this time period. This compromised subgroup also experienced greater increases in CD4 counts at week-4 than PBO pts (median +42 vs. −17 cells/mL, p=0.02). Duodenal CD4 densities increased from 217 to 329 cells/mm^2^ (median increase of 145 cells/mm^2^ (p=0.02)) in biopsies obtained from eight pts, consistent with earlier findings. Duodenal crypt cells expressing Ki67 decreased in 6/7 pts from 41 to 24% (p=0.08, n=7) which correlated with the decreased number of Paneth cells per crypt (p=0.048).

**Conclusions:**

Oral SBI may be a novel strategy to restore mucosal immunity and systemic immune reconstitution among pts who have not achieved normal CD4 counts despite prolonged suppressive ART.

# Sub article: HIV-exposure, gut microbiome, and vaccine responses in South African infants

**DOI:** 10.7448/IAS.18.5.20327

**Published:** 2015-07-22

**Authors:** Katie Viljoen, Jerome Wendoh, Ulas Karaoz, Eoin Brodie, Nicola Mulder, Gerrit Botha, Elvis Kidzeru, James Butcher, Clive Gray, Ken Rosenthal, Alash'le Abimiku, Bill Cameron, Alain Stintzi, Heather Jaspan

**Affiliations:** 1 Clinical Lab Sciences, Institute of Infectious Disease and Molecular Medicine, University of CapeTown, Cape Town, South Africa; 2 Lawrence Berkeley National Laboratory, Berkeley, United States; 3 Institute of Infectious Disease and Molecular Medicine, University of Cape Town, Cape Town, South Africa; 4 Ottawa Institute of Systems Biology, Ottawa, Canada; 5 Institute for Molecular Medicine and Health, McMaster University, Hamilton, Canada; 6 Institute of Human Virology, Baltimore, United States; 7 Ottawa Hospital Research Institute, Ottawa, Canada; 8 Seattle Children's Research Institute, Seattle, United States; 9 Department of Pediatrics, University of Washington, Global Health, Seattle, United States

## Abstract

**Introduction:**

The gut microbiome is crucial for mucosal and systemic immune development. In mice, certain bacteria are required for induction of Treg and Th17 cell development in the gut. Likewise, gut microbiota enhance immune responses to influenza vaccination in the mouse model. HIV-infected women have altered vaginal and gut microbiome, and HIV-exposed infants (HEU) and their mothers receive antibiotics for pneumocystis pneumonia prophylaxis, therefore HEU may have altered gut microbiota. HEU have higher morbidity and mortality than HIV-unexposed (HU) infants, and respond poorly to certain infant vaccinations. We hypothesized that the aetiology of this relative immune deficiency is mediated by gut dysbiosis.

**Methods:**

HEU and HU infants were recruited at birth from informal settlements of Cape Town. Blood and stool were collected after informed consent was obtained. Stool DNA was extracted using MoBio PowerFecal DNA kit and 454 or Illumina sequencing was performed. Data was pre-processed using QIIME and UPARSE and imported into R for further analyses using phyloseq. Differential abundance testing was performed at Operational Taxonomic Unit (OTU) level using the R metagenomeSeq package. Whole blood was incubated with BCG, positive and negative controls, and proliferation and cytokine expression measured using multi-parameter flow cytometry.

**Results:**

We found substantial differences in bacterial diversity between HEU and HU infants by Shannon index. Moreover, at all taxonomic levels, there were differences between the HIV exposure groups via PCoA analysis. Several OTUs of the phylum Firmicutes were differentially abundant between HEU and HU infants, three of which were of the genus Veillonella. Several key species were significantly correlated with both proliferative and cytokine responses to BCG. For example, at six weeks of age, significantly decreased abundance of *Bacteroides* species, and in particular *B. fragilis*, were present in infants with high CD4+IL-2+, CD8+ki67+ and CD8+IL-17+ responses to BCG vaccination at six weeks of age.

**Conclusions:**

Gut microbial composition could explain the immunological differences between HU and HEU infants. These differences should be considered in development of HIV vaccines for exposed neonates.

# Sub article: SIV-induced translocation of bacterial products in the liver mobilizes myeloid dendritic and natural killer cells associated with liver damage

**DOI:** 10.7448/IAS.18.5.20568

**Published:** 2015-07-22

**Authors:** Jamie Schafer, Tristan Evans, Haiying Li, R Keith Reeves

**Affiliations:** 1 Center for Virology and Vaccine Research, Beth Israel Deaconess Medical Center, Boston, United States; 2 New England Primate Research Center, Harvard University, Southborough, United States; 3 Center for Virology and Vaccine Research, Harvard Medical School/Beth Israel Deaconess Medical Center, Boston, United States

## Abstract

**Introduction:**

Disruption of the mucosal epithelium during immunodeficiency lentivirus infections permits translocation of microbial products into the circulation, causing systemic immune activation and driving disease progression. However, the specific effects of microbial products in liver, as a blood-filtering organ, are unclear.

**Methods:**

In this study, we investigated the effects of simian immunodeficiency virus (SIV) infection of rhesus macaques on microbial translocation in the liver by immunohistochemistry. We also compared liver infiltration by myeloid dendritic cells (mDCs), trafficking to the liver by lymphocytes, and liver-resident natural killer (NK) cell frequencies, phenotypes and functions in naïve and chronically SIVmac239- or SIVmac251-infected rhesus macaques using flow cytometry.

**Results:**

In livers of normal rhesus macaques, very low levels of bacteria and lipopolysaccharide (LPS) were detectable, but increased up to 20-fold in chronically SIV-infected animals. Increased microbial products in the liver of infected macaques was associated with the production of the chemoattractant, CXCL16, by mDCs. Subsequently, lymphocytes expressing the CXCL16 receptor, CXCR6, were mobilized in blood and hypercytotoxic NK cells were recruited to the liver. Microbial accumulation, mDC activation and hepatic cytotoxic NK cell frequency were all significantly correlated with markers of liver damage.

**Conclusions:**

Collectively, these data indicate that SIV-associated accumulation of microbial products in the liver initiates a cascade of innate immune activation resulting in liver damage. These findings have implications for the liver pathology associated with HIV, especially in instances of coinfection with HCV.

# Sub article: Field evaluation of point-of-care testing for early infant diagnosis in Cape Town, South Africa

**DOI:** 10.7448/IAS.18.5.20328

**Published:** 2015-07-22

**Authors:** Max Kroon, Lorna Dunning, Marvin Hsiao, Landon Myer

**Affiliations:** 1 Division of Neonatal Medicine, Department of Paediatrics & Child Health, University of Cape Town, Cape Town, South Africa; 2 Division of Epidemiology & Biostatistics, School of Public Health & Family Medicine, University of Cape Town, Cape Town, South Africa; 3 Division of Medical Virology, University of Cape Town, Cape Town, South Africa

## Abstract

**Introduction:**

Provision of rapid early infant HIV diagnosis (EID) service remains a challenge for prevention of mother-to-child transmission programmes globally. Point-of-care (POC) EID testing may improve access and turnaround times, but while several POC technologies are in development there are few data on implementation.

**Methods:**

We conducted an implementation study of the Alere q Detect POC system for EID at two public sector health facilities. At a maternity hospital the POC device was used to test HIV-exposed neonates soon after birth; at a primary care clinic the device was used for routine six-week EID testing. At each site infants undergoing laboratory-based HIV PCR testing per local protocols were tested on the POC device by doctors or nurses with results available within one hour. Analysis examined the performance of POC versus laboratory testing of the same specimen, and semi-structured interviews with providers to assess implementation issues and acceptability.

**Results:**

Overall 476 tests were conducted: 291 birth tests in the maternity hospital (mean child age, <1 day) and 195 six-week tests in primary care (mean child age, 51 days). Twelve percent of all tests resulted in an error with no differences by site; most error results resolved with retesting. POC EID was more sensitive (100%; lower confidence limit, 40%) and specific (100%, lower confidence limit, 98%) among older children tested in primary care compared birth testing in hospital (92% (95% CI, 62–100%) and 99% (95% CI, 99–100%), respectively), though test performance improved with repeated lab testing and negative predictive value was high (>99%) at both sites. In interviews, providers felt that the ease of use of the device coupled with the rapid turnaround time of POC EID results facilitated decision-making in the management of infants, but many wanted to understand better the cause of errors on the POC device to assist in repeat testing.

**Conclusions:**

POC EID testing performs well in field implementation in health care facilities and is highly acceptable to health care providers. While further research is needed to understand POC EID implementation at scale, the rapid turnaround time of POC testing may allow immediate identification and management of HIV-infected infants.

# Sub article: High rates of baseline NNRTI-resistance and virologic failure among ART naïve HIV-1-infected children in Mali

**DOI:** 10.7448/IAS.18.5.20329

**Published:** 2015-07-22

**Authors:** Claudia S Crowell, Almoustapha Issiaka Maiga, Mariam Sylla, Babafemi Taiwo, Niaboula Koné, Robert Leo Murphy, Anne-Genevieve Marcelin, Ban Traore, Djeneba Bocar Fofana, Ellen G Chadwick

**Affiliations:** 1 Department of Pediatric Infectious Diseases, University of Washington, Seattle Children's Hospital, Seattle, United States; 2 Faculty of Pharmacy, University of Sciences Techniques and Technologies of Bamako, Unité d'Epidémiologie Moléculaire de la Résistance du VIH aux ARV, SEREFO, Bamako, Mali; 3 Département de Pédiatrie, Centre Hospitalier Universitaire Gabriel Toure, Bamako, Mali; 4 Department of Infectious Diseases, Northwestern University, Chicago, United States; 5 Department of Virology, Hôpital Pitié-Salpêtrière, Paris, France; 6 Division of Pediatric Infectious Diseases, Lurie Children's Hospital, Chicago, United States

## Abstract

**Introduction:**

Limited data exist on drug resistance and antiretroviral treatment (ART) outcomes in HIV-1 infected children in West Africa. We determined the prevalence of baseline resistance, and correlates of virologic failure (VF) and on-treatment resistance in a cohort of HIV-1 infected children in Mali.

**Methods:**

Prospective observational study of HIV-1 infected children <10 years of age initiating first-line ART in Bamako, Mali. Assessments occurred at baseline and after six months of ART. Genotypic resistance testing on stored baseline and six-month samples occurred at study end. Reverse transcriptase and protease genes were sequenced using in-house methods. Resistance was defined as intermediate or high-level according to the Stanford HIV Genotypic Resistance Algorithm v7.0. Virologic failure was defined as viral load (VL) ≥1000 copies/mL. Clinical and immunological failures were based on WHO criteria. Logistic regression was used to evaluate factors associated with VF and resistance.

**Results:**

A total of 150 children were enrolled; 60% male and mean age 3.4 years. Ninety-four percent reported no prevention of mother-to-child transmission (PMTCT) exposure. Median baseline CD4 count and VL were 633 cells/mm3 (IQR: 381–1039) and 675,651 copies/mL (IQR: 40,000–1,583,200). Initial ART regimens were lopinavir/ritonavir-based (43%) or non-nucleoside reverse transcriptase inhibitors (NNRTI) (efavirenz or nevirapine)-based (57%). Of 141 children with amplifiable baseline samples, 28 (19.86%) had NNRTI resistance, only two of whom had PMTCT exposure and none had protease inhibitor (PI) resistance. Mean age of children with baseline NNRTI resistance was 2.3 years. By six months of ART, 11 died, 8 were lost to follow-up and 6 had missing VL data. Among 125 remaining children, 41 (33%) had VF, 24 of whom (58%) had drug resistance (23 with NNRTI and one with PI mutations). A total of 93% of children with VF did not meet criteria for clinical or immunological failure. In multivariate analyses adjusting for age, gender, adherence and ART regimen, baseline NNRTI resistance was strongly associated with VF and six-month resistance (OR: 6.7, p=0.001; OR: 20, p<0.001).

**Conclusions:**

Baseline NNRTI resistance was common in Malian children without prior NNRTI exposure and was associated with VF and a high resistance rate during ART. Clinical and immunologic criteria rarely detected VF. Our findings support WHO recommendations of PI-based regimens in all children <3 years, and virological monitoring.

# Sub article: T cell activation and treatment outcomes among infants receiving early ART

**DOI:** 10.7448/IAS.18.5.20330

**Published:** 2015-07-22

**Authors:** Kristjana Hrönn Ásbjörnsdóttir, Jennifer A Slyker, Dalton Wamalwa, Stephen De Rosa, James P Hughes, Ali Rowhani-Rahbar, Bhavna H Chohan, Sarah Benki-Nugent, Kenneth Tapia, Grace C John-Stewart

**Affiliations:** 1 Department of Epidemiology, University of Washington, Seattle, United States; 2 Department of Global Health, University of Washington, Seattle, United States; 3 Department of Paediatrics and Child Health, University of Nairobi, Nairobi, Kenya; 4 Laboratory Medicine, University of Washington, Seattle, United States; 5 Center for AIDS Research Immunology Core, University of Washington, Seattle, United States; 6 Fred Hutchinson Cancer Research Center, Vaccine and Infectious Disease Division, Seattle, United States; 7 Department of Biostatistics, University of Washington, Seattle, United States; 8 Department of Medical Microbiology, University of Nairobi, Nairobi, Kenya; 9 Center for AIDS Research Biometrics Core, University of Washington, Seattle, United States; 10 Department of Pediatrics and Medicine, University of Washington, Seattle, United States

## Abstract

**Introduction:**

Chronic immune activation is associated with HIV disease progression in adults; however, data in children, especially infants, are limited. We determined levels and correlates of T-cell activation and the effect of baseline activation on response to antiretroviral treatment (ART) in HIV-infected infants.

**Methods:**

This investigation utilized specimens from the Optimizing Pediatric HAART study of early infant ART (NCT00428116). Kenyan infants less than five months of age were enrolled between 2007 and 2010 and started on ART. Peripheral blood mononuclear cell (PBMC) samples collected before ART initiation were analyzed using flow cytometry and the activated (HLA-DR+/CD38^high^) T-cell percentage quantified. Factors associated with T-cell activation at baseline were identified using Mann-Whitney U tests or linear regression. The effect of baseline activation on survival, CD4 reconstitution and HIV-1 log_10_ viral load (VL) suppression was assessed using Cox proportional hazard models.

**Results:**

Among 72 infants, median age at enrolment was 111 days, median VL was 6.6 log_10_ copies/mL and median CD4 was 19%. Most infants had symptomatic disease; 49% were WHO stage 3/4, median weight-for-age Z-score (WAZ) was −2.5 and median length-for-age Z-score (LAZ) was −2.1. Twenty infants died, including eight before ART initiation. Median CD8+ T-cell activation at baseline pre-ART was 17.0% (interquartile range (IQR) 10.4, 31.8) and median CD4+ T-cell activation was 3.3% (IQR 1.6, 5.8). At enrolment, CD8+ T-cell activation was associated with younger age (−0.15%/day (95% Confidence Interval (CI) −0.28, −0.01), p=0.05) and weight-for-age Z-score (2.4%/WAZ standard deviation (95% CI 0.64–4.2), p=0.02), but not with CD4% or VL. CD4+ T-cell activation at enrolment was inversely associated with CD4% (−0.20%/CD4% (95% CI −0.36, −0.05), p=0.01). T-cell activation pre-ART was not associated with time to CD4% reconstitution or VL suppression. Low CD8+ T-cell activation (<5%) was associated with mortality (hazard ratio=3.8 (95% CI 1.3, 11.4), p=0.02).

**Conclusions:**

Contrary to findings in adults, low CD8+ T-cell activation was strongly associated with mortality in this infant cohort. Among infants, low CD8+ T-cell activation in symptomatic HIV infection may be a marker of ineffective immune response.

**Abstract MOAB0103–Figure 1:** Survival to one year by CD8+ T-cell activation.

# Sub article: Changes in renal laboratory parameters and bone mineral density in treatment-naïve HIV-1-infected adolescents initiating therapy with INSTI-based single-tablet regimens containing tenofovir alafenamide or tenofovir disoproxil fumarate

**DOI:** 10.7448/IAS.18.5.20331

**Published:** 2015-07-22

**Authors:** Hilda Kizito, Aditya Gaur, Wasana Prasitsuebsai, Natella Rakhmanina, Kulkanya Chokephaibulkit, Jan Fourie, Linda-Gail Bekker, Yongwu Shao, Sean Bennett, Erin Quirk

**Affiliations:** 1 Joint Clinical Research Centre, Kampala, Uganda; 2 St. Jude Children's Research Hospital, Memphis, United States; 3 HIVNAT, Bangkok, Thailand; 4 Children's National Health System, Washington, United States; 5 Siriraj Hospital, Bangkok, Thailand; 6 Mpati Medical Centre, Dundee, South Africa; 7 Desmond Tutu HIV Centre, Cape Town, South Africa; 8 Gilead Sciences, Inc., Foster City, United States

## Abstract

**Introduction:**

EVG/COBI/FTC/TAF (E/C/F/TAF) and EVG/COBI/FTC/TDF (Stribild, STB) are integrase inhibitor (INSTI)-based single-tablet regimens (STRs) in clinical development for HIV-1-infected adolescents. Exposures of all components have been shown to be within the range associated with antiviral activity in adults. Preliminary comparative safety data through 24 weeks are reported.

**Methods:**

Treatment-naïve 12 to <18-year-olds weighing ≥35 kg with HIV-1 RNA ≥1000 copies/mL, CD4 >100 cells/µL and eGFR ≥90 mL/min/1.73 m^2^ received E/C/F/TAF or STB once daily in two ongoing 48-week, single-arm, open-label trials. Adverse events (AE), laboratory tests, bone mineral density (BMD) by dual X-ray absorptiometry and height-age adjusted (HA) Z-scores were assessed through Week 24.

**Results:**

The E/C/F/TAF and STB trials enrolled 50 and 33 adolescents, respectively (median age 15 vs. 16 years, 56% vs. 30% female, 88% vs. 76% Black, 22% vs. 27% with baseline HIV-1 RNA >100,000 copies/mL, median CD4 count 456 vs. 407 cells/µL median eGFR 156 vs. 143 mL/min/1.73 m^2^). Most AEs in both trials were mild and unrelated to treatment, with no deaths or AEs leading to treatment discontinuation. At Week 24, the median increase in serum creatinine was +0.08 mg/dL in E/C/F/TAF participants, with and +0.10 mg/dL in STB participants, with median eGFR decreases of −17.0 and −18.0 mL/min/1.73 m^2^, respectively, consistent with COBI's inhibition of renal tubular creatinine secretion. Proteinuria (any grade) occurred in 26% of E/C/F/TAF participants vs. 52% of STB participants, with Grade 2 or higher proteinuria occurring in 4% vs. 21% of participants, respectively. Of those participants with BMD measurements at Week 24, the median increase in spine BMD was +1.98% in E/C/F/TAF participants, with a decrease of ≥4% in 3/41 participants (7%), versus a median decrease of −1.29% in the STB cohort, with a decrease of ≥4% in 6/20 participants (30%). Spine HA Z-scores decreased by −0.02 and −0.21 respectively.

**Conclusions:**

Compared with STB, E/C/F/TAF exhibited similar effects on eGFR, a lower incidence and severity of proteinuria, and a median increase in spine mineralization. Both STRs were well-tolerated through 24 weeks. These findings support INSTI-based STRs as initial HIV-1 treatment in adolescents and suggest that TAF could offer safety advantages in paediatric populations.

# Sub article: Treatment and resistance outcomes of Asian children on second-line antiretroviral therapy

**DOI:** 10.7448/IAS.18.5.20332

**Published:** 2015-07-22

**Authors:** Wasana Prasitsuebsai, Sirinya Teeraananchai, Khanh Huu Truong, Jintanat Ananworanich, Viet Chau Do, Lam Van Nguyen, Pope Kosalaraksa, Nia Kurniati, Tavitiya Sudjaritruk, Kulkanya Chokephaibulkit, Thida Singtoroj, Stephen J Kerr, Annette H Sohn, TASER-Pediatrics Study

**Affiliations:** 1 HIV-NAT, Thai Red Cross – AIDS Research Centre, Bangkok, Thailand; 2 Children's Hospital 1, Ho Chi Minh City, Vietnam; 3 HIV-NAT, SEARCH, Thai Red Cross – AIDS Research Centre, Bangkok, Thailand; 4 U.S. Military HIV Research Program, Walter Reed Army Institute of Research, Silver Spring; 5 Henry M. Jackson Foundation for the Advancement of Military Medicine, Bethesda, United States; 6 Children's Hospital 2, Ho Chi Minh City, Vietnam; 7 National Hospital of Pediatrics, Hanoi, Vietnam; 8 Division of Infectious Diseases, Department of Pediatrics, Khon Kaen University, Khon Kaen, Thailand; 9 Cipto Mangunkusumo General Hospital, Jakarta, Indonesia; 10 Faculty of Medicine and Research Institute for Health Sciences, Chiang Mai University, Chiang Mai, Thailand; 11 Faculty of Medicine Siriraj Hospital, Mahidol University, Bangkok, Thailand; 12 TREAT Asia/amfAR, Foundation for AIDS Research, Bangkok, Thailand; 13 Amsterdam Institute of Global Health and Development (AIGHD), Amsterdam, The Netherlands

## Abstract

**Introduction:**

With limited paediatric third-line antiretroviral therapy (ART) in resource-limited settings, data on treatment efficacy and drug resistance following second-line failure are needed to guide future management.

**Methods:**

HIV-infected children <18 years old who were taking or switching to second-line ART were enrolled from Indonesia, Thailand and Vietnam. Clinical and laboratory assessments were retrospectively and prospectively obtained from the time of second-line switch (baseline). Genotyping was performed upon virologic failure (VF; HIV-RNA >1000 copies/mL). Cox proportional hazards regression was used to evaluate factors predicting post-switch VF.

**Results:**

A total of 277 children were enrolled; 41% were female. Baseline values included median (interquartile range; IQR) age 7.5 (5.3–10.3) years, CD4 count 300 (146–562) cells/mm^3^, CD4 percentage 13 (7–20)%, HIV-RNA 5 (4.4–5.5) log_10_ copies/mL. The median duration of first-line ART was 2.7 (1.7–4.2) years. Resistance mutations at first-line failure were available for 156 of 277 children (all had prior non-nucleoside reverse transcriptase (NNRTI)-based regimens) and included ≥4 thymidine analogue mutations (TAMs; 18%), Q151 M (8%), M184 V (82%) and ≥1 NNRTI mutation (92%). Current second-line regimens contained lamivudine (90%), tenofovir (43%), zidovudine or abacavir (30%) and boosted lopinavir (LPV) or atazanavir (ATV; 98%). After a median of 3.3 (1.8–5.3) years on second-line, the median CD4 was 767 (556–1060) cells/mm^3^ and 26 (20–31)%. Eighteen (7%) had WHO stage 3 or 4 events; 3 (2%) died from HIV-related illnesses. VF occurred in 73 (27%; incidence 7 per 100 person-years, 95% confidence interval (CI) 5.8–9.1), at which time 23% had <95% adherence by pill count. Fifty of 73 with second-line VF had ≥4 TAMs (10%), Q151 M (4%), M184 V (55%), and ≥1 major LPV (8%), ≥6 LPV (2%), and ≥1 major ATV mutations (4%). Age >11 years (hazard ratio (HR) 4.06; 95% CI 2.15–7.66) and HIV-RNA >5 log_10_ copies/mL (HR 2.4; 95% CI 1.27–4.59) at second-line switch were predictors of VF.

**Conclusions:**

One-fourth of children had VF while on second-line ART. However, <10% developed major mutations to protease inhibitors, which may have been related to poor adherence or duration of VF. Greater advocacy is needed to create access to third-line antiretrovirals in resource-limited settings.

# Sub article: Week 48 safety and efficacy of a rilpivirine (TMC278)-based regimen in HIV-infected treatment-naïve adolescents: PAINT phase II trial

**DOI:** 10.7448/IAS.18.5.20333

**Published:** 2015-07-22

**Authors:** Johan Lombaard, Torsak Bunupuradah, Patricia Flynn, John Ramapuram, Francis Ssali, Herta Crauwels, Annemie Hoogstoel, Veerle Van Eygen, Marita Stevens, Katia Boven

**Affiliations:** 1 Josha Research, Bloemfontein, South Africa; 2 Thai Red Cross AIDS Research Centre, Bangkok, Thailand; 3 St. Jude Children's Research Hospital, Memphis, United States; 4 Kasturba Medical College Hospital, Mangalore, India; 5 Joint Clinical Research Centre, Kampala, Uganda; 6 Janssen Infectious Diseases BVBA, Beerse, Belgium; 7 Janssen Research & Development LLC, Titusville, United States

## Abstract

**Introduction:**

Rilpivirine 25 mg qd exposure was similar in adults and adolescents (Week 4 PAINT pharmacokinetic analysis). Week 48 safety and efficacy results are reported here.

**Methods:**

PAINT (NCT00799864) is a Phase II, ongoing, open-label, single-arm trial of rilpivirine plus two investigator-selected N[t]RTIs in treatment-naïve HIV-1-infected adolescents (≥12 to <18 years, from sites in India, Thailand, Uganda, South Africa, USA). After the adult approved indication, only patients with viral load (VL)≤100,000 copies/mL were enrolled. Virologic response was defined as VL<50 copies/mL (time-to-loss-of-virologic-response (TLOVR) algorithm).

**Results:**

Of 36 patients, 20 (56%) were female, 18 (50%) aged 12 to <15 years and 32 (89%) Black/African American; 28 (78%) had baseline (BL) VL ≤100,000 copies/mL; 24 (67%) received emtricitabine/tenofovir disoproxyl fumarate (TDF), 8 (22%) lamivudine/TDF and 4 (11%) lamivudine/zidovudine.

At Week 48, 26/36 (72%) patients overall, 22/28 (79%) with BLVL ≤100,000 copies/mL and 4/8 (50%) with BLVL >100,000 copies/mL achieved virologic response. Of the 10 non-responders (28%), eight were virologic failures (VFs), one was dosed although a protocol violator (screening NNRTI RAM) and withdrawn and one withdrew due to an AE (pulmonary tuberculosis). CD4^+^ count increased by median (range) 250.5 (−135 to 740) cells/mm^3^.

For 2/8 VFs, overall adherence (pill count) was <95% (one of these also had BLVL >100,000 copies/mL). Five of eight VFs developed rilpivirine RAMs, mostly E138K (n=4), K101E (n=2) and M230L (n=2); 4/5 developed N[t]RTI RAMs, mostly M184V (n=3).

Mean (standard deviation) rilpivirine AUC_24h_ and C_0h_ were 2391 (991) ng.h/mL and 84 (39) ng/mL, respectively (population pharmacokinetic analysis). Most AEs were grade 1 or 2. Seven patients (19%) had grade 3 or 4 AEs regardless of causality, mainly malaria and depression (each n=2 and not related to rilpivirine). AEs considered at least possibly related to rilpivirine occurred in 13 (36%) patients, mainly (excluding investigations) somnolence (n=5, 14%) and nausea (n=2, 6%).

**Conclusions:**

This 48-week analysis supports use of rilpivirine 25 mg qd combined with other antiretrovirals in treatment-naïve HIV-1-infected adolescents (≥12 to <18 years) with VL≤100,000 copies/mL. Rilpivirine safety, virological and pharmacokinetic results were similar to those observed in adults.

# Sub article: *In utero* tenofovir exposure is not associated with foetal long bone growth

**DOI:** 10.7448/IAS.18.5.20480

**Published:** 2015-07-22

**Authors:** Jennifer Jao, Landon Myer, Tamsin Phillips, Greg Petro, Allison Zerbe, Elaine J Abrams

**Affiliations:** 1 Department of Medicine, Icahn School of Medicine at Mount Sinai, New York, United States; 2 Department of Obstetrics, Gynecology, and Reproductive Science, Icahn School of Medicine at Mount Sinai, New York, United States; 3 Division of Epidemiology and Biostatistics, Desmond Tutu HIV Centre, University of Cape Town, Cape Town, South Africa; 4 Division of Epidemiology and Biostatistics, University of Cape Town, Cape Town, South Africa; 5 Division of Obstetrics & Gynaecology, University of Cape Town, Cape Town, South Africa; 6 ICAP, Columbia University, New York, United States

## Abstract

**Introduction:**

Despite widespread use of tenofovir (TDF) in pregnant and breast-feeding women, few data have been published on foetal bone development or child growth after *in utero* TDF exposure.

**Methods:**

We evaluated foetal long bone measurements in HIV-infected pregnant woman/foetus dyads in Cape Town, South Africa. Measurements were conducted by a trained research sonographer using high-resolution ultrasound. Foetal femur (FLZ) and humerus (HLZ) length *z*-scores were compared by duration of *in utero* TDF exposure in three categories: 1) TDF-exposed since conception (TDF-C) versus 2) TDF-exposed for ≥4 weeks and initiated after first trimester (TDF-E), versus 3) TDF-exposed for <4 weeks or TDF-unexposed (TDF-U). Ultrasounds performed at <10 weeks gestational age (GA), twin pregnancies and those resulting in intrauterine foetal demise were excluded. Linear mixed effects models were used to assess the effect of duration of TDF exposure category on FLZ and HLZ.

**Results:**

A total of 1957 foetal ultrasounds (408 TDF-C, 581 TDF-E, 968 TDF-U) in 1030 women (73% of whom had ≥2 ultrasounds) were available for analysis. Women in the TDF-C group were older and had lower CD4 cell counts than women in the other categories but did not differ in anthropometry or history of low birth weight deliveries (Table). Median duration of TDF exposure was 26.9, 13.0 and 0 weeks, respectively, in the TDF-C, TDF-E and TDF-U groups. Mean FLZ and HLZ did not differ by TDF exposure category (FLZ: 0.321 vs. 0.300 vs. 0.333, p=0.570, and HLZ: 0.130 vs. 0.318 vs. 0.048, p=0.832). These relationships persisted after adjusting for maternal age, gestation, gravidity, socioeconomic status, CD4 cell count, HIV RNA level and maternal BMI (β=0.038, p=0.563 for TDF-C vs. TDF-U and β=−0.002, p=0.964 for TDF-E vs. TDF-U foetal FLZ; β=0.009, p=0.903 for TDF-C vs. TDF-U and β=−0.006, p=0.885 for TDF-E vs. TDF-U foetal HLZ). No other factors related to HIV disease severity were associated with foetal FLZ or HLZ.

**Conclusions:**

*In utero* TDF exposure does not appear to alter foetal long bone growth. These results are reassuring and support the continued use of TDF in HIV-infected pregnant women.

**Abstract MOAB0107LB–Table 1:** Characteristics of women and foetal ultrasound measurement

Characteristics of pregnant women	TDF-exposed since conception (n=226)	TDF-exposed for >4 weeks and initiated after first trimester (n=232)	TDF-exposed for <4 weeks or TDF-unexposed (n=572)	p
Age of mother, years	31 (27–34)	27 (23–32)	28 (25–32)	<0.001
GA, weeks	20 (14–28)	21 (14–28)	21 (16–27)	0.805
Maternal BMI at enrolment, kg/m^2^	29.14 (25.81–33.91)	28.50 (25.00–33.75)	28.63 (25.15–34.24)	0.790
CD4 cell count, cells/mm^3^	399 (273–523)	360 (239–478)	340 (232–507)	0.015
Log HIV RNA level at enrolment	1.59 (1.59–1.59)	4.13 (3.52–4.57)	3.99 (3.37–4.65)	<0.001
Number of women with >2 ultrasound scans	147 (64.8)	126 (54.3)	479 (83.6)	0.001
Characteristics of ultrasound scans	(n=408)	(n=581)	(n=968)	p value
Femur length z score	0.32 (−0.03, 0.70)	0.30 (−0.03, 0.63)	0.33 (−0.07, 0.79)	0.570
Humerus length z score	0.13 (−0.29, 0.59)	0.32 (−0.04, 0.59)	0.05 (−0.33, 0.46)	0.832

# Sub article: The durability of isoniazid preventive therapy for tuberculosis: long-term follow-up from a prospective cohort of HIV-infected adults in South Africa

**DOI:** 10.7448/IAS.18.5.20334

**Published:** 2015-07-22

**Authors:** Colleen Hanrahan, Neil Martinson, Grace Link-Barnes, Reginah Msandiwa, Richard Chaisson, Jonathan Golub

**Affiliations:** 1 Johns Hopkins Bloomberg School of Public Health, Baltimore, United States; 2 Perinatal HIV Research Unit, University of the Witwatersrand, Johannesburg, South Africa; 3 Center for TB Research, Johns Hopkins School of Medicine, Baltimore, United States

## Abstract

**Introduction:**

Isoniazid preventive therapy (IPT) has been demonstrated to reduce the risk of active tuberculosis (TB) in HIV-infected adults, but the effectiveness of shorter IPT regimens (6–9 months) rapidly wanes in high TB burden settings. We examined the long-term durability of six months of IPT among HIV-infected adults in South Africa.

**Methods:**

We analyzed the experience of a prospective clinical cohort of HIV-infected adults at one urban and one rural hospital in South Africa. The exposures of interest were receipt of IPT and antiretroviral therapy (ART), and the primary outcome was incident TB. We used multivariate Poisson regression to examine the association of IPT and ART with risk of TB.

**Results:**

From 2003 to 2010, 3465 HIV-infected adults were followed for 9908 person-years (PY) during which 372 incident TB cases were diagnosed (incidence rate (IR): 3.8/100PY; 95% CI: 3.4–4.2). A total of 776 participants received IPT (median treatment length: 5 months (IQR: 2–6)). During 1886 PY of follow-up after initiating IPT, 54 incident cases of TB were diagnosed (IR: 2.9/100 PY; 95% CI: 2.2–3.7), while during 8022 PY of follow-up without IPT exposure, 318 incident TB cases were diagnosed (IR: 4.0/100 PY; 95% CI: 3.6–4.4).

After adjusting for age, sex, study site, ART use and CD4 count, IPT was associated with a 23% reduction in TB incidence over seven years of follow-up (adjusted IRR: 0.77; 95% CI: 0.7–1.0; p=0.070). IPT appeared to be protective only for the first year following initial IPT exposure (aIRR: 0.46; 95% CI: 0.36–0.98; p=0.042), after which the risk of TB was not significantly reduced.

**Conclusions:**

In this prospective cohort of HIV-infected adults in South Africa, receipt of six months of IPT resulted in a marked (40%) reduction in risk for TB during the first year following IPT initiation, independent of ART status. No reduction in TB risk was observed beyond one year, confirming similar findings in settings of high TB burden. We demonstrate that IPT remains an important intervention for HIV-infected individuals, and that even a short regimen can provide crucial protection from TB of up to one year for those not yet initiated on highly active antiretroviral therapy.

**Abstract MOAB0201–Table 1:** Incidence of TB by time following IPT exposure

	IPT unexposed (n=2689)		IPT exposed (n=776)		Incidence rate ratio (95% CI)			
			
Time interval	Cases/PY	Rate/100 PY	Cases/PY	Rate/100 PY	Unadjusted	p	Adjusted	p
Overall	318/8022	4.0 (3.6–4.4)	54/1886	2.9 (2.2–3.7)	0.72 (0.53–0.99)	0.042	0.77 (0.57–1.0)	0.070
0–1 year	151/2672	5.7 (4.8–6.6)	16/615	1.6 (1.6–4.2)	0.46 (0.26–0.80)	0.006	0.60 (0.36–0.98)	0.042
≥1–2 years	75/2138	3.5 (2.8–4.4)	20/519	3.9 (2.5–6.0)	1.1 (0.63–1.9)	0.740	1.1 (0.67–1.8)	0.733
≥2–3 years	47/1362	3.5 (2.6–4.6)	10/301	3.3 (1.8–6.2)	0.96 (0.50–1.9)	0.880	0.95 (0.51–1.8)	0.880
≥3 years	45/1851	2.4 (18–3.3)	8/451	1.8 (0.89–3.7)	0.73 (0.30–1.6)	0.413	0.73 (0.34–1.6)	0.394

**Abstract MOAB0201–Figure 1:** Time to incident TB by IPT and HAART exposure.

# Sub article: Treatment outcomes of drug-resistant TB patients in South Africa, disaggregated by HIV status, as reported in a national electronic drug-resistant TB register

**DOI:** 10.7448/IAS.18.5.20335

**Published:** 2015-07-22

**Authors:** Denise Hilary Evans, Kate Schnippel, Eric Budgell, Kate Shearer, Rebecca Berhanu, Lawrence Long, Sydney Rosen

**Affiliations:** 1 Health Economics and Epidemiology Research Office, Department of Internal Medicine, School of Clinical Medicine, Faculty of Health Sciences, University of the Witwatersrand, Johannesburg, South Africa; 2 Right to Care, Johannesburg, South Africa; 3 Center for Global Health & Development, Boston University School of Public Health, Boston, United States

## Abstract

**Introduction:**

South Africa reports the third highest number of drug-resistant TB (DR-TB) cases and the largest population living with HIV in the world. We describe treatment outcomes of patients from the South African Electronic Drug Resistant Tuberculosis Register (EDRweb), the national database of all DR-TB cases, after January 2009.

**Methods:**

Retrospective, de-identified descriptive analysis of all patients with multidrug resistant (MDR) TB who initiated DR-TB treatment in South Africa between 01/01/09 and 30/09/11. During this period, guidelines specified all MDR-TB patients were admitted to specialized referral hospitals for the six-month intensive phase of treatment or until culture conversion, then followed as outpatients for 12–18 months. Treatment outcomes included success (cured and treatment completed), failed, lost to follow-up and died. Person-time accrued from treatment initiation until the earliest of outcome date recorded or 24 months on treatment. Cox hazard models were used to evaluate the relationship between HIV status and all-cause mortality. Models were adjusted for age, gender and previous history of TB treatment.

**Results:**

In total, 13,692 confirmed MDR-TB patients initiated treatment (median age 35.4 years; 53% male; 99% pulmonary TB). Eighty-one percent (11,028/13,692) had HIV status recorded; of these 66% (7289/11,028) were co-infected with HIV.

Among those with an outcome reported (8465/13,692; 62%), overall mortality and success rates were 24.6% (95% CI 23.7–25.6) and 42.4% (41.3–43.4), respectively. Success was similar between HIV negative patients (42.9% (41.0–44.8); 18.1/100 person-years (pys)) and those co-infected with HIV (42.5% (41.0–43.9); 15.5/100 pys).

Mortality was substantially higher in the HIV positive (27.3% (26.0–28.6); 10.1/100 pys) than the HIV negative (17.3% (15.9–18.7); 8.0/100 pys) group (adjusted hazard ratio 1.45 (1.30–1.62)). Fewer HIV positive patients were lost to follow-up or failed treatment compared to HIV negatives (21.6 and 8.6% vs. 29.0 and 10.8%).

**Conclusions:**

In this analysis of the outcomes of MDR-TB treatment in the South African national database, the reported rate of treatment success was low (42%) and did not vary by HIV status. Mortality was high in both groups but almost 1.5 times more in HIV co-infected patients. New guidelines allowing decentralized (outpatient) treatment of some MDR-TB patients and newly available drug regimens may improve treatment results.

**Abstract MOAB0202–Table 1:** Summary of treatment outcomes of MDR-TB patients in South Africa (n=13,692)

		Treatment success					
		
	Total	Cured	Completed treatment	Died	Failed treatment	Lost to follow-up	Not evaluated
HIV status							
HIV −ve	3739 (27.3%)	803 (21.5%)	341 (9.1%)	460 (12.3%)	289 (7.7%)	773 (20.7%)	1073 (28.7%)
HIV +ve	7289 (53.2%)	1356 (18.6%)	576 (7.9%)	1243 (17.1%)	390 (5.4%)	985 (13.5%)	2739 (37.5%)
Unknown	2664 (19.5%)	328 (12.3%)	184 (6.9%)	383 (14.4%)	118 (4.4%)	236 (8.9%)	1415 (53.1%)
TB treatment history							
New	3250 (23.7%)	653 (20.1%)	291 (9.0%)	439 (13.5%)	164 (5.0%)	476 (14.6%)	1227 (37.8%)
Relapse	2351 (17.2%)	436 (18.5%)	177 (7.5%)	328 (14.0%)	139 (5.9%)	331 (14.1%)	940 (40.0%)
LTF^a^	1232 (9.0%)	132 (10.7%)	80 (6.5%)	274 (22.2%)	99 (8.1%)	286 (23.2%)	361 (29.3%)
Failed 1^st^	3959 (28.9%)	735 (18.6%)	333 (8.4%)	567 (14.3%)	186 (4.7%)	485 (12.3%)	1653 (41.7%)
Failed 2^nd^	2516 (18.4%)	495 (19.7%)	197 (7.8%)	415 (16.5%)	180 (7.2%)	352 (14.0%)	877 (34.8%)
Other	384 (2.8%)	36 (9.3%)	23 (6.0%)	63 (16.4%)	29 (7.6%)	64 (16.7%)	169 (44.0%)

a LTF lost to follow-up; 1^st^ first-line; 2^nd^ second-line.

# Sub article: Excess TB mortality in HIV patients in Eastern Europe: restructured approach to care needed

**DOI:** 10.7448/IAS.18.5.20336

**Published:** 2015-07-22

**Authors:** Anna Schultze, Anne Marie Werlinrud Efsen, Frank A Post, Alexander Panteleev, Hansjakob Furrer, Robert Miller, Marcelo H Losso, Javier Toibaro, Aliaksandr Skrahin, Jose M Miro, Joan A Caylà, Enrico Girardi, Mathias Bruyand, Niels Obel, Daria N Podlekareva, Jens D Lundgren, Amanda Mocroft, Ole Kirk, on behalf of the TB: HIV Study Group in EuroCoord

**Affiliations:** 1 Department of Infection and Population Health, University College London, London, United Kingdom; 2 Department of Infectious Diseases and Rheumatology, CHIP, Finsencentret, Rigshospitalet, University of Copenhagen, Copenhagen, Denmark; 3 Department of Sexual Health, King's College Hospital, Caldecot Centre, London, United Kingdom; 4 Department of HIV/TB, TB Hospital 2, St. Petersburg, Russian Federation; 5 Bern University Hospital, Bern, Switzerland; 6 Department of Infectious Diseases, University of Bern, Bern, Switzerland; 7 Centre for Sexual Health and HIV Research, University College London, Mortimer Market Centre, London, United Kingdom; 8 Department of Immunocompromised, Hospital J.M. Ramos Mejia, Buenos Aires, Argentina; 9 Clinical Department, Republican Research and Practical Centre for Pulmonology and TB, Minsk, Belarus; 10 Infectious Diseases Service, Hospital Clinic – IDIBAPS, University of Barcelona, Barcelona, Spain; 11 Programa Integrado de Investigación en Tuberculosis de SEPAR (PII-TB), Centro de Investigación Biomédica en Red de Epidemiología y Salud Pública (CIBERESP), Agencia de Salud Pública de Barcelona, Barcelona, Spain; 12 Department of Infectious Diseases, INMI'L. Spallanzani’ Ospedale L Spallanzani, Rome, Italy; 13 INSERM, ISPED, Centre Inserm U 897- Epidemiologie-Biostatistique, Bordeaux, France

## Abstract

**Introduction:**

Management of TB in HIV patients in Eastern Europe (EE) is challenged by high MDR-TB prevalence, low rates of drug susceptibility testing (DST) and poor access to ART. We report 1-year mortality estimates from a multi-regional (EE, Western Europe (WE) and Latin America (LA)) cohort study.

**Methods:**

Deaths within 12 months of starting TB therapy (baseline) among consecutive HIV patients with TB in 2011–2013 were classified as being TB-related or not. Risk factors for all-cause and TB-related death were assessed using standard survival analysis methods.

**Results:**

Among 1410 patients starting TB therapy (EE=835, WE=319, LA=256), 257 (18%) died within 12 months of baseline; 170 (66%) of these were TB-related. The cumulative probability of all-cause and TB-related death at 12 months was 29, 5 and 11% (p<0.001) and 22, 1 and 4% (p<0.001) in EE, WE and LA respectively. In EE, fewer patients were on cART at 12 months (68% vs. 90% and 85%, p<0.001), and many were treated without access to baseline DST (66% vs. 37% and 69%, p<0.001). Among those with DST, the empiric treatment regimen (composed when DST results were not yet known) included <3 active TB-drugs in 36, 7 and 9% (EE, WE, LA, p<0.001); of those 81, 46 and 86% had MDR-TB. Patients who started <3 active drugs were at excess risk of dying from TB compared to patients starting ≥3 active drugs (aHR=3.20, 95% CI=1.82–5.66). Patients without DST results (and thus no option for targeting subsequent therapy) also had a greater risk of death (aHR=2.33, 1.40–3.87). This appeared driven by deaths in EE (Figure, aHR=2.37, 1.66–3.40, analyses restricted to EE), although a formal test for interaction with region was not significant (p=0.44), potentially due to few deaths outside EE.

**Conclusions:**

There is an elevated risk of death from TB in HIV patients managed in EE compared to WE and LA. This is partly explained by modifiable risk factors including low rates of DST, hampering the optimized choice of TB drugs in a setting of high MDR-TB prevalence. Our data call for urgent action to improve the care of HIV/TB patients in EE.

**Abstract MOAB0203–Figure 1:** TB-related death among HIV-positive patients according to the number of active drugs used as part of empiric TB therapy.

# Sub article: Missed opportunities in the TB/HIV cascade of care in 14 high burden TB/HIV African countries, 2012

**DOI:** 10.7448/IAS.18.5.20337

**Published:** 2015-07-22

**Authors:** Annabel Baddeley, Meg Doherty, Avinash Kanchar, Alberto Matteelli, Hazim Bakir Timimi, Haileyesus Getahun

**Affiliations:** 1 Global TB Programme, World Health Organization, Geneva, Switzerland; 2 Department of HIV/AIDS, World Health Organization, Geneva, Switzerland

## Abstract

**Introduction:**

Despite being preventable and curable, tuberculosis (TB) remains the leading cause of morbidity and mortality of people living with HIV (PLHIV). The past decade has seen considerable scale-up of collaborative TB/HIV activities, however, implementation remains suboptimal. Closer inspection at each stage of the cascade of TB/HIV care is warranted to assess the gaps and to identify opportunities for strengthened service delivery in order to eliminate HIV-associated TB mortality.

**Methods:**

Data were downloaded from the Global TB Programme Database on 22/01/2015 on the latest available TB treatment outcomes (2012 cohort), disaggregated by HIV status, from reporting high TB/HIV burden countries in the WHO African Region, along with related data on the implementation of collaborative TB/HIV activities. Data were analysed and missed opportunities identified.

**Results:**

Fourteen countries reported the required outcome data, accounting for some 570,000 HIV-positive incident TB cases, (Table 1), or 63% of the African burden and 49% of the global burden in 2012. More than 50,000 reported HIV-positive TB cases died or were lost to follow-up, representing 18% of evaluated cases, compared with 11% of evaluated HIV-negative TB cases, (Figure 1).

Of the estimated HIV-positive TB cases almost 260,000 (46%) went unreported, (Table 1). Among registered TB patients, 11% (around 80,000) did not have an HIV test in the TB register. In eight countries that reported, there was a gap of over 1,700,000 reported as not having received a TB screen, (53% of the 3,300,000 people in HIV care). Among notified HIV-positive TB cases, 42% (nearly 130,000) were not reported as receiving ART. Only five of the 14 countries reported providing Isoniazid Preventive Therapy (IPT) to people newly registered in HIV care. In the four countries that reported a denominator, 69% (some 900,000) people newly enrolled in HIV care did not receive IPT.

**Conclusions:**

This analysis highlights some considerable gaps in the care cascade, resulting from suboptimal implementation and/or recording and reporting. In order to prevent disproportionate TB mortality among PLHIV, countries are encouraged to scrutinize weaknesses in the care cascade at every level to enhance early detection of HIV-associated TB, timely ART initiation and scaled-up TB prevention.

**Abstract MOAB0204–Figure 1:** Comparison of TB treatment outcomes according to HIV status in 14 African Countries 2012 cohort.

**Abstract MOAB0204–Table 1:** Analysis of the cascade of TB/HIV care in 14 high TB/HIV burden African Countries, 2012 cohort

	Est. HIV-pos incident TB cases	% of est. HIV-pos TB cases unreported	Notified TB cases	% of TB cases with unreported HIV status	Notified TB cases with HIV-pos status	% of HIV-pos TB cases not on ART
14 high burden TB/HIV African countries	570,000	46	695,580	11	306,398	42

# Sub article: Empiric TB therapy does not decrease early mortality compared to isoniazid preventive therapy in adults with advanced HIV initiating ART: results of ACTG A5274 (REMEMBER study)

**DOI:** 10.7448/IAS.18.5.20481

**Published:** 2015-07-22

**Authors:** Mina Hosseinipour, Greg Bisson, Sachiko Miyahara, Xin Sun, Agnes Moses, Cynthia Riviere, Fredrick K Kirui, Sharlaa Badal-Faesen, David Lagat, Mulinda Nyirenda, Kogieleum Naidoo, James Hakim, Peter Mugyenyi, German Henostroza, Paul D Leger, Javier R Lama, Lerato Mohapi, Jorge Alave, Vidya Mave, Valdilea G Veloso, Sandy Pillay, Nagalingeswaran Kumarasamy, Jing Bao, Evelyn Hogg, Lynne Jones, Andrew Zolopa, Johnstone Kumwenda, Amita Gupta, Adult AIDS Clinical Trials Group 5274 Study Team

**Affiliations:** 1 UNC Project, Lilongwe, Malawi; 2 School of Medicine, University of North Carolina, Chapel Hill, United States; 3 University of Pennsylvania Perelman School of Medicine, Department of Biostatistics and Epidemiology, Philadelphia, United States; 4 Harvard School of Public Health, Department of Biostatistics, Harvard University, Boston, United States; 5 GHESKIO Centers, Port-au-Prince, Haiti; 6 Kenya Medical Research Institute (KEMRI), Kisumu, Kenya; 7 Clinical HIV Research Unit, Department of Medicine, University of Witwatersrand, Johannesburg, South Africa; 8 School of Medicine, Moi University, Eldoret, Kenya; 9 Johns Hopkins Project, Blantyre, Malawi; 10 Centre for the AIDS Programme of Research in South Africa, Durban, South Africa; 11 Department of Medicine, University of Zimbabwe, Harare, Zimbabwe; 12 Joint Clinical Research Centre, Kampala, Uganda; 13 Centre for Infectious Diseases Research, Lusaka, Zambia; 14 Asociacion Civil Impacta Saludy Educacion, Lima, Peru; 15 Perinatal HIV Research Unit (PHRU), Johannesburg, South Africa; 16 Johns Hopkins Clinical Trials Unit, B.J. Medical College, Pune, India; 17 Evandro Chagas National Institute of Infectious Diseases/Fiocruz, Rio de Janeiro, Brazil; 18 University of KwaZulu-Natal, Durban, South Africa; 19 YRGCARE Medical Centre, VHS, Chennai, India; 20 National Institutes of Health, Bethesda, United States; 21 Social & Scientific Systems, Silver Spring, United States; 22 Frontier Sciences, Buffalo, United States; 23 Stanford University, Palo Alto, United States; 24 School of Medicine, Johns Hopkins University, Baltimore, United States

## Abstract

**Introduction:**

Strategies for reducing the high early mortality seen among patients initiating antiretroviral therapy ART in resource-limited settings (RLS) are urgently needed. We hypothesized that given the high burden of tuberculosis (TB) in these settings, empiric TB treatment among patients at high risk for death would reduce early mortality.

**Methods:**

REMEMBER (*R*educing *E*arly *M*ortality and *E*arly *M*orbidity by *E*mpiric Tuberculosis Treatment *R*egimens) is a multicountry randomized clinical trial comparing two management strategies: ART+empiric 4 drug TB therapy (Empiric) vs. ART+isoniazid preventive therapy (IPT) in HIV-infected individuals with CD4 count <50 cells/mm^3^. Participants were screened for TB prior to entry using symptom screen, locally available diagnostics per standard of care, and GeneXpert when available. The study was stratified according to CD4 count (<25 vs. ≥25 cells/mm^3^) and poor prognostic factors (body mass index <18.5, haemoglobin <8 g/dl, recent hospitalization). The primary endpoint was survival (death or unknown status) at 24 weeks postrandomization, and Kaplan–Meier estimates of the endpoint rates across arms were compared by the *z*-test.

**Results:**

Of 1368 participants screened, 850 (62%) were randomized; 53% were male, 90% were black and median (quartiles) age was 36 (30–42) years. The median (quartiles) CD4 count at study entry was 18 cells/mm^3^ (9, 32). At week 24, both arms had the same primary endpoint rate of 5.2% (95% CI: 3.5–7.8% for Empiric and 3.4–7.8% for IPT) with an absolute risk difference of −0.06% (95% CI: −3.05 to 2.94%). Primary endpoint rates were similar across arms for the stratification factors and for other secondary outcomes: viral load <400 copies/mL was achieved in 84% Empiric and 85% IPT; Grade 3 or 4 symptoms occurred in 12% Empiric and 11% IPT; Grade 3 or 4 laboratory abnormalities in 23% both arms; and new clinical events in 49% Empiric and 51% IPT.

**Conclusions:**

Among highly TB screened participants with advanced HIV in RLS, empiric TB therapy did not reduce mortality at 24 weeks compared to IPT. The low mortality seen in both arms supports enhanced screening for TB prior to ART initiation and the routine use of IPT.

**Abstract MOAB0205LB–Figure 1:** KM graph for 5274

# Sub article: Final results of the HPTN 052 randomized controlled trial: antiretroviral therapy prevents HIV transmission

**DOI:** 10.7448/IAS.18.5.20482

**Published:** 2015-07-22

**Authors:** Myron Cohen, Ying Chen, Marybeth McCauley, Theresa Gamble, Mina Hosseinipour, Nagalingeshwaran Kumarasamy, James Hakim, Newton Kumwenda, Tania Brum, Beatriz Grinsztejn, Sheela Godbole, Suwat Chariyalertsak, Riegel Breno Santos, Kenneth Mayer, Irving Hoffman, Susan Eshleman, Estelle Piwowar-Manning, San-San Ou, Leslie Cottle, Joseph Makhema, Lisa Mills, Ravindre Panchia, Sharlaa Badal-Faesen, Joseph Eron, Joel Gallant, Diane Havlir, Susan Swindells, Vanessa Elharrar, David Burns, Taha Taha, Karin Nielsen, David Celentano, Max Essex, Thomas Fleming, HPTN 052 Study Group

**Affiliations:** 1 Division of Infectious Diseases, Department of Medicine, University of North Carolina at Chapel Hill, Chapel Hill, United States; 2 Statistical Center for HIV/AIDS Research and Prevention, Fred Hutchinson Cancer Research Center, Seattle, United States; 3 HPTN, HIV Prevention Trials Network, Washington, United States; 4 HPTN, HIV Prevention Trials Network, Washington, United States; 5 Institute for Global Health and Infectious Diseases, University of North Carolina at Chapel Hill, Chapel Hill, United States; 6 UNC Project-Malawi, Institute for Global Health and Infectious Diseases, Lilongwe, Malawi; 7 Y.R. Gaitonade Center for AIDS Research and Education, Chennai, India; 8 University of Zimbabwe, Harare, Zimbabwe; 9 Johns Hopkins Project, College of Medicine, Blantyre, Malawi; 10 Laboratorio de AIDS e Imunologia Molecular-IOC/Fiocruz, Hospital Geral de Nova Iguacu, Rio de Janeiro, Brazil; 11 Instituto Nacional de Infectologia Evandro Chagas-INI-Fiocruz, Rio de Janeiro, Brazil; 12 National AIDS Research Institute (ICMR), Pune, India; 13 Research Institute for Health Sciences, Chiang Mai University, Chiang Mai, Thailand; 14 Hospital Nossa Senhora da Conceição, Porto Alegre, Brazil; 15 The Fenway Institute, Boston, United States; 16 Beth Israel Deaconess Medical Center, Harvard Medical School, Boston, United States; 17 Institute of Global Health and Infectious Diseases, University of North Carolina, Chapel Hill, United States; 18 Department of Pathology, School of Medicine, Johns Hopkins University, Baltimore, United States; 19 Fred Hutchinson Cancer Research Center, Seattle, United States; 20 Botswana Harvard Aids Institute, Gaborone, Botswana; 21 KEMRI-Centers for Disease Control and Prevention, Kisumu, Kenya; 22 Chris Hani Baragwanath Hospital, Soweto HPTN CRS, Soweto, South Africa; 23 University of the Witwatersrand, Clinical HIV Research Unit, Johannesburg, South Africa; 24 Southwest CARE Center, Albuquerque, United States; 25 University of California, San Francisco, United States; 26 University of Nebraska Medical Center, Omaha, United States; 27 Clinical Prevention Research Branch/Prevention Sciences Program, Division of AIDS, National Institute of Allergy and Infectious Diseases, National Institutes of Health, Bethesda, United States; 28 Bloomberg School of Public Health, Johns Hopkins University, Baltimore, United States; 29 David Geffen UCLA School of Medicine, Los Angeles, United States; 30 Department of Epidemiology, Bloomberg School of Public Health, Johns Hopkins University, Baltimore, United States; 31 Harvard T.H. Chan School of Public Health AIDS Initiative, Boston, United States; 32 Botswana Harvard AIDS Institute Partnership, Gaborone, Botswana; 33 Department of Biostatistics, University of Washington, Seattle, United States

## Abstract

**Introduction:**

The HPTN 052 trial was designed to evaluate whether antiretroviral therapy reduces sexual transmission of HIV. The trial started in April 2005 and ended in May 2015.

**Methods:**

HPTN 052 enrolled 1763 HIV serodiscordant couples in Malawi, Zimbabwe, South Africa, Botswana, Kenya, Thailand, India, Brazil and the U.S. (97% heterosexual). HIV-infected index participants had CD4 cell counts between 350 and 550 cells/mm^3^ at enrolment. Index participants were randomized to receive ART at enrolment (early arm) or when their CD4 cell count fell to ≤250 cells/mm^3^ or they developed an AIDS-defining illness (delayed arm). The primary analysis was based on genetically linked viral transmission events. When interim analysis in May 2011 demonstrated the benefits of early ART, ART was offered to all index participants in the delayed arm (*N Engl J Med 2011*;365:493–505); the study then continued otherwise unchanged.

**Results:**

At the end of the trial, 1171 (66%) of 1763 couples remained in follow-up (603/886 early arm; 568/877 delayed arm). Index participants were followed for 9822 person-years (py). ART was initiated by all 886 index participants in the early arm and 785 (90%) of 877 index participants in the delayed arm. Before ART was offered to all index participants, there was 1 linked infection in the early ART arm (4 total infections/1776 py) and 35 linked infections in the delayed arm (42 total infections/1757 py). After ART was offered to index participants in both study arms, there were two linked infections in the early arm (15 total infections/2537 py) and six linked infections in the delayed arm (17 total infections/2412 py). Only seven linked infections were diagnosed while the index participant was receiving ART: four infections were diagnosed shortly after the index participant started ART and three were diagnosed after ART failure. These findings demonstrate that HIV transmission is very unlikely when viral replication is suppressed.

**Conclusions:**

The previously reported efficacy of early ART for HIV prevention was sustained for the duration of the HPTN 052 study. ART, combined with counselling and provision of condoms provides durable, highly effective protection from HIV transmission in serodiscordant couples.

# Sub article: Level of viral suppression and cascade of HIV care in a South African semi-urban setting in 2012

**DOI:** 10.7448/IAS.18.5.20338

**Published:** 2015-07-22

**Authors:** Kevin Jean, Adrian Puren, Ewaldé Cutler, Beverley Singh, Julie Bouscaillou, Reathe Rain-Taljaard, Dirk Taljjard, Pascale Lissouba, Gilles Peytavin, Bertran Auvert

**Affiliations:** 1 INSERM, Villejuif, France; 2 NICD-NHLS, Johannesburg, South Africa; 3 Progressus, Johannesburg, South Africa; 4 CHAPS, Johannesburg, South Africa; 5 APHP Bichat-Cl Bernard, Paris, France; 6 Ouest medical school of the University of Versailles, University of Versailles, Versailles – Saint Quentin en Yvelines, Versailles, France; 7 APHP – Hopital Ambroise Pare, Boulogne, France

## Abstract

**Introduction:**

For antiretroviral treatment (ART) programs to have a preventive impact, the proportion of HIV-infected people being treated should be high. In 2012, seven years after the beginning of ART programs in the South-African township of Orange Farm, we measured the proportion of HIV+ who were virally suppressed, especially among age groups highly exposed to HIV (women 18–29 years and men 25–34 years).

**Methods:**

A community-based cross-sectional representative survey conducted in 2012 among 3293 men and 3473 women. Study procedures included a face-to-face questionnaire and collection of blood samples that were tested for HIV, 10 antiretroviral drugs (ARVs) and HIV-viral load (VL).

**Results:**

HIV prevalence was 17.0% (95% Confidence Interval: 15.7–18.3%) among men and 30.1% (28.5–31.6%) among women. Overall, 59.1% (57.4–60.8%) of men and 79.5% (78.2–80.9%) of women reported having ever been tested for HIV. When controlling for age, circumcised men were more likely to ever have been tested (66.1% vs. 53.6%; p<0.001). Among HIV+ individuals, 21.0% (17.7–24.6%) of men and 30.5% (27.7–33.3%) of women tested positive for any ARV. The ratio of ARV+ people over those HIV− was 0.084. Using basic calculations, we found that if ART programs were actually treating all eligible patients since 2005, this ratio should have been 0.21–0.28, indicating an effectiveness of ART programs around 47–63%. Among ARV+ participants, 91.9% (88.7–94.3%) had viral suppression (VL<400 cp/mL). The proportion of viral suppression among HIV+ was 27.0% (24.3–29.9%) among women and 17.5% (14.4–20.9%) among men. These proportions were lower among the highly-exposed age groups: 15.6% (12.1–19.7%) among women and 8.4% (5.0–13.1%) among men.

**Conclusions:**

In Orange Farm, in the 2005–2012 period, ART programs were sub-optimal and, among HIV+, proportion of viral suppression was low, especially among the highly-exposed age groups. This suggests that, up to 2012, ART programs may not have substantially impacted HIV incidence. However, our study showed at community level that, when effectively taken, ARVs present a high effectiveness in suppressing VL.

# Sub article: A mathematical model to determine potential costs and benefits of increasing antiretroviral therapy coverage in female sex workers: the case of Panama

**DOI:** 10.7448/IAS.18.5.20339

**Published:** 2015-07-22

**Authors:** Lorna Jenkins, Jamie Nordio, Krisztina Vasarhelyi, Aurelio Nunez, Rolando Barrios, Alenxander Rutherford

**Affiliations:** 1 USF-Health and Education International Foundation, University of South Florida, Panama, Panama; 2 IRMACS, Simon Fraser University, Burnaby, Canada; 3 Faculty of Health Sciences, Simon Fraser University, Burnaby, Canada; 4 National STI/HIV/AIDS Program, Ministry of Health, Panama, Panama; 5 British Columbia Centre for Excellence in HIV/AIDS, Vancouver, Canada; 6 Department of Mathematics, Simon Fraser University, Burnaby, Canada

## Abstract

**Introduction:**

Panama adopted Treatment as Prevention (TasP) in February 2014 and is now seeking efficient and effective ways to expand antiretroviral therapy (ART) coverage in key populations. We developed a mathematical model to determine the ART coverage and associated costs required to meet HIV incidence reduction targets for the female sex worker (FSW) population, which has a 1.6% HIV prevalence.

**Methods:**

The Government of Panama, British Columbia Centre for Excellence in HIV/AIDS and Simon Fraser University are collaborating to develop mathematical models for informing Panama's TasP strategy. Quantitative and qualitative information was collected from national reports, key informant interviews and focus groups with civil society to inform a compartmental HIV transmission model incorporating disease progression and treatment. The model was calibrated and validated for 2013. Estimated FSW population size is 17,000 and according to the Global AIDS Response Progress report, current ART coverage for both FSW and the hard-to-reach client population is about 47%. Annual ART cost/individual is US$625. Simulation scenarios for meeting 50, 70 or 90% reduction in HIV incidence in FSW in 15 years assumed ART expansion either for FSW and their clients (Scenario 1) or for FSW only (Scenario 2).

**Results:**

ART expansion for FSW costs slightly more in Scenario 1 than 2. However, overall for both populations of FSW and clients, more infections are averted and treatment programme costs are lower for the strategy targeting FSW only (see Table 1). Furthermore, initial aggressive expansion of ART coverage leads to overall cost savings and a more effective means of averting new infections (see Figure 1). The result of no action compared to the 90% Scenario 2 strategy would be 170% more HIV infections and 50% more treatment costs over 15 years.

**Conclusions:**

Rapid expansion of TasP for female sex workers in Panama would avert infections and treatment costs already within 15 years. Initial short-term investment to increase ART coverage would be offset by long-term savings. Since Panama adopted TasP, UNAIDS has announced the 90–90–90 targets for HIV diagnosis, treatment and suppression, which call for an even more rapid reduction in incidence. Ongoing analyses are evaluating costs and outcomes of reaching the new targets by 2020.

**Abstract MOAC0103–Figure 1:** Treatment cost for FSW population.

**Abstract MOAC0103–Table 1:** Outcomes and costs of TasP expansion scenarios

Population	Target incidence reduction in FSW in 15 years (%)	No ART expansion new cases in 15 years	No ART expansion US$ costs in 15 years	TasP Scenario 1 new cases in 15 years	TasP Scenario 1 US$ costs in 15 years	TasP Scenario 2 new cases in 15 years	TasP Scenario 2 US$ costs in 15 years
FSW	50	2816	$1,240,560	2003	$911,016	2000	$1,025,737
Clients	50	4096	$3,841,072	2878	$3,308,139	2847	$3,139,332
Both	50	6912	$5,061,632	4881	$4,219,155	4847	$4,165,069
FSW	70	2816	$1,240,560	1620	$863,324	1605	$1,093,578
Clients	70	4096	$3,841,072	2331	$3,030,029	2259	$2,782,224
Both	70	6912	$5,061,632	3951	$3,893,353	3864	$3,875,802
FSW	90	2816	$1,240,560	1118	$777,438	1074	$1,107,593
Clients	90	4096	$3,841,072	1615	$2,818,353	1480	$2,260,854
Both	90	6912	$5,061,632	2733	$3,595,791	2554	$3,368,447

# Sub article: Does a universal test and treat strategy impact ART adherence in rural South Africa? ANRS 12249 TasP cluster-randomized trial

**DOI:** 10.7448/IAS.18.5.20340

**Published:** 2015-07-22

**Authors:** Collins Iwuji, Rosemary Dray-Spira, Alexandra Calmy, Joseph Larmarange, Joanna Orne-Gliemann, François Dabis, Deenan Pillay, Kholoud Porter

**Affiliations:** 1 Africa Centre for Health and Population Studies, University of KwaZulu-Natal, Mtubatuba, South Africa; 2 Research Department of Infection and Population Health, University College London, London, United Kingdom; 3 INSERM-UPMC Univ Paris 06, UMR_S 1136, Paris, France; 4 Unité VIH/Sida, Hôpitaux Universitaires, Geneva, Switzerland; 5 CEPED (UMR 196 U. Paris Descartes INED IRD), Paris, France; 6 ISPED, Epidemiologie-Biostatistique, Centre INSERM U 897, University of Bordeaux, Bordeaux, France; 7 Infection and Immunity, University College London, London, United Kingdom; 8 MRC Clinical Trials Unit at UCL, London, United Kingdom

## Abstract

**Introduction:**

HIV treatment guidelines are recommending ART at increasingly higher CD4 counts for maximizing individual and population benefits. However, the expansion of ART use may be at the expense of optimal adherence. We report on adherence and virological suppression when initiating ART at different CD4 thresholds within the Treatment as Prevention (ANRS 12249) trial of universal home-based testing and immediate ART initiation in rural KwaZulu-Natal.

**Methods:**

Using data of a cluster-randomized trial of immediate ART versus initiation according to current national guidelines (CD4≤350 cells/mm^3^), we compared adherence levels (≥95% vs. <95%) measured using a visual analogue scale (VAS) and pill count (PC) and virological suppression at six months (<400 c/mL) according to CD4 count at ART initiation through logistic regression models, adjusting for possible confounders (age, sex, marital status, education and employment).

**Results:**

During March 2012–May 2014, 601 participants who were not on ART entered care in trial clinics; 382 initiated ART; 254 have completed ≥6 months on ART, 227 of whom had six months HIV RNA data and were included in analyses. One hundred sixty-nine were women; median (IQR) age and CD4 at ART initiation were 35 years (28, 46) and 313 cells/mm^3^ (206, 513). Adherence ≥95% at six months was high (88 and 83% by PC and VAS, respectively) with no evidence that this was associated with CD4 at initiation (aOR=0.97 per 100 cells/mm^3^ higher, 95% CI: 0.83–1.12, p=0.65 for VAS; aOR 1.13 per 100 cells/mm^3^ higher, 0.98–1.31, p=0.09 for PC). Male sex was independently associated with <95% adherence (2.58, 1.24–5.35, p=0.01; ref. females). Eighty-three percent (183/227) of those who started ART achieved HIV suppression by six months with no association with CD4 at initiation (1.13 per 100 cells/mm^3^ higher, 0.96–1.33, p=0.40). Compared to those with ≥95% adherence by VAS, individuals with <95% adherence were somewhat less likely to suppress (0.44, 0.19–1.03, p=0.06).

**Conclusions:**

We found no evidence that, among people newly entering HIV care, higher CD4 at ART initiation was associated with reduced adherence or poorer virological suppression, at least in the short-term. In this rural South African setting, motivation to adhere to ART may be independent of the presence of symptomatic HIV disease.

# Sub article: Community-based HIV testing and linkage effectively delivers combination HIV prevention: results from a multisite randomized trial

**DOI:** 10.7448/IAS.18.5.20483

**Published:** 2015-07-22

**Authors:** Ruanne Barnabas, Heidi van Rooyen, Elioda Tumwesigye, Justin Brantley, Jared Baeten, Alastair van Heerden, Bosco Turyamureeba, Philip Joseph, Meighan Krows, James Hughes, Connie Celum, Linkages

**Affiliations:** 1 Department of Global Health, University of Washington, Seattle, United States; 2 Human Sciences Research Council, KwaZulu-Natal, South Africa; 3 Integrated Community-Based Initiatives, Kabwohe, Uganda; 4 Vaccine and Infectious Diseases Division, Fred Hutchinson Cancer Research Center, Seattle, United States

## Abstract

**Introduction:**

To have a population impact in generalized HIV epidemics in Africa, high coverage of combination HIV prevention strategies that reduce the susceptibility of uninfected persons and the infectiousness of infected persons is needed. Community-based HIV testing and counselling, with linkage to care and prevention, is a potential delivery platform for combination HIV prevention.

**Methods:**

We conducted a multisite programme of community-based HIV testing and counselling, linkage to HIV care, and demand creation for voluntary medical male circumcision (VMMC) in rural communities in KwaZulu-Natal, South Africa and Sheema district, Uganda. HIV testing was done at home or through mobile units. HIV-positive persons were randomly allocated to linkage to care strategies: clinic facilitation by lay-counsellors at the initial clinic visit, lay-counsellor follow-up visits at home, or standard clinic referral. HIV-negative uncircumcised men were randomized to VMMC demand creation strategies: lay counsellor follow-up visits at home, SMS reminders, or standard VMMC promotion at the time of testing.

**Results:**

Between June 2013 and February 2015, 15,332 persons received HIV testing and counselling. Among 1325 HIV-positive persons randomized to linkage strategies, the overall clinic linkage was high (93%). Compared to standard linkage, lay counsellor clinic facilitation increased linkage to care (RR=1.09, 95% CI: 1.05–1.13), and home follow-up visits increased antiretroviral therapy (ART) initiation (RR=1.23, 95% CI: 1.02–1.47). In all arms, ART initiation was limited by bottlenecks in service delivery at the clinics, although 67% of those eligible initiated ART by nine months. Overall, 82% of persons initiating ART achieved viral suppression without significant difference between study arms. Of 750 HIV-negative uncircumcised men randomized to VMMC promotion strategies, the uptake of circumcision was 41% by month 3. Compared to standard messages, VMMC uptake was significantly higher in the SMS promotion (RR=1.72, 95% CI: 1.36–2.17) and lay counsellor follow-up arms (and RR=1.67, 95% CI: 1.29–2.14).

**Conclusions:**

Community-based HIV testing and linkage to care and prevention effectively deliver combination HIV prevention. Simple strategies, such as SMS reminders or lay-counsellor visits, increase linkage for ART initiation and male circumcision. Community-based strategies require integration with efficient clinical services, and additional strategies are needed to address clinic delays that are barriers to ART delivery.

# Sub article: Treatment as prevention: characterization of partner infections in the HIV Prevention Trials Network 052 trial

**DOI:** 10.7448/IAS.18.5.20484

**Published:** 2015-07-22

**Authors:** Susan H Eshleman, Sarah E Hudelson, San San Ou, Andrew D Redd, Ronald Swanstrom, Stephen F Porcella, Ying Q Chen, Estelle Piwowar-Manning, Marybeth McCauley, Theresa Gamble, Matthew Sievers, Craig A Martens, Daniel Bruno, Li-Hua Ping, Elena Dukhovlinova, Thomas C Quinn, Johnstone Kumwenda, Madalitso Maliwichi, Nehemiah Nhando, Victor Akelo, Sikhulile Moyo, Ravindre Panchia, Nagalingeshwaran Kumarasamy, Nuntisa Chotirosniramit, Marineide M Rocha, Flavio Bustorff, Beatriz Grinsztejn, Kenneth H Mayer, James P Hughes, Myron S Cohen, for the HPTN 052 Study Team

**Affiliations:** 1 Department of Pathology, School of Medicine, Johns Hopkins University, Baltimore, United States; 2 Vaccine and Infectious Disease Division, Fred Hutchinson Cancer Research Center, Seattle, United States; 3 Department of Medicine, School of Medicine, Johns Hopkins University, Baltimore, United States; 4 Laboratory of Immunoregulation, National Institute of Allergy and Infectious Diseases, National Institutes of Health, Baltimore, United States; 5 Department of Biochemistry and Biophysics, University of North Carolina at Chapel Hill, Chapel Hill, United States; 6 Genomics Unit, Research Technologies Section, Rocky Mountain Laboratories, Division of Intramural Research, National Institute of Allergy and Infectious Diseases, National Institutes of Health, Hamilton, United States; 7 FHI 360, Washington, United States; 8 FHI 360, Durham, United States; 9 Lineberger Comprehensive Cancer Center, University of North Carolina at Chapel Hill, Chapel Hill, United States; 10 Johns Hopkins Project, College of Medicine, Blantyre, Malawi; 11 UNC Project, Lilongwe, Malawi; 12 University of Zimbabwe, Harare, Zimbabwe; 13 KEMRI-CDC, Kisumu, Kenya; 14 Botswana Harvard AIDS Institute, Gaborone, Botswana; 15 Soweto HPTN CRS, Chris Hani Baragwanath Hospital, Soweto, South Africa; 16 Y. R. Gaitonade Center for AIDS Research and Education, Chennai, India; 17 Research Institute for Health Sciences, Chiang Mai University, Chiang Mai, Thailand; 18 Hospital Nossa Senhora da Conceição, Porto Alegre RS, Brazil; 19 Laboratorio de AIDS e Imunologia Molecular-IOC/Fiocruz, Hospital Geral de Nova Iguacu, Rio de Janeiro, Brazil; 20 Instituto Nacional de Infectologia Evandro Chagas-INI-Fiocruz, Fiocruz, Rio de Janeiro, Brazil; 21 The Fenway Institute, Boston, United States; 22 Harvard Medical School, Beth Israel Deaconess Medical Center, Boston, United States; 23 Department of Medicine, University of North Carolina at Chapel Hill, Chapel Hill, United States

## Abstract

**Introduction:**

In 2011, results from an interim analysis of the HPTN 052 trial demonstrated that early antiretroviral therapy (ART) was highly effective for the prevention of HIV transmission from HIV-infected adults (index participants) to their HIV-uninfected sexual partners. All index participants were offered ART after May 2011; the trial ended in May 2015. This report describes the analysis of partner infections in HPTN 052.

**Methods:**

HIV from index-partner pairs was analyzed. Phylogenetic methods were used to compare HIV *pol* sequences from index-partner pairs and controls. Linkage probability was further assessed by comparing the genetic distances between *pol* sequences (Bayesian analysis). Selected samples were also analyzed using next generation sequencing (*envy* region). Three infections that occurred close to the time of index ART initiation were analyzed by BEAST and serologic methods to determine the probable timing of HIV transmission. This abstract presents provisional findings based on data available as of May 2015.

**Results:**

Seventy-five partner infections were confirmed (64 in Africa, 6 in Asia, 5 in the Americas), including 39 described previously (JID 2011; 204:1918–1926). Linkage status was determined for 70 cases (five cases failed analysis). Of these 70 cases, 26 (37%) were classified as unlinked (the partner was most likely infected from someone other than the index participant), and 44 (63%) were classified as linked (the index was most likely the source of the partner′s HIV infection). In 7 of the 44 linked cases, the partner seroconverted while the index was receiving study ART. In four of these seven cases, the partner seroconverted shortly after the index started ART, likely before the index was virally suppressed. In the remaining three cases, the partner seroconverted when the index was not virally suppressed due to ART failure.

**Conclusions:**

Laboratory and statistical methods were used to identify and characterize linked partner infections in HPTN 052. Seven linked infections were observed in partners after index participants started study ART: four occurred shortly after ART initiation and three occurred in the setting of ART failure. The timing of the linked transmission events supports the model that HIV transmission is very unlikely in the setting of viral suppression.

# Sub article: Post prevention of mother-to-child-transmission: 30-months outcomes in the Malawian “Option B+ programme”

**DOI:** 10.7448/IAS.18.5.20341

**Published:** 2015-07-22

**Authors:** Andreas D Haas, Lyson Tenthani, Malango T Msukwa, Andreas Jahn, Dalitso Midiani, Eustice Mhango, Oliver Gadabu, Kali Tal, Adrian Spörri, Matthias Egger, Frank Chimbwandira, Joep J van Oosterhout, Olivia Keiser

**Affiliations:** 1 Institute of Social and Preventive Medicine, University of Bern, Bern, Switzerland; 2 International Training and Education Centre for Health, University of Washington, Seattle, United States; 3 Department of HIV and AIDS, Ministry of Health, Lilongwe, Malawi; 4 Baobab Health Trust, Lilongwe, Malawi; 5 Dignitas International, Zomba, Malawi

## Abstract

**Introduction:**

Under the Option B+ PMTCT strategy, HIV-infected pregnant and breastfeeding women initiate lifelong ART. Long-term retention after weaning is unknown. We examine treatment outcomes for up to 30-months after ART initiation.

**Methods:**

We examined cumulative incidence of mortality, no follow-up after ART initiation, loss to follow-up after the first follow-up visit (LTF), treatment discontinuation and retention in the Malawian “Option B+ programme.” We analyzed 24-months aggregated facility-level data (65,749 patients, 654 facilities) and 30-months individual-level data (3225 patients; six large facilities) from Option B+ patients who initiated ART during 2011–2014. We excluded patients who transferred to another facility.

**Results:**

In *facility-level data*, 79.9% (52,525/65,749) and 75.0% (40,509/54,029) of all patients were still in care 6 and 12 months after ART initiation. After 24 months, 70.6% (17,257/24,245) were retained, 26.8% were LTF, 1.5% had died and 0.6% stopped ART. In six large facilities with *individual-level data*, slightly more patients defaulted or discontinued treatment: 24 and 30 months after ART initiation retention was 67.2 and 62.6%. Most patients were lost early and many did not return after the first visit (Figure 1), but after 18 months, further LTF was low. Of those who started ART during *pregnancy*, 15.8% (95% confidence interval (CI): 14.4–17.4%) had no follow-up, 18.0% (95% CI: 16.0–20.0%) were LTF, 6.6% (95% CI: 5.1–8.3%) stopped ART and 0.5% (95% CI: 0.3–1.0%) died during 30 months of follow-up. Of those who initiated ART while *breastfeeding*, 8.5% (95% CI: 6.8–10.4%) had no follow-up, 18.6% (95% CI: 15.7–21.7%) were LTF, 1.9% (95% CI: 1.0–3.5%) stopped ART and 0.6% died (95% CI: 0.2–1.3%) (Fig. 1). Patients who collected <85% of the prescribed drugs during the first year of ART were at higher risk of LTF between 13 and 30 months compared to patients who collected >95% of the prescribed drugs (aHR: 3.02; 95% CI: 1.99–4.59).

**Conclusions:**

Suboptimal long-term retention in care (67–70% after two years) needs to be addressed. Attrition rates are higher in those starting ART during pregnancy versus breast-feeding. Poor early drug adherence predicts later LTF. If women stay in care throughout breast-feeding, retention after weaning is likely.

**Abstract MOAC0201–Figure 1:** ART outcomes for Option B+ patients.

# Sub article: Recruiting male partners for couple HIV counselling and testing in Malawi's Option B+ programme: a randomized controlled trial

**DOI:** 10.7448/IAS.18.5.20342

**Published:** 2015-07-22

**Authors:** Nora Rosenberg, Tiwonge Mtande, Friday Saidi, Christopher Stanley, Edward Jere, Lusubiro Mwangomba, Kondwani Kumwenda, Innocent Mofolo, Mwawi Mwale, Annie Chauma, William C Miller, Irving Hoffman, Mina C Hosseinipour

**Affiliations:** 1 UNC Project, Lilongwe, Malawi; 2 Department of Medicine, University of North Carolina, Chapel Hill, United States; 3 District Health Office, Lilongwe, Malawi

## Abstract

**Introduction:**

In Malawi's antenatal programme, HIV counselling and testing (HCT) for pregnant women is nearly universal, but couple HCT (cHCT) is uncommon, even though it is included in the Option B+ guidelines. cHCT is critical for HIV-infected women: many have HIV-infected partners in need of HIV diagnosis and treatment or HIV-uninfected partners in need of HIV prevention. cHCT may also increase Option B+ retention. Two partner recruitment strategies were assessed for cHCT uptake, male HIV status, female Option B+ retention and consistent condom use.

**Methods:**

Newly diagnosed HIV-infected pregnant women≥16 years with male partners in Lilongwe were recruited from Bwaila District Hospital Antenatal Unit from March to October 2014 to participate in a randomized controlled trial. Women in the “invitation only” arm received an invitation inviting male partners to antenatal care; women in the “invitation plus tracing” arm received the same invitation but male partners were traced by phone and/or home visit if they failed to present within one week. Women were assessed one month later. Analyses were conducted using Chi-squared tests.

**Results:**

Of 220 eligible women, 200 (90%) consented and enrolled. cHCT uptake was 52% in the invitation only arm and 74% in the invitation plus tracing arm (p=0.001). Among the 126 men who presented for cHCT, 25% already knew they were HIV-infected, 47% learned they were HIV-infected for the first time and 25% were HIV-uninfected with no difference by arm (p=0.8). There was a trend towards greater one-month retention among women in the invitation plus tracing arm (91%) compared to the invitation only arm (83%) (p=0.09). Among HIV-discordant couples, unprotected sex declined from 94 to 23% (p<0.001) following cHCT. Participation did not lead to intimate partner violence in either arm.

**Conclusions:**

The invitation plus tracing strategy was extremely effective for recruiting male partners for cHCT and substantially more effective than the invitation only strategy. Both strategies identified many HIV-infected men and HIV-discordant couples. cHCT resulted in higher ART retention, declines in unprotected sex in HIV-discordant couples and no intimate partner violence. Scaling up an invitation plus tracing strategy within the Option B+ programme would have substantial public health benefits.

# Sub article: Zimbabwe approaching virtual elimination of mother to child transmission of HIV following implementation of Option A

**DOI:** 10.7448/IAS.18.5.20343

**Published:** 2015-07-22

**Authors:** Raluca Buzdugan, Sandra I McCoy, Constancia Watadzaushe, Mi-Suk Kang Dufour, Maya Petersen, Jeffrey Dirawo, Angela Mushavi, Hilda Angela Mujuru, Agnes Mahomva, Barbara Engelsmann, Anna Hakobyan, Owen Mugurungi, Frances M Cowan, Nancy S Padian

**Affiliations:** 1 School of Public Health, University of California, Berkeley, United States; 2 Centre for Sexual Health and HIV Research Zimbabwe, Harare, Zimbabwe; 3 Center for AIDS Prevention Studies, University of California, San Francisco, United States; 4 Ministry of Health and Child Care, Harare, Zimbabwe; 5 Department of Paediatrics and Child Health, University of Zimbabwe, Harare, Zimbabwe; 6 Elizabeth Glaser Pediatric AIDS Foundation, Harare, Zimbabwe; 7 Organization for Public Health Interventions and Development, Harare, Zimbabwe; 8 Children's Investment Fund Foundation, London, United Kingdom; 9 Institute of Epidemiology & Health, University College London, London, United Kingdom

## Abstract

**Introduction:**

We evaluated the impact of Option A, rolled out in August–December 2011, on HIV-free infant survival and mother-to-child transmission (MTCT) in Zimbabwe.

**Methods:**

In 2012 and 2014, we conducted cross-sectional community-based serosurveys of mother-infants pairs residing in the catchment areas of 157 health facilities randomly selected from 5 of 10 provinces in Zimbabwe. Eligible infants (alive or deceased) were born 9–18 months before each survey to mothers ≥16 years old. We randomly selected mother-infant pairs and conducted questionnaires and verbal autopsies and collected blood samples. The impact analysis was limited to 113 catchment areas *unexposed* to Option A activities at baseline according to facility records; we estimated the HIV-free infant survival and MTCT rate within each catchment area and compared the 2012 and 2014 estimates using a paired t-test.

**Results:**

We enrolled 8568 mother-infant pairs with viable maternal specimens in 2012 and 9619 in 2014, of whom 1107 (12.9%) and 1176 (12.2%) mothers respectively were HIV-infected. Among infants born to HIV-infected mothers, 90.6% (95% confidence interval (CI): 88.8, 92.3) of infants were alive and HIV-uninfected at 9–18 months in 2012, compared to 94.7% (95% CI: 93.4, 96.0) of infants in 2014 (p=0.001); MTCT was 9.0% (95% CI: 7.3, 10.7) in 2012 and 5.3% (95% CI: 4.0, 6.6) in 2014. In the 113 catchment areas where Option A was implemented *after* the infants surveyed in 2012 were born, there was a 6.5 percentage point (95% CI: 3.3, 9.7) mean increase in HIV-free infant survival (89.8 to 96.3%, p<0.001), and 6.2 percentage point (95% CI: 3.0, 9.4) mean decrease in MTCT (9.9 to 3.7%, p<0.001).

**Conclusions:**

We found a substantial and statistically significant increase in HIV-free infant survival and decrease in MTCT among infants aged 9–18 months following the implementation of Option A in Zimbabwe. Our estimates capture transmissions during pregnancy, delivery and the first 9–18 months of breastfeeding. Notably, 72% of HIV-exposed infants were still breastfeeding at baseline and 78% at endline, so additional infections may occur. The 2014 survey also provides a baseline for evaluating Option B+, which has been recently rolled out in Zimbabwe and should further accelerate efforts to eliminate MTCT.

# Sub article: Antiretroviral intensification to prevent intrapartum HIV transmission in late comers

**DOI:** 10.7448/IAS.18.5.20344

**Published:** 2015-07-22

**Authors:** Marc Lallemant, Billy Amzal, Saik Urien, Patumrat Sripan, Tim Cressey, Nicole Ngo-Giang-Huong, Boonsong Rawangban, Prapan Sabsanong, Thitiporn Siriwachirachai, Tapnarong Jarupanich, Prateep Kanjanavikai, Phaiboon Wanasiri, Suporn Koetsawang, Gonzague Jourdain, Sophie Le Cœur, PHPT-5 study team

**Affiliations:** 1 IRD174/PHPT, Chiang Mai, Thailand; 2 Department of Immunology and Infectious Diseases, Harvard Chan School of Public Health, Boston, United States; 3 Faculty of Associated Medical Sciences, Chiang Mai University, Chiang Mai, Thailand; 4 LASER Analytica, London, United Kingdom; 5 EA 08, Sorbonne Paris Cité, Université Paris Descartes, Paris, France; 6 0901 INSERM, URC Paris Centre Necker Cochin, Paris, France; 7 Department of Statistics, Faculty of Science, Kasetsart University, Bangkok, Thailand; 8 Nopparat Rajathanee Hospital, Bangkok, Thailand; 9 Samutprakarn Hospital, Samutprakarn, Thailand; 10 Khon Kaen Hospital, Khon Kaen, Thailand; 11 Banglamung Hospital, Chonburi, Thailand; 12 Kalasin Hospital, Kalasin, Thailand; 13 Family Health Research Center, Mahidol University, Bangkok, Thailand; 14 Institut National d'Etudes Démographiques (INED), Paris, France

## Abstract

**Introduction:**

Infants born to HIV-infected pregnant women presenting late are at high risk of intrapartum infection. Mother/infant antiretroviral (ARV) intensification may substantially reduce this risk.

**Methods:**

In a multicentre, phase 3, adaptive single-arm trial in Thailand, pregnant women with <8 weeks of standard ARVs (zidovudine (ZDV)+lamivudine (3TC)+lopinavir/ritonavir) and their infants received “ARV intensification” to prevent transmission at delivery: women took a single nevirapine (NVP) dose in labour and continued ARVs for four weeks; formula-fed neonates received two weeks AZT+3TC+NVP followed by two weeks AZT+3TC, instead of standard one-week ZDV. Infants were tested for HIV at birth, one, two, four, six months. A negative DNA PCR <48 hours, followed by a confirmed positive PCR defined intrapartum transmission.

Data from 3965 mother/infant pairs (84 intrapartum transmissions) in three PHPT randomized perinatal HIV prevention trials (NCT00386230, NCT00398684 and NCT00409591) conducted in the same setting were used to define an historical control and build an intrapartum transmission model. Viral load (VL) during pregnancy was modelled as a function of ARVs exposure and intrapartum transmission was predicted through a logistic model with VL, maternal/infant ARVs, delivery mode and prematurity status as covariates. The Bayesian estimation of the risks of intrapartum transmission with/without intensification used all historical information and decision rules to stop for futility or superiority of ARV intensification over standard of care (risk ratio, RR<1) were determined for three interim analyses. Prior intrapartum transmission probabilities were subsequently updated using the results of the intensification trial to derive posterior probabilities (credibility interval, CrI) as well as probability distributions of RR<1 and RR<0.5.

**Results:**

At first interim analysis, the DSMB recommended stopping enrolment and reporting intensification efficacy. Overall 88 mother/infant pairs received intensification with no intrapartum transmission.

The posterior probability of intrapartum transmission was 0.4% (95% CrI: 0.1–1.4%) with intensification compared to 2.0% (0.3–5.2%) without. The probability of superiority of intensification over standard of care (RR<1) was 94.1%, and that of at least a two-fold reduction of risk (RR<0.5) was 82.9%. ARV intensification appeared safe.

**Conclusions:**

ARV intensification is very effective in preventing intrapartum transmission in pregnant women receiving a short course antepartum ARVs before delivery.

**Abstract MOAC0204–Table 1:** Women's baseline characteristics

Characteristics	Historical data	Intensification
N	3965	88
Age (IQR) – years	25.7 (22.5–29.7)	26.3 (22.3–33.0)
CD4 (IQR) – cells/mm^3^	380 (260–527)	368 (255–503)
VL baseline (IQR) – log10 copies/mL	4.0 (3.4–4.6)	4.3 (3.7–4.7)
VL delivery (IQR) – log10 copies/mL	3.2 (2.3–4.0)	2.2 (1.8–2.9)
GA delivery (IQR) – weeks	38.7 (37.9–39.7)	38.6 (38.0–39.3)
C/section (%)	(21%)	(36%)

**Abstract MOAC0204–Figure 1:** Intrapartum transmission posterior probabilities.

# Sub article: Costs of Zimbabwe's accelerated prevention of mother-to-child transmission of HIV programme

**DOI:** 10.7448/IAS.18.5.20485

**Published:** 2015-07-22

**Authors:** Ivan Ochoa-Moreno, Collin Mangenah, Raluca Buzdugan, Nancy S Padian, Sandra I Mccoy, Frances M Cowan, Sergio Bautista-Arredondo

**Affiliations:** 1 National Institute of Public Health, Cuernavaca, Mexico; 2 Centre for Sexual Health and HIV/AIDS Research, Harare, Zimbabwe; 3 University of California, Berkeley, United States; 4 University College London, London, United Kingdom

## Abstract

**Introduction:**

In 2010 and 2013, World Health Organization issued revised guidelines on the recommended approaches for prevention of mother-to-child transmission of HIV (PMTCT) (Options A, B, B+). Estimating the cost of these PMTCT regimens is essential. We estimated the cost of Option A in Zimbabwe, which was rolled out in 2011. These data also represent baseline estimates to assess the cost-effectiveness of Option B+, rolled out in Zimbabwe in late 2013.

**Methods:**

We conducted a cross-sectional survey of 157 randomly selected health facilities offering PMTCT services in 5 of 10 provinces in Zimbabwe. In each facility, we collected data on the output and cost of PMTCT services, including staff and supplies for the whole year and for each month of 2013. We also assessed the time allocation of staff providing these services. We estimated the average cost of PMTCT services per facility and for specific services in the PMTCT cascade such as HIV testing and antiretroviral prophylaxis. We also examined the variation in costs by the type of provider.

**Results:**

We estimated that the average cost of PMTCT services is approximately US$13,600 (median US$9074) per facility-year, which varies widely by facility size and type. On average, 80% of the overall cost corresponds to staff (US$10,900) and the remaining 20% to supplies (US$2700). The average cost per pregnant woman tested was US$75 (median US$44) and the average cost per HIV-infected pregnant woman on antiretroviral prophylaxis or treatment was US$1040 (median US$527) per year. Scale was associated with cost; 40% of the variation in the cost per pregnant woman tested can be explained by number of HIV+ women on ART/ARV, as was 50% of the variation in prophylaxis and treatment costs (see Figure).

**Conclusions:**

These findings are the first empirical estimations of PMTCT programmes costs in Zimbabwe. Given limited resources, calls for the elimination of MTCT have challenged the international community to optimize the use of resources to increase coverage of PMTCT priority services. Information about costs is essential to determine the highest possible quality HIV services at the lowest feasible cost and thus maximize efficiency.

**Abstract MOAC0205LB–Figure 1:** Facility-level variation of average cost per service in two stages of the PMTCT cascade vs. scale.

# Sub article: Increasing uptake of voluntary medical male circumcision among men aged 20–34 years in Njombe and Tabora regions, Tanzania: a cluster-randomized controlled trial

**DOI:** 10.7448/IAS.18.5.20486

**Published:** 2015-07-22

**Authors:** Mwita Wambura, Richard Hayes, Jonathan Grund, Saidi Kapiga, Hally Mahler, Gerry Mshana, Evodius Kuringe, Marya Plotkin, Naomi Bock, Natasha Larke, John Changalucha, Helen Weiss, VMMC Tanzania Study Group

**Affiliations:** 1 NIMR, Mwanza, Tanzania, United Republic of Tanzania; 2 LSHTM, London, United Kingdom; 3 CDC/CGH/DGHA, Atlanta, United States; 4 MITU/LSHTM, Mwanza, Tanzania, United Republic of Tanzania; 5 Jhpiego/Tanzania, Dar es Salaam, Tanzania, United Republic of Tanzania

## Abstract

**Introduction:**

Tanzania introduced voluntary medical male circumcision (VMMC) in 2009 as part of its national HIV prevention strategy. Reaching men aged 20–34 years with circumcision may affect the most immediate reduction in HIV incidence. However, approximately 80% of VMMC clients in Tabora and Njombe regions are aged 10–19 years. This study evaluated the effect of a strategy to increase VMMC uptake among men aged 20–34 years in Njombe and Tabora.

**Methods:**

A cluster-randomized controlled trial at 20 VMMC outreach sites was conducted in Njombe and Tabora, focusing on increasing VMMC uptake. The intervention, which was informed by formative research, included 1) additional demand-creation messages (non-HIV benefits of VMMC, voluntary nature of HIV testing), 2) involvement of recently circumcised men as auxiliary peer promoters, 3) separate waiting and education areas for men aged >20 years, and 4) sessions on wound healing and post-circumcision abstinence targeting female partners. Analysis was based on cluster-level summary measures.

**Results:**

Overall, 6251 men were enrolled in 10 intervention sites (1809 Njombe and 4442 Tabora) and 3968 men in the 10 control sites (1035 Njombe and 2933 Tabora). The proportion of clients aged 20–34 was greater in intervention sites compared to control sites (17.7% vs. 13.0%; RR=1.4; 95% CI: 0.9–2.0; p=0.11) and was associated with a greater number of clients in both regions (overall mean difference=227; 95% CI: 33–420; p=0.03). The effect of the intervention varied by region: in Njombe, there was little difference in attendance between control and intervention sites (11.3% vs. 14.7%; RR=0.77, 95% CI: 0.4–1.6; p=0.43), while in Tabora, there was over a twofold difference (27.5% vs. 11.5%; RR=2.39, 95% CI: 1.7–3.4; p=0.03). Similarly, the mean number of clients aged 20–34 was greater in intervention facilities in Tabora (mean difference=182; 95% CI: 5–359; p=0.05) and there was little difference in Njombe (mean difference=12; 95% CI: −13 to 36; p=0.31).

**Conclusions:**

The intervention was associated with a significant increase in the proportion of VMMC clients aged 20–34 years in Tabora but not in Njombe. The lack of intervention effect in Njombe may be due to saturation, as VMMC has been available for longer. The results suggest that the intervention may be more likely to be effective in areas newly targeted for VMMC.

# Sub article: Acceptability and feasibility of a novel approach to promote HIV testing in sexual and social networks using HIV self-tests

**DOI:** 10.7448/IAS.18.5.20487

**Published:** 2015-07-22

**Authors:** Harsha Thirumurthy, Immaculate Akello, Katherine Murray, Samuel Masters, Suzanne Maman, Eunice Omanga, Kawango Agot

**Affiliations:** 1 Department of Health Policy and Management, University of North Carolina at Chapel Hill, Chapel Hill, United States; 2 Carolina Population Center, University of North Carolina at Chapel Hill, Chapel Hill, United States; 3 Impact Research and Development Organization, Kisumu, Kenya; 4 Department of Health Behavior, University of North Carolina at Chapel Hill, Chapel Hill, United States

## Abstract

**Introduction:**

Identifying interventions to increase men's uptake of HIV testing in sub-Saharan Africa is essential for the success of combination prevention strategies, including treatment as prevention. HIV self-testing is an emerging approach with high acceptability, but limited evidence exists on optimal strategies for distributing self-tests and reaching men in particular. This study explored a novel approach of providing *multiple* self-tests to women with high HIV incidence to promote HIV testing among their sexual partners.

**Methods:**

HIV-uninfected women aged 18–39 years were recruited at two sites in Kisumu, Kenya between January and March 2015: a drop-in centre for female sex workers (FSWs) and a health facility with antenatal and postpartum clinics. Following informed consent and instructions on using the OraQuick Rapid HIV 1/2 Test, index participants (IPs) enrolled at the health facility and drop-in centre received three and five self-tests, respectively. Structured interviews were conducted with IPs at enrolment and multiple times over three months to determine how self-tests were used. Key outcomes included the proportion of IPs reporting their primary sexual partner used a self-test.

**Results:**

A total of 278 IPs were enrolled (101 FSWs, 61 antenatal, 116 postpartum). Follow-up interviews were completed with 262 IPs (94.2%) by May 9, 2015. Most self-tests provided at enrolment were either used by the IP or given to other persons (mean 2.7 (90%) for antenatal and postpartum IPs, 4.7 (94%) for FSWs). All but two IPs gave ≥1 self-tests to other persons, and a large majority gave a self-test to their primary sexual partner (77% FSWs, 91.8% antenatal and 86% postpartum). Ninety-eight percent of self-tests given to other persons were reported to be used. Among 367 persons who received self-tests from FSWs and used them, commercial sex clients were the largest group (211, 57%). In total, 10.6% (72/681) of those who received self-tests from IPs and used them were reported to obtain an HIV-positive result; 55% of them sought confirmatory testing.

**Conclusions:**

Provision of multiple HIV self-tests to sub-populations of women with high HIV incidence was successful in promoting HIV testing among their sexual partners. This novel strategy warrants further consideration as countries develop self-testing policies.

# Sub article: Community outbreak of HIV infection linked to injection drug use of oxymorphone – Indiana, 2015

**DOI:** 10.7448/IAS.18.5.20488

**Published:** 2015-07-22

**Authors:** Joan Duwve, Karen Hoover, Caitlin Conrad, Romeo Galang, Daniel Hillman, Brooke Hoots, Monita Patel, Philip Peters, Pam Pontones, Jeremy Roseberry, Jessica Shields, Dorothy Waterhouse, Paul Weidle

**Affiliations:** 1 Indiana State Department of Health, Indianapolis, United States; 2 Richard M. Fairbanks School of Public Health, Indiana University, Indianapolis, United States; 3 Centers for Disease Control and Prevention, Atlanta, United States; 4 Clark County Health Department, Jeffersonville, United States

## Abstract

**Introduction:**

On 23 January 2015, the Indiana State Department of Health began investigating an outbreak of HIV infection after disease intervention specialists (DIS) reported 11 confirmed HIV cases traced to a rural community in southeastern Indiana that had reported five HIV cases between 2004 and 2013. From 2009 to 2013, the community (population 4200) had substantial unemployment (8.9%), many adults without high school diplomas (21.3%), a substantial proportion living in poverty (19%) and a limited healthcare access. A public health emergency was declared on March 26 by executive order. We report on efforts to diagnose HIV infection in this community.

**Methods:**

For individuals newly diagnosed with HIV infection, partner services’ interviews elicited information about needle-sharing and sex partners and social contacts (who could benefit from an HIV test) within the past 12 months. HIV testing was offered to all contacts who could be located.

**Results:**

DIS identified 491 unique individuals during contact tracing, and as of May 13, 390/491 (79%) persons were located, assessed for risk and tested for HIV. Overall, 153/390 (39%) persons were diagnosed with HIV infection. There was no difference in age and sex between HIV-positive and HIV-negative tested persons (Table 1). Compared with HIV-negative contacts (n=239), the 153 HIV-infected individuals were more likely to be named as needle-sharing partners (81% vs. 52%; p<0.0001) and less likely to be named as sexual partners only (1% vs. 15%; p<0.0001) during contract tracing. All individuals reporting injection drug use described practices including crushing, dissolving and cooking OPANA^®^ ER or extended-release generic oxymorphone. The reported daily numbers of injections ranged from 4 to 15, and the number of injection partners ranged from 1 to 6 per injection event. Individuals reported that injection drug use in this community is a multigenerational activity with family and community members injecting together, frequently sharing syringes and drug preparation equipment.

**Conclusions:**

This outbreak highlights the vulnerability of rural, resource-poor populations to drug use, misuse and addiction; the importance of timely HIV surveillance activities and rapid response to interrupt disease transmission and the need for expanded mental health and substance use treatment programmes in medically underserved rural areas.

**Abstract MOAC0303LB–Table 1:** HIV cases and contacts identified during investigation

	HIV-positive N (%)	HIV-negative N (%)	Total N (%)
Overall	153	237	390
Male sex	88 (58)	132 (56)	220 (56)
Median age (range)	34 (18–57)	35 (13–75)	34 (13–75)
HIV risk factor			
Sexual risk only	2 (1)	36 (15)	38 (10)
Needle-sharing risk only	65 (42)	87 (37)	152 (39)
Sexual and needle-sharing risk	59 (39)	36 (15)	95 (24)
Unknown	27 (18)	78 (33)	105 (27)

# Sub article: HIV-1 and HCV molecular epidemiology of a large community outbreak of HIV-1 infection linked to injection drug use of oxymorphone – Indiana, 2015

**DOI:** 10.7448/IAS.18.5.20489

**Published:** 2015-07-22

**Authors:** Romeo R Galang, Jessica Gentry, Philip J Peters, Sara J Blosser, Erika L Chapman, Caitlin Conrad, Joan M Duwve, Lilia Ganova-Raeva, Walid Heneine, Daniel Hillman, Hongwei Jia, Lixia Liu, Wei Luo, Judy Lovchik, Silvina Masciotra, S Michele Owen, Andrea Perez, Paula Peyrani, Pam Pontones, Sumathi Ramachandran, Jeremy C. Roseberry, Michelle Sandoval, Anupama Shankar, Hong Thai, Guoliang Xia, Yury Khudyakov, William M Switzer

**Affiliations:** 1 Division of HIV/AIDS Prevention, National Center for HIV/AIDS, Viral Hepatitis, STD, and TB Prevention, CDC, Atlanta, United States; 2 Epidemic Intelligence Service, CDC, Atlanta, United States; 3 Indiana State Department of Health, Indianapolis, United States; 4 Richard M. Fairbanks School of Public Health, Indiana University Indianapolis, United States; 5 Division of Infectious Diseases, National Center for HIV/AIDS, Viral Hepatitis, STD, and TB Prevention, CDC, Atlanta, United States; 6 Division of Viral Hepatitis, National Center for HIV/AIDS, Viral Hepatitis, STD, and TB Prevention, CDC, Atlanta, United States; 7 University of Louisville, Louisville, United States

## Abstract

**Introduction:**

In January 2015, a cluster of HIV-1 infections was detected in a rural county in southeastern Indiana among persons who reported injection of the prescription opioid oxymorphone. As of 13 May 2015, HIV-1 infection has been diagnosed in 153 individuals. We compare molecular analyses of HIV-1 and HCV sequences among a subset of individuals in this outbreak to infer the timing of HIV transmission relative to HCV.

**Methods:**

Serum and plasma samples were collected from November 2014 to April 2015. HIV polymerase (*pol*) gene sequences from persons with newly diagnosed HIV infection were phylogenetically analyzed. Phylogenetic clusters were defined when HIV-1 *pol* sequences were highly genetically related (>97% nucleotide identity) and statistical evidence supporting relatedness was high (Shimodaira–Hasegawa probabilities >0.99). Recency of HIV infection was determined by avidity testing using a modified Bio-Rad HIV 1/2 plus O assay (BRAI). HCV NS5B gene sequences were phylogenetically analyzed to determine the number of clusters of independent HCV strains within this population.

**Results:**

The *pol* gene was sequenced for 57 HIV-1-infected persons. Two clusters of HIV-1 subtype B infection were identified (Cluster 1, n=55; Cluster 2, n=2; Figure, panel a). Among 49 specimens available for BRAI testing, 45 (91.8%) were recent infections. Of 36 HIV-infected specimens with HCV antibody results, 34 (94%) were HCV co-infected. The NS5B gene was sequenced for 119 HCV-infected persons. Genotype 1a (n=82) was most common, followed by genotype 3a (n=29), 2b (n=5) and 1b (n=3). Three unique clusters of HCV strains were identified (Cluster 1, n=45; Cluster 2, n=9; Cluster 3, n= 7; Figure, panel b). Of 118 HCV-infected specimens with HIV antibody results, 38 (32.2%) were HIV co-infected.

**Conclusions:**

In this prescription opioid injection-associated outbreak, a single strain of HIV-1 was introduced into a population infected with multiple HCV strains. In contrast to the homogeneity of HIV strains observed in this cohort, the heterogeneity of HCV strains (clustering and non-clustering) suggests earlier introduction of HCV compared with HIV. These data demonstrate the outbreak potential with the introduction of HIV-1 into a community where HCV prevalence is high among persons who inject prescription opioids.

**Abstract MOAC0304LB–Figure 1:** Maximum likelihood phylogenetic tree of (a) HIV-1 pol sequences and (b) HCV NS 5b sequences.

# Sub article: HPTN 067/ADAPT study: a comparison of daily and intermittent pre-exposure prophylaxis dosing for HIV prevention in men who have sex with men and transgender women in New York city

**DOI:** 10.7448/IAS.18.5.20538

**Published:** 2015-07-22

**Authors:** Sharon Mannheimer, Yael Hirsch-Moverman, Avelino Loquere, Julie Franks, James Hughes, San-San Ou, K Rivet Amico, Craig Hendrix, Bonnie J Dye, Estelle Piwowar-Manning, Mark Marzinke, Vanessa Elharrar, Michael Stirratt, Robert M Grant, HPTN 067/ADAPT Harlem Study Team

**Affiliations:** 1 Department of Medicine, Harlem Hospital/Columbia University, New York, United States; 2 Department of Epidemiology, Columbia University Mailman School of Public Health, New York, United States; 3 ICAP, Columbia University Mailman School of Public Health, New York, United States; 4 Department of Biostatistics, University of Washington, Seattle, United States; 5 Fred Hutchinson Cancer Research Center, Seattle, United States; 6 Department of Health Behavior and Health Education, University of Michigan, Ann Arbor, United States; 7 Department of Medicine, Johns Hopkins University, Baltimore, United States; 8 FHI 360, Durham, United States; 9 Department of Pathology, Johns Hopkins University, Baltimore, United States; 10 Clinical Prevention Research Branch, PSP/DAIDS/NIAID/NIH, Bethesda, United States; 11 Division of AIDS Research, National Institute of Mental Health, Bethesda, United States; 12 Gladstone Institutes, University of California, San Francisco, San Francisco, United States

## Abstract

**Introduction:**

Daily oral FTC/TDF (Truvada) is US FDA-approved for HIV pre-exposure prophylaxis (PrEP). HPTN 067/ADAPT, a phase II randomized, open-label PrEP trial, assessed the feasibility of intermittent FTC/TDF-based PrEP for HIV prevention among men who have sex with men (MSM) and transgender women (TGW) in New York City (NYC).

**Methods:**

MSM and TGW were eligible if: male at birth, and reported anal intercourse and ≥1 other HIV risk factor in the past six months. Exclusion criteria included HIV infection, hepatitis B infection, acute HIV symptoms and abnormal renal function. Following six weeks of once/week directly observed dosing, participants were randomly assigned 1:1:1 to 24 weeks of PrEP dosed: daily (D), twice weekly plus one post-sex dose (time-driven (T)), or one pre- and one post-sex dose (event-driven (E)). Regimens were compared for prophylactic coverage (PrEP within four days pre- and 24 hours post-sex) of sex events, pills taken, side effects and plasma drug levels. Adherence and coverage were assessed using electronic monitoring adjusted by self-reported sex and pill taking behaviour collected in detailed weekly interviews.

**Results:**

A total of 179 participants were randomized: 176 MSM, 3 TGW; median age 30 years; 70% black, 13% white and 25% Hispanic. D arm participants had significantly higher complete coverage of sex acts (66% D, 47% T, 52% E; p=0.03; Table 1) and highest adherence to regimen (65% D, 46% T, 41% E; p<0.001). Significantly fewer pills were used with intermittent (T and E) PrEP (p<0.001). Side effects were similar across arms, with gastrointestinal and neurologic symptoms most common. Participants reporting recent sex in all PrEP dosing arms achieved similar rates of detectable plasma tenofovir levels and of concentrations associated with effective PrEP dose frequency.

**Conclusions:**

While this cohort of mostly black MSM in NYC reported higher prophylactic coverage of sex acts and higher adherence to daily PrEP, non-daily PrEP users who reported recent sex achieved comparable rates of effective tenofovir plasma concentrations. Intermittent PrEP required substantially fewer pills, although side effects were similar. This study demonstrates the feasibility of intermittent PrEP, a potentially more cost-effective alternative to daily PrEP, among U.S. black MSM.

**Table:** Abstract MOAC0305LB–Table 1

Characteristic	Study regimen daily (D), n=59	Study regimen time (T), n=60	Study regimen event (E), n=60	p
Number of sex events during study, excluding oral sex	1083	1311	1502	0.20
% total sex events with complete coverage (or for sex events with partial coverage % pre-sex only, % post-sex only)	66 (24, 2)	47 (30, 8)	52 (29, 6)	0.03
Total number of required pills taken	5370	1708	1063	<0.001
Total % PrEP adherence	65	46	41	<0.001
% Participants with neurologic side effects (e.g. headache, dizzy and lightheaded)	24	20	18	0.64
% Participants with gastrointestinal side effects (e.g. nausea, vomiting, diarrhoea, bloating, gas)	39	18	28	0.51
% Participants with detectable tenofovir (TFV) (>0.31 ng/mL) in plasma when reporting sex in last 7 days at 10 weeks, at 30 weeks	74, 61	76, 56	64, 50	0.58
Median plasma TFV concentration (ng/mL) in plasma when reporting sex in last 7 days at 10 weeks, at 30 weeks	83, 31	24, 11	15, 1	0.49
% achieving effective plasma TFV concentration (>5 ng/mL) when reporting sex in last 7 days at 10 weeks, at 30 weeks	63, 56	72, 50	61, 39	0.65

# Sub article: HPTN 067/ADAPT study: a comparison of daily and non-daily pre-exposure prophylaxis dosing in Thai men who have sex with men, Bangkok, Thailand

**DOI:** 10.7448/IAS.18.5.20539

**Published:** 2015-07-22

**Authors:** Timothy H Holtz, Anupong Chitwarakorn, Marcel E Curlin, James Hughes, K Rivet Amico, Craig Hendrix, Bonnie J Dye, Peter L Anderson, San-San Ou, Vanessa Elharrar, Susan H Eshleman, Michael Stirratt, Robert M Grant, Bangkok HPTN 067/ADAPT Study Team

**Affiliations:** 1 HIV/STD Research Program, Thailand Ministry of Public Health – U.S. Centers for Disease Control and Prevention Collaboration, Bangkok, Thailand; 2 U.S. Division of HIV/AIDS Prevention, Centers for Disease Control and Prevention, Atlanta, United States; 3 Department of Disease Control, Ministry of Public Health, Nonthaburi, Thailand; 4 Department of Biostatistics, University of Washington, Seattle, United States; 5 Fred Hutchinson Cancer Research Center, Seattle, United States; 6 Department of Health Behavior and Health Education, University of Michigan, Ann Arbor, United States; 7 Department of Medicine, Johns Hopkins University, Baltimore, United States; 8 FHI 360, Durham, United States; 9 Department of Pharmaceutical Sciences, University of Colorado, Aurora, United States; 10 Clinical Prevention Research Branch, PSP/DAIDS/NIAID/NIH, Bethesda, United States; 11 Department of Pathology, Johns Hopkins University, Baltimore, United States; 12 Division of AIDS Research, National Institute of Mental Health, Bethesda, United States; 13 Gladstone Institutes, University of California, San Francisco, United States

## Abstract

**Introduction:**

Oral FTC/TDF PrEP is effective for preventing sexual HIV acquisition when used daily. An alternate dosing (non-daily) regimen was effective in the IPERGAY trial. Daily and non-daily regimens have not been compared directly with respect to prophylactic coverage for sexual exposure.

**Methods:**

We enrolled men who have sex with men (MSM) into a phase 2, randomized, open-label trial of oral FTC/TDF PrEP in Bangkok, Thailand. We randomly assigned participants to one of three self-administered dosing regimens for 24 weeks: daily (D); time-driven twice weekly with a post-sex dose (T) or event-driven before and after sex (E). We contacted participants weekly to collect dates/times of PrEP use (monitored electronically by Wisepill™) and sex events. We defined adherence as the proportion of tablets taken as recommended, and coverage as taking ≥1 tablet in the four days before sex and ≥1 tablet within 24 hours after sex.

**Results:**

We randomized 178 MSM (median age 31 years). PrEP coverages were similar in arms D and T (85% vs. 84%, p=0.79) and both were greater than in arm E (74%; p<0.05). Adherence was greater in D (85%) compared with T (79%) or E (65%; p<0.001). Compared with D, the number of doses required for full adherence was reduced by 57% in T and by 80% in E (p<0.001). Among MSM reporting sex in the past week, PBMC tenofovir diphosphate was detectable (≥9.1 fmol/million cells) among 31/31 (100%) in D, 28/29 (96.6%) in T and 28/30 (93.3%) in E at week 10 on study, and in 21/23 (91.3%), 18/19 (94.7%) and 12/14 (85.7%) at week 30, respectively (p=0.54). Median PBMC drug concentrations at week 30 were highest among men in D (102.0 vs. 46.8 vs. 32.9 fmol/million cells for D, T and E, respectively, p<0.001). No HIV infections occurred after randomization.

**Conclusions:**

Compared with the daily regimen, the time-driven dosing regimens offered comparably high PrEP coverage for sex acts for Thai MSM, despite slightly less adherence, while requiring fewer tablets. However, since non-daily dosing results in significantly lower PBMC drug concentrations, stricter adherence is required under these regimens to maintain prophylactic drug concentrations.

**Abstract MOAC0306LB–Table 1:** Results from Bangkok HPTN 067/ADAPT study (n=178)

Characteristic	Daily (D)	Time-driven (T)	Event-driven (E)	Total	p value
N	60	59	59	178	–
Median age	31	28	31	31	–
Number of sex events over full study, not including oral sex	1485	1337	1018	–	0.16
% total events fully covered	85	84	74	–	See text
Total required tablets actually taken	8047	3272	1255	–	<0.001
Total tablets required	9420	4121	1928	–	<0.001
Total % adherence	85	79	65	–	<0.001
% detectable (>9.1 fmol/million) in PBMCs when reporting sex in last 7 days (at 10 weeks of follow up; at 30 weeks of follow up)	100; 91.3	96.6; 94.7	93.3; 85.7	96.7; 91.1	0.54
Median drug concentration in PBMCs (fmol/million cells) when reporting sex in last 7 days (at 10 weeks of follow up; at 30 weeks of follow up)	81.1; 102.0	35.3; 46.8	26.4; 32.9	45.5; 60.7	<0.001

# Sub article: Rapid uptake and adoption of the WHO 2013 consolidated ARV guideline recommendations: paving the way to achieving the 90/90/90 global target

**DOI:** 10.7448/IAS.18.5.20345

**Published:** 2015-07-22

**Authors:** Meg Doherty, Michel Beusenberg, Emil Asamoah-Odei, Frank Lule, Razia Pendse, Massimo Ghidinelli, Ying-Ru Lo, Gabriele Reidner, Martin Donoghoe, Marco Vitoria, Gottfried Hirnschall

**Affiliations:** 1 HIV/Hepatitis Department, World Health Organization, Geneva, Switzerland; 2 AFRO, World Health Organization, Brazzaville, Republic of the Congo; 3 SEARO, World Health Organization, New Delhi, India; 4 PAHO/WHO, Washington, DC, United States; 5 WPRO, World Health Organization, Manila, Philippines; 6 EMRO, World Health Organization, Cairo, Egypt; 7 EURO, World Health Organization, Copenhagen, Denmark

## Abstract

**Introduction:**

Progress towards the ending the AIDS epidemic by 2030 critically depends on adoption of global guidelines that address evidenced based proven approaches to optimally treat all people living with HIV and how to best deliver interventions. With the *2013 Consolidated ARV Guidelines*, WHO successfully launched new policy recommendations on the clinical, operational, programmatic and M&E aspects of HIV treatment and care.

**Methods:**

WHO HQ with regional and country offices, held nine capacity building and dissemination consultations for >100 countries from 2013 to 2014. Through triangulation of baseline surveys, e-surveys with the country MoH HIV focal point and data compiled from the 2014 Global AIDS Response Progress Reporting, we have documented the adoption of priority HIV treatment policies within the 58 WHO focal countries. Data are presented through end 2014.

**Results:**

Within 18 months of the launch of the 2013 consolidated antiretroviral drugs (ARVs) guidelines, 44 of 58 (76%) of focus countries adopted at least one of the major recommendations; globally another 25 countries were in the process of adopting. Sixty percent of focus countries adopted a CD4 count initiation of ≤500 cells/mm^3^, while Brazil, Thailand and Yemen offer treatment to all adults regardless of CD4 cell count. Seventy-one percent adopted a policy to treat all children with HIV <5 years; Ethiopia treats all children <15 years. More than 90% of countries adopted PMTCT Option B/B+; 59% adopted treatment for all HIV serodiscordant couples; and 86% adopted the use of TDF+3TC (or FTC)+EFV as the preferred first-line therapy, granting more people access to better treatment regimens; and 69% planned to implement routine viral load monitoring. Adoption varied by WHO region (Figure 1). An update on the country implementation of these policies will be available in April 2015.

**Conclusions:**

With the *2013 Consolidated ARV Guidelines*, WHO brought together 56 new recommendations across the continuum of HIV treatment and care, and supported countries to more rapidly adopt new policies than ever before; if fully implemented, countries can achieve the 90/90/90 global target.

**Abstract MOAD0101–Figure 1:** WHO ARV Guidelines Adoption by region.

# Sub article: Can the UNAIDS 90–90–90 target be reached? Analysis of 12 national level HIV treatment cascades

**DOI:** 10.7448/IAS.18.5.20346

**Published:** 2015-07-22

**Authors:** Jacob Levi, Alice Raymond, Anton Pozniak, Pietro Vernazza, Philipp Kohler, Nathan Ford, Andrew Hill

**Affiliations:** 1 Department of Public Health, School of Public Health, Imperial College London, London, United Kingdom; 2 St. Stephens Centre, Chelsea and Westminster Hospital, London, United Kingdom; 3 Cantonal Hospital, St. Gallen, Switzerland; 4 Division of Infectious Diseases and Hospital Epidemiology, University Hospital, Zurich, Switzerland; 5 World Health Organisation, Geneva, Switzerland

## Abstract

**Introduction:**

UNAIDS has set the “90–90–90” target for all countries: to diagnose 90% of all HIV positive people, provide antiretrovirals for 90% of those diagnosed and achieve undetectable HIV RNA for 90% of those treated, in every country worldwide by 2020. This translates to at least 73% of all HIV positive people achieving undetectable HIV RNA in every country. We used national level HIV treatment cascades to analyze whether countries have achieved these targets.

**Methods:**

We compared published estimates of HIV treatment cascades across 12 countries in Western and Eastern Europe, North and South America, Australia and sub-Saharan Africa. Cascades were selected based on reliable, generalizable, recently published results from large cross-sectional and longitudinal study cohorts. Data were analyzed in six stages: 1) HIV positive people, 2) Diagnosed, 3) Linked to care, 4) Retained in care, 5) On antiretroviral treatment (ART), 6) Undetectable HIV RNA. Each country level cascade was analyzed to identify whether each stage of the 90–90–90 target was met.

**Results:**

The percentage of HIV positive people who both received ART and achieved undetectable HIV-RNA ranged from 9% (Russia) to 73% (Switzerland). None of the 12 countries met the UNAIDS target of 90% of HIV positive people diagnosed. One country (Switzerland) met the target of 90% of diagnosed people on ART. Five countries (Switzerland, Australia, UK, Denmark and The Netherlands) met the target of 90% of treated people with undetectable HIV RNA. While five Western European countries achieved >50% undetectable HIV-RNA, three Eastern European countries achieved under <20%. USA achieved undetectable HIV-RNA for 30% overall, the lowest amongst high-income countries, comparable to sub-Saharan Africa (29%). The largest fall between stages in the treatment cascades was between prevalence and diagnosis for Switzerland, UK, The Netherlands, Sub-Saharan Africa and Russia; from diagnosis to receiving ART for Australia, Brazil, USA, Georgia and Estonia, and between treatment and achieving undetectable HIV RNA for France and Canada.

**Conclusions:**

Only one of the 12 countries analyzed achieved the UNAIDS 90–90–90 coverage target of 73% of HIV positive people with undetectable HIV RNA. There were disparities between countries. A standardized reporting method should be implemented to facilitate comparisons between countries to better identify gaps and inform policy.

**Abstract MOAD0102–Table 1:** Country level cascades versus 90–90–90 target

Country	% Diagnosed	% On ART	% Undetectable HIV-RNA	Country	% Diagnosed	% On ART	% Undetectable HIV-RNA
UNAIDS 90–90–90 targets for 2020	90	82	73	Brazil (2013)	80	48	40
Switzerland (2012)	84	76	73	Canada (BC) (2011)	71	51	35
Australia (2013)	86	66	62	USA (2013)	82	40	30
United Kingdom (2013)	76	68	61	Sub-Saharan Africa (2013)	45	39	29
Denmark (2010)	85	62	59	Georgia (2012)	52	26	20
The Netherlands (2013)	73	59	53	Estonia (2013)	87	29	19
France (2010)	81	60	52	Russia (2013)	49	11	9

# Sub article: Major outcomes of early HAART programs at CCASAnet sites: “first wave of HAART” study

**DOI:** 10.7448/IAS.18.5.20347

**Published:** 2015-07-22

**Authors:** Marcelo Wolff, Claudia P Cortes, Bryan E Shepherd, Mark Giganti, Catherine Mc Gowan, Caribbean, Central America and South America Network (CCASAnet)

**Affiliations:** 1 School of Medicine, University of Chile, Santiago, Chile; 2 Department of Biostatistics, Vanderbilt University, Nashville, United States

## Abstract

**Introduction:**

Expanded access to HAART in Latin America began slowly in the late 1990s and faster in early 2000s; many antiretrovirals used then, are now outdated and most patients presented with advanced disease stages. Characterizing these patients’ major outcomes (death, loss to follow-up (LTFU), viral suppression, CD4+ cell (CD4) count evolution and regimen changes) after a decade of HAART – not well defined at present – may provide insights into their present and future situation and provide information relevant for the management of patients who initiated HAART more recently.

**Methods:**

The study included adults from six CCASAnet sites: Argentina, Brazil, Chile, Haiti, Honduras and Mexico who initiated HAART before 2004, without exclusion of non-ART-naïve. Status (active, LTFU or dead) for each patient was registered at six-month intervals for up to 10 years, as well as CD4 and viral load (VL) in active patients. The proportions of patients in first, second, third or further HAART regimen or not on HAART were also measured.

**Results:**

In total, 4975 patients (66% male) met inclusion criteria. At HAART initiation, the median age was 35 years, 23% had AIDS and 45% were not ART-naïve. At 1, 3, 5, 7 and 10 years, overall rates of mortality were 4.2, 6.8, 9.0, 10.8 and 13.6% respectively. LTFU rates for the same periods were 2.4, 6.8, 10.9, 14.8 and 24.2% respectively; 62% remained in active care at 10 years (Figure 1). At the end of follow up, 85% of active patients had VL<400 copies/mL (Haiti excluded because VL not regularly measured) and median CD4 increased from 153 to 517 cells/mm^3^_._ After 10 years, only 11% of patients remained active and on their first HAART regimen, 13% were on their second, 12% were on their third and 23% were on their fourth or more regimen. Heterogeneity in outcomes between sites was substantial.

**Conclusions:**

Despite advanced disease and use of mostly old antiretrovirals, a large proportion of first HAART initiators in these Latin American cohorts were alive, in active control, with substantial immune recovery and virologic suppression after 10 years. Early death was a problem as well as persistent LTFU and frequent change of therapy.

**Abstract MOAD0103–Figure 1:** Major outcomes of early HAART programs at CCASAnet.

# Sub article: Integrating HIV-care into primary care clinics improved access to treatment and did not compromise primary health care: province-wide trend analysis over four years during implementation in Free State, South Africa

**DOI:** 10.7448/IAS.18.5.20348

**Published:** 2015-07-22

**Authors:** Angeli Rawat, K E Uebel, David Moore, Annalee Yassi

**Affiliations:** 1 School of Population and Public Health, University of British Columbia, Vancouver, Canada; 2 Free State Department of Health, Bloemfontein, South Africa; 3 British Columbia Centre for Excellence in HIV/AIDS, Vancouver, Canada

## Abstract

**Introduction:**

The integration of HIV-care into primary health care (PHC) clinics is a strategy to expand access to antiretroviral therapy (ART). However, integration may compromise PHC service delivery within weak health systems. We designed a study to examine changes in PHC service provision (pre and post-integration) in public-sector PHC clinics in Free State, South Africa.

**Methods:**

We analyzed administrative data on 15 PHC indicators. The data were collected monthly over a critical four year period as integration was implemented into 131 PHC clinics representing a catchment population of 1.5 million. We defined integration as the month and year the PHC clinic provided comprehensive HIV-care, from testing to treatment to follow-up. We utilized interrupted time series analysis at ±18 and ±30 months from HIV integration in each clinic to identify changes in PHC services post-integration. We conducted sensitivity analyses with linear mixed effect models to study the relationship between HIV service indicators and the PHC indicators.

**Results:**

The number of patients receiving ART in the 131 PHC clinics studied increased from 121 (April 2009) to 57,958 (March 2013). We did not observe any changes in service indicators for 11 of the 15 PHC indicators we examined, However, we did observe decreases in population-level immunization coverage after integration by 0.98% (SE=0.25, p<0.001) at ±18 months and by 1.31% (SE=0.16, p<0.001) at ±30 months. Clinic level immunization coverage also decreased by 33 infants per 100,000 patients (SE=8, p<0.001) at ±30 months. None of these changes were associated with the number of HIV patients at the clinics. We also observed decreases in total clinic visits per year for adults and children under five years old.

**Conclusions:**

Despite an extraordinary increase in patients accessing ART in PHC clinics during our study period, the vast majority of PHC indicators remained unchanged. Our findings suggest that the integration of HIV-care into public-sector PHC clinics is a viable strategy through which to expand access to ART. However, further research is needed to understand how immunization coverage is affected.

# Sub article: Implementation scale up of the Adherence Club model of care to 30,000 stable antiretroviral therapy patients in the Cape Metro: 2011–2014

**DOI:** 10.7448/IAS.18.5.20540

**Published:** 2015-07-22

**Authors:** Lynne Wilkinson, Beth Harley, Shahida Jacobs, Carol Cragg, Ebrahim Kriel, Suhair Solomon, Neshaan Peton, Karen Jennings, Michele Youngleson, Anna Grimsrud

**Affiliations:** 1 Médecins Sans Frontières, Cape Town, South Africa; 2 City Health, Cape Town Municipality, Cape Town, South Africa; 3 Department of Health, Western Cape Government, Cape Town, South Africa; 4 Institute for Healthcare Improvement, Boston, United States; 5 University of Cape Town, Cape Town, South Africa

## Abstract

**Introduction:**

The Adherence Club (AC) model of care was piloted by Médecins Sans Frontières starting in 2007. ACs are groups of approximately 30 stable antiretroviral therapy (ART) patients who met every eight weeks for group support, brief symptom screen and collection of pre-packed ART facilitated by a lay-healthcare worker. Following good pilot outcomes, from 2011 the Cape Metro health district in turn piloted, using a collaborative quality improvement approach and then adopted the model of care. Few data on large-scale implementation of novel models of care exist. We describe the implementation scale-up across the district highlighting key efficiencies and context-specific adaptations to the model.

**Methods:**

We describe the scale-up from January 2011 to December 2014. Data from routine electronic monitoring of the ART programme provide the total number of ART patients retained in care (RIC) while monitoring of AC participation is reported monthly by each AC.

**Results:**

AC implementation expanded over the four-year period with the number of patients retained in AC care increasing annually from 5675 in December 2011 to 30,790 in December 2014 (Figure 1). By December 2014, ACs were offered at 76.1% of ART facilities (51/76) with only 7.5% of ART patients in care at a facility where ACs were not operating. The proportion of patients receiving ART within an AC grew from 7.3% in 2011 to 25.0% by the end of 2014 (Figure 1).

**Conclusions:**

Over a four-year period, the AC model of care was widely accepted and expanded to support a quarter of all patients receiving ART in the district. Adaptations to the model of care supported implementation within the various facility contexts. Some facilities offered ACs at the facility while others decentralized the model to outreach community and home venues. Most used various lay cadres of staff, while some used nurses to facilitate the groups. The model offered efficiencies both to patients and the health system. For ACs to expand to provide quality care to a greater proportion of ART patients, appropriate resources are required. Further research is needed to evaluate the outcomes of AC patients.

**Abstract MOAD0105LB–Figure 1:** Number of patients receiving care within an Adherence Club and the proportion of all ART patients in the Cape Metro health district receiving care within an Adherence Club, January 2011–December 2014.

# Sub article: Phylogenetically estimated HIV diversification rates reveal prevention of HIV-1 by antiretroviral therapy

**DOI:** 10.7448/IAS.18.5.20349

**Published:** 2015-07-22

**Authors:** Jeffrey Joy, Richard Liang, Rosemary McCloskey, Thuy Nguyen, Chanson Brumme, Guillaume Colley, Robert Hogg, Julio Montaner, P Richard Harrigan, Art Poon

**Affiliations:** BC Centre for Excellence in HIV/AIDS, Vancouver, Canada

## Abstract

**Introduction:**

Treatment of HIV infection with antiretrovirals reduces individuals’ plasma viral loads to undetectable levels and in turn decreases the risk of transmission. Despite epidemiological evidence supporting the efficacy of “Treatment as Prevention,” quantifying this success remains a significant challenge. Phylogenetic analysis of viral sequence data can yield crucial insights into epidemic processes, including transmission dynamics. We sought to evaluate the impact of treatment on HIV transmission rates in British Columbia (BC), Canada, using phylogenetic methods.

**Methods:**

We recovered 27,296 anonymized HIV protease and RT sequences from 7747 HIV patients in BC from the BC Centre for Excellence in HIV/AIDS database. Sequences were annotated with: sample collection date, treatment status at sample collection, date of first antiretroviral treatment and risk factor (intravenous drug use (IDU), men having sex with men (MSM) and heterosexual (HET)). Codons associated with known drug resistance were censored from the alignment prior to tree inference. We inferred a set of 1000 maximum likelihood phylogenetic trees. We calculated a lineage level phylogenetic branching rate for each HIV lineage in the trees, which provides an approximate measure of transmission rates. We stratified branching rates by treatment experience and risk factor. To assess the impact of treatment on onward transmission of HIV, we compared the mean HIV branching rate between treatment-experienced and treatment-naive lineages across the BC epidemic as a whole and among risk factors.

**Results:**

Phylogenetic branching rates were significantly lower among treatment-experienced HIV lineages relative to treatment-naive lineages (p<0.001), implying reduced rates of HIV transmission in the former. Importantly, treatment experienced lineages had significantly lower HIV branching rates irrespective of HIV transmission risk factor (p<0.001 for IDU, MSM and HET) or exposure to different antiretroviral drug classes (p<0.001 NRTI, NNRTI, PI), suggesting these results are not driven by penetrance of health care into particular risk groups or therapeutic regimens.

**Conclusions:**

Our results provide independent evidence that antiretroviral HIV treatment has limited the onward transmission of HIV to new hosts. These results are based on a lineage level measure, are measured phylogenetically rather than epidemiologically and are replicated both across different risk exposure categories and different treatment regimens.

# Sub article: Phenotypic properties influencing HIV-1 transmission fitness

**DOI:** 10.7448/IAS.18.5.20350

**Published:** 2015-07-22

**Authors:** Katja Klein, Annette Ratcliff, Gabrielle Nickel, Immaculate Nankya, Mike Lobritz, Yong Gao, Robin Shattock, Eric Arts

**Affiliations:** 1 Department of Microbiology and Immunology, University of Western Ontario, London, Canada; 2 Department of Medicine, Case Western Reserve University, Cleveland, United States; 3 Department of Infectious Diseases, Imperial College London, London, United Kingdom

## Abstract

**Introduction:**

Sexual HIV-1 infection requires penetration of the virus across the mucosal barrier and the establishment of infection in target cells. It is widely accepted that only one or a small number of HIV-1 clones is successfully transmitted from the donor to the recipient. However, little is known about the phenotypic properties of the transmitted virus and the influence the phenotype plays in the genetic bottleneck selection process. Here we evaluated possible phenotypic differences between acute and chronic HIV-1 that may effect transmission fitness.

**Methods:**

We compared the genetic diversity of HIV-1 isolates from the female genital tract with isolates from the blood of the same donor by 454 pyrosequencing of the env region. Furthermore, we generated chimeric viruses from acute and chronic envelope genes using a yeast-based cloning strategy. The chimeric clones were then evaluated for host cell entry and receptor efficiency, sensitivity to entry inhibitors and for replication fitness in PBMCs, T cells and macrophages. Additionally we evaluated the transmission fitness across mucosal tissues by multi-virus competitions.

**Results:**

Both acute and chronic HIV-1 clones showed similar cell entry and receptor efficiency, sensitivity to inhibitors and replication fitness. Sequence analysis revealed that primary infection in the cervix resulted in a highly genetically diverse HIV-1 population, while only one or a few HIV-1 clones are in matched blood. Analysis of mixed competitions of acute and chronic HIV-1 env-clones in *ex vivo* tissue models revealed higher transmission fitness of acute isolates than chronic. We observed that higher transmission fitness was related to a reduced number of conserved *N*-linked glycans on the envelope of acute viruses.

**Conclusions:**

Chronic HIV-1 isolates appear to stay and replicate in the mucosal tissue, while acute isolates are preferentially bound by tissue residing dendritic cells/langerhans cells (DCs/LCs) and are subsequently transmitted to T cells. High levels of mannose binding proteins in tissue and lectins on epithelial cells may be responsible for a passive selection process of HIV-1 with fewer glycans for transmission due to reduced lectin binding.

# Sub article: Population-level spread of immune-driven mutations in HIV-1 polymerase during the North American epidemic

**DOI:** 10.7448/IAS.18.5.20351

**Published:** 2015-07-22

**Authors:** Natalie Nicole Kinloch, Daniel R Macmillan, Anh Q Le, Laura A Cotton, Rosemary Mccloskey, David R Bangsberg, Susan Buchbinder, Mary Carrington, Jonathan Fuchs, P Richard Harrigan, Beryl Koblin, Martin Markowitz, Kenneth Mayer, M J Milloy, Martin T Schechter, Theresa Wagner, Bruce D Walker, Jonathan M Carlson, Art F Y Poon, Zabrina L Brumme

**Affiliations:** 1 Faculty of Health Sciences, Simon Fraser University, Burnaby, Canada; 2 British Columbia Centre for Excellence, Vancouver, Canada; 3 Ragon Institute of MGH, MIT, and Harvard, Cambridge, United States; 4 San Francisco Department of Public Health, San Francisco, United States; 5 Laboratory of Experimental Immunology, Frederick National Laboratory for Cancer Research, Frederick, United States; 6 University of California, San Francisco, United States; 7 New York Blood Center, New York, United States; 8 Aaron Diamond AIDS Research Center, New York, United States; 9 Fenway Community Health, Boston, United States; 10 Harvard Medical School, Cambridge, United States; 11 Faculty of Medicine, University of British Columbia, Vancouver, Canada; 12 Microsoft Research, Seattle, United States

## Abstract

**Introduction:**

HLA-driven HIV-1 immune escape mutations that persist following transmission could gradually spread in the viral population, compromising host antiviral immunity over time. We investigate the extent and correlates of escape mutation accumulation in HIV-1 Polymerase (Pol) sequences in North America from 1979 to present.

**Methods:**

HIV-1 RNA Pol and HLA class I genotyping was performed on 338 Historic (1979–1989) and 278 Modern (2001–2011) specimens from Boston, New York, San Francisco and Vancouver. HLA-associated polymorphisms were defined according to published lists. Historic and modern datasets were also investigated for the presence for novel HLA-associated mutations using phylogenetically- informed methods. Ancestral reconstruction of the HIV-1 epidemic founder sequence was performed using bayesian evolutionary analysis by sampling trees (BEAST) and Hypothesis testing using Phylogenies (HyPhy).

**Results:**

The estimated HIV-1 epidemic founder sequence dated to ~1969 and was near-identical to the modern subtype B consensus, suggesting no historic selective sweeps have occurred to shift the population consensus. No HLA-associated polymorphisms unique to the historic dataset were identified. Nevertheless, pairwise sequence diversity of modern HIV-1 sequences was approximately two-fold greater than historic sequences, with diversification predominating at HLA-associated sites (p<0.0002). N=20 published HLA-associated polymorphisms were investigated for spread over time. Overall, their median “background” frequencies (in individuals lacking the restricting HLA) were 6.6% vs. 16.8% in historic and modern eras respectively (p=0.0004); polymorphism frequencies in reconstructed pre-1979 ancestral sequences were also consistent with gradual spread (p<0.01). No correlation was observed between HLA allele frequency and relative spread of its associated polymorphisms (r=−0.13, p=0.8); rather, polymorphisms restricted by protective HLA alleles exhibited greater relative spread than those restricted by non-protective alleles (r=0.83, p=0.0047). Despite these overall increases, the frequency of many polymorphisms (e.g. B*51-associated RT-I135T) remained consistent throughout the eras. Moreover, at the whole-sequence level, the median extent of adaptation of the typical circulating modern HIV-1 Pol sequence to the average North American host remains 0%, indicating a low overall risk of acquiring HIV-1 harbouring adaptations to one's HLA profile.

**Conclusions:**

Immune escape mutations in HIV-1 Pol have spread significantly in the population since the genesis of the North American epidemic; however, these changes are unlikely to herald immediate consequences for host antiviral immunity on this continent.

# Sub article: Primary resistance against dolutegravir decreases HIV integration

**DOI:** 10.7448/IAS.18.5.20352

**Published:** 2015-07-22

**Authors:** Thibault Mesplède, Kaitlin Anstett, Nathan Osman, Said Hassounah, Jiaming Liang, Yingshan Han, Mark Wainberg

**Affiliations:** McGill University AIDS Centre, Lady Davis Institute for Medical Research-Jewish General Hospital, Montréal, Canada

## Abstract

**Introduction:**

Dolutegravir is an integrase inhibitor that has shown a high genetic barrier against the emergence of resistant strains. No resistance substitution has been observed in treatment-naïve individuals treated with this drug. In tissue culture experiments, we have identified the R263K resistance substitution as a signature substitution for HIV resistance against dolutegravir, an observation that was later confirmed in highly treatment-experienced individuals. Given the importance of DNA integration in the establishment of HIV persistence, we tested the ability of dolutegravir-resistant HIV strains to integrate within human DNA.

**Methods:**

We used an Alu-mediated quantitative PCR to measure levels of integration of dolutegravir-resistant variants in primary human PBMCs. Levels of integration were normalized using the b-actin gene. These experiments were performed using subtype B and C viruses.

**Results:**

Our results show that dolutegravir-resistant variants are impaired in their ability to integrate within human DNA. The integration levels of subtype B and C R263K variants were decreased by 30% and 40% compared to WT viruses, respectively. More important, the addition of several secondary substitutions failed to restore integration to a level comparable to WT and, in some cases, further lowered integration to only 20% of WT.

**Conclusions:**

The relative inability of dolutegravir-resistant variants to integrate within human DNA may contribute to a progressive decrease in the viral reservoir of individuals who develop these substitutions.

# Sub article: HIV-1 integrase variants retarget proviral integration and are associated with disease progression

**DOI:** 10.7448/IAS.18.5.20353

**Published:** 2015-07-22

**Authors:** Jonas Demeulemeester, Sofie Vets, Rik Schrijvers, Paradise Madlala, Marc De Maeyer, Jan De Rijck, Thumbi Ndung'u, Zeger Debyser, Rik Gijsbers

**Affiliations:** 1 Department of Pharmaceutical and Pharmacological Sciences, KU Leuven University, Leuven, Belgium; 2 HIV Pathogenesis Program, Doris Duke Medical Research Institute, Nelson R Mandela School of Medicine, University of KwaZulu-Natal, Durban, South Africa; 3 KU Leuven University, Chemistry, Leuven, Belgium

## Abstract

**Introduction:**

Distinct integration patterns of different retroviruses, including HIV-1, have puzzled virologists for over 20 years. A tetramer of the viral integrase (IN) assembles on the two viral cDNA ends, docks onto the target DNA (tDNA) to form the target capture complex (TCC) and catalyzes viral genome insertion into the host chromatin.

**Methods:**

We combined structural information on the Prototype Foamy Virus TCC with conservation in retroviral IN protein alignments to determine aa-tDNA base contacts. We generated HIV-1 variants based on the observed variability at these positions, assessed replication capacities and performed integration site sequencing to reveal their integration preferences. Finally, we examined their effect on disease progression in a chronic HIV-1 subtype C infection cohort.

**Results:**

We identified retroviral IN amino acids affecting molecular recognition in the TCC and resulting in distinct local tDNA nucleotide biases. These residues also determine the propensity of the virus to integrate into flexible tDNA sequences. Remarkably, natural polymorphisms IN_S119G_ and IN_R231G_ retarget viral integration away from gene dense regions. Precisely these variants were associated with rapid disease progression in a chronic HIV-1 subtype C infection cohort.

**Conclusions:**

Our findings reveal how polymorphisms at positions corresponding to HIV IN_119_ and IN_231_ affect both local and global integration site targeting. Intriguingly, these findings link integration site selection to virulence and viral evolution but also to the host immune response and antiretroviral therapy, since HIV-1 IN_119_ is under selection by HLA alleles and integrase inhibitors.

# Sub article: HIV-1-specific IgG antibody levels correlate with the presence of a specific HLA class II allele to impact acquisition and vaccine efficacy

**DOI:** 10.7448/IAS.18.5.20541

**Published:** 2015-07-22

**Authors:** Heather Prentice, Georgia Tomaras, Daniel Geraghty, Richard Apps, Youyi Fong, Philip Ehrenberg, Morgane Rolland, Gustavo Kijak, Wyatt Nelson, Allan Decamp, Xiaoying Shen, Nicole Yates, Susan Zolla-Pazner, Sorachai Nitayaphan, Supachai Rerks-Ngarm, Punnee Pitisuttithum, Guido Ferrari, David Montefiori, Juliana McElrath, Robert Bailer, Richard Koup, Robert O'Connell, Merlin Robb, Nelson Michael, Jerome Kim, Rasmi Thomas

**Affiliations:** 1 US Military HIV Research Program, Walter Reed Army Institute of Research, Silver Spring, United States; 2 Duke University School of Medicine, Durham, United States; 3 Fred Hutchinson Cancer Research Center, Seattle, United States; 4 Frederick National Laboratory for Cancer Research, Leidos Inc., Frederick, United States; 5 New York University School of Medicine, New York, United States; 6 AFRIMS, Bangkok, Thailand; 7 Ministry of Public Health, Nonthaburi, Thailand; 8 Mahidol University, Bangkok, Thailand; 9 Vaccine Research Center, NIH, Bethesda, United States

## Abstract

**Introduction:**

The RV144 trial had a vaccine efficacy of 31%, and IgG antibodies to HIV-1 Envelope (Env) amino acid positions 120–204 were identified as a predictor of decreased risk of infection. The IgG responses were binding to scaffolded Env antigen comprising the variable loops 1 and 2, flanked by partial regions of the first and second conserved domains. Since HLA class II molecules are expressed on antigen-presenting cells and modulate CD4 T-cell stimulation of antibody production by B cells, we tested whether HLA allotypes influenced vaccine response and efficacy.

**Methods:**

HLA-DRB1, DQB1 and DPB1 were genotyped in 760 individuals. Direct associations of 31 HLA class II alleles on Env (120–204)-specific IgG were compared using linear regression models. Interaction of HLA with IgG response to Env (120–204) was tested for an effect on acquisition by logistic regression.

**Results:**

Higher levels of Env (120–204) IgG antibody directly correlated with the presence of DPB1*13 (p=0.002, q=0.05). Env (120–204)-specific IgG antibody levels also associated with decreased risk of HIV-1 infection only with the presence of DPB1*13 (OR=0.29 per 1-SD increase, p=0.006). Both of these findings were replicated with Env antigens across multiple viral subtypes. Vaccine efficacy increased to 71% among individuals that were DPB1*13+ and had higher levels of Env (120–204)-specific IgG levels relative to the placebos. To delineate the anti-Env antibody responses in DPB1*13+ individuals, we screened overlapping peptides to Env (120–204). Frequency and magnitude of IgG response specifically to Env peptide positions 119–133, which are involved in Env binding to CD4, associated with both presence of DPB1*13 and protection from HIV-1 acquisition among individuals with a DPB1*13 allele. Further evidence that immune responses induced by vaccination in individuals carrying DPB1*13 are different from those without DPB1*13 was apparent in significant viral sequence differences specifically in infected vaccine recipients with DPB1*13.

**Conclusions:**

DPB1*13-associated immune responses to vaccination is associated with decreased risk of HIV-1 acquisition. The specific differences in vaccine-induced responses elicited by individuals with HLA-DPB1*13 should be examined to determine the mechanism of protection of the vaccine. Understanding this HLA class II restricted mechanism will enable improved HIV vaccine design.

# Sub article: Zinc finger nuclease gene editing for functional cure in a non-human primate model of HIV/AIDS

**DOI:** 10.7448/IAS.18.5.20354

**Published:** 2015-07-22

**Authors:** Christopher Peterson, Jianbin Wang, Patricia Polacino, Michael Holmes, Shiu-Lok Hu, Philip Gregory, Hans-Peter Kiem

**Affiliations:** 1 Fred Hutchinson Cancer Research Center, Seattle, United States; 2 Sangamo BioSciences, Richmond, United States; 3 Washington National Primate Research Center, University of Washington, Seattle, United States

## Abstract

**Introduction:**

Nuclease-mediated gene editing in hematopoietic stem cells (HSCs) holds great promise in the cure of HIV infection, but little information is available regarding the feasibility of this approach in large animal models. To better evaluate the function of HSCs following gene editing, we have engineered cells with disrupted CCR5 alleles and assessed engraftment following autologous transplant in the pigtailed macaque, *M. nemestrina*. Disrupted CCR5 alleles in this model should directly protect against infection with simian/human immunodeficiency virus (SHIV). We are evaluating the extent to which CCR5-disrupted cell progeny engraft in macaques and testing whether these cells impede infection by SHIV.

**Methods:**

Zinc finger nucleases (ZFNs) are used to target the CCR5 locus in macaque HSCs. Engraftment and persistence of these autologous stem cells and stem cell-derived lymphoid and myeloid cells are measured *ex vivo* and *in vivo*. Animals are challenged with SHIV virus containing an HIV envelope; to approximate the status of an HIV^+^ patient, three-drug combination antiretroviral therapy (cART) is initiated following viral set point. Animals reach undetectable levels of plasma viremia prior to autologous transplant with gene-edited cells.

**Results:**

CCR5 targeting experiments yield up to 60% gene disruption in CD34^+^ cells *ex vivo*, translating to approximately 5% steady state bulk disruption *in vivo*. Gene-disrupted cells demonstrate long-term, multilineage engraftment in macaques, including comparable levels of disruption in CD3^+^, CD20^+^, CD14^+^ and granulocyte subsets. We also observe biallelic disruption of CCR5 in colony forming assays. Importantly, this approach is equally feasible in SHIV-naïve and in SHIV-infected, cART-suppressed animals. During robust SHIV replication, our preliminary data suggest that CCR5-deleted cells undergo positive selection *in vivo*.

**Conclusions:**

This is the first demonstration of successful long-term multilineage engraftment of ZFN-edited, CCR5-deleted HSCs in a non-human primate (NHP) transplantation model. Our strategy results in robust levels of target gene disruption *in vivo*, yet does not impair HSC engraftment or differentiation. CCR5-deleted cells can undergo positive selection following challenge with SHIV. Our model enables the evaluation of novel therapeutic approaches not only in the context of acute HIV exposure, but also in the clinically relevant setting of pre-existing latent HIV infection.

# Sub article: Crispr/Cas9 gene editing eradicates latent and protects cells against new HIV-1 infection

**DOI:** 10.7448/IAS.18.5.20355

**Published:** 2015-07-22

**Authors:** Rafal Kaminski, Wenhui Hu, Yonggang Zhang, Jonathan Karn, Kamel Khalili

**Affiliations:** 1 Department of Neuroscience, Temple University, Philadelphia, United States; 2 Case Western Reserve, Cleveland, United States

## Abstract

**Introduction:**

A sterilizing cure for HIV-1/AIDS requires a strategy that eliminates all or at least some critical regions of the HIV-1 genome including the promoter positioned within the 5’ LTR of the viral genome from cells serving as a stable reservoir for HIV-1, that is, resting CD4+ T-lymphocytes, macrophages and brain microglia, with no adverse impact on the host cells.

**Methods:**

We have tailored CRISPR/Cas9 gene editing by bioinformatic screening, surveyor assay, and whole genome sequencing and have successfully developed a series of guide RNAs (gRNAs) that, in complex with Cas9 nuclease, effectively and safely eliminate integrated copies of HIV-1 proviral DNA in several human cell culture models. We assessed the impact of our gene editing strategy on viral transcription and replication by measuring the level of a GFP reporter and viral p24, upon reactivation of virus from the latent stage by treatment with phorbol myristate acetate (PMA) and trichostatin A (TSA).

**Results:**

We demonstrated inactivation of HIV-1 gene expression and replication in latently infected T-lymphocytes and promonocytic human cell lines as well as microglial cells upon excising the proviral DNA fragment corresponding to the entire coding sequence of HIV-1 spanning the 5’ to 3’ LTRs from the host chromosome by the CRISPR/Cas9 approach. Further, we demonstrate that the presence of LTR-specific multiplex of guide RNAs in cells expressing Cas9 acts as an efficient inhibitor blocking new HIV-1 infection.

**Conclusions:**

Our findings suggest that the strategy involving the newly developed CRISPR/Cas9 serves as a promising platform that can be advanced for eradication of HIV-1 and a cure for AIDS.

**Abstract TUAA0203–Figure 1:** Eradication of HIV-1 DNA in latently infected cells. A. Treatment of latently infected T-lymphocytes with PMA and TSA activates viral gene expression and expression of GFP reporter in more than 93% of the cells. The presence of gRNAs (LTR A/B) and Cas9 dramatically prevented viral replication. B. Examination of DNA by PCR and direct sequencing verifies removal of integrated proviral DNA from chromosome 16.

# Sub article: Investigating the role of the immune checkpoint receptor TIGIT in T cells during HIV disease progression and as a target for immune restoration

**DOI:** 10.7448/IAS.18.5.20542

**Published:** 2015-07-22

**Authors:** Glen Chew, Tsuyoshi Fujita, Kiera Clayton, Naoto Ishii, Mohamed Abdel-Mohsen, Teri Liegler, Fredrick Hecht, Mario Ostrowski, Cecilia Shikuma, Mark Maurer, Alan Korman, Steven Deeks, Lishomwa Ndhlovu

**Affiliations:** 1 Department of Tropical Medicine, Medical Microbiology, and Pharmacology, University of Hawaii, Manoa, Honolulu, United States; 2 Department of Microbiology and Immunology, Tohoku University, Sendai, Japan; 3 Department of Immunology, University of Toronto, Toronto, Canada; 4 Department of Medicine, University of California, San Francisco, United States; 5 Hawaii Center for HIV/AIDS, University of Hawaii, Manoa, Honolulu, United States; 6 Biologics Discovery California, Bristol-Myers Squibb, Redwood City, United States

## Abstract

**Introduction:**

HIV infection induces a series of phenotypic and functional changes to T cells that eventually results in a state of T-cell exhaustion and failure to control viral replication. T-cell-Ig-and-ITIM-domain (TIGIT) is a recently described negative checkpoint receptor expanded on CD8^+^ T cells during LCMV infection in mice and inhibits anti-viral effector CD8^+^ T-cell activity. We hypothesized that during progressive HIV infection, TIGIT surface expression will mark an expanded population of dysfunctional T cells, and that novel monoclonal antibodies (mAb) targeting TIGIT would restore anti-HIV-specific T-cell responses.

**Methods:**

Surface expression of TIGIT and PD-1 on T cells was measured by flow cytometry from 103 HIV-infected participants (non-controllers (n=20), elite controllers (n=20), antiretroviral (ART) suppressed (n=39), acutely infected (n=24)) and 20 age- and gender-matched HIV-uninfected controls. Quantified cell associated HIV (CA-HIV) DNA and RNA from purified CD4^+^ T cells. Functional characterization of TIGIT^+^ T cells was performed, and *ex vivo* HIV-specific cytokine and proliferative responses were assessed in the presence of mAb targeting TIGIT and/or PD-1 pathways (anti-TIGIT mAb and anti-PD-L1 mAb).

**Results:**

In controls, a median of 28.05% of CD8^+^ T cells was TIGIT^+^ (IQR 24.43, 39.15). In comparison, we found a significant expansion of TIGIT^+^CD8^+^ T cells during chronic (median 57.1%, IQR 42.6, 63.45; p<0.0001) and a non-significant trend in acute HIV infection (40.40%, IQR 28.3, 47.8; p=0.08). TIGIT expression remained elevated despite viral suppression and associated with CD4^+^ CA-HIV DNA. TIGIT^+^ and TIGIT^+^PD-1^+^ CD8^+^ T cells inversely correlated with CD4 count (p=0.0016, r=−0.658; p=0.0024, r=−0.385, respectively). TIGIT was expressed on >50% HIV-specific CD8^+^ T cells; however, TIGIT^+^ T cells failed to produce cytokines in response to HIV antigens. Single blockade of TIGIT led to a significant increase of interferon gamma response to HIV Gag compared to no blockade (p=0.027). Co-blockade of TIGIT and PD-L1 led to greater restoration of HIV-specific CD8^+^ T-cell proliferative responses (4.10%, IQR 1.46, 22.28) than single blockade of TIGIT (3.47%, IQR 1.11, 10.08; p=0.0078) or PD-L1 (3.945%, IQR 1.15, 17.53; p=0.039).

**Conclusions:**

These findings identify TIGIT as a novel marker of dysfunctional HIV-specific T cells and suggest TIGIT along with other checkpoint receptors may be novel curative HIV targets.

# Sub article: Oestrogen blocks HIV re-emergence from latency and points to gender-specific differences in HIV reservoirs

**DOI:** 10.7448/IAS.18.5.20543

**Published:** 2015-07-22

**Authors:** Jonathan Karn, Biswajit Das, Curtis Dobrowolski, Eileen Scully, Steven Deeks, Monica Gandhi, Rowena Johnston

**Affiliations:** 1 Department of Molecular Biology and Microbiology, Case Western Reserve University School of Medicine, Cleveland, United States; 2 Brigham and Women's, Boston, United States; 3 Massachusetts General Hospital, Boston, United States; 4 UCSF School of Medicine, San Francisco, United States; 5 amfAR, The Foundation for AIDS Research, New York, United States

## Abstract

**Introduction:**

Unbiased shRNA library screens have been used to identify novel genes and pathways that are required to maintain HIV latency and/or play an essential role in HIV transcription. One of the most prominent and robust “hits” was the oestrogen receptor type 1 (ESR-1).

**Methods:**

The activities of ESR-1 agonists, antagonists and oestrogen on proviral reactivation were studied in transformed and primary cell models of latency and in patient cells.

**Results:**

Specific antagonists of ESR-1, such as Tamoxifen and Fulvestrant, are weak proviral activators but sensitize latently infected cells to very low doses of the proviral activators TNF-α (NF-κB inducer) and SAHA (HDAC inhibitor). By contrast, a selective ESR-1 agonist propylpyrazoletriol and the broader spectrum ESR-1 agonist diethylstilbestrol strongly suppress both TNF-α and SAHA reactivation. In contrast to the ESR-1 antagonists, ESR-2 antagonists were not effective inducers of HIV expression in cell models. Co-activator 3 (SRC-3) is an upstream modulator of ESR-1, which was also identified as a hit in the shRNA screen. Blocking of SRC-3 by its inhibitor Gossypol also induces latent proviruses. Consistent with these results, specific knock-down of ESR-1 in Jurkat 2D10 cells with shRNA constitutively re-activates the latent provirus. In the HAART-treated patient samples, there was a modest increase of spliced HIV env mRNA when resting memory cells were treated with the ESR antagonists Fulvestrant or Tamoxifen alone. Proviral reactivation by ESR antagonists was synergistically increased by SAHA. By contrast, β-estradiol at concentrations in the physiological range led to dramatic reductions in proviral reactivation efficiencies. This is consistent with earlier observations that high levels of β-estradiol can block HIV replication.

**Conclusions:**

ESR-1 is a pharmacologically attractive target that can be exploited in the design of therapeutic strategies aimed at eradication of the latent reservoir. Our results show that drugs targeting ESR-1 can be used to either promote the re-activation of latent proviruses (antagonists) or limit their responses (agonists). The profound effects of β-estradiol on HIV reservoir reactivation suggest that there may be gender-specific differences in HIV reservoirs and highlight the need to tailor latency reactivation strategies for both men and women.

# Sub article: Atazanavir/ritonavir 200/100 mg is non-inferior to atazanavir/ritonavir 300/100 mg in virologic suppressed HIV-infected Thai adults: a multicentre, randomized, open-label trial: LASA

**DOI:** 10.7448/IAS.18.5.20356

**Published:** 2015-07-22

**Authors:** Torsak Bunupuradah, Sasisopin Kiertiburanakul, Anchalee Avihingsanon, Ploenchan Chetchotisakd, Malee Techapornroong, Niramon Leerattanapetch, Pacharee Kantipong, Chureeratana Bowonwatanuwong, Sukit Banchongkit, Virat Klinbuayaem, Sripetcharat Mekviwattanawan, Sireethorn Nimitvilai, Supunnee Jirajariyavej, Wisit Prasithsirikul, Warangkana Munsakul, Sorakij Bhakeecheep, Sushada Chaivooth, Praphan Phanuphak, David A Cooper, Tanakorn Apornpong, Stephen J Kerr, Sean Emery, Kiat Ruxrungtham, LASA Study Group

**Affiliations:** 1 HIV-NAT, Thai Red Cross – AIDS Research Centre, Bangkok, Thailand; 2 Faculty of Medicine Ramathibodi Hospital, Mahidol University, Bangkok, Thailand; 3 Division of Allergy and Clinical Immunology, Department of Medicine, Chulalongkorn University, Bangkok, Thailand; 4 Department of Medicine, Khon Kaen University, Khon Kaen, Thailand; 5 Prapokklao Hospital, Chanthaburi, Thailand; 6 Khon Kaen Hospital, Khon Kaen, Thailand; 7 Chiangrai Prachanukroh Hospital, Chiangrai, Thailand; 8 Chonburi Hospital, Chonburi, Thailand; 9 Rayong Hospital, Rayong, Thailand; 10 Sanpatong Hospital, Chiang Mai, Thailand; 11 Pranangklao Hospital, Nonthaburi, Thailand; 12 Nakhon Phatom Hospital, Nakhon Phatom, Thailand; 13 Taksin Hospital, Bangkok, Thailand; 14 Bamrasnaradura Infectious Disease Institute, Nonthaburi, Thailand; 15 Faculty of Medicine, Vajira Hospital, University of Bangkok Metropolitan Administration, Bangkok, Thailand; 16 National Health Security Office, Nonthaburi, Thailand; 17 Kirby Institute for Infection and Immunity in Society, University of New South Wales, Sydney, Australia

## Abstract

**Introduction:**

Asian HIV-infected patients generally experience higher systemic exposure to HIV protease inhibitors (PIs). We compared the efficacy and safety of switching to lower versus standard dose of atazanavir/ritonavir (ATV/r) in virologically suppressed second-line patients.

**Methods:**

Patients with plasma HIV-RNA (pVL) <50 copies/mL, ALT <200 IU/L and creatinine clearance (Crcl) ≥60 mL/min while using PI-based regimens were randomized to ATV/r 200/100 mg (A200) vs. ATV/r 300/100 mg (A300) once daily with 2NRTIs at 14 sites in Thailand.

Patients were followed every 12 weeks until week-48. Virological failure (VF) was defined as had confirmed pVL >200 copies/mL. Patients in ATV200 with VF resumed standard dose PI-based regimens. Treatment groups were regarded as non-inferior if the lower limit of the 95% confidence interval (95% CI) for the difference in VF was above −10% in an intention-to-treat (ITT) analysis at 48 weeks.

**Results:**

A total of 559 patients were randomized (ATV200; N=279 vs. ATV300; N=280). At baseline, 85% used lopinavir/ritonavir, mean age was 42 years, body weight was 59 kg, CD4 was 539 cells/mm^3^ and total bilirubin was 0.85 mg/dL.

At week 48, by ITT, the proportion of patients in ATV200 vs. ATV300 with pVL <200 copies/mL (difference, 95% CI) was 97.1% vs. 96.4% (0.68, −2.29 to 3.65), the proportions with pVL <50 copies/mL were 93.4% vs. 91.7% (1.71, −2.67 to 6.09). In per-protocol analyses, the proportions with pVL <200 copies/mL were 98.5% vs. 99.2% (−0.72, −2.6 to 1.16). Only one ATV200 recipient developed major resistance (I50 L) to ATV.

Discontinuation from randomized therapy was 8 (2.9%) in ATV200 (1 death, 2 VF, 1 jaundice, 2 rash, 2 others) and 21 (7.5%) in ATV300 (2 deaths, 7 jaundice, 7 rash, 5 others) (p=0.01). At week-48, there was no difference between treatment arms in CD4, total cholesterol, triglyceride and Crcl (all p>0.1). Comparing ATV200 vs. ATV300, the number (%) of patients with total bilirubin >3.2 mg/dL was 27 (10%) vs. 46 (17%) respectively (p=0.017).

**Conclusions:**

A lower dose of ATV/r-based regimens in Thais is non-inferior compared to standard dose ATV/r. Higher dose ATV was associated with higher rates of treatment discontinuation. ATV/r 200/100 mg can be recommended as part of routine care for Asian adults who have well-controlled HIV infection on a PI-based regimen.

# Sub article: Switching from a tenofovir disoproxil fumarate (TDF)-based regimen to a tenofovir alafenamide (TAF)-based regimen: data in virologically suppressed adults through 48 weeks of treatment

**DOI:** 10.7448/IAS.18.5.20357

**Published:** 2015-07-22

**Authors:** Tony Mills, Jamie Andrade, Giovanni Diperri, Jan Van Lunzen, Ellen Koenig, Richard Elion, Matthias Cavassini, Jose Valdez Madruga, Jason Brunetta, David Shamblaw, Edwin Dejesus, Calvin Cohen, Andrew Plummer, YaPei Liu, Scott McCallister

**Affiliations:** 1 Anthony Mills MD, Inc., Los Angeles, United States; 2 Hospital Civil de Guadalajara, Guadalajara, Mexico; 3 University Hospital Amedeo De Savoia, Turin, Italy; 4 Universitaetsklinikum Hamburg Eppendorf, Hamburg, Germany; 5 Instituto Dominicano de Estudios Virologicos IDEZ, Santo Domingo, Dominican Republic; 6 Whitman-Walker Health, Washington, United States; 7 Centre Hospitalier Universitaire Valdois, Lausanne, Switzerland; 8 Centro de Referencia e Treinamento Em DST/AIDS, Sao Paolo, Brazil; 9 Maple Leaf Research, Toronto, Canada; 10 La Playa Medical Group, San Diego, United States; 11 Orlando Immunology Center, Orlando, United States; 12 Community Research Initiative of New England, Boston, United States; 13 Gilead Sciences, Foster City, United States

## Abstract

**Introduction:**

Despite a favourable efficacy and safety profile, TDF-based regimens may be associated with renal toxicity and reduced bone mineral density (BMD). TAF is a novel tenofovir prodrug in which TFV plasma levels are 90% lower than seen with TDF, thereby reducing off-target side effects. Week 48 data in patients switching to a once-daily fixed dose combination regimen containing elvitegravir 150 mg, cobicistat 150 mg, emtricitabine 200 mg and TAF 10 mg (E/C/F/TAF) are described.

**Methods:**

Virologically suppressed adults (HIV-1 RNA <50 copies/mL) with normal renal function taking one of four different TDF-based regimens for at least 48 weeks were randomized 2:1 to receive E/C/F/TAF or to retain their prior TDF-based regimen. Following randomization, all treatments were open-label.

**Results:**

Of 1196 patients completing at least 48 weeks of treatment, 799 received E/C/F/TAF and 397 received their prior TDF regimen: E/C/F/TDF, 31.9%; EFV/FTC/TDF, 26.1%; ATV/RTV + FTC/TDF, 26.8%; ATV/COBI + FTC/TDF, 15.0%. Virologic success <50 copies/mL occurred in 95.6% on E/C/F/TAF and 92.9% on FTC/TDF + 3rd Agent (weighted difference: 2.7%; 95% CI: −0.3% to +5.6%), with virologic failure in 1.1% and 1.3% of patients, respectively. General safety was similar between the arms. The mean percent change (SD) in hip BMD: +1.95% (3.0) for E/C/F/TAF and −0.14% (3.0) for FTC/TDF+3rd Agent (p<0.001); the mean percent change (SD) in spine BMD: +1.86% (3.1) for E/C/F/TAF and −0.11% (3.7) for FTC/TDF+3rd Agent (p<0.001). There were no cases of Fanconi Syndrome on E/C/F/TAF and one case on FTC/TDF+3rd Agent. For patients on either a COBI or RTV boosted regimen prior to randomization, the estimated GFR increased 1.8 mL/min for E/C/F/TAF and decreased 3.7 mL/min for FTC/TDF+3rd Agent (p<0.001). As shown in the table, multiple measures of quantitative proteinuria, including tubular proteinuria, had statistically significant improvements for patients switching to E/C/F/TAF as compared with those retaining their prior TDF-based regimen.

**Conclusions:**

These 48 week data demonstrate that patients who switch from a TDF-based regimen to E/C/F/TAF maintain high efficacy, have statistically significant increases in BMD and have statistically significant improvements in multiple tests of renal function, as compared with patients remaining on their prior TDF-based regimen.

**Abstract TUAB0102–Table 1:** Changes in proteinuria and tubular proteinuria

Median % change baseline to Week 48	E/C/F/TAF	FTC/TDF+3rd agent	Significance
Urine protein: creatinine (UPCR)	−18.5%	+9.4%	p<0.001
Urine albumin: creatinine (UACR)	−18.4%	+5.3%	p<0.001
Retinol binding protein: creatinine (RBP: CR)	−32.9%	+15.7%	p<0.001
Beta-2-microglobulin: creatinine (B2MG: CR)	−49.2%	+14.4%	p<0.001

# Sub article: Subjects with renal impairment switching from tenofovir disoproxil fumarate to tenofovir alafenamide have improved renal and bone safety through 48 weeks

**DOI:** 10.7448/IAS.18.5.20358

**Published:** 2015-07-22

**Authors:** Samir Gupta, Anton Pozniak, Josa Arribas, Frank Post, Mark Bloch, Joseph Gathe, Paul Benson, Joseph Custodio, Michael Abram, Xuelian Wei, Andrew Cheng, Scott McCallister, Marshall Fordyce

**Affiliations:** 1 Indiana University, Indianapolis, United States; 2 Chelsea and Westminster Hospital NHS Foundation Trust, London, United Kingdom; 3 Hospital La Paz, Madrid, Spain; 4 Kings College Hospital NHS Foundation Trust, London, United Kingdom; 5 Holdsworth House Medical Practice, Sydney, Australia; 6 Baylor College of Medicine, Houston, United States; 7 Be Well Medical Center, Berkley, United States; 8 Gilead Sciences Inc., Foster City, United States; 9 Gilead Sciences, Foster City, United States

## Abstract

**Introduction:**

Tenofovir (TFV) is renally eliminated, and the prodrug, tenofovir disoproxil fumarate (TDF), has been associated with renal toxicity and reduced bone mineral density (BMD). Tenofovir alafenamide (TAF) is a novel prodrug of tenofovir (TFV) that results in 90% lower plasma TFV levels as compared to TDF. The safety and efficacy of a once-daily single tablet regimen of elvitegravir, cobicistat, emtricitabine and TAF (E/C/F/TAF) was assessed in HIV-1 infected patients with mild to moderate renal impairment.

**Methods:**

Virologically suppressed adults with stable renal impairment (eGFR_CG_ 30–69 mL/min) had their treatment switched from both TDF- and non-TDF-containing regimens to open-label E/C/F/TAF. Week 48 safety data by pre-switch TDF use are presented.

**Results:**

Of 242 subjects switched to E/C/F/TAF (mean age 58 years (range: 24–82), 18% Black, 39% HTN and 14% DM) 158 subjects (65%) were taking TDF-containing regimens prior to switch. At Week 48, the median (Q1, Q3) change from baseline for eGFR_C-G_ was +0.2 (−5.8, 6.3) mL/min (p=0.81) and for eGFR-cystatin C was +2.7 (−6.2, 14.1) mL/min/1.73 m2 (p=0.003). The following measures of renal tubular function improved significantly (p<0.001 for all) for subjects switching from TDF-containing regimens to E/C/F/TAF: quantified proteinuria (UPCR, median (Q1, Q3) % change; −55 (−70, −28)), albuminuria (UACR, median (Q1, Q3) % change; −61 (−81, −27)), retinol binding protein (RBP:Cr, median (Q1, Q3) % change; −82 (−95, −55)) and beta-2-microglobulin (β-2-Mg:Cr, median (Q1, Q3) % change; −89 (−97, −61)). The prevalence of clinically significant proteinuria (UPCR >200 mg/g) and albuminuria (UACR ≥30 mg/g) decreased from 48 to 13% and from 56 to 22%, respectively. Significant increases in mean% change in hip (+1.29%) and spine (+2.60%) BMD were observed at 48 weeks (p<0.001 for both). Subjects taking non-TDF based regimens pre-switch (n=84) had no significant changes from baseline measures of renal function or BMD.

**Conclusions:**

Subjects with mild and moderate renal impairment (eGFR 30 to 69 mL/min) who switched from TDF-containing regimens to once daily single-tablet E/C/F/TAF experienced improvements in multiple assessments of renal and bone safety through 48 weeks. These data support the safety of E/C/F/TAF in patients with impaired renal function.

# Sub article: Efficacy and safety of doravirine 100 mg QD vs. efavirenz 600 mg QD with TDF/FTC in ART-naive HIV-infected patients: week 24 results

**DOI:** 10.7448/IAS.18.5.20359

**Published:** 2015-07-22

**Authors:** Jose Gatell, Francois Raffi, Andreas Plettenberg, Don Smith, Joaquin Portilla, Christian Hoffmann, Keikawus Arasteh, Melanie Thompson, Debbie Hagins, Javier Morales-Ramirez, Xia Xu, Hedy Teppler

**Affiliations:** 1 University of Barcelona, Barcelona, Spain; 2 Department of Infectious Diseases, Hotel-Dieu University Hospital, Nantes, France; 3 ifi-Institute for Infections, Hamburg, Germany; 4 Albion Centre, Sydney, Australia; 5 University Miguel Hernandez, Alicante, Spain; 6 ICH Study Center, Hamburg, Germany; 7 EPIMED/Vivantes Auguste-Viktoria-Klinikum, Berlin, Germany; 8 AIDS Research Consortium of Atlanta, Atlanta, United States; 9 Chatham County Health Department, Savannah, United States; 10 Clinical Research Puerto Rico, San Juan, Puerto Rico; 11 Merck & Co., Inc., Kenilworth, United States

## Abstract

**Introduction:**

Doravirine (DOR), an investigational NNRTI with a novel resistance profile, was compared with efavirenz (EFV) in a double-blind, randomized, 2-part study in ART-naïve HIV-infected patients who also received tenofovir/emtricitabine (TDF/FTC). In Part 1 (dose selection), DOR at 25, 50, 100 and 200 mg QD showed rates of virologic suppression similar to EFV 600 mg QD; DOR 100 mg was selected for ongoing evaluation. Part 2 enrolled additional patients to receive DOR 100 mg or EFV. Using data from Parts 1+2 combined, DOR 100 mg showed significantly fewer CNS AEs than EFV at week 8.

**Methods:**

Week 24 efficacy and safety results were analyzed for all patients who received DOR 100 mg or EFV in Part 1 (n=42 per group) and Part 2 (n=66 per group) combined. Patients were stratified at randomization by screening RNA ≤ or >100,000 copies/mL. Primary endpoints were the proportion of patients with HIV RNA <40 c/mL (efficacy) and the proportion of patients with pre-specified CNS events (safety).

**Results:**

Of the 108 patients randomized and treated per group, mean baseline RNA was 4.6 log_10_ c/mL in both the DOR and EFV groups, and mean CD4 counts were 432 and 448 cells/mm^3^, respectively. Discontinuations in the DOR and EFV groups, respectively, were 4.6 and 12.0%.

The most common drug-related clinical AEs in the DOR and EFV groups, respectively, were nausea (7.4%; 5.6%), dizziness (6.5%; 25.0%), abnormal dreams (5.6%; 14.8%), nightmares (4.6%; 8.3%) and sleep disorder (3.7%; 6.5%). Drug-related AEs leading to discontinuation were hallucination for DOR (n=1) and dysesthesia, hallucination, drug eruption, dizziness and disturbance in attention for EFV (n=5). The most common CNS AEs (all causality) were dizziness (DOR 9.3%; EFV 27.8%), insomnia (7.4%; 2.8%), abnormal dreams (6.5%; 17.6%) and nightmares (6.5%; 8.3%). Lab abnormalities of Grade 2 or greater were uncommon in both groups.

**Conclusions:**

DOR 100 mg qd demonstrated antiretroviral activity and immunological effect similar to EFV (each with TDF/FTC) and was generally safe and well tolerated during 24 weeks of treatment in ART-naïve, HIV-1 infected patients. Treatment-emergent CNS AEs through week 24 were significantly less common in the DOR group than in the EFV group.

**Abstract TUAB0104–Table 1:** Week 24 Efficacy, including subgroup responses by screening RNA ≤ or >100,000 c/mL

Endpoint	DOR† (N=108)	EFV† (N=108)	Difference [DOR-EFV] (95% CI)
HIV RNA<40c/mL††	72.2%	73.1%	−1.2 (−13.0, 10.5)
screening RNA ≤100K§ (n=66, 63)	83.3%	85.7%	−2.4 (−15.3, 10.6)
screening RNA >100K§ (n=38, 38)	60.5%	65.8%	−5.3 (−26.4, 16.4)
HIV RNA<200c/mL††	88.9%	87.0%	1.9 (−7.0, 11.0)
screening RNA≤100K§ (n=66, 63)	92.4%	92.1%	0.4 (−9.8, 10.8)
screening RNA >100K§ (n=38, 38)	92.1%	94.7%	−2.6 (16.5, 10.7)
Mean change in CD4 count§	154/mm^3^	146/mm^3^	8 (−37, 52)

† with TDF/FTC.

†† Non-completer=Failure (NC=F) approach to missing data.

§ Observed Failure (OF) approach to missing data.

**Abstract TUAB0104–Table 2:** Week 24 Clinical Adverse Event (AE) Summary & Primary Safety Analysis (CNS AEs)

Proportion of patients with:	DOR† (N=108)	EFV† (N=108)	Difference [DOR-EFV] (95% CI)
One or more AEs	75.9%	84.3%	−83 (−19.1, 2.4)
Drug-related AEs	27.8%	55.6%	−27.8 (−39.9, 14.8)
Serious AE	0.9%	4.6%	−3.7 (−9.6, 0.9)
Serious drug-related AEs	0%	0.9%	−0.9 (−5.1, 2.5)
Discontinued due to AEs	0.9%	5.6%	−4.6 (−10.8, 0.1)
One or more CNS AEs	26.9%	46.3%	−19.4 (−31.7, 6.6)*

† with TDF/FTC.

* Pre-specified safety hypothesis, p<0.001.

# Sub article: Raltegravir for prevention of mother-to-child transmission of HIV

**DOI:** 10.7448/IAS.18.5.20360

**Published:** 2015-07-22

**Authors:** Marie-Julie Trahan, Valérie Lamarre, Marie-Elaine Metras, Normand Lapointe, Fatima Kakkar

**Affiliations:** 1 Université de Montréal, Faculté de Médecine, Montreal, Canada; 2 CHU Ste-Justine, Département des Maladies Infectieuses, Montreal, Canada; 3 Centre Maternel et Infantile sur le Sida, Montreal, Canada; 4 CHU Ste-Justine, Département de Pharmacie, Montreal, Canada; 5 CHU Ste-Justine, Département d'Immunologie, Montreal, Canada

## Abstract

**Introduction:**

Raltegravir (RAL), though currently category C in pregnancy and not recommended for use in newborns, has been used in exceptional cases for prevention of mother-to-child-transmission (PMTCT). We report on the outcomes of 14 infants exposed *in utero* to RAL and the first newborn to be treated with RAL for six weeks for PMTCT.

**Methods:**

Infants born to mothers treated with RAL during pregnancy from the Centre Maternel et Infantile sur le Sida (CMIS) mother-child cohort between 2010 and 2014 were included in the study. RAL levels were tested on the first available stored plasma sample after birth, and in the treated newborn, therapeutic drug monitoring was done at weekly intervals.

**Results:**

In RAL-exposed infants, RAL was given to mothers at standard dosing of 400 mg BID, started at a mean GA of 30 weeks (range pre-conception-37.5 weeks). Indications for RAL included drug resistance and/or detectable viral load in the third trimester. Mean GA was 38.5 weeks (±1.76), and mean birthweight was 3200 g (±540). There were no clinical adverse events noted among RAL-exposed infants (mean follow-up time 119 weeks, range 48–144), and all were confirmed HIV negative. RAL levels tested in two exposed newborns at 16 and 30 hours of life were detectable at 0.9345 mg/L and 0.0381 mg/L, respectively, and undetectable in six other infants tested at days 4–14. RAL granules for suspension (Merck, special access) were obtained for prophylaxis of a term newborn (39 weeks GA) from a mother with multidrug-resistant virus and started at 1.5 mg/kg BID, along with zidovudine and lamivudine at standard doses. RAL levels were consistently above the targeted trough for treatment (0.02 mg/L) (Table 1) for the duration of therapy. RAL was well tolerated and at follow-up, the infant was confirmed HIV negative.

**Conclusions:**

RAL in late pregnancy had no adverse effects on infants exposed in utero. RAL treatment in the newborn at doses of 1.3–1.6 mg/kg BID was well tolerated and resulted in therapeutic drug levels. Given detectable levels of RAL in the first 30 hours of life in exposed infants, the timing and role of RAL in PMTCT should further be considered.

**Abstract TUAB0105–Table 1:** Raltegravir levels in a treated newborn

Day of life	Weight (kg)	Dose	mg/kg/dose	Trough (hours)	Trough level	Peak (hours)	Peak level	Adjusted
6	3.115	5 mg BID	1.61	11.67	0.36	1.97	0.87	No
9	3.220	5 mg BID	1.55	11.25	0.75	1.25	0.15	No
20	3.565	5 mg BID	1.40	12	0.07	1.17	0.33	No
27	3.835	5 mg BID	1.30	11	0.06	1.15	0.02	Increased to 6 mg BID
40	4.275	6 mg BID	1.40	N/A	N/A	N/A	N/A	Stopped

# Sub article: Second-generation HIV-1 maturation inhibitor BMS-955176: antiviral activity and safety with atazanavir±ritonavir

**DOI:** 10.7448/IAS.18.5.20544

**Published:** 2015-07-22

**Authors:** Carey Hwang, Dirk Schürmann, Christian Sobotha, Marta Boffito, Heather Sevinsky, Neelanjana Ray, Palanikumar Ravindran, Hong Xiao, Mark Krystal, Ira Dicker, Dennis Grasela, Max Lataillade, for the BMS HIV Global Development Team

**Affiliations:** 1 Bristol-Myers Squibb, Research and Development, Princeton, United States; 2 Charité Research Organisation GmbH, Berlin, Germany; 3 St. Stephen's Centre, Chelsea and Westminster Hospital, London, United Kingdom; 4 Research and Development, Bristol-Myers Squibb, Wallingford, United States

## Abstract

**Introduction:**

BMS-955176 is a second-generation HIV-1 maturation inhibitor that targets the HIV-1 Gag polyprotein, inhibiting the last protease cleavage event between capsid protein p24 and spacer peptide 1, resulting in the release of immature, non-infectious virions. Ten days of BMS-955176 monotherapy resulted in maximum median declines in HIV-1 RNA that plateaued at ~1.64 log_10_ c/mL at doses between 40 mg and 120 mg once daily (QD). Two drug combination studies *in vitro* demonstrated that BMS-955176+atazanavir (ATV) had an additive effect. Due to the proximity of their sites of inhibition in the virus life cycle and the potential for synergy, we assessed the antiviral activity and safety of BMS-955176 with ATV±ritonavir (RTV) for 28 days in HIV-1-infected subjects. In addition, this combination is being further evaluated to potentially serve as part of a booster-sparing and nucleot(s)ide-sparing strategy.

**Methods:**

AI468002 (NCT01803074) was a Phase 2a, randomized, multipart trial. In Part B, 28 HIV-1 subtype B-infected subjects (HIV-1 RNA≥5000 c/mL, CD4+ T-cell counts≥200 cells/µL) were randomized 2:2:2:1 to four treatment groups (all QD): BMS-955176 40 mg+ATV 400 mg; BMS-955176 40 mg+ATV 300 mg+RTV 100 mg; BMS-955176 80 mg+ATV 400 mg; and a standard-of-care (SOC) control of tenofovir disoproxil fumarate 300 mg+emtricitabine 200 mg (fixed-dose combination)+ATV 300 mg+RTV 100 mg.

**Results:**

Median change in HIV-1 RNA at Day 29 was −1.66, −1.99, −2.18 and −2.22 log_10_ c/mL, and maximum median change in HIV-1 RNA from baseline to end of study/discharge (Day 42) was −1.86, −2.20, −2.23 and −2.39 log_10_ c/mL, for BMS-955176 40 mg+ATV 400 mg, BMS-955176 40 mg+ATV 300 mg+RTV 100 mg, BMS-955176 80 mg+ATV 400 mg, and the SOC control, respectively (Table 1 and Figure 1). There were no deaths, serious adverse events (SAEs), or AEs leading to discontinuation. Furthermore, the median bilirubin level was below the upper limit of normal for subjects receiving unboosted ATV with BMS-955176, in contrast to the level observed for subjects receiving BMS-955176 40 mg+ATV+RTV or SOC.

**Conclusions:**

In this study, BMS-955176 80 mg+ATV and 40 mg+ATV+RTV had similar maximum median declines in HIV-1 RNA compared with the SOC control. BMS-955176 with ATV±RTV was generally well tolerated. A Phase 2b study investigating BMS-955176 in a booster-sparing and nucleot(s)ide-sparing regimen in treatment-experienced patients will begin in Q2 2015.

**Abstract TUAB0106LB–Table 1:** Changes in HIV-1 RNA from baseline

	BMS-955176 (40 mg QD)+ATV (400 mg QD)	BMS-955176 (40 mg QD)+ATV (300 mg QD)+RTV (100 mg QD)	BMS-955176 (80 mg QD)+ATV (400 mg QD)	Tenofovir disoproxil fumarate (300 mg QD)+emtricitabine (200 mg QD) (fixed-dose combination)+ATV (300 mg QD)+RTV (100 mg QD)
N	8	8	8	4
Maximum decline in HIV-1 RNA (log_10_ c/mL); median (min, max)	−1.86 (−1.49, −2.37)	−2.20 (−1.24, −3.52)	−2.23 (−1.87, −2.68)	−2.39 (−1.83, −3.04)
Median decline in HIV-1 RNA (log_10_ c/mL) on Day 29 (min, max)	−1.66 (−1.19, −2.04)	−1.99 (−1.04, −3.32)	−2.18 (−1.53, −2.68)	−2.22 (−1.83, −2.84)

**Abstract TUAB0106LB–Figure 1:** Median change in HIV-1 RNA (log_10_ c/mL) over time.

# Sub article: A longitudinal analysis of liver fibrosis progression among NNRTI and PI users in the Canadian co-infection cohort study

**DOI:** 10.7448/IAS.18.5.20361

**Published:** 2015-07-22

**Authors:** Laurence Brunet, Erica EM Moodie, James Young, Sharon Walmsley, Mark Hull, Curtis Cooper, Marina B Klein, Canadian Co-Infection Cohort Study Investigators

**Affiliations:** 1 Epidemiology & Biostatistics, McGill University, Montreal, Canada; 2 Basel Institute for Clinical Epidemiology and Biostatistics, University Hospital Basel, Basel, Switzerland; 3 University Health Network, University of Toronto, Toronto, Canada; 4 BC Centre for Excellence in HIV/AIDS, Vancouver, Canada; 5 The Ottawa Hospital-General Campus, Ottawa, Canada; 6 Medicine, McGill University, Montreal, Canada; 7 Chronic Viral Illness Service, McGill University Health Centre, Montreal, Canada

## Abstract

**Introduction:**

Both protease inhibitors (PI) and non-nucleoside reverse transcriptase inhibitors (NNRTI) have been associated with acute hepatotoxicity, but their long-term effect on liver fibrosis remains uncertain. We explored rates of change in liver fibrosis as measured by the aspartate-to-platelet ratio index (APRI) among HIV-hepatitis C (HCV) co-infected users of modern PI- or NNRTI-based regimens.

**Methods:**

Data from a Canadian prospective multicentre cohort were analyzed for 397 HCV PCR+ persons who initiated antiretroviral therapy in or after 2000, with regimens at cohort entry comprised of a backbone of either Tenofovir/Emtricitabine or Abacavir/Lamivudine with a PI or NNRTI as the anchor agent. The natural logarithm of the APRI score was the outcome of interest. Three multivariate linear regression analyses with generalized estimating equations were performed. Analysis 1 (intention-to-treat) used baseline exposure to PI or NNRTI; analysis 2 (per protocol) was restricted to persons with a viral load under 1000 copies/mL and censored participants when the class of anchor agent was changed; analysis 3 (as treated) allowed for changes in the class of anchor agent during follow-up.

**Results:**

At cohort entry, 74% of participants were male, the median age was 44 years and 56% had used alcohol in the past six months. Therapy was started a median of 1.9 years before cohort entry (IQR: 0.3, 5.0), 70% used a PI and 69% were on a backbone of Tenofovir/Emtricitabine. PI use was associated with a median increase in APRI per 5 years of 16% (95% CI: 3%, 30%) in Analysis 1, 16% (95% CI: 0%, 32%) in Analysis 2 and 13% (95% CI: −1%, 27%) in Analysis 3. NNRTI use was not significantly associated with change in APRI in any of the three analyses, as shown in the Table.

**Conclusions:**

PI use seems to be associated with a faster progression of liver fibrosis, as measured by the median change in APRI score over five years. The consistency of estimates across the three analyses suggests that this is not the result of the type of patients using PI-based regimens, although we could not account for all patient characteristics influencing the choice of an anchor agent.

**Abstract TUAB0201–Table 1:** Multiplicative median change in APRI per 5 years

Analysis	APRI score at cohort entry, median (IQR)	PI users (APRI units/5 years), Exp(β) (95% CI)^a^	NNRTI users (APRI unit/5 years), Exp(β) (95% CI)^a^
1. Intention-to-treat	0.63 (0.39–1.30)	1.16 (1.03, 1.30)	1.05 (0.90, 1.20)
2. Per protocol	0.60 (0.39–1.22)	1.16 (1.00, 1.32)	1.07 (0.89, 1.24)
3. As treated	0.63 (0.39–1.30)	1.13 (0.99, 1.27)	1.09 (0.93, 1.25)

a Adjusted for baseline age, sex and time since HCV infection and updated alcohol use, CD4 cell count, viral load or virologic failure and number or type of previous regimens.

# Sub article: Ledipasvir/sofosbuvir for 12 weeks in patients co-infected with HCV and HIV-1

**DOI:** 10.7448/IAS.18.5.20362

**Published:** 2015-07-22

**Authors:** Susanna Naggie, Curtis Cooper, Michael Saag, Jenny C Yang, Luisa M Stamm, Philip S Pang, John G McHutchison, Douglas Dieterich, Mark S Sulkowski

**Affiliations:** 1 Infectious Diseases, Duke University Medical Center, Durham, United States; 2 Division of Infectious Diseases, University of Ottawa, Ottawa, Canada; 3 Department of Medicine, University of Alabama at Birmingham, Birmingham, United States; 4 Gilead Sciences, Inc., Foster City, United States; 5 Department of Medicine, Mount Sinai School of Medicine, New York, United States; 6 Infectious Disease Center for Viral Hepatitis, Johns Hopkins University School of Medicine, Baltimore, United States

## Abstract

**Introduction:**

Historically HIV co-infection was considered a negative predictor of HCV response to treatment with interferon/ribavirin (IFN/RBV). For sofosbuvir-based regimens, HIV/HCV patients have achieved similar sustained virologic response (SVR) rates as HCV mono-infected patients. We evaluated the safety and efficacy of the IFN-free, RBV-free, single tablet regimen of ledipasvir/sofosbuvir (LDV/SOF) in HCV genotype 1 or 4 patients co-infected with HIV-1 in the Phase 3 ION-4 study.

**Methods:**

HCV treatment naïve and experienced HIV co-infected patients on stable, approved antiretroviral (ARV) regimens were enrolled and received LDV/SOF (90 mg/400 mg) once daily for 12 weeks. Patients with compensated cirrhosis were eligible. Permitted concomitant ARVs included tenofovir and emtricitabine (TDF+FTC) with raltegravir (RAL), efavirenz (EFV) or rilpivirine (RPV). Safety evaluations included adverse event (AE) and standard laboratory parameter monitoring in addition to enhanced renal toxicity monitoring, CD4 count and HIV-1 RNA levels. The primary efficacy endpoint was SVR12.

**Results:**

A total of 335 patients with GT1a (75%), GT1b (23%) and GT4 (2%) were enrolled; 82% were male, 61% were white, mean age was 52 (range 26–72), mean baseline HCV RNA was 6.7 log_10_ IU/mL (range 4.1–7.8), median baseline CD4 count was 662 cells/µL (Q1, Q3=469, 823), 20% had cirrhosis, 24% were *IL28B* CC genotype and 55% had not responded to prior HCV treatment. Patients were taking EFV (48%) or RAL (44%) or RPV (9%). The table shows SVR12 by ARV regimen. Overall, the SVR12 rate was 96% (320/335); two patients had on-treatment virologic failure likely due to non-compliance and 10 had virologic relapse after discontinuing treatment. SVR12 was similar among non-cirrhotic (96%) and cirrhotic (94%) patients and also among treatment naïve (94%) and treatment experienced (97%) patients. No patient had confirmed HIV virologic rebound (HIV-1 RNA≥400 copies/mL). No patients discontinued study drug due to an AE. AEs occurring in ≥10% of patients were headache (25%), fatigue (21%) and diarrhoea (11%). No significant lab abnormalities were observed.

**Conclusions:**

The IFN-free, RBV-free, single tablet regimen of LDV/SOF administered once daily for 12 weeks is highly effective and well tolerated in treatment-naïve and experienced, genotype 1 or 4 HCV-infected patients with HIV-1 co-infection, including those with cirrhosis.

**Abstract TUAB0202–Table 1:** SVR12 by HIV regimen and overall

Virologic response	TDF+FTC+EFV (N=160)	TDF+FTC+RAL (N=146)	TDF+FTC+RPV (N=29)	Overall (N=335)
SVR12, n (%)	151 (94)	141 (97)	28 (97)	320 (96)
On-Treatment Failure, n (%)	1 (<1)	0	1 (3)	2 (<1)
Relapse, n (%)	8 (5)	2 (1)	0	10 (3)
Other, n (%)	0	3 (2)	0	3 (<1)

# Sub article: High SVR rates in HCV/HIV-1 co-infected patients regardless of baseline characteristics

**DOI:** 10.7448/IAS.18.5.20363

**Published:** 2015-07-22

**Authors:** David Wyles, Joseph J Eron, Jay Lalezari, Chia Wang, Peter J Ruane, Gary Blick, Laveeza Bhatti, Yiran B Hu, Melannie Co, Krystal Gibbons, Roger Trinh, Mark S Sulkowski

**Affiliations:** 1 Department of Medicine, University of California San Diego, San Diego, United States; 2 Department of Medicine, University of North Carolina, Chapel Hill, United States; 3 Quest Clinical Research, San Francisco, United States; 4 Virginia Mason Medical Center, Seattle, United States; 5 Ruane Medical and Liver Health Institute, Los Angeles, United States; 6 CIRCLE CARE Center, Norwalk, United States; 7 AIDS Healthcare Foundation, Beverly Hills, United States; 8 AbbVie Inc., Chicago, United States; 9 Infectious Disease Center for Viral Hepatitis, Johns Hopkins University, Baltimore, United States

## Abstract

**Introduction:**

The 3 direct-acting antiviral (3D) regimen of ombitasvir (OBV), paritaprevir (identified by AbbVie and Enanta; co-dosed with ritonavir; PTV/r) and dasabuvir (DSV) with ribavirin (RBV) is approved to treat HCV genotype 1 infection in patients with HIV-1 co-infection. In the TURQUOISE-I trial, response rates were 94 and 91% in this population when treated for 12 and 24 weeks, respectively. We report the week 12 post-treatment sustained virologic response rates (SVR12) by baseline characteristics.

**Methods:**

Patients were randomized to receive OBV/PTV/r + DSV + RBV for 12 (N=31) or 24 weeks (N=32). Eligible patients in this open-label study were treatment-naïve or pegIFN/RBV-experienced with or without cirrhosis, had CD4+ count ≥200 cells/mm^3^ or CD4+% ≥14%, and plasma HIV-1 RNA suppressed while receiving a stable atazanavir- or raltegravir-inclusive antiretroviral (ART) regimen.

**Results:**

Sixty-three patients were enrolled, of whom 92% were male, 24% black race, 19% with compensated cirrhosis and 16% with a prior null response to pegIFN/RBV treatment. Two patients in the 12-week treatment group (1 withdrawn consent, 1 HCV relapse), and three in the 24-week treatment group (1 on-treatment virologic breakthrough, 2 post-treatment HCV re-infections) did not achieve SVR12. The patients with on-treatment breakthrough and relapse were both genotype 1a-infected with prior null response to pegIFN/RBV and had F4 fibrosis (cirrhosis). High SVR12 rates were achieved in patients with historically difficult-to-cure characteristics including those with IL28B non-CC genotype, high viral load, prior treatment failure and advanced liver disease (Table 1). Lower baseline CD4+ T-cell counts did not negatively affect SVR12 rates. The regimen was well tolerated with no discontinuation due to adverse event or serious adverse event.

**Conclusions:**

In HCV genotype 1 patients co-infected with HIV-1, OBV/PTV/r + DSV + RBV achieved high rates of SVR12 regardless of baseline host, viral and disease characteristics whether treated with 12 or 24 weeks of therapy.

**Abstract TUAB0203–Table 1:** SVR12 rates by baseline characteristic, n/N (%)

Characteristic	12-week OBV/PTV/r + DSV + RBV	24-week OBV/PTV/r + DSV + RBV
Overall	29/31 (94)	29/32 (91)
Black race Hispanic or Latino ethnicity	7/7 (100) 7/8 (88)	7/8 (88) 7/8 (88)
Age, ≥55 years	7/8 (88)	12/12 (100)
BMI ≥30	3/3 (100)	7/7 (100)
IL28B genotype CT TT	16/16 (100) 8/10 (80)	19/20 (95) 4/5 (80)
Prior pegIFN/RBV treatment experience Naïve Relapser Partial response Null response	19/20 (95) 1/1 (100) 5/5 (100) 4/5 (80)	20/22 (91) 3/3 (100) 2/2 (100) 4/5 (80)
Baseline HCV RNA ≥800,000 IU/mL Baseline CD4+ T-cell cells/mm^3^ <350 350 – <500	25/27 (93) 2/2 (100) 8/8 (100)	26/28 (93) 5/5 (100) 7/8 (88)
Baseline CD4+ T-cells/mm^3^ <350 350 – <500 Baseline fibrosis stage F2 F3 F4	2/2 (100) 8/8 (100) 5/5 (100) 3/4 (75) 5/6 (83)	5/5 (100) 7/8 (88) 5/5 (100) 1/1 (100) 5/6 (83)

SVR12, sustained virologic response at post-treatment week 12; OBV, ombitasvir; PTV, paritaprevir; r, ritonavir; DSV, dasabuvir; RBV, ribavirin.

# Sub article: Liver fibrosis regression after anti HCV therapy and the rate of death, liver-related death, liver-related complications and hospital admissions in HIV/HCV co-infected patients with cirrhosis

**DOI:** 10.7448/IAS.18.5.20364

**Published:** 2015-07-22

**Authors:** Jose Luis Casado, Sara Bañon, Carmen Quereda, Ana Moreno, Maria J Perez Elías, Santiago Moreno

**Affiliations:** Infectious Diseases, Ramon y Cajal Hospital, Madrid, Spain

## Abstract

**Introduction:**

There are few data about the clinical outcome of hepatitis C (HCV)/HIV co-infected patients with liver cirrhosis after therapy, considering the possibility of fibrosis regression (FR).

**Methods:**

We compared the incidence rate (IR), and the time to develop a liver complication and death, in 139 cirrhotic patients according to sustained virological response (SVR) or/and FR, as established by a confirmed 1-point decrease in Metavir score by transient elastography (TE).

**Results:**

Overall, 42 patients reached SVR, and 23 of them (55%) had FR, in comparison with only 14 of the 91 (15%) without SVR. During a median follow up of 6.8 years (916.8 person-years), the IR of death, liver-related death, liver-related complications and hospital admissions were significantly lower in patients with SVR/FR (Table). SVR patients without FR had a worse IR of death (5.36) and liver-related death (2.68) than non-SVR patients with FR (1.3 and 0.65, respectively; p<0.01). In Cox multivariate analysis, only FR was associated with a lower risk of death (adjusted hazard ratio, HR, 0.36; 95% CI 0.15–0.86), and liver-related death (HR 0.15; 95% CI 0.03–0.65), whereas both FR (HR 0.09; 95% CI 0.03–0.3, p<0.01) and SVR (HR 0.24; 95% CI 0.07–0.87) decreased the risk of liver-related complications.

**Conclusions:**

FR is frequent after anti-HCV therapy in HIV/HCV co-infected patients with compensated cirrhosis who achieve SVR, and it is associated with the highest reduction of death of any cause, liver-related mortality, liver-related complications and hospital admissions.

**Table:** Abstract TUAB0204–Table 1

	SVR (42)			No SVR (91)		
		
	FR (23, 55%)	No FR (19, 45%)	p	FR (14, 15%)	No FR (77, 85%)	p
TE (Kpa)	7.1 (6.3–8.8)	17.5 (13.8–26.3)	<0.01	11.6 (6.3–11.2)	21.3 (17.2–45.4)	<0.01
Death (n, %) IR	4 (17%) 2.45	6 (32%) 5.36	0.01	2 (14%) 1.3	37 (48%) 7.6	<0.01
Liver-related death (n, %), IR	1 (4%) 0.61	3 (16%) 2.68	0.01	1 (7%) 3.65	29 (38%) 5.9	<0.01
Liver-related complications (n, %) IR	1 (4%) 1.22	2 (11%) 1.78	0.2 0.15	5 (36%) 3.25	33 (43%) 6.81	0.01<0.01
Hospital admissions (n, %) IR	2 (9%) 1.22	3 (16%) 2.68	0.7 0.13	4 (29%) 2.6	27 (30%) 5.6	0.2 0.04

# Sub article: How generalizable are direct antiviral agents (DAA) trials for real world people co-infected with HIV/hepatitis C?

**DOI:** 10.7448/IAS.18.5.20365

**Published:** 2015-07-22

**Authors:** Sahar Saeed, Kathleen Rollet-Kurhajec, Erin C Strumpf, Marina B Klein, The Canadian Co-Infection Cohort (CTN22)

**Affiliations:** 1 Department of Epidemiology, Biostatistics, and Occupational Health, McGill University, Montreal, Canada; 2 Department of Medicine, Division of Infectious Diseases/Chronic Viral Illness Service, Royal Victoria Hospital, McGill University Health Centre, Montreal, Canada; 3 CIHR Canadian HIV Trials Network, Vancouver, Canada

## Abstract

**Introduction:**

Worldwide, approximately seven million people are co-infected with HIV-Hepatitis C (HCV). The most common risk factor for co-infection is injection drug use. HCV treatments have evolved at an unprecedented speed; Simpeprevir (SIM) and Sofosburvir (SOF) are among the latest DAAs approved for use. However clinical trials conducted with these agents have enrolled a small number of individuals, in ideal circumstances with strict inclusion/exclusion criteria. This provokes the question: how generalizable are their results?

**Methods:**

We examined the study population characteristics (based on published inclusion/exclusion criteria) from the only two efficacy trials evaluating SIM (NCT01479868) and SOF (NCT01667731: PHOTON-1) for HIV-HCV co-infected patients and compared them to participants in the Canadian Co-Infection Cohort (CCC), a prospective cohort following 1383 co-infected people from across Canada (representing ~23% co-infected population in care).

**Results:**

Due to eligibility criteria, 30% (49/160) of screened subjects from 32 international study locations and 29% (96/330) of screened subjects from 27 American sites were excluded from the SIM and SOF trials, respectively. Of 1383 CCC participants, 1054 (76%) had evidence of chronic HCV (RNA+) at last visit; 699 (66%) infected with HCV genotype 1 and 887 (84%) infected with genotype 1, 2 or 3 and therefore could have been eligible for these trials. After applying all the available trial inclusion/exclusion criteria, only 8.6% of genotype 1 (60/699) and similarly 8.6% (76/887) overall would have been eligible to participate. Active drug use within 12 months accounted for 46% of reasons for non-eligibility, restriction to specific antiretroviral therapies and liver fibrosis staging were also highly exclusive as described in Table 1.

**Conclusions:**

Limited population level data makes it difficult to examine external validity of clinical trials. However using data from the CCC, we have illustrated that results obtained from clinical trials are not generalizable to the HIV-HCV patients in Canada and caution should be used when translating trial results in the real world.

**Abstract TUAB0205–Table 1:** Inclusion/exclusion criteria

Exclusion criteria (exclusive)	No (%) among genotype 1 (n=699)	No (%) among genotypes 1, 2 and 3 (n=887)
Specific cART regimens^a^	380 (54)	484 (55)
Active drug abuse within 12 months (excluding marijuana use)	320 (46)	402 (45)
HIV VL>50 copies/mL	175 (25)	225 (25)
HbA1c>10% (used HOMA IR>2 as surrogate)	171 (24)	217 (24)
APRI^b^ of <1 or ≥2	129 (18)	171 (19)
CD4 T-cell count <200 cells/mm^3^	106 (15)	136 (15)
Decompensated liver disease	23 (3)	27 (3)

a SOF trial: emtricitabine/tenofovir plus atazanavir/ritonavir; or darunavir/ritonavir; efavirenz; raltegravir; rilpivirine. SIM trial: excluded all boosted PIs and allowed only raltegravir, sustiva and ripilvirine;

b aspartate aminotransferase/platelet ratio index (APRI) <1 defined as non-cirrhotic or ≥2 defined as cirrhotic based on SOF trial.

# Sub article: High efficacy of grazoprevir/elbasvir in HCV genotype 1, 4 and 6-infected patients with HIV co-infection: the phase 3 C-EDGE co-infection study

**DOI:** 10.7448/IAS.18.5.20545

**Published:** 2015-07-22

**Authors:** Jürgen K Rockstroh, Mark Nelson, Christine Katlama, Jay Lalezari, Josep Mallolas, Mark Bloch, Gail Matthews, Michael S Saag, Philippe Zamor, Chloe Orkin, Jacqueline Gress, Melissa Shaughnessy, Stephanie Klopfer, Heather L Platt, Michael Robertson, Mark Sulkowski

**Affiliations:** 1 Bonn University Hospital, Bonn, Germany; 2 Chelsea and Westminster Hospital, London, United Kingdom; 3 Paris VI and Hôpital Pitié-Salpêtrière, Université Pierre et Marie Curie, Paris, France; 4 Quest Clinical Research, San Francisco, United States; 5 Hospital Clinic, University of Barcelona, Barcelona, Spain; 6 Holdsworth House Medical Practice, Darlinghurst, Australia; 7 St. Vincent's Hospital, Sydney, Australia; 8 University of Alabama at Birmingham, Birmingham, United States; 9 Carolinas Medical Center, Charlotte, United States; 10 Royal London Hospital, Bart's Health NHS Trust, London, United Kingdom; 11 Merck & Co. Inc., Kenilworth, United States; 12 School of Medicine, Johns Hopkins University, Baltimore, United States

## Abstract

**Introduction:**

The fixed-dose combination of grazoprevir (GZR, MK-5172, 100 mg, an NS3/4 protease inhibitor)/elbasvir (EBR, MK-8742, 50 mg, an NS5A inhibitor), an interferon-free, ribavirin-free, once-daily tablet has shown robust efficacy and safety in diverse populations. C-EDGE co-infection is an on-going phase-III study evaluating GZR/EBR among treatment-naïve, HIV/HCV co-infected patients with GT1, 4, or 6.

**Methods:**

Enrolled patients were on a stable antiretroviral (ARV) regimen (tenofovir or abacavir, and lamivudine or emtricitabine; and either raltegravir, dolutegravir or rilpivirine) with a CD4 >200 cells/mm^3^ and an HIV RNA <20 copies/mL, or were HIV treatment-naive with CD4 >500 cells/mm^3^ and VL <50,000 copies/mL. All patients received open-label GZR/EBR for 12 weeks. The primary efficacy endpoint was sustained virologic response at follow-up week 12 (SVR12). Adherence was assessed using electronic study medication diaries and pharmacokinetic (PK) assessment. All patients underwent testing for HCV resistance associated variants (RAVs) at baseline, and at failure and follow-up in those with virologic failure. Phylogenetic analysis was performed to distinguish relapse from reinfection.

**Results:**

A total of 218 patients were enrolled; 211 had suppressed HIV viraemia; 7 were ARV-naïve. In the Full Analysis Set population, SVR12 was achieved by 207/218 (95%) patients, including 35/35 (100%) patients with cirrhosis (Table 1). Of the 11 non-SVR12 patients, 4 failed for reasons other than virologic failure and 7 patients met criteria for virologic failure. Phylogenetic analysis of the seven failures demonstrated five were relapses and two were reinfections (Table 1). Thus, 5/218 (2.3%) patients failed to clear HCV infection that was present pre-therapy. Of the five virologic relapses, two had baseline NS5A RAVs with >5× resistance to EBR *in vitro* (L31M, Y93S). Adverse events (AEs) were reported in 157/218 (72%) patients; serious AEs occurred in 2/218 (0.9%) patients. Adherence was >90% in the total population, including virologic failures. There was no difference in PK parameters in patients who achieved SVR12 versus patients who did not achieve SVR12.

**Conclusions:**

A 12-week regimen of GZR/EBR FDC was highly effective among HIV/HCV co-infected patients with GT1, 4 or 6 infection, with a favourable safety profile. SVR was high across all patient subgroups including African-Americans and those with cirrhosis.

**Abstract TUAB0206LB–Table 1:** SVR12 by genotype

	All patients	GT1a	GT1b	GT4
	(N=218^a^)	(N=144)	(N=44)	(N=28)
**SVR12^b^**				
n/N	207/218	136/144	42/44	27/28
%	95.0%	94.4%	95.5%	96.4%
95% CI	91.2, 97.5	89.3, 97.6	84.5, 99.4	81.7. 99.9
				
**LTFU or unrelated to VF^c^**	4	3	1	0
Relapse^d^	5	4	0	1
Reinfection	2	1	1	0

a FAS (Full Analysis Set): all patients who received at least one dose of GZR/EBR.

b HCV RNA assessed via COBAS TaqMan v2.0 [lower limit of quantitation <15 IU/mL].

N=Number of subjects included in the analysis.

n (%)=Number of subjects who achieved SVR12 and the percentage calculated as (n/N)*100.

c Two subjects were lost to follow-up; one patient was discontinued for taking a prohibited concomitant medication, and one subject's FW12 visit was outside the analysis window.

d At baseline in the NSSA gene, one of the relapses had L31M/L RAV and one of the relapses had the Y93S RAV. The other three relapses had the WT NSSA gene at baseline.

# Sub article: Daclatasvir plus sofosbuvir with or without ribavirin in patients with HIV-HCV co-infection: interim analysis of a French multicentre compassionate use programme

**DOI:** 10.7448/IAS.18.5.20546

**Published:** 2015-07-22

**Authors:** Karine Lacombe, Helene Fontaine, Catherine Dhiver, Eric Rosenthal, Sophie Metivier, Teresa Antonini-Michelle, Marc Antoine Valantin, Patrick Miailhes, Stanislas Harent, Dominique Batisse, Georges-Philippe Pageaux, Hugues Aumaitre, Stephanie Dominguez, Julie Chas, Thierry Allegre, Alain Lafeuillade, Pierre De Truchis, Victor De Ledinghen, Vincent Leroy, Eric Billaud, Phillipe Sogni, Francois Dabis, Linda Wittkop, Claudine Duvivier, Anne Filipovics, Larysa Fedchuk, Yacia Bennai, Dominique Salmon

**Affiliations:** 1 AP-HP, Hôpital Saint-Antoine, INSERM UMR-S707, UPMC – Paris VI, Paris, France; 2 Cochin Hospital, Paris, France; 3 Conception Hospital, Marseille, France; 4 Archet Hospital, University Hospital of Nice, Nice, France; 5 Purpan University Hospital, Toulouse, France; 6 Paul-Brousse Hospital, Villejuif, France; 7 Pitié Salpêtrière Hospital, Paris, France; 8 Lyon Civil Hospices, Lyon, France; 9 Maladies Infectieuses, Parasitaires et Tropicales, Bichat, Paris, France; 10 Georges Pompidou European Hospital, Paris, France; 11 Saint-Eloi University Hospital, Montpellier, France; 12 Saint-Jean Hospital, Perpignan, France; 13 Henri Mondor Hospital, Créteil, France; 14 UPMC Tenon Hospital, Paris, France; 15 Pays d'Aix Hospital, Aix-en-Provence, France; 16 Sainte-Musse Hospital, Toulon, France; 17 R Poincare Hospital, Garches, France; 18 Haut-Lévêque Hospital, Pessac, France; 19 Hépato-gastroentérologie, CHU Grenoble, France; 20 Hôtel-Dieu University Hospital, Nantes, France; 21 INSERM U897, Bordeaux, France; 22 Necker Hospital, Paris, France; 23 Research and Development, Bristol-Myers Squibb, Rueil-Malmaison, France; 24 Department of Internal medicine and Infectious diseases, Saint Antoine Hospital, Paris, France

## Abstract

**Introduction:**

All-oral regimen with daclatasvir (DCV; NS5A replication complex inhibitor)+sofosbuvir (SOF; NS5B polymerase inhibitor)±weight-based ribavirin (RBV) has demonstrated high sustained virologic response (SVR) rates in HCV mono-infected patients. This analysis reports SVR4 and SVR12 results from an ongoing multicentre compassionate use programme (ATU) in France.

**Methods:**

HIV-HCV co-infected patients with advanced liver disease from 221 centres have been included since March 2014. All patients received DCV+SOF QD for 12 or 24 weeks, with RBV added at the physician's discretion. Baseline characteristics, virological response rates and adverse events were collected through a standardized form.

We report interim SVR rates at 4 and 12 weeks after the end of treatment for patients who have completed treatment to date.

**Results:**

Of 562 patients enrolled, 73.8% were males, median age was 52.3 years (30–74), 395 (71.0%) were cirrhotic and 460 (82.6%) were treatment-experienced. Child Pugh was A=85.4%, B=12.9%, C=1.7%. Genotype distribution was as follows: 387 GT1 (69.7%), 2 GT2 (0.4%), 72 GT3 (13.0%), 93 GT4 (16.8%) and 1 GT6 (0.1%), 7 missing data. Median HCV-RNA was 6.10 logUI/mL (1.08–7.97).

Combined antiretroviral therapy included: NRTI in 88%, PI in 36.4%, NNRTI in 23% and INI in 63.7% of the patients. Baseline median CD4 count was 551/mm^3^ (0–1922). HIV-RNA was undetectable in 505 patients (98.4%).

RBV was added to DCV+SOF in 67 patients (12.0%). Treatment duration was 24 weeks in 478 (85.1%) and 12 weeks in 84 (14.9%) patients.

Overall, SVR4 was obtained in 90.2% (148/164) and SVR12 in 95.9% (94/98) of the cases

Among patients treated with DCV+SOF for 12 or 24 weeks, 96.0% (24/25) and 95.1% (58/61) achieved an SVR12, respectively, compared to 100% (6/6) and 100% (6/6) for patients receiving DCV+SOF+RBV. Neither duration of treatment nor cirrhosis status and genotype influenced the rate of SVR12 (Table 1).

Treatment discontinuations occurred in 17 patients (3%) and were related to an adverse event (n=5), death (n=4, not related to treatment), patient decision (n=3), contraindication (n=3), unknown reason (n=1) and patient lost to follow-up (n=1).

**Conclusions:**

DCV+SOF±RBV regimen was well tolerated and demonstrated high SVR12 rate in HIV-HCV co-infected patients with advanced liver disease.

**Abstract TUAB0207LB–Table 1:** Efficacy of DCV+SOF±RBV regimens in HIV/HCV co-infection

Treatment duration			Genotype status					
	
	12 weeks	24 weeks	GT1 (all)	GT1 cirrhotic	GT3 (all)	GT3 cirrhotic	GT4 (all)	GT4 cirrhotic
SVR4, N=164	41/49 (83.7%)	107/115 (93.0%)	104/116 (89.7%)	80/87 (92.0%)	13/15 (86.7%)	12/13 (92.3%)	26/28 (92.9%)	16/17 (94.1%)
SVR12, N=98	30/31 (96.8%)	64/67 (95.5%)	66/68 (97.1%)	52/53 (98.1%)	11/11 (100%)	11/11 (100%)	14/15 (93.3%)	10/11 (90.9%)

# Sub article: Impact of conditional cash incentives on HSV-2 and HIV prevention in rural South African high school students: results of the CAPRISA 007 cluster randomized controlled trial

**DOI:** 10.7448/IAS.18.5.20547

**Published:** 2015-07-22

**Authors:** Quarraisha Abdool Karim, Kerry Leask, Ayesha Kharsany, Hilton Humphries, Fanele Ntombela, Natasha Samsunder, Cheryl Baxter, Janet Frohlich, Lynn van der Elst, Salim Abdool Karim

**Affiliations:** 1 CAPRISA, Durban, South Africa; 2 Department of Epidemiology, Columbia University, New York, United States; 3 MIET Africa, Durban, South Africa

## Abstract

**Introduction:**

Young women in southern Africa have high rates of sexually transmitted infections, including herpes simplex virus type-2 (HSV-2) and HIV. We investigated whether conditional cash incentives (CCIs) reduced the incidence of HSV-2 and HIV in rural high school students in South Africa.

**Methods:**

An open-label, matched-pair, cluster randomized controlled trial (CAPRISA 007) was undertaken in 3217 consenting male (n=1517) and female (n=1700) grade 9 and 10 students. A locally developed HIV prevention programme, *My Life! My Future!*, was actively implemented in all 14 schools. Seven schools (n=1592 students) were randomly assigned to receive; in addition, cash incentives (maximum of $175 over two years) for fulfilling any combination of four conditions; annual HIV testing, performance in school tests, participation in *My Life! My Future!*, and a written report on their community involvement project. HSV-2 and HIV serology was undertaken at baseline, 12 months and 24 months. In the intent-to-treat analysis, incidence rate ratios (IRRs) and p-values were adjusted for the matched-pair cluster design.

**Results:**

HSV-2 prevalence at baseline was 9.0% in CCI schools and 7.3% in control schools. During follow-up, there were 319 new HSV-2 infections, with an incidence rate of 6.2 per 100 person-years in CCI schools compared to 8.7 per 100 person-years in control schools (IRR=0.70, 95% CI: 0.57–0.86; p=0.007). HSV-2 incidence was 7.1 per 100 person-years in the 760 students who received <$65, 6.3 per 100 person-years in the 304 students who received $65–$95, and 4.2 per 100 person-years in the 265 students who received >$95 (Trend test, p=0.12). The lower-than-anticipated overall HIV incidence rate of 1.6 per 100 person-years was similar in both groups of schools (IRR=1.26, 95% CI: 0.66–2.39; p=0.419). A fourfold larger study would be required for 80% power to observe a 30% HIV incidence reduction.

**Conclusions:**

CCI schools had 30% lower HSV-2 incidence. Students who received larger cash incentives had lower HSV-2 incidence rates. The impact of CCI on HIV could not be adequately assessed as incidence was lower than expected, likely due to HIV lowering effects of both study-initiated and background community HIV interventions.

# Sub article: The effect of conditional economic compensation and lottery-based rewards on uptake of medical male circumcision in Kenya: a randomized trial

**DOI:** 10.7448/IAS.18.5.20366

**Published:** 2015-07-22

**Authors:** Harsha Thirumurthy, Samuel Masters, Samwel Rao, Katherine Murray, Eunice Omanga, Kawango Agot

**Affiliations:** 1 Department of Health Policy and Management, University of North Carolina at Chapel Hill, Chapel Hill, United States; 2 Carolina Population Center, University of North Carolina at Chapel Hill, Chapel Hill, United States; 3 Impact Research and Development Organization, Kisumu, Kenya

## Abstract

**Introduction:**

Low uptake of male circumcision has been a major challenge to scaling-up and maximizing the HIV prevention impact of voluntary medical male circumcision (VMMC) services in eastern and southern Africa. There is limited evidence on effective demand creation strategies for VMMC that address reported barriers to male circumcision. Building on insights from behavioural economics, we assessed whether providing compensation for opportunity costs of time or lottery-based rewards can increase VMMC uptake among men in Nyanza Province, Kenya.

**Methods:**

Uncircumcised men aged 21–39 years were provided information on VMMC services and randomized in 1:1:1 ratio to two intervention groups or a control group. One intervention group was offered compensation of US$12.50 conditional on VMMC uptake. Compensation was provided in the form of food vouchers valid at shops in the study region. A second intervention group was offered the opportunity to participate in a lottery with high-value prizes upon undergoing circumcision. The primary outcome was VMMC uptake within three months.

**Results:**

Among 903 participants enrolled, those randomized to receive compensation of US$12.50 had the highest VMMC uptake (8.4%, 26/308), followed by those receiving lottery-based rewards (3.3%, 10/302) and those in the control group (1.3%, 4/299). Logistic regression analysis showed that compared to the control group, the US$12.50 group had significantly higher VMMC uptake (Adjusted odds ratio (AOR) 7.1; 95% CI 2.4–20.8). Participants in the lottery-based rewards group were not significantly more likely to become circumcised than participants in the control group (AOR 2.5; 95% CI 0.8–8.1). The effect of providing compensation of US$12.50 was largest among participants who were contemplating circumcision at the time of enrolment.

**Conclusions:**

Providing conditional economic compensation was effective in increasing circumcision uptake among men in a short time period. The results are consistent with studies showing that small incentives can modify health behaviours by addressing barriers such as opportunity costs of time and present-biased decision-making. Contrary to findings from studies in high-income countries, lottery-based rewards did not significantly increase circumcision uptake. Testing economic interventions in other settings and applying them to different HIV behaviours can be useful for assessing the generalizability of the findings.

# Sub article: Estimating the population-level effect of homelessness on HIV viral suppression among people who use drugs: an observational study

**DOI:** 10.7448/IAS.18.5.20367

**Published:** 2015-07-22

**Authors:** Brandon D L Marshall, Beth Elston, Sabina Dobrer, Julio Montaner, Thomas Kerr, Evan Wood, M-J Milloy

**Affiliations:** 1 Department of Epidemiology, Brown University, Providence, United States; 2 British Columbia Centre for Excellence in HIV/AIDS, Vancouver, Canada; 3 Department of Medicine, University of British Columbia, Vancouver, Canada

## Abstract

**Introduction:**

Homelessness has been identified as an important structural barrier to effective antiretroviral therapy (ART) utilization among HIV-infected people who use drugs (PWUD). However, the potential effect of reducing homelessness on viral suppression rates at the community level is unknown. We used an imputation-based marginal modelling approach to estimate change in the prevalence of viral suppression among HIV-infected PWUD, if homelessness were eliminated from the population.

**Methods:**

We used data from a cohort study of community-recruited PWUD in Vancouver, Canada. Of note, HIV/AIDS treatment and care is provided free of charge in this setting. Persons were eligible to participate if they were HIV-infected and used an illicit drug in the month prior to enrolment. We assessed self-reported baseline housing status in the past six months. Viral suppression was defined as HIV RNA viral load <50 copies per mm^3^ at first study visit. We estimated the effect of homelessness on viral suppression using modified-Poisson regression, adjusting for demographics, socioeconomic characteristics, trauma history, depression, addiction treatment and other confounders. Then, a marginal modelling approach was applied. First, we imputed the outcome probability for each individual while manipulating the exposure (homelessness) to never exposed, and then averaged these probabilities across the population. Bootstrapping was conducted to calculate 95% confidence limits.

**Results:**

Of 718 eligible individuals enrolled between January 2005 and December 2013, the majority was male (66%), white race/ethnicity (55%) and had a history of injection drug use (94%). At baseline, 230 (32%) reported homelessness. The prevalence of viral suppression was 35% (95% CI: 31–38%). Adjusted marginal models estimated a 14% relative increase (95% CI: 10–24%) in viral suppression prevalence in the entire sample – to 40% (95% CI: 36–45%) – if all homeless individuals were housed. Among those homeless at baseline, adjusted marginal models estimated that eliminating this exposure would increase viral suppression from 19% (95% CI: 14–24%) to 37% (95% CI: 33–42%).

**Conclusions:**

Reducing homelessness among HIV-infected PWUD could have significant population-level benefits on outcomes in the HIV care continuum. Low threshold shelter and housing support programs should be considered as key components in comprehensive strategies to increase population-level viral suppression for PWUD.

# Sub article: Applying principles of behavioural economics to ART adherence: discount rate, future expectations and intrinsic motivation for adherence among ART initiates in Shinyanga region, Tanzania

**DOI:** 10.7448/IAS.18.5.20368

**Published:** 2015-07-22

**Authors:** Nancy Czaicki, Prosper Njau, Sergio Bautista-Arredondo, Otieno Simba, Nancy Padian, Sandra McCoy

**Affiliations:** 1 Epidemiology, University of California Berkeley, Berkeley, United States; 2 PMTCT, Ministry of Health and Social Welfare, Dar es Salaam, United Republic of Tanzania; 3 Department of Health Economics, National Institute of Public Health, Cuernavaca, Mexico; 4 Regional Medical Office, Shinyanga, United Republic of Tanzania; 5 Division of Epidemiology, School of Public Health, University of California Berkeley, Berkeley, United States

## Abstract

**Introduction:**

Behavioural economic theory suggests that understanding motivations and future preferences of people living with HIV infection (PLHIV) can inform the development of interventions supporting adherence to treatment and care. For example, PLHIV with high levels of intrinsic motivation to adhere to ART may require less external motivation, such as cash incentives. In addition, PLHIV who disproportionally value the present and heavily discount the future may be less likely to adhere to ART, a behaviour with future benefits and present costs. We measured these constructs among antiretroviral therapy (ART) initiates at four HIV care and treatment clinics in Shinyanga Region, Tanzania.

**Methods:**

We analyzed data collected from in-person interviews between December 2013 and December 2014 with food-insecure, HIV-infected adults who initiated ART in the past 90 days. Temporal discount rate, the rate at which individuals discount future costs and benefits, was measured using a bidding process to assess the acceptable percent increase of a hypothetical monetary offer they would receive in three months compared to a smaller amount received today. Future health expectations were assessed for one year from now, and intrinsic motivation for ART adherence was measured as the mean score (range: 0–3) on a Likert-scale using questions in the Treatment Self-Regulation Questionnaire.

**Results:**

Overall, 511 food-insecure recent ART initiates were interviewed (mean age: 37, 64% female). Nearly all (99%) expected their health to be somewhat (55%) or much better (44%) one year from now. Excluding those who initiated treatment on the same day of the interview, mean internal motivation was 2.75 (standard deviation 0.36; n=423). Temporal discount rates (n=489) fell into four ranges: <50% (8%), 50–100% (37%), 101–200% (54%) and >200% (2%).

**Conclusions:**

These data indicate high levels of both intrinsic motivation for ART adherence and optimism towards future health among food-insecure ART initiates in Tanzania, suggesting that interventions designed to strengthen and sustain intrinsic motivation may be appropriate. The high discount rates indicate a greater focus on the present; thus, interventions aiming to overcome the short-term cost barriers to adherence and care (e.g. time, transport and competing needs) in order to achieve future gains may be highly effective among this population.

# Sub article: Negative impact of South Africa's disability grants on HIV/AIDS recovery

**DOI:** 10.7448/IAS.18.5.20369

**Published:** 2015-07-22

**Authors:** Noah Haber, Frank Tanser, Kobus Herbst, Deenan Pillay, Till Bärnighausen, Wellcome Trust Africa Centre for Health and Population Studies

**Affiliations:** 1 Department of Global Health and Population, Harvard School of Public Health, Boston, United States; 2 Health Systems and Impact Evaluation, Wellcome Trust Africa Centre for Health and Population Studies, Mtubatuba, South Africa

## Abstract

**Introduction:**

The South African disability grant (DG) has been theorized to incentivize poor recovery by tying grant receipt to AIDS sickness. Prior to 2008, many official guidelines defined qualifying AIDS disability as a CD4 count below 200 mmHg, and this recommendation persists unofficially. We make two predictions:

1) The population distribution of CD4 counts will have an observable discontinuity with excess mass just below the CD4 qualification threshold of 200 mmHg, and

2) individuals receiving the grant will recover more slowly around this threshold than those who do not, due to threat of grant loss.

**Methods:**

The analysis utilizes a two-stage panel regression methodology to absorb individual trends and identify differential recovery rates around the CD4 threshold of 200 mmHg. The dataset for this analysis utilizes the Africa Centre Demographic Information System (ACDIS), an open cohort health and demographic monitoring programme consisting mainly of annual surveys, individually matched with an HIV-focused clinical informatics system in rural KwaZulu-Natal, South Africa. Data are restricted to HIV+ individuals from 2004 to 2011, who have at least four observed CD4 counts, with at least one observed CD4 count above and below 200 mmHg.

**Results:**

The cohort for this analysis consists of 11,160 observations from 1450 individuals. The distribution of CD4 counts shows clear excess mass just below a CD4 count of 200 mmHg, with more pronounced for CD4 counts occurring in 2008 or earlier. Among observations around the threshold, the rate of recovery of those receiving DGs is 0.23 mmHg/year lower (p=0.020) than that of those not receiving DGs, controlling for individual recovery trends, age, education, time, household assets and employment. Stratifying on gender, the effect is seen much stronger among women with a differential recovery rate of 58 mmHg/year (p=0.018). The effect is significantly larger for observations in 2008 or earlier.

**Conclusions:**

This study finds that the South African DG system resulted in a modest but significant manipulation of CD4 counts in order to qualify for the grant. While policy changes have likely reduced the severity of the effect, policy makers should ensure that incentives from grants are aligned with health incentives to reduce poor outcomes, infectivity and drug resistance.

# Sub article: HPTN 068 conditional cash transfer to prevent HIV infection among young women in South Africa: results of a randomized controlled trial

**DOI:** 10.7448/IAS.18.5.20548

**Published:** 2015-07-22

**Authors:** Audrey Pettifor, Catherine MacPhail, Amanda Selin, Xavier Gomez-Olivé, James Hughes, Ryan Wagner, Wonderful Mabuza, Immitrude Mokoena, Susan Eshleman, Estelle Piwowar-Manning, Rhian Twine, Aimée Julien, Cheryl Marcus, Philip Andrew, Jing Wang, Yi Xing, Laura McKinstry, Erica Hamilton, Yaw Agyei, Susannah Allison, Paul Sato, Ellen Townley, Stephen Tollman, Kathleen Kahn, HPTN 068 Study Team

**Affiliations:** 1 Department of Epidemiology, University of North Carolina at Chapel Hill, Chapel Hill, United States; 2 School of Public Health, University of the Witwatersrand, Johannesburg, South Africa; 3 University of New England, Armindale, Australia; 4 Carolina Population Center, University of North Carolina, Chapel Hill, United States; 5 MRC/Wits Rural Public Health and Health Transitions Unit, School of Public Health, University of the Witwatersrand, Johannesburg, South Africa; 6 University of Washington, Seattle, United States; 7 School of Medicine, Johns Hopkins University, Baltimore, United States; 8 School of Medicine, University of North Carolina, Chapel Hill, United States; 9 FHI360, Durham, United States; 10 Division of AIDS Research, National Institutes of Health, National Institutes of Mental Health, Rockville, United States; 11 Office of AIDS Research, National Institutes of Health, Bethesda, United States; 12 National Institutes of Allergies and Infectious Diseases, DAIDS, National Institutes of Health, Rockville, United States

## Abstract

**Introduction:**

Young women in South Africa face a particularly high risk of HIV infection. Structural factors such as schooling, socio-economic status (SES) and financial dependence on partners contribute to this risk. Cash transfers have shown promise in reducing HIV risk in young women by addressing these factors. HPTN 068 is the first randomized trial to examine the impact of conditional cash transfers on HIV incidence among young women.

**Methods:**

HPTN 068 is a phase III individually randomized trial to assess the impact of a conditional cash transfer on the acquisition of HIV among South Africa young women. Young women and their parent/guardian in the intervention arm received a monthly cash transfer conditional on 80% school attendance, which was verified using school attendance rosters. The intervention ran from April 2011 to March 2015. Participants enrolled in the study were aged 13–20, in high school, not married or pregnant and resident in the Agincourt Health and Demographic Surveillance System (AHDSS) site in rural Mpumalanga Province. Participants were seen at baseline, then annually for up to three follow-up visits, where HIV and HSV-2 testing was conducted and an interview was completed using Audio Computer-Assisted Self Interviewing (ACASI). The interview assessed sexual behaviour including partner-specific details, schooling, mental health, SES and gender power dynamics. Participants were tested for HIV infection using two HIV rapid tests with Western blot confirmation. Stored samples from all participants at all visits were also tested at the HPTN Laboratory Center using assays that included an HIV antigen/antibody test and a qualitative HIV RNA test. To compare treatment arms, time to first HIV detection was analysed using a Cox proportional hazards model.

**Results:**

We will present the impact of the conditional cash transfer on HIV incidence, unprotected sex, pregnancy, age difference with partners, number of sex partners, transactional sex, age of sexual debut and school attendance.

**Conclusions:**

Cash transfers are increasingly being included as part of the package of prevention services that should be offered to young women to reduce HIV risk in sub-Saharan Africa. The evidence from this RCT will have important implications for HIV prevention policy and practice.

# Sub article: The safety of HIV pre-exposure prophylaxis in the presence of hepatitis B infection

**DOI:** 10.7448/IAS.18.5.20370

**Published:** 2015-07-22

**Authors:** Marc M Solomon, Mauro Schechter, Albert Y Liu, Vanessa McMahan, Juan V Guanira, Robert J Hance, Suwat Chariyalertsak, Kenneth H Mayer, Robert M Grant, iPrEx Study Team

**Affiliations:** 1 University of California, San Francisco, Division of Infectious Diseases, San Francisco, United States; 2 Gladstone Institutes of Virology and Immunology, San Francisco, United States; 3 Projeto Praça Onze, Hospital Escola São Francisco de Assis, Universidade Federal do Rio de Janeiro, Rio de Janeiro, Brazil; 4 Bridge HIV, San Francisco Department of Public Health, San Francisco, United States; 5 Investigaciones Medicas en Salud, Lima, Peru; 6 Research Institute for Health Sciences and Department of Community Medicine, Faculty of Medicine, Chiang Mai University, Chiang Mai, Thailand; 7 Fenway Institute, Fenway Health, Beth Israel Deaconess Medical Center, Harvard Medical School, Boston, United States; 8 University of California, San Francisco, San Francisco, United States

## Abstract

**Introduction:**

Pre-exposure prophylaxis (PrEP) with daily oral FTC/TDF prevents HIV infection and is safe, but concern has been raised that PrEP could cause hepatitis B virus (HBV)-associated flares when discontinued by people with HBV infection, particularly among individuals with cirrhosis. The safety and feasibility of providing HIV PrEP in the setting of HBV infection was evaluated in the iPrEx study.

**Methods:**

The iPrEx study randomized 2499 HIV-negative men and transgender women who have sex with men to once-daily oral FTC/TDF versus placebo. Hepatitis serologies and transaminases were obtained at screening and at PrEP discontinuation. Participants with a reactive hepatitis B surface antigen were enrolled if there was no clinical evidence of cirrhosis and transaminases were <2.5-fold the ULN. HBV DNA was assessed by PCR and drug resistance was assessed by population sequencing (Abbott labs) at least once for individuals with evidence of HBV DNA. Vaccination was offered to individuals susceptible to HBV.

**Results:**

Among 2499 enrolled participants, 12 (0.5%; including six randomized to FTC/TDF) had chronic HBV infection. After stopping study drug, five of six in the active arm had LFTs performed at follow-up. LFTs remained within normal limits at post-stop visits except for a Grade 1 elevation in one participant at post-stop week 12 (ALT=90, AST=61). There was no evidence of flares. PCR of stored samples showed that four had evidence of acute HBV infection at enrolment (two in the active arm). Both had evidence of grade 4 transaminase elevations by week 4 with subsequent resolution. Overall, there was no evidence of TDF or FTC resistance among tested genotypes. Of 1633 eligible for vaccination, 1587 (97.2%) received at least one vaccine and 1383 (84.7%) received the complete series. Anti-HBs detection was 44.4% after one, 74.5% after two and 86.9% after three doses.

**Conclusions:**

PrEP can be safely offered to persons with HBV infection if there is no evidence of cirrhosis or substantial transaminase elevation. As information is limited and treatment for HBV is complex, referral to a specialist is appropriate when available. HBV vaccination rates at screening were low globally, yet uptake and efficacy were high when offered.

# Sub article: Adherence, sexual behaviour and HIV/STI incidence among men who have sex with men and transgender women in the US PrEP demonstration (Demo) project

**DOI:** 10.7448/IAS.18.5.20371

**Published:** 2015-07-22

**Authors:** Albert Liu, Stephanie Cohen, Eric Vittinghoff, Peter Anderson, Susanne Doblecki-Lewis, Oliver Bacon, Wairimu Chege, Richard Elion, Susan Buchbinder, Michael Kolber, Demo Project Study Group

**Affiliations:** 1 San Francisco Department of Public Health, San Francisco, United States; 2 Department of Epidemiology & Biostatistics, University of California, San Francisco, United States; 3 Department of Pharmaceutical Sciences, University of Colorado, Aurora, United States; 4 University of Miami, Miami, United States; 5 National Institutes of Health, Bethesda, United States; 6 Whitman-Walker Health, Washington, DC, United States

## Abstract

**Introduction:**

Pre-exposure prophylaxis (PrEP) has demonstrated efficacy in reducing HIV acquisition in men who have sex with men (MSM) and transgender women (TGW). Little is known about adherence, sexual behaviour and HIV/STI incidence among those who elect to take PrEP in real-world settings.

**Methods:**

The Demo Project is the first US multi-site open-label study assessing PrEP delivery in municipal STD (San Francisco, Miami) and community-health (Washington, DC) clinics. HIV-uninfected MSM/TGW were offered 48 weeks of PrEP. Tenofovir-diphosphate levels were measured in dried blood spots (DBS) in a random sample of participants (pts). Correlates of adherence were assessed using multivariable logistic regression. Sexual behaviours, PrEP discontinuations and HIV/STI incidence are described.

**Results:**

From 9/2012 to 1/2014, 557 pts enrolled, with 83% retained for the final visit (468.8 person-years (py)). Longitudinal drug levels, sexual behaviour and STI incidence are shown (Figure). Among 147 pts with DBS testing, 65% had drug levels consistent with taking ≥4 doses/week at all visits, 3% always had DBS levels <2 doses/week, and 32% had an inconsistent pattern. Black pts, being self-referred to the PrEP programme and having a greater number of condomless anal sex (AS) partners were independently associated with DBS ≥4 doses/week (all p<0.05). Median AS partners in the past three months declined from baseline to week 48 (5 to 4, p<0.0008). Two-thirds reported condomless receptive AS (CRAS) at baseline, which remained stable during follow-up (p=0.96). Twenty pts chose to stop PrEP due to low self-perceived HIV risk, however 65% of these pts reported CRAS in the prior three to six months. Three participants were acutely infected at enrolment, and one seroconverted during follow-up (HIV incidence 0.21/100 py). This subject had DBS <2 doses/week at all prior visits. Overall, 27.5% had early syphilis, GC or CT at screening, and 38% had ≥1 STI during follow-up; STI incidence was high (47.9, 42.8 and 12.6/100 py for CT, GC and syphilis) but did not increase over time (p=0.87).

**Conclusions:**

PrEP adherence was high and HIV incidence was low in this cohort at ongoing high sexual risk for HIV. STIs were common during PrEP use, highlighting the importance of screening and treatment. Strategies for counselling on appropriate PrEP discontinuation are warranted.

**Abstract TUAC0202–Figure 1:** Adherence, Risk Behavior and STI incidence Over Time in the Demo Project.

# Sub article: Characteristics and oral PrEP adherence in the TDF2 open-label extension in Botswana

**DOI:** 10.7448/IAS.18.5.20372

**Published:** 2015-07-22

**Authors:** Faith Henderson, Allan Taylor, Lovemore Chirwa, Tiffany Williams, Craig Borkowf, Michael Kasonde, Rodreck Mutanhaurwa, Onkabetse Matlhaba, Kathy Hageman, Paula Casillas

**Affiliations:** 1 Centers for Disease Control and Prevention, Atlanta, United States; 2 Centers for Disease Control and Prevention, Francistown, Botswana; 3 ICF Macro, Atlanta, United States; 4 Centers for Disease Control and Prevention, Gaborone, Botswana

## Abstract

**Introduction:**

The TDF2 Study was a randomized, double-blind, placebo-controlled trial of daily oral co-formulated tenofovir disoproxil fumarate (300 mg)/emtricitabine (200 mg) (TDF/FTC) for pre-exposure prophylaxis of HIV infection (PrEP) among young heterosexual adults in Gaborone and Francistown, Botswana. TDF2 completed follow-up in 2011, demonstrating 62% overall protective efficacy. We describe final results of a 12-month open-label extension (OLE).

**Methods:**

Between February and May 2013, former TDF2 participants were screened and offered 30-day supplies of TDF/FTC for up to 12 months. OLE exclusion criteria included HIV infection, pregnancy/breastfeeding and abnormal serum creatinine clearance or phosphorus. Demographic and sexual behaviour data were collected at baseline. Dual rapid fingerstick HIV testing, sexual behaviour questionnaires and self-reported adherence measures were conducted monthly. Dried blood spots (DBS) were collected monthly. Tenofovir levels were measured from DBS for a subset of 30 randomly selected participants at months 1, 3, 6, 9 and 12.

**Results:**

Of 1219 TDF2 participants, 736 were contacted, and 229 (Male: 55.5%) were eligible and started drug. 71.2% were single, and 23.9% were married/cohabitating. 60.3% of participants completed at least 10 monthly visits. Across all visits, 71.2% reported one sex partner in the prior 30 days; 8.7% reported two partners, and 2.4% reported ≥3 partners. For the prior three days, 87.8% reported taking TDF/FTC daily, while 5.5% reported taking it 1–2 times and 6.7% reported taking none. Overall, 58.3% reported “very good” adherence in the prior 30 days, and 32.3% reported “good” adherence. Of the 30 participants (Male: 77%) selected for DBS testing, the overall proportion with detectable mean tenofovir levels (>25 ng/mL) was 94%. At months 1, 3, 6, 9 and 12, the proportion with detectable mean tenofovir levels were 93, 93, 100, 93 and 90%, respectively. After starting drug, no HIV infections were observed during the study.

**Conclusions:**

In this open-label study of TDF/FTC for oral PrEP, we observed high self-reported three-day medication adherence, high percentage of detectable DBS tenofovir levels and no HIV infections. These findings lend support to efforts to expand availability of PrEP in the context of generalized epidemics in resource-limited settings. Further work is needed to define longer-term adherence for such populations.

# Sub article: An HIV pre-exposure prophylaxis demonstration project and safety study for young men who have sex with men in the United States (ATN 110)

**DOI:** 10.7448/IAS.18.5.20549

**Published:** 2015-07-22

**Authors:** Sybil Hosek, Bret Rudy, Raphael Landovitz, Bill Kapogiannis, George Siberry, Nancy Liu, Brandy Rutledge, Jennifer Brothers, Jim Rooney, Craig M Wilson, Adolescent Medicine Trials Network for HIV/AIDS Interventions (AT)

**Affiliations:** 1 Psychiatry, Stroger Hospital of Cook County, Chicago, United States; 2 New York University Medical Center, New York, United States; 3 University of California Los Angeles, Los Angeles, United States; 4 NICHD/MPIDB, Bethesda, United States; 5 Westat, Rockville, United States; 6 Gilead Sciences, Foster City, United States; 7 University of Alabama at Birmingham, Birmingham, United States

## Abstract

**Introduction:**

Young men who have sex with men (YMSM), particularly racial/ethnic minority YMSM, are a key population for implementation of domestic pre-exposure prophylaxis (PrEP) interventions. This open-label PrEP study examined uptake and adherence to PrEP and assessed sexual risk behaviour among a diverse sample of YMSM in 12 U.S. cities.

**Methods:**

ATN110 combined PrEP with evidence-based behavioural risk reduction interventions along with frequent sexual health and adherence promotion counselling. Eligible participants were 18- to 22-year-old HIV-uninfected MSM who reported HIV transmission risk behaviour in the past six months. Participants were recruited and screened for preliminary eligibility through venue-based outreach, community presentations and online advertising. Laboratory screening determined final eligibility. Study visits occurred at baseline, monthly through week 12, then quarterly through week 48. Dried blood spots were serially collected for the quantification of tenofovir diphosphate (TFV-DP) blood levels.

**Results:**

Between March and September 2013, 2186 individuals were approached, 277 (13%) were preliminarily eligible and 200 were enrolled (mean age=20.2; 54.5% Black, 26.5% Latino). Eleven (4%) had undiagnosed HIV infection at screening and two acute HIV infections were diagnosed at baseline. Diagnosis of STIs at baseline was high (22%) and remained high across visits. Most participants (98%) chose to take PrEP. Figure 1 shows TFV-DP levels. At week 4, 56% of participants had TFV-DP levels consistent with ≥4 pills/week. By week 48, 34% of participants had TFV-DP levels consistent with ≥4 pills/week, with a noticeable drop-off occurring at Week 24. Four HIV seroconversions occurred on study (3.29/100 person-years); all had TFV-DP BLQ at diagnosis. Condomless sex was reported by >80% of participants throughout the study and condomless anal sex with last partner was associated with higher TFV-DP levels.

**Conclusions:**

ATN110 enrolled a diverse sample of YMSM vulnerable to HIV. PrEP uptake was high with the majority achieving protective drug levels during initial monthly visits. As visits decreased in frequency, so did adherence, while reported sexual risk behaviour remained constant. Given the frequency of STI diagnoses, HIV infections may have been higher without PrEP. YMSM in the U.S. may need access to PrEP in youth-friendly settings with tailored adherence support and potentially augmented visit schedules.

**Abstract TUAC0204LB–Figure 1:** Tenofovir diphosphate levels (fmol/punch) and PrEP dosing estimates as measured by dried blood spot assay.

# Sub article: Pre-exposure prophylaxis uptake and associated factors among MSM and TGW in the PrEP Brasil demonstration project

**DOI:** 10.7448/IAS.18.5.20550

**Published:** 2015-07-22

**Authors:** Brenda Hoagland, Valdilea G Veloso, Raquel B De Boni, José Valdez Madruga, Esper G Kallas, Nilo Martinez Fernandes, Ronaldo I Moreira, Albert Y Liu, Beatriz Grinsztejn, PrEP Brasil Study Team

**Affiliations:** 1 IPEC, Fundação Oswaldo Cruz, Rio de Janeiro, Brazil; 2 Fiocruz, Rio de Janeiro, Brazil; 3 CRT, São Paulo, Brazil; 4 USP, São Paulo, Brazil; 5 Bridge HIV, San Francisco Department of Public Health, San Francisco, United States

## Abstract

**Introduction:**

In Brazil, men who have sex with men (MSM) and transgender women (TGW) are the populations most heavily affected by the AIDS epidemic. Although the WHO recommends pre-exposure prophylaxis (PrEP) for these populations, the feasibility and interest in this prevention strategy in real-world settings in low- and middle-income countries are unknown. This study aims to describe PrEP uptake and associated factors in Brazil.

**Methods:**

PrEP Brasil is a demonstration project to assess the feasibility of implementing PrEP provided at no cost to high risk MSM and TGW within the Brazilian public health system. The project was advertised through social and other media. Participants were assessed for PrEP eligibility at FIOCRUZ-RJ, CRT-SP and USP-SP. At USP, 100% participants were self-referred, while at FIOCRUZ and CRT, they were either self-referred or assessed for participation during HIV-testing or post-exposure prophylaxis provision. Predictors of PrEP uptake were assessed using a Poisson regression model.

**Results:**

Of 986 MSM/TGW approached between April/2014 and April/2015, 798 were potentially eligible and 409 were enrolled. PrEP uptake was 51.25%. Median age at enrolment was 29 years (IQR 25–35); 93.5% had≥12 years of education; 83.9%, 8.8% and 5.9% identified themselves as homosexual, bisexual or TGW, respectively (Table); syphilis prevalence, rectal *Chlamydia* and *Gonorrhoea* detection were 21.3%, 8.2% and 4.7%, respectively. In multivariate analysis, factors associated with PrEP uptake were: recruitment at CRT-SP (aRR 1.27; 95% CI 0.99–1.62) or USP-SP (aRR 1.72; 95% CI 1.33–2.24) versus FIOCRUZ; having a steady partner (aRR 1.45, 95% CI 1.18–1.78); having an HIV-test within the last 12 months (aRR 1.33, 95% CI 1.01–1.74); prior PrEP awareness (aRR 1.27, 95% CI 1.0–1.59) and having≥2 male condomless anal sex partners within the last 12 months (aRR 1.65, 95% CI 1.32–2.05).

**Conclusions:**

This is the first PrEP demonstration project for MSM and TGW in a middle-income country. Overall, PrEP uptake was high. The higher uptake among those at higher risk and with an existing awareness of PrEP emphasizes the importance of establishing strategies to improve HIV risk perception and PrEP awareness in the MSM and TGW communities in Brazil.

**Abstract TUAC0205LB–Table 1:** Study population characteristics and PrEP uptake

	Approached (1) N (%)	Potentially Eligible (2) N (%)	Included (3) N (%)	Declined (4) N (%)	Percent of PrEP uptake*	p-value**
Overall	986	798	409	365	51.25	
Site location (5)						<0.001
FIOCRUZ	622 (63.08)	455 (57.02)	175 (42.79)	282 (77.26)	38.46	
CRT-SP	225 (22.82)	216 (27.07)	135 (33.01)	57 (15.62)	62.5	
USP-SP	139 (14.1)	127 (15.91)	99 (24.21)	26 (7.12)	77.95	
Age years						0.26
18–25	335 (33.98)	266 (33.33)	127 (31.05)	128 (35.07)	47.74	
26–35	435 (44.12)	358 (44.86)	189 (46.21)	165 (45.21)	52.79	
36–45	160 (16.23)	124 (15.54)	62 (15.16)	57 (15.62)	50	
>45	56 (5.68)	50 (6.27)	31 (7.58)	15 (4.11)	62	
Sexual Identity						0.04
Homosexual	823 (83.55)	658 (82.56)	343 (83.86)	293 (80.49)	52.13	
Bisexual	99 (10.05)	87 (10.92)	36 (8.8)	47 (12.91)	41.38	
Tranagender woman	44 (4.47)	36 (4.52)	24 (5.87)	14 (3.85)	66.67	
Other	19 (1.93)	16 (2.01)	6 (1.47)	10 (2.75)	37.5	
Color/Race						0.05
White	455 (46.15)	399 (50)	219 (53.55)	161 (44.11)	54.89	
Non-white	531 (53.85)	399 (50)	190 (46.45)	204 (55.89)	47.62	
Schoolong years						0.06
<12	89 (9.03)	66 (8.27)	26 (6.36)	42 (11.51)	39.39	
>12	897 (90.97)	732 (91.73)	383 (93.64)	323 (88.49)	52.32	
Steady partner						<0.001
Yes	472 (47.87)	385 (48.25)	223 (54.52)	149 (40.82)	57.92	
No	514 (52.13)	413 (51.75)	186 (45.48)	216 (59.18)	45.04	
Perceived likelihood of getting HIV in the next year						<0.001
0–25%	569 (57.71)	437 (54.76)	189 (46.21)	237 (64.93)	43.25	
50–100%	417 (42.29)	361 (45.24)	220 (53.79)	128 (35.07)	60.94	
Previous HIV test (last 12 months)						<0.001
Yes	657 (66.63)	575 (72.06)	334 (81.66)	219 (60)	58.09	
No	329 (33.37)	223 (27.94)	75 (18.34)	146 (40)	33.63	
Prior PrEP awareness						<0.001
Yes	594 (60.43)	498 (62.64)	296 (72.55)	183 (50.41)	59.44	
No	389 (39.57)	297 (37.36)	112 (27.45)	180 (49.59)	37.71	
# Male condomless anal sex partners (last 12 months)						<0.001
<2	512 (51.93)	370 (46.37)	143 (34.96)	222 (60.82)	38.65	
2 or more	474 (48.07)	428 (53.63)	266 (65.04)	143 (39.18)	62.15	
Anal sex with HIV-positive partners (12 months)						<0.001
Yes	346 (35.09)	324 (40.6)	208 (50.86)	104 (28.49)	64.2	
No	211 (21.4)	87 (10.9)	41 (10.02)	44 (12.05)	47.13	
I do not know	429 (43.51)	387 (48.5)	160 (39.12)	217 (59.45)	41.34	
STD diagnosis (12 months)						0.01
Yes	138 (14)	128 (16.04)	79 (19.32)	39 (10.68)	61.72	
No	848 (86)	670 (83.96)	330 (80.68)	326 (89.32)	49.25	

(1) All individuals approached for pre-screening who were age 18 or older, male at birth, lived in the State, self-reported HIV negative status and reported having at least one male sexual partner in last 12 months.

(2) Includes all individuals approached at pre-screening (1) who: a) reported 2 or more male condomless anal sex partners OR anal sex with HIV positive partner OR STD diagnosis in last 12 months; and b) had a negative HIV test results.

(3) Individuals who enrolled the study.

(4) Decline represents the sum of refusals in all steps. Indivuduals who agreed to participate but did not show up at the screen or enrollment visit were considered as declining.

(5) FIOCRUZ-RJ: Fundação Oswaldo Cruz, located in Rio de Janelro; CRT-SP: Centro de Referencla e Treinamento em DST e AIDS, located in São Paulo; USP-SP: Universidade de São Paulo.

* % uptake- # Included/# Potentially elligible at pre-screening.

** chi-square for bivariate analyses.

# Sub article: Pharmacokinetics and pharmacodynamics of tenofovir reduced-glycerin 1% gel in the rectal and vaginal compartments in women: a cross-compartmental study with directly observed dosing

**DOI:** 10.7448/IAS.18.5.20551

**Published:** 2015-07-22

**Authors:** Gonasagrie Nair, Jessica E Justman, Jeanna Piper, Mark Marzinke, Craig Hendrix, James Dai, Jason Pan, Beth Galaska, Lisa Levy, Jill Shwartz, Ian McGowan, Charlene Dezutti

**Affiliations:** 1 Centre for AIDS Programme of Research in South Africa (CAPRISA), University of KwaZulu Natal, Durban, South Africa; 2 ICAP at Columbia University, Columbia University, New York, United States; 3 NIAID/DAIDS, Bethesda, United States; 4 Johns Hopkins University, Baltimore, United States; 5 FHCRC-SCHARP, Seattle, United States; 6 Microbicides Trial Network, Pittsburgh, United States; 7 FHI 360, Washington, United States; 8 CONRAD, Arlington, United States; 9 Magee-Womens Research Institute, University of Pittsburgh, Pittsburgh, United States

## Abstract

**Introduction:**

Tenofovir (TFV) gel, when used consistently as a vaginal microbicide, prevents HIV infection. As unprotected anal intercourse is prevalent amongst heterosexual women, data on TFV concentrations and anti-HIV activity in the rectal compartment following vaginal application, and vice versa, are needed.

**Methods:**

MTN-014 is a phase 1 cross-over, randomized trial comparing the pharmacokinetics of TFV reduced-glycerin (RG) 1% gel following 14 days each of daily rectal versus vaginal directly observed dosing (DOD), with a six-week washout period in between each phase. Vaginal and rectal tissue and fluid and blood samples were collected 24 hours after the end of each phase and analyzed for TFV and TFV-diphosphate (TFV-DP) concentrations. Vaginal and rectal fluids were tested for HIV inhibition using a TZM-bl assay.

**Results:**

Fourteen HIV-uninfected women, mean age 34 years, were enrolled at the Bronx Prevention Center in New York City and 13 completed all study procedures. Of the 392 expected doses, 91% were DOD, two (0.5%) were missed and the remaining doses were reported as used. Mean plasma TFV concentrations were similar after 14 days of either dosing route (Table). Rectal concentrations of TFV and TFV-DP were detectable after vaginal dosing in only 1 of 13 and 2 of 13 tissue samples, respectively, while vaginal concentrations of TFV and TFV-DP were detectable after rectal dosing in 6 of 14 and 3 of 14 samples, respectively. Rectal and vaginal dosing phases each resulted in markedly lower levels of tissue TFV and TFV-DP concentrations in the opposite compartment, with at least 1.7 log 10 differences between mean concentrations in the two compartments.

After vaginal dosing, inhibition of HIV increased by 42% in vaginal fluid, but no change was found in rectal fluid. No change in HIV inhibition in vaginal or rectal fluid was noted after rectal dosing.

**Conclusions:**

Cross-compartmental concentrations of TFV and TFV-DP were low in this study comparing rectal and vaginal DOD TFV RG 1% gel, and pharmacodynamics activity was noted only in the vaginal fluid compartment. Whether these low tissue concentrations are protective remains to be determined.

**Abstract TUAC0206LB–Table 1:** Compartmental pharmacokinetics of tenofovir gel

	Vaginal use phase			Rectal use phase		
		
Compartment	Mean (standard deviation)	number of samples with detectable drug	Median, IOR	Mean (standard deviation)	number of samples with detectable drug	Median, IQR
Plasma						
TFV (ng/mL)	0.99 (1.27)	10/14 (71%)	0.58 (0, 1.31)	1.19 (1.74)	10/14 (71%)	0.82 (0, 1.22)
Vaginal tissue						
TVF (ng/mg)	45.8 (72.6)	12/13 (92%)	8.5 (1.0, 44.8)	0.09 (0.12)	6/14 (43%)	0 (0, 0.16)
TFV-DP (fmol/mg)	1945 (4105)	12/13 (92%)	166 (37, 2377)	13 (30)	3/14 (21%)	0 (0, 0)
Rectal tissue						
TFV (ng/mL)	0.02 (0.06)	1/13 (8%)	0 (0, 0)	12.2 (27.1)	12/14 (86%)	3.0 (0.7, 10.9)
TFV-DP (fmol/mg)	10.48 (25.81)	2/13 (15%)	0 (0,0)	710 (1306)	10/14 (71%)	196 (0, 550)

# Sub article: Worsen epidemic of early HIV infection among men who have sex with men in China: implication for real time action

**DOI:** 10.7448/IAS.18.5.20373

**Published:** 2015-07-22

**Authors:** Junjie Xu, Weiming Tang, Huachun Zou, Tanmay Mahapatra, Qinghai Hu, Gengfeng Fu, Zhe Wang, Lin Lu, Minghua Zhuang, Xi Chen, Jihua Fu, Yanqiu Yu, Jinxin Lu, Yongjun Jiang, Wenqing Geng, Xiaoxu Han, Hong Shang

**Affiliations:** 1 The First affiliated hospital of China Medical University, Shengyang, China; 2 Project-China, University of North Carolina, Guangzhou, China; 3 Department of Epidemiology, University of California at Los Angeles, Los Angeles, United States; 4 Jiangsu Provincial Center for Disease Control and Prevention, Nanjing, China; 5 Henan Provincial Center for Disease Prevention and Control, Zhengzhou, China; 6 Yunnan Provincial Center for Disease Prevention and Control, Kunming, China; 7 Shanghai Center for Disease Prevention and Control, Shanghai, China; 8 Hunan Provincial Center for Disease Prevention and Control, Changsha, China; 9 Shangdong Provincial Center for Disease Prevention and Control, JInan, China

## Abstract

**Introduction:**

Recent upsurge of new HIV infections among men who have sex with men (MSM) is a major concern in China. Paucity of national-level information regarding the burden and predictors of this progressive epidemic of new infections called for a multi-centric, comprehensive investigation.

**Methods:**

Mixed methods were used to recruit MSM (engaged in sex with men (oral and/or anal) within the last one year, aged 18 years or older and agreed to provide written informed consent) from seven cities (Shanghai, Nanjing, Changsha, Zhengzhou, Ji'nan, Shenyang and Kunming) in different regions of China between 2012 and 2013. Early and established HIV infections were determined by Western Blot and BED HIV-1 capture enzyme immunoassay. Syphilis and herpes simplex virus-2 (HSV-2) were also tested. The study process and content were approved (No. 2011(36)) by the Ethics Committee of The First Affiliated Hospital of China Medical University.

**Results:**

A total of 4496 eligible MSM were recruited. The majority was aged ≤35 years (77.5%), migrants (60.3%), never married (69.8%) and played receptive role in anal sex (70.5%). The HIV prevalence was 9·9% and 41·9% were recently infected, with HIV incidence of 8·9/100 person-years. The prevalence of HSV-2 and syphilis were 12·5 and 8·5%, respectively. Early HIV infection was associated with having multiple male partners (aOR=1.4, 95% CI 1.1–1.9), recreational drug use (aOR=2.2, 95% CI 1.6–3.0), anal bleeding (aOR=2.1, 95% CI 1.4–3.0), circumcision experience (aOR=2.0, 95% CI.1·3–3.1), syphilis infection (aOR=2·8, 95% CI 1.9–4.3) and HSV-2 infection (aOR=2.3, 95% CI 1.5–3.3).

**Conclusions:**

HIV epidemic among Chinese MSM was worsening with an alarming number of recently infected HIV patients along with high burden of STIs. High rate of early HIV infection is potentially resulting in progressive deterioration of the overall HIV epidemic among MSM in China. Interventions specifically targeting high-risk MSM especially those having high-risk behaviours (especially multiple partners and recreational drug use), syphilis or HSV-2 infection and anal bleeding were urgently required for efficient control of HIV among MSM in China.

# Sub article: Repeat HIV voluntary counselling and testing within one year among men who have sex with men, Bangkok, Thailand 2006–2013

**DOI:** 10.7448/IAS.18.5.20374

**Published:** 2015-07-22

**Authors:** Wipas Wimonsate, Sarika Pattanasin, Anuwat Sriporn, Pikunchai Luechai, Kesinee Satumay, Narongritt Tippanonth, Nutthawoot Promda, Timothy Holtz, Anupong Chitwarakorn, Eileen Dunne

**Affiliations:** 1 Thailand MOPH – U.S. CDC Collaboration, HIV/STD Research Program, Nonthaburi, Thailand; 2 Centers for Disease Control and Prevention, Division of HIV/AIDS Prevention, Atlanta, United States; 3 Ministry of Public Health, Department of Disease Control and Prevention, Nonthaburi, Thailand

## Abstract

**Introduction:**

Current Thailand Ministry of Public Health (MOPH) recommendations state that men who have sex with men (MSM) should repeat HIV testing every 6–12 months. We investigated the proportion and trend of repeat HIV voluntary counselling and testing (VCT) within 12 months among Thai MSM attending Silom Community Clinic @TropMed.

**Methods:**

Silom Community Clinic @Trop Med has been located in downtown Bangkok since late 2005, with easy access and convenient operating hours for MSM. It provides free-of-charge, confidential and rapid HIV VCT by MSM-friendly staff. We advertize the clinic via website, Facebook, outreach and friend referrals. For first-time testers, we recommend that they repeat VCT every 6–12 months. For this analysis, we included men with an initial HIV test who had visited the clinic for ≥12 months and had a baseline HIV-negative result; the first VCT visit occurred between 2006 and 2013 with follow-up period through October 2014. On a yearly basis, we looked at the number and proportion of first-time testers who had another VCT visit within the next 12 months. We used chi-square test for trend to test changes in the proportion of repeat testing within 12 months by calendar year.

**Results:**

Between 2006 and 2013, 9345 MSM were tested by our testing services and 4597 met the criteria above and were included in this analysis. Most (67.1%) were 25 years and older and most (87.9%) lived in Bangkok or nearby provinces at time of first test. Among these MSM, 2016 (43.9%) repeated VCT. The number of new testers increased annually from 340 men in 2006 to 880 in 2013. The proportion of MSM who repeated VCT within one year varied between 15.3 and 26.1% by calendar year (mean=22.2%) and there was a statistically significant increasing trend from 2006 to 2013 (p<0.01) (Figure 1).

**Conclusions:**

Between 2006 and 2013, the number of new testers doubled, and the proportion of men who repeated VCT significantly increased. Given that roughly one-fifth of MSM repeated VCT within 12 months, counselling to emphasize repeating VCT according to Thailand MOPH recommendations should be strengthened and systematic strategies to retain testers should be implemented.

**Abstract TUAC0302–Figure 1:** Proportion of HIV repeat testers.

# Sub article: Dramatic declines in lifetime HIV risk and persistence of racial disparities among men who have sex with men in King County, Washington, USA

**DOI:** 10.7448/IAS.18.5.20375

**Published:** 2015-07-22

**Authors:** Galant Chan, Amy Bennett, Susan Buskin, Julia Dombrowski, Matthew Golden

**Affiliations:** 1 Department of Medicine, University of Washington, Seattle, United States; 2 Public Health- Seattle & King County, Seattle, United States; 3 Department of Epidemiology, University of Washington, Seattle, United States

## Abstract

**Introduction:**

In the United States, HIV disproportionately affects men who have sex with men (MSM), who account for >60% of new cases. Although recent data suggest HIV incidence is declining nationally, rates in MSM are stable, and the proportion of cases occurring in black MSM is increasing. Because sexual mixing is largely age-assortative, using life tables to estimate risk within birth cohorts may be useful in assessing and anticipating trends in the population's risk.

**Methods:**

We constructed life tables for the period 1982–2012 to estimate the cumulative risk of HIV diagnosis among MSM born 1940–1994 in King County. We used U.S. Census data to define the size of the white and black male populations of King County, Washington, national and local survey data to estimate the proportion of men who are MSM, and local surveillance data to define the number of HIV diagnoses in MSM each year.

**Results:**

We estimated that 6% of the local male population was MSM. Age-specific risk of HIV diagnosis increased in birth cohorts from the 1940s until the mid-1960s and thereafter declined, plateauing among cohorts born after the mid-1970s (Figure 1).

This trend occurred in both white and black MSM. In the peak risk cohort, among MSM born 1960–64, >40% of white and >60% of black MSM had been diagnosed with HIV by age 50. A dramatic decline in this risk was evident when comparing the percentage of MSM diagnosed with HIV in different birth cohorts. Among white and black MSM born 1960–1964, the cumulative risk of HIV diagnosis by age 35 was 29 and 42%, respectively, while among MSM born 1975–1979, this risk decreased to 9 and 15%, respectively. However, as absolute risk of HIV diagnosis decreased overall in younger cohorts, relative differences between white and black MSM appeared to increase. Throughout the period and across birth cohorts, cumulative HIV risk was 18 to 84% higher among black versus white MSM.

**Conclusions:**

Comparing birth cohorts, cumulative HIV risk among MSM in King County has declined approximately 65% in those born after the mid-1960s, although racial disparities persist. Our findings highlight the importance of evaluating HIV risk within birth cohorts and demonstrate remarkable local progress in HIV prevention.

**Abstract TUAC0303–Figure 1:** Cumulative risk of acquiring HIV among MSM.

# Sub article: Ethical considerations for inclusion of men who have sex with men under the age of 18 in epidemiological research: evidence from six sub-Saharan African countries

**DOI:** 10.7448/IAS.18.5.20376

**Published:** 2015-07-22

**Authors:** Ashley Grosso, Shauna Stahlman, Tampose Mothopeng, Noah Taruberekera, Gautier Ouedraogo, Odette Ky-Zerbo, Vincent Pitche, Simplice Anato, Bhekie Sithole, Xolile Mabuza, Nuha Ceesay, Daouda Diouf, Gift Trapence, Eric Umar, Stefan Baral

**Affiliations:** 1 Center for Public Health and Human Rights, Johns Hopkins University, Baltimore, United States; 2 Matrix Support Group, Maseru, Lesotho; 3 Population Services International, Johannesburg, South Africa; 4 Institute de Recherche en Sciences de la Sante, Ouagadougou, Burkina Faso; 5 Programme d'Appui au Monde Associatif et Communautaire, Ouagadougou, Burkina Faso; 6 Conseil National de Lutte contre le SIDA du Togo, Lomé, Togo; 7 Arc en Ciel, Lomé, Togo; 8 Department of Health Sciences, University of Stellenbosch, Stellenbosch, South Africa; 9 Rock of Hope, Manzini, Swaziland; 10 UNAIDS, Banjul, Gambia; 11 Enda Sante, Dakar, Senegal; 12 Center for the Development of People, Blantyre, Malawi; 13 Malawi College of Medicine, Blantyre, Malawi

## Abstract

**Introduction:**

In many settings, laws or institutional review board policies require parental permission for youth <18 years to participate in research. Individual and social risk factors for HIV acquisition often occur before age 18. Youth may be unwilling to participate in HIV epidemiological research requiring parental consent due to the sensitive nature of risk factors such as sexual behaviours and experiences of violence. Young men who have sex with men (MSM) are at especially high risk for HIV acquisition and are often unwilling or unable to disclose their sexual orientation or practices to their parents. In sub-Saharan Africa, where HIV prevalence among MSM is high and sex between men is criminalized or highly stigmatized in many countries, epidemiologic research on this vulnerable population of young MSM is particularly relevant and sparse. One strategy for assessing the potential size of the population of young (<18) MSM is to ask adult MSM retrospective questions about the age at which they first had anal sex with a man.

**Methods:**

MSM aged 18 or older were recruited using respondent-driven sampling in Burkina Faso, Togo, Lesotho, Malawi and Swaziland. MSM aged 15 and above were recruited using snowball sampling in The Gambia. Participants completed a survey that included a question asking how old they were when they first had anal sex with another man. This variable was dichotomized and tabulated to assess the prevalence of anal sex under the age of 18.

**Results:**

Across settings, 40.20% (1106/2751) of MSM had anal sex with a man before the age of 18. The highest percentage was in Lome, Togo (63.84%), while the smallest percentage was in Swaziland (14.46%). MSM under the age of 18 represented 12.14% of the study sample in The Gambia.

**Conclusions:**

A substantial proportion of MSM participants had anal sex with a man under the age of 18. Further research on this group, including a waiver of requirements for parental consent for participation, is warranted. Given the relatively small proportion of study participants under the age of 18 in a setting where this was feasible, additional outreach strategies such as web-based recruitment may be necessary.

**Abstract TUAC0304–Table 1:** Proportion of MSM sampled who had anal sex <18 yr

Country	Percentage (n/N) of MSM study participants who first had anal sex with a man when they were under the age of 18	Percentage of study participants under 18 years old
Bobo-Dioulasso, Burkina Faso	51.21% (169/330)	N/A
Ouagadougou, Burkina Faso	51.31% (176/343)	N/A
Kara, Togo	41.95% (138/329)	N/A
Lome, Togo	63.84% (226/354)	N/A
Gambia	43.69% (90/206)	12.14% (25/206)
Maputsoe, Lesotho	40.95% (129/315)	N/A
Maseru, Lesotho	35.85% (76/212)	N/A
Malawi	16.32% (55/337)	N/A
Swaziland	14.46% (47/325)	N/A

# Sub article: Nuanced seroadaptive behaviours among Seattle men who have sex with men: sexual decision-making based on ART use/viral load and recency of partner HIV testing

**DOI:** 10.7448/IAS.18.5.20377

**Published:** 2015-07-22

**Authors:** Christine M Khosropour, David A Katz, Julia C Dombrowski, Matthew R Golden

**Affiliations:** 1 Department of Epidemiology, University of Washington, Seattle, United States; 2 Department of Medicine, University of Washington, Seattle, United States; 3 HIV/STD Program, Public Health – Seattle & King County, Seattle, United States

## Abstract

**Introduction:**

Seroadaptive behaviours among men who have sex with men (MSM) may protect against HIV. Anecdotally, some MSM incorporate partners’ antiretroviral therapy (ART)/viral load (VL) or HIV testing frequency into sexual decision-making. The frequency and effect of these strategies is unknown.

**Methods:**

HIV-negative MSM attending an STD clinic in Seattle, WA from March–December 2014 were enrolled in a study of seroadaptive behaviours. Men completed a computer-based survey on behaviours in the past 12 months. HIV testing was performed per clinic protocol. Among HIV-negative men with HIV-negative partners, we examined if the timing of the partner's last HIV test was associated with condomless anal intercourse (CAI). Of those with HIV-positive partners, we asked (in aggregate) if respondents’ decision to have sex or use condoms was based on partner ART use or VL (i.e. ART/VL serosorting). We compared proportions with chi-square tests.

**Results:**

We enrolled 988 (58%) of 1718 eligible HIV-negative MSM. The mean age was 33 and 62% were white, non-Hispanic. Most (69%) had CAI with HIV-negative partners, 18% had CAI with HIV-positive partners and 22% reported no CAI. The majority (86%) asked HIV-negative partners when the partner last tested negative. CAI was more common among men whose most recent partner tested ≤3 months ago compared to men whose partner tested >3 months ago or the partner did not know when he last tested (48% vs. 40%, p=0.02). Of 222 men with HIV-positive partners, 60 and 64% decided whether to have sex/use condoms based on their partners’ ART use or VL, respectively. CAI with an HIV-positive partner was more common among men who reported ART/VL serosorting compared to those who did not (79% vs. 57%, p=0.03), but testing newly positive for HIV was less common among men who reported ART/VL serosorting compared to men who did not (1/120 (1%) vs. 2/23 (9%)).

**Conclusions:**

Among Seattle MSM, nuanced seroadapative behaviours such as ART/VL serosorting and using the recency of a partner's HIV test to inform sexual decision-making are common. The high prevalence of these behaviours suggests they could impact HIV incidence rates, but the individual- and population-level effects of these behaviours are uncertain.

# Sub article: Viral load awareness and risk behaviour in male serodiscordant couples in Australia, Brazil and Thailand

**DOI:** 10.7448/IAS.18.5.20378

**Published:** 2015-07-22

**Authors:** Benjamin Robert Bavinton, Fengyi Jin, Garrett Prestage, Iryna Zablotska, Beatriz Grinsztejn, Nittaya Phanuphak, Ruth Khalili Friedman, Andrew Edwin Grulich, Opposites Attract Study Group

**Affiliations:** 1 The Kirby Institute, University of New South Wales, Kensington, Australia; 2 Australian Research Centre in Sex, Health and Society, La Trobe University, Melbourne, Australia; 3 Instituto de Pesquisa Clínica Evandro Chagas, Fundação Oswaldo Cruz, Rio de Janeiro, Brazil; 4 Thai Red Cross AIDS Research Centre, Bangkok, Thailand

## Abstract

**Introduction:**

There are very limited data from homosexual male serodiscordant couples (HM-SDC) on the impact of antiretroviral therapy (ART) and viral load (VL) on HIV transmission risk and on risk behaviours within such couples. To date, no studies have investigated the issue in middle income countries.

**Methods:**

*Opposites Attract* is an ongoing multisite cohort study of HM-SDC in Australia, Brazil and Thailand. HIV-positive partners (HPP) had VL tested; HIV-negative partners (HNP) had HIV antibody tests and reported sexual behaviour and perception of the HPP's most recent VL test. Undetectable VL (UVL) was defined as <200 copies/mL. We compared couples from the three countries; baseline differences were examined with bivariate logistic regression.

**Results:**

By January 2015, 242 couples were enrolled (Australia=137, Brazil=53, Thailand=52). The majority of HPP were taking ART (80.2%); this was lower in Thailand than in Australia and Brazil (p<0.001), accompanied by higher proportions with UVL in Australia (88.2%) and Brazil (85.0%) than in Thailand (69.2%, p=0.008). Overall, 61.2% of HNP perceived their HPP's last VL test result to be undetectable. Brazilian and Thai HNP were more likely not to know the result (17.0 and 38.5%) compared to Australians (5.1%, p<0.001). Australian HNP reported more sex with other partners than Brazilian (p=0.013) but not Thai HNP (p=0.183). Australian HNP reported more condomless anal intercourse (CLAI) with outside partners compared to both Brazilians (p=0.002) and Thais (p=0.012). 54.6% of HNP reported CLAI with study partner in the last three months. Compared to Australia (67.9%), this was lower in Brazil (45.3%, p=0.005) and Thailand (28.9%, p< 0.001). Overall, 63.5% of HNP who perceived the HPP's VL to be undetectable reported CLAI in the last three months, compared to only 40.4% of HNP in which the HPP's VL was perceived to be detectable/unknown (OR=0.39, 95% CI=0.23–0.66, p=0.001). While this was strongly associated amongst Australian couples (p=0.002), there was no such association in Brazil or Thailand.

**Conclusions:**

Australian HNP were more aware of their partner's VL results. Australian HM-SDC with perceived UVL practiced more CLAI, suggesting they may be acting upon beliefs that treatment-as-prevention is effective. This pattern was not seen in Brazil and Thailand.

**Abstract TUAC0306–Table 1:** Baseline characteristics of HIV-positive and HIV-negative

	Total (n=242)	Australia (n=137)	Brazil (n=53)	Thailand (n=52)
HPP: taking ART	194 (80.2)	124 (90.5)	45 (84.9)	25 (48.1)
HPP: viral load <200 copies/mL (available for 227 HPP)	189 (83.3)	119 (88.2)	34 (85.0)	36 (69.2)
HPP: Adherence to ART >90% (of those taking ART)	170 (91.9)	107 (92.2)	40 (88.9)	23 (95.8)
HNP: perceived VL of HPP				
Undetectable VL	148 (61.2)	107 (78.1)	34 (64.2)	7 (13.5)
Detectable VL	58 (24.0)	23 (16.8)	10 (18.9)	25 (48.1)
Don't know VL	36 (14.9)	7 (5.1)	9 (17.0)	20 (38.5)
HNP: any CLAI with outside partners, last three months	45 (18.6)	38 (27.7)	2 (3.8)	5 (9.6)
HNP: any CLAI with study partner, last three months	132 (54.6)	93 (67.9)	24 (45.3)	15 (28.9)

# Sub article: Estimating the number of people who inject drugs in two urban areas in Mozambique using four different methods, 2014

**DOI:** 10.7448/IAS.18.5.20379

**Published:** 2015-07-22

**Authors:** Isabel Sathane, Roberta Horth, Makini Boothe, Cynthia Sema Baltazar, Peter Young, Henry Fisher, Eugenia Teodoro, Maria Lídia Gouveia, Tim Lane, Willi Mcfarland

**Affiliations:** 1 MARPS, I-TECH Mozambique, Maputo, Mozambique; 2 Department of Public Health (SFDPH), Surveillance Prevention & Public Health Group, University of California, San Francisco, United States; 3 National Institute of Health, Ministry of Health, Maputo, Mozambique; 4 Centers for Disease Control and Prevention, Maputo, Mozambique; 5 Department of Mental Health, Ministry of Health, Maputo, Mozambique

## Abstract

**Introduction:**

There are few[VR1] data on HIV prevalence and the number of people who inject drugs (PWID) in Mozambique. As part of the Integrated Biological and Behavioral Surveillance (IBBS) Survey implemented in 2014, we conducted the first population size estimation among PWID in two urban areas, Maputo (n=353) and Nampula (n=139).

**Methods:**

Given the lack of a gold standard, we synthesized four independent methods to estimate the number of PWID: unique object multiplier, wisdom of the crowd, sequential sampling and literature review. The unique object estimate is calculated as the number of objects distributed to PWID pre-survey, divided by the proportion of survey participants who reported receiving the objects. The wisdom of the crowd method polls the participants on how many people they believe inject drugs in each city (responses equal to the personal network size were excluded). The sequential sampling method applies a Bayesian approach to the self-reported PWID network size of each participant to infer the size of the hidden population. In the literature review, estimates were based on proportions of adults who are PWID from other African locations applied to the 2014 census projections for Maputo and Nampula. A consensus meeting among stakeholders agreed that the median of all four methods was the best estimate of population size of PWID in each city and also agreed to the lowest and highest estimates as “acceptable bounds.”

**Results:**

HIV prevalence was 50.3% (95% confidence interval (CI): 40.7–58.9) and 36.8% (CI: 24.3–49.3) in Maputo and Nampula, respectively. The numbers of PWID were estimated at 1445 (0.19% of adults) (acceptable bounds: 1281 (0.17%) to 3524 (0.46%)) and 465 (0.14%) (acceptable bounds: 354 (0.10%) to 3921 (1.16%)). Using these population size estimates, there are 727 and 171 PWID infected with HIV and in need of care and/or treatment services in Maputo and Nampula, respectively.

**Conclusions:**

Our results highlight the feasibility of using the median of multiple methods to estimate the size of PWID in two urban areas in Mozambique. Given the limited population size and high rates of infection, harm-reduction, prevention interventions and HIV care and treatment services should be practical and affordable in this population.

**Abstract TUAC0401–Figure 1:** (a) Population size estimation using four independent methods, Maputio City. (b) Population size estimation using four independent methods, Nampula.

# Sub article: Efficacy of a network intervention in reducing HIV incidence among people who inject drugs in Ukraine: preliminary results from a clustered randomized trial

**DOI:** 10.7448/IAS.18.5.20380

**Published:** 2015-07-22

**Authors:** Robert Booth, Jonathan Davis, Sergiy Dvoryak, John Brewster, Steffanie Strathdee, Carl Latkin

**Affiliations:** 1 Department of Psychiatry, University of Colorado Denver, Denver, United States; 2 Ukrainian Institute on Public Policy, Kiev, Ukraine; 3 University of California San Diego, San Diego, United States; 4 Johns Hopkins University, Baltimore, United States

## Abstract

**Introduction:**

HIV incidence among people who inject drugs (PWID) in Ukraine is among the highest in the world. We assessed the efficacy of two interventions, a network-based peer intervention combined with HIV testing and counselling (T/C combined; experimental condition, N=614) versus HIV testing and counselling alone (T/C alone; control condition, N=592), in reducing HIV incidence among PWID.

**Methods:**

Between 2010 and 2014, 1205 HIV-seronegative PWID were recruited from street settings in Odessa, Donetsk and Nikolayev. We used a clustered randomized design that consisted of 611 networks and included: peer-leaders; first wave network members; and second wave network members. Participants were randomly assigned to interventions in groups of 16 and interviewed at baseline, 6 and 12 months. Interviewers and HIV tester/counsellors were not blinded to intervention. Cox regression was used to compare HIV incidence between groups, incorporating GEE to account for clustering.

**Results:**

Preliminary results suggest that mean age and duration of injection was 31.8 and 11.7 years, respectively; 75% were male. In the past 30 days, 43% injected daily, 46% always injected with others, 78% had ≥1 sex partner. HIV incidence was 19.0 per 100 person-years (py) in the experimental condition compared to 31.8 per 100 py in the control condition (p<0.001). PWID in the experimental condition had a 39% reduced hazard for HIV seroconversion versus the control group (p<0.001). With each year increase in age, the hazard increased by 5% (p<0.001), and with each injection episode in the past 30 days, the hazard increased by 0.6% (p=0.02). Those who were sexually active in the last 30 days had a 26% reduced hazard (p=0.03).

**Conclusions:**

The combined network-based peer intervention and was more efficacious in reducing HIV incidence among PWID in Ukraine than T/C alone.

# Sub article: Factors associated with initiation of antiretroviral therapy among HIV-infected people who use illicit drugs

**DOI:** 10.7448/IAS.18.5.20381

**Published:** 2015-07-22

**Authors:** Brenden Joseph, M. J Milloy

**Affiliations:** 1 British Columbia Centre for Excellence in HIV/AIDS, Vancouver, Canada; 2 Faculty of Medicine, University of British Columbia, Vancouver, Canada

## Abstract

**Introduction:**

Treatment-as-prevention-based efforts to reduce HIV/AIDS-associated morbidity, mortality and HIV viral transmission among people who use illicit drugs (PWUD) rely on prompt engagement in antiretroviral therapy (ART). However, the longitudinal factors that promote or block initiation of ART among PWUD are not well described. Thus, we sought to identify factors associated with time from seroconversion to ART initiation among PWUD.

**Methods:**

Using data from two observational prospective cohorts of illicit drug users linked to comprehensive ART dispensation records, we included HIV-seronegative individuals at baseline who seroconverted during follow-up. We fit multivariable Cox proportional hazards models adjusted for a time-updated measure of clinical eligibility for ART to identify factors independently associated with time to treatment initiation following seroconversion.

**Results:**

We included 133 individuals of whom 98 (73.7%) initiated ART during follow-up at a rate of 17.6 per 100 person-years. In a multivariable model adjusted for clinical eligibility, living in the HIV epicentre (adjusted hazard ratio (AHR)=1.62, 95% confidence interval (95% CI)=1.01–2.58), methadone maintenance therapy (MMT) (AHR=2.37, 95% CI=1.56–3.60) and a later year of interview (AHR=1.07, 95% CI=1.02–1.13) were associated with shorter time to ART initiation. Barriers to ART initiation were illicit income generation (AHR=0.51, 95% CI=0.32–0.79) and incarceration (AHR=0.52, 95% CI=0.28–0.97).

**Conclusions:**

In this sample of community-recruited HIV-positive PWUD with well-defined dates of seroconversion, we found that illicit income generation and incarceration were barriers to ART initiation while MMT and living in the HIV epicentre promoted ART initiation independent of clinical eligibility. Current efforts to scale-up HIV treatment among PWUD should consider these factors in order to reduce HIV/AIDS-associated morbidity, mortality and HIV viral transmission.

# Sub article: Periodic HIV testing and immediate antiretroviral therapy among people who inject drugs in Vietnam

**DOI:** 10.7448/IAS.18.5.20382

**Published:** 2015-07-22

**Authors:** Huu Hai Nguyen, Duc Duong Bui, Thi Thanh Thuy Dinh, Minh Sang Nguyen, Hong Tram Tran, Le Hai Nguyen, Thi Thuy Van Nguyen, Quoc Dat Vu, Thi Nhan Do, Ai Kim Anh Le, Ba Can Nguyen, Amitabh Suthar, Ying-Ru Lo, Hong Thang Pham, Minh Giang Le, Hoang Long Nguyen, Masaya Kato

**Affiliations:** 1 Viet Nam Authority for HIV/AIDS Control, Hanoi, Vietnam; 2 Center for Research and Training on HIV/AIDS, Ha Noi Medical University, Hanoi, Vietnam; 3 National Institute of Hygiene and Epidemiology, Hanoi, Vietnam; 4 World Health Organization, Hanoi, Vietnam; 5 Thai Nguyen Province AIDS Center, Thai Nguyen, Vietnam; 6 Thanh Hoa Province AIDS Center, Hanoi, Vietnam

## Abstract

**Introduction:**

In Vietnam, injecting drug use is[VR1] the leading cause of HIV transmission. Multiple local transmission models suggest periodic HIV testing and counselling (HTC) and initiating antiretroviral therapy (ART) irrespective of CD4 count in people who inject drugs (PWID) can markedly reduce HIV-related mortality and transmission. Programme experience with this approach in Vietnam is limited. Therefore, the acceptability and feasibility of this approach was assessed in two high-burden provinces. We present preliminary ART outcomes.

**Methods:**

Village health workers, PWID peer educators, and health staff were educated on the new approach and the benefits and risks of immediate ART initiation. Since April 2014, HTC has been recommended to PWID every six months, and immediate ART, that is, initiation irrespective of CD4 count, has been offered to PWID living with HIV in Thai Nguyen and Thanh Hoa provinces. Following consent, PWID were followed for 12 months. HIV viral load (VL) was assessed before ART start (baseline) and at months 6 and 12.

**Results:**

Of 232 identified HIV-positive PWID, 218 (94%) agreed to participate and initiate immediate ART, among which 102 initiated ART before 30 June 2014. Of this cohort, 97.1% were males, median age was 36 years, 47.1% reported methadone use in the past three months, 38.2% had baseline CD4 counts greater than 350 cells/mm^3^ and median baseline VL was 4.1 (IQR 2.3–5.2) log10 copies/mL. Ninety-one of the 102 participants (89.2%) were retained after six months (eight died and three lost-to-follow-up). Retention was 84.1 and 97.4% among PWID with baseline CD4 counts below and above 350 cells/mm^3^, respectively (Figure 1). Excluding five patients who transferred to other care sites and seven patients whose samples were not available due to logistical issues, 67 of the 79 participants (84.8%) achieved viral suppression (i.e. VL<1000 copies/mL) at month six. Viral suppression was 84.4 and 85.3% among PWID with CD4 counts below and above 350 cells/mm^3^, respectively (Figure 1).

**Conclusions:**

The preliminary results suggest high uptake and adherence to ART irrespective of CD4 count among PWID; however, late presentation to care remains a critical problem. The results are informing the revision of the national guidelines to include immediate ART in key populations.

**Abstract TUAC0404–Figure 1:** Retention on ART and viral suppression among PWID enrolled in the study disaggregated by CD4 at ART initiation.

# Sub article: Social and socio-economic benefits of antiretroviral therapy adherence among HIV-infected people who use illicit drugs in Vancouver, Canada

**DOI:** 10.7448/IAS.18.5.20383

**Published:** 2015-07-22

**Authors:** Lindsey Richardson, Thomas Kerr, Bob Hogg, Silvia Guillemi, Julio Montaner, Evan Wood, M-J Milloy

**Affiliations:** 1 B.C. Centre for Excellence in HIV/AIDS, Vancouver, Canada; 2 Department of Sociology, University of British Columbia, Vancouver, Canada; 3 Division of AIDS, University of British Columbia, Faculty of Medicine, Vancouver, Canada; 4 Faculty of Health Sciences, Simon Fraser University, Burnaby, Canada

## Abstract

**Introduction:**

There is extensive documentation of the direct clinical benefits of antiretroviral therapy (ART) adherence leading to plasma HIV RNA-1 viral load suppression. However, very little is known about the social, socio-economic and ancillary clinical benefits of ART adherence, particularly among people who use illicit drugs (PWUD).

**Methods:**

We used longitudinal data from a prospective cohort of community-recruited HIV-positive PWUD in Vancouver, Canada, a setting of free and universal access to HIV care. Participant data were linked to comprehensive HIV clinical monitoring and ART dispensation records. We developed a series of generalized linear mixed effects models, adjusting for potential confounders. Models examine whether, among ART-exposed individuals, becoming optimally adherent to ART medication (i.e. ≥95% using a validated measure of pharmacy dispensation) resulted in associated social, socio-economic and ancillary clinical benefits, such as relationship initiation, transitioning out of homelessness, entering employment, ceasing involvement in illegal or prohibited income generation activity (e.g. street-based income generation, sex work, drug dealing or other illegal activities) and enrolling in addiction treatment.

**Results:**

Between December 2005 and November 2013, of the 724 eligible study participants, 241 (33.3%) self-reported as women and 404 (55.8%) as Caucasian, with 463 (64.0%) individuals becoming ≥95% adherent to ART at least once during the study period. In final multivariate models, becoming adherent to ART was positively and significantly associated with ceasing prohibited or illegal income generation activities (adjusted odds ratio (AOR): 1.52; 95% confidence interval (CI): 1.20–1.94) and transitioning out of homelessness (AOR: 1.38; 95% CI: 1.12–1.71), while ART adherence was marginally associated with initiating a romantic relationship (AOR: 1.31, 95% CI: 0.96–1.81).

**Conclusions:**

These findings suggest that becoming adherent to ART results not only in virologic suppression among HIV infected PWUD, but also increases the likelihood of reducing key drivers of social and socio-economic vulnerability. These secondary benefits of ART adherence hold the potential to reinforce ongoing engagement in HIV care and support significant improvements in quality of life and individual health among this marginalized population. Findings reinforce the clinical and non-clinical importance of promoting access and adherence to ART among HIV-positive individuals who use illicit drugs.

# Sub article: Modelling the impact of improvements in the cascade of care for chronic hepatitis C among people who inject drugs (PWID) in Montréal, Canada

**DOI:** 10.7448/IAS.18.5.20552

**Published:** 2015-07-22

**Authors:** Anthony Cousien, Pascale Leclerc, Carole Morissette, Julie Bruneau, Élise Roy, Yazdan Yazdanpanah, Joseph Cox

**Affiliations:** 1 INSERM, IAME, UMR 1137, Paris, France; 2 IAME, UMR 1137, Univ Paris Diderot, Sorbonne Paris Cité, Paris, France; 3 Direction de santé publique du Centre intégré universitaire de santé et de services sociaux du Centre-Est-de-l'Ile-de-Montréal, Montréal, Canada; 4 Centre de recherche, Centre hospitalier de l'Université de Montréal (CRCHUM), Montréal, Canada; 5 Faculté de médecine et des sciences de la santé, Université de Sherbrooke, Campus Longueuil, Longueuil, Canada; 6 Service des Maladies Infectieuses et Tropicales, Hôpital Bichat Claude Bernard, Paris, France; 7 Chronic Viral Illness Service, McGill University Health Centre, Department of Epidemiology, Biostatistics and Occupational Health, McGill University, Montréal, Canada

## Abstract

**Introduction:**

Since 2010, HCV incidence among active (i.e. injection past six months) PWID in Montréal remains greater than 15 of 100 person-years (p-y). The arrival of new direct-acting antivirals (DAA) with high sustained virological response rates and improved tolerability raises the question of whether treatment could be used to prevent HCV transmission. Our objective was to assess how improvements in the cascade of care can impact future HCV incidence, prevalence and complications among PWID in Montréal.

**Methods:**

We used a dynamic model to simulate HCV transmission and natural history among active PWID in Montréal from 2015. The reference scenario (scenario 1) was the current cascade of care including new DAA as standard treatment (see Table 10). HCV prevalence and incidence after 10 years and the number of liver complications avoided after 40 years were estimated under different conditions: decreased time from chronic infection to diagnosis (scenario 2), greater adherence to treatment (scenario 3), improved treatment rate (scenarios 4 and 5) and a combination of these interventions (scenario 6). Due to a lack of data on time to linkage to care (time between diagnosis and first consultation related to hepatitis C), simulations considered three such intervals: one, three and five years. A thousand simulations were performed per scenario.

**Results:**

Scenarios 2 and 3 showed similar results for HCV prevalence (53.3–59.5%) and incidence (9.1–10.3/100 p-y) after 10 years, and less than a 3.4% difference in the number of liver complications after 40 years relative to the reference scenario. Improving access to treatment (scenarios 4 and 5) demonstrated a great decrease in all outcomes. When combining all interventions (scenario 6), prevalence and incidence decreased until 26.9% and 4.9/100 p-y, respectively, and the number of liver complications until 39.3%, depending on the time to linkage to care.

**Conclusions:**

Our results suggest that decreasing time to diagnosis or improving treatment adherence is not sufficient to impact HCV prevalence, incidence and complications among PWID in Montréal. The current level of treatment access in the cascade of care is limiting a massive decrease in disease burden and transmission. A substantial treatment scale-up is necessary in this population.

**Table:** Abstract TUAC0406LB–Table 1

Scenario	Average time before linkage to care (years)	Prevalence after 10 years (%) mean (95% CI)	Incidence after 10 years (/100 persons-years) mean (95% CI)	% of compilation avoided compared to Scenario 1 over 40 years mean (95% CI)
Scenario 1 (Reference)				
New DAAs under the current cascade of care:	1	54.6 (54.4;54.8)	9.4 (9.1;9.6)	/
average time from chronic infection to diagnosis δ=2.0 years;	3	57.7 (57.5;57.8)	10.1 (9.8;10.4)	/
annual lost to follow-up probability Ψ=14%;	5	59.5 (59.4;59.7)	10.3 (10;10.6)	/
initiation of treatment if linked to care α=5%/year;				
SVR rate with current adherence to treatment				
(SVR)=81.3%				
Scenario 2	1	53.5 (53.3;53.7)	9.1 (8.9;9.4)	0.9 (−0.2;2.0)
Decrease time from chronic infection to diagnosis:	3	56.8 (56.7;57)	9.9 (9.6;10.1)	1.7 (0.8;2.6)
δ=0.5 years	5	58.7 (58.5;58.9)	10.1 (9.9;10.4)	1.7 (0.7;2.7)
Scenario 3	1	53.3 (53.1;53.5)	9.1 (8.8;9.4)	2.7 (1.6;3.8)
Improve adherence to treatment:	3	56.6 (56.5;56.8)	9.6 (9.4;9.9)	3.2 (2.2;4.2)
SVR rate likewise in clinical trials (SVR=90%)	5	58.5 (58.3;58.7)	9.8 (9.5;10.1)	3.4 (2.6;4.3)
Scenario 4	1	45.6 (45.4;45.8)	7.8 (7 6;8.0)	15.5 (14.7;16.3)
Improve treatment rate:	3	50.5 (50.3;50.7)	8.4 (8.2;8.7)	14.6 (13.7;15.4)
α=10%/year	5	53.5 (53.4;53.7)	9.1 (8.9;9.4)	12.4 (11.5;13.2)
Scenario 5	1	34.1 (33.9;34.3)	6.1 (5.9;6.3)	29.6 (28.9;30.2)
Improve treatment rate:	3	41.4 (41.2;41.6)	7.3 (7.1;7.5)	27.2 (26.3;28.0)
α=20%/year	5	45.7 (45.5;45.9)	7.8 (7.6;8.1)	24.3 (23.6;25.1)
Scenario 6	1	26.9 (26.7;27.0)	4.9 (4.8;5.1)	39.3 (38.8;39.8)
Combined scenario	3	35.7 (35.5;35.8)	6.4 (6.2;6.6)	34.8 (34.2;35.4)
combine scenarios 2, 3 and 5	5	41.2 (41;41.4)	7.2 (7.1;7.4)	31.6 (30.8;32.4)

CI: confidence interval

SVR: Sustained virological response

# Sub article: Where to strengthen care: model-based triangulation of trends in the HIV care cascade

**DOI:** 10.7448/IAS.18.5.20384

**Published:** 2015-07-22

**Authors:** Anna Bershteyn, Daniel Klein, Kevin Oishi, Philip Eckhoff

**Affiliations:** Institute for Disease Modeling, Bellevue, United States

## Abstract

**Introduction:**

The HIV “cascade of care” provides a framework for identifying priority areas for improvement of HIV services. In sub-Saharan Africa, the cascade has yet to be characterized nationally due to challenges such as distinguishing first initiation of care from re-initiation absent unique patient identifiers. Lacking direct data characterizing the cascade, we hypothesize that national-level temporal trends in care can be triangulated based on epidemiological, actuarial and programmatic information fed into a quantitative model.

**Methods:**

We simulated the HIV care cascade in South Africa using an epidemiological model calibrated to age- and gender-specific HIV prevalence and mortality, national population dynamics and monitoring data from the public-sector HIV treatment programme. Data were available up to 2012, beyond which we assumed continuation of current trends in scale-up. HIV-associated mortality in the model was classified into those dying without initiating care, having initiated late (CD4<200), lost to follow-up (LTFU) after previous initiation or currently in care.

**Results:**

Failure to initiate care constituted the largest but most rapidly declining category of HIV mortality, predicted to decline from 47% of HIV-associated deaths in 2015 to 37% in 2020. Late initiation was the second-largest and declined more slowly because increasing CD4 counts at initiation were partially offset by growing numbers of patients initiating care. LTFU was the third-largest but the most rapidly-growing category of HIV mortality. Programmatic data about re-initiation of care is lacking, but under the assumption that half of patients LTFU will re-initiate care, deaths LTFU were not expected to surpass deaths due to late initiation by 2020. Those receiving care constituted 3% of HIV-associated deaths, mostly among those receiving treatment rather than in pre-ART care. This proportion remained constant over time because the growing population on treatment was offset by improvements in treatment quality, such as expansion of virological monitoring and availability of second-line regimens.

**Conclusions:**

More data are required to fully characterize the spatial heterogeneities and dynamics of the care cascade. Nevertheless, trends revealed by model-based triangulation were consistent with findings in well-studied populations such as demographic surveillance sites. Failure to access care remains the largest but most rapidly declining category of HIV mortality.

**Abstract TUAD0101–Figure 1:** HIV mortality along the care cascade.

# Sub article: Optimizing tools for measuring short-term antiretroviral adherence from pharmacy refill data to predict virologic outcomes in resource-limited settings

**DOI:** 10.7448/IAS.18.5.20385

**Published:** 2015-07-22

**Authors:** Rory Leisegang, James Mcmahon, Michael Hislop, Kathryn Stinson, Andrew Boulle, Julian Elliott, Gary Maartens

**Affiliations:** 1 Department of Medicine, Division of Clinical Pharmacology, University of Cape Town, Cape Town, South Africa; 2 Department of Infectious Diseases, Alfred Hospital, Melbourne, Australia; 3 Aid for AIDS, Afrocentric Health Pty Ltd, Cape Town, South Africa; 4 Department of Public Health, University of Cape Town, Cape Town, South Africa; 5 Centre for Population Health, Burnet Institute, Melbourne, Australia

## Abstract

**Introduction:**

Estimates of adherence to antiretroviral therapy (ART) using pharmacy refill data have outperformed self-report and can identify patients at risk for virologic failure, especially in settings where viral load testing is limited. Uncertainty exists about the best method to estimate adherence using pharmacy refill data and the optimal duration of data to predict virologic outcomes.

**Methods:**

We identified individuals over 18 on first and second line ART from a national private sector (Aid for AIDS) and regional public sector (Khayelitsha) programme. The area under (AUC) the receiver operating characteristic (ROC) curves for virologic suppression (VS) (viral load<400 copies/mL) was used to compare three short-term adherence estimate methods:

1) “crude” – refills divided by months,

2) “average” – days ART dispensed plus unused ART from prior dispensing divided by interval duration, and

3) “gap” – interval duration less the number of days without ART coverage, divided by interval duration.

The “gap” method is different to the “average” method as it does not allow the adherence estimate to be artificially increased by additional ART dispensed after a possible “gap” in ART coverage. The interval for pharmacy refill varied from 3 to 12 months.

**Results:**

We included 56,472 individuals from the private programme (median 1.7 years, 65% female) and 24,466 from the public programme (median 2.1 years, 65% female). The “gap” method consistently outperformed the other two methods (see Figure 1). In the public programme, the “gap” method was 12% less potent due to significant data capture errors. Longer pharmacy refill intervals outperformed shorter intervals (“gap” ROC 0.837 (12 months), 0.812 (3 months)) in the more powered private dataset. When further separated by regimen line, the “gap” method for second line was superior but the ROC AUCs estimates did not vary by the pharmacy refill interval. We identified possible cut-points for virological failure (VL>1000 copies/mL) in the private programme: 80 and 72% for first and second line therapy. respectively.

**Conclusions:**

Adherence measures that identify gaps in pharmacy data were superior and consistent across programs and regimen lines and could be used to identify people at risk of poor ART outcomes.

**Abstract TUAD0102–Figure 1:** Area under ROC curve with 95% CI (private programme).

# Sub article: Estimating national coverage of antiretroviral therapy among HIV-infected persons using multiple methods, Kenya 2012

**DOI:** 10.7448/IAS.18.5.20386

**Published:** 2015-07-22

**Authors:** Andrea Kim, Lucy Nganga, Irene Mukui, Joyce Wamicwe, Sofie Mwanyumba, Nancy Bowen, Lubbe Wiesner, Kevin De Cock, KAIS Study Group

**Affiliations:** 1 Division of Global HIV/AIDS, US Centers for Disease Control and Prevention, Nairobi, Kenya; 2 National AIDS and STI Control Programme, Ministry of Heath, Nairobi, Kenya; 3 National Public Health Laboratory Services, Ministry of Heath, Nairobi, Kenya; 4 Division of Pharmacology, University of Cape Town, Cape Town, South Africa

## Abstract

**Introduction:**

Accurate estimates of antiretroviral therapy (ART) coverage are needed to track progress towards global targets from the Joint United Nations Programme on HIV/AIDS (UNAIDS) which aim for 90% of HIV+ persons on ART by 2020. ART coverage is reported annually to UNAIDS using mathematically-modelled estimates of the number of HIV+ persons eligible for ART based on an assumed distribution of CD4 counts in the HIV+ population and the number of persons receiving ART in health facilities. We compared ART coverage reported to UNAIDS with coverage estimated from a nationally representative survey in Kenya using two independent methods.

**Methods:**

The 2012 Kenya AIDS Indicator Survey was a population-based household survey of persons aged 18 months-64 years conducted from 10/2012 to 2/2013. Interviews collected data on ART use for persons reporting HIV+ status. Blood samples were tested for HIV, and HIV+ samples tested for ART by High Performance Liquid Chromatography coupled to Tandem Mass Spectrometry. We estimated and compared ART coverage among HIV+ persons aged 15–64 years based on: 1) routine programme monitoring data; 2) self-report; and 3) biological confirmation of ART. ART eligibility in the survey was defined as: CD4 count <350 cells/mm^3^ or having active tuberculosis. Estimates were weighted to adjust for survey design and non-response.

**Results:**

According to ART programme monitoring data, 549,000 adults were receiving ART in 2012, covering 39.6% (confidence interval (CI) 36.8–43.0) of HIV+ persons and 78.3% (CI 74.8–82.8) of those ART-eligible. Of 11,626 survey respondents, 648 (5.6%) were HIV+ and 559 (86.3%) had samples available for ART testing. Among those, 42.5% (CI 0.4–47.7) tested positive for ART while 34.2% (CI 29.1–39.3) reported receiving ART. Based on biological confirmation of ART, coverage among ART-eligible persons was 71.0% (CI 63.2–78.9) or 444,000 persons while coverage based on self-report was 63.4% (CI 53.2–73.6) or 374,000 persons.

**Conclusions:**

Self-report underestimated ART coverage by 70,000 persons while programme data may overestimate coverage by up to 105,000 persons. Until monitoring systems for the national ART programme are strengthened and mathematical models are updated to reflect actual need for ART, surveys that provide biological confirmation of ART may be required to accurately track national estimates of ART coverage.

# Sub article: Crowdsourcing to spur first-time HIV testing among men who have sex with men and transgender individuals in China: a non-inferiority pragmatic randomized controlled trial

**DOI:** 10.7448/IAS.18.5.20387

**Published:** 2015-07-22

**Authors:** Larry Han, Weiming Tang, John Best, Ye Zhang, Julie Kim, Fengying Liu, Katie Mollan, MIchael Hudgens, Barry Bayus, Fern Terris-Prestholt, Samuel Galler, Ligang Yang, Rosanna Peeling, Paul Volberding, Baoli Ma, Bin Yang, Shujie Huang, Kevin Fenton, Chongyi Wei, Joseph Tucker

**Affiliations:** 1 Biostatistics, University of North Carolina at Chapel Hill, Chapel Hill, United States; 2 Guangdong Provincial STD Control Center, Guangzhou, China; 3 Medicine, University of California – San Francisco, San Francisco, United States; 4 Kenan-Flagler Business School, University of North Carolina at Chapel Hill, Chapel Hill, United States; 5 London School of Hygiene & Tropical Medicine, London, United Kingdom; 6 Department of International Development, Oxford University, Oxford, United Kingdom; 7 Blue City, Beijing, China; 8 Public Health England, London, United Kingdom; 9 Epidemiology and Biostatistics, University of California – San Francisco, San Francisco, United States; 10 University of North Carolina Project – China, Guangzhou, China

## Abstract

**Introduction:**

Improving first-time HIV testing among key populations, especially young MSM and transgender (TG) individuals, is a global health priority. However, most HIV testing campaigns do not reach untested populations and have minimal input from key populations. Crowdsourcing, the process of taking a task performed by an individual and opening it to a large group in the form of a contest, may enhance HIV testing interventions. We organized a non-inferiority, pragmatic randomized controlled trial to compare first-time HIV testing rates among MSM and TG individuals who received either a crowdsourced HIV test promotion intervention or a health marketing intervention.

**Methods:**

Participants were recruited through three large MSM web portals in China. We randomly assigned 721 MSM and TG individuals (≥16 years old, never before tested for HIV) to one of two video interventions. The crowdsourced video was developed using an open contest and formal transparent judging while the evidence-based health marketing video was designed by experts. We followed up four weeks post-intervention via text message to assess HIV test uptake. Descriptive statistics and sensitivity analyses for missing data were carried out to assess test uptake. Cost-minimization analysis was used to evaluate economic and financial costs of the two interventions. The trial was registered (NCT02248558).

**Results:**

Overall, 624/721 (86.5%) MSM and TG individuals responded to the text message. HIV test uptake was similar between the crowdsourced arm (37.1%, 114/307) and the health marketing arm (35.0%, 111/317). Sensitivity analysis using imputation supported the similarity of the two approaches. Within the crowdsourced arm, individuals who previously viewed the video were more likely to receive HIV testing compared to first-time viewers (52.4% vs. 26.5%, p<0.001). Among those tested, 30.7% (69/225) reported a new HIV diagnosis. The crowdsourced intervention cost substantially less than the health marketing intervention in eliciting first-time testing ($131/person vs. $238) and detecting new HIV diagnoses ($415/person vs. $799).

**Conclusions:**

We provide proof of principle for using crowdsourcing as a tool to enhance community engagement and improve HIV testing services. Crowdsourcing may be a cost-effective method to optimize HIV interventions, especially interventions targeting young key populations.

**Abstract TUAD0104–Table 1:** Pre-specified sub-analyses among MSM in China

	Crowdsourced				Health marketing			
		
	Tested/total (%)	RR	95% CI	p	Tested/total (%)	RR	95% CI	p
Multi-time video watching	66/126 (52.4%)	1.97	1.47–2.65	<0.001	67/151 (44.4%)	1.67	1.23–2.28	0.001
First-time video watching	48/181 (26.5%)	Ref			44/166 (26.5%)	Ref		
Northern web portal	106/316 (33.5%)	1.27	0.70–2.33	0.42	90/266 (26.6%)	0.82	0.57–1.88	0.30
Other web portals	8/36 (22.2%)	Ref			21/51 (41.2%)	Ref		
Yes – condomless sex	28/71 (39.4%)	1.21	0.84–1.74	0.62	26/62 (41.9%)	1.30	0.90–1.88	0.16
No – condomless sex	50/153 (32.7%)	Ref			52/161 (32.3%)	Ref		
Overall	114/307 (37.1%)				111/317 (35.0%)			

# Sub article: The effect of a population-based health department Data-to-Care intervention to increase HIV care engagement and antiretroviral use: a controlled evaluation

**DOI:** 10.7448/IAS.18.5.20553

**Published:** 2015-07-22

**Authors:** Julia C Dombrowski, James P Hughes, Susan E Buskin, Jane M Simoni, David Katz, Mark Fleming, Angela Nunez, Matthew R Golden

**Affiliations:** 1 Medicine, University of Washington, Seattle, United States; 2 HIV/STD Program Public Health – Seattle & King County, Seattle, United States; 3 Biostatistics, University of Washington, Seattle, United States; 4 Epidemiology, University of Washington, Seattle, United States; 5 Psychology, University of Washington, Seattle, United States

## Abstract

**Introduction:**

The US CDC promotes the use of HIV surveillance data to identify out-of-care persons and return them to care (“Data-to-Care”).

**Methods:**

We used stepped wedge cluster randomization to institute a Data-to-Care programme in Seattle-King County, Washington, DC, USA. We attempted to provide the intervention to all eligible persons in the county, initiated in a randomly assigned order based on cases’ medical provider (the cluster). Eligible persons had 1) no CD4 or viral load (VL) reported for ≥12 months or 2) VL>500 and CD4<350 at last report. Programme staff contacted patients to offer assistance relinking to HIV care and treatment. The primary study outcome was time to viral suppression (first VL <200 reported to surveillance), starting from the programme implementation date. The secondary outcome was care relinkage (first VL or CD4 reported). We used Cox Proportional Hazards to compare outcomes during control periods (before initiation of each case's provider cluster) to intervention periods (after initiation of the cluster). We censored cases at the time of ascertainment of relocation or death, or end of the observation period. The intention-to-treat (ITT) analysis included all eligible cases; the modified ITT (mITT) analysis excluded cases found to have died or moved.

**Results:**

The ITT and mITT analyses included 1008 and 824 persons, respectively (Figure). Study staff provided the individual intervention to 165 persons, of whom 73% relinked to care within one month and 70% achieved viral suppression within six months. The incidence rate (IR) of viral suppression was higher during the intervention versus control periods, but the difference was not statistically significant (Table). The HR associated with the intervention was higher among persons with last VL>500 in the past year than persons with no labs in the past year.

**Conclusions:**

Data-to-Care programmes can relink some persons to HIV care, but the effect of these programmes may be limited by the large numbers of persons who have moved, died or cannot be reached, and the rate of relinkage to care in the absence of the intervention. Focusing on persons with recently reported unsuppressed VLs rather than a gap in lab reports may be more effective and efficient.

**Abstract TUAD0105LB–Figure 1:** Flowchart of programme implementation.

**Abstract TUAD0105LB–Table 1:** Summary of intention-to-treat (ITT) and modified I

Population, outcome	% Achieved by end of observation period	Hazard ratio (95% CI) of incidence rates in intervention versus control period
Total population, viral suppression		
ITT analysis (N=1008)	30	1.27 (0.89–1.80)
mITT analysis (N=824)	37	1.18 (0.83–1.68)
No labs for 12 months, relinkage		
mITT analysis (N=276)	47	0.99 (0.74–1.34)
No labs for 12 months, viral suppression		
mITT analysis (N=276)	28	0.79 (0.40–1.55)
Last VL>500 in past year, viral suppression		
mITT analysis (N=548)	41	1.4 (0.96–2.19)

# Sub article: Retaining mother-baby-pairs in care and treatment: the mothers2mothers Mentor Mother Model

**DOI:** 10.7448/IAS.18.5.20388

**Published:** 2015-07-22

**Authors:** Kathrin Schmitz, Esca Scheepers, Emeka Okonji, Vicent Kawooya

**Affiliations:** 1 Department of Programmes and Technical Support, mothers2mothers, Cape Town, South Africa; 2 Research and Strategic Information, mothers2mothers, Cape Town, South Africa; 3 mothers2mothers Uganda, Jinja, Uganda

## Abstract

**Introduction:**

Retaining HIV-positive mothers and their babies in prevention of mother-to-child HIV transmission (PMTCT) care is critical for the elimination of mother-to-child-transmission. mothers2mothers is a peer education and psychosocial support programme operating in six Option B+ countries in Africa. m2m Mentor Mothers are women living with HIV who have recently experienced PMTCT. They are trained and employed to support other mothers and their families through the same process. In 2014, the m2m Mentor Mother Model implemented under the STAR-EC Programme in Uganda was evaluated externally in order to investigate whether maternal and infant PMTCT outcomes and maternal psychosocial well-being outcomes were associated with exposure to m2m Mentor Mothers.

**Methods:**

A quasi-experimental matched area comparison design was used. PMTCT outcomes were measured retrospectively among 2282 mother-baby-pairs who accessed PMTCT services between January 2011 and March 2014 in 31 intervention facilities (where m2m Mentor Mothers provided peer education and psychosocial support) and 31 matched control facilities (where no peer education and psychosocial support were provided). Furthermore, 796 pregnant women and new mothers accessing PMTCT between June 2012 and March 2014 across both study arms participated in facility based Psychosocial Wellbeing surveys. Bivariate and multivariate inferential statistical analysis was done using STATA 12. Propensity Score Matching was used to investigate the net effect attributable to the m2m standard-of-care.

**Results:**

Comparison of the intervention and control sites indicated that clients in m2m-supported health facilities showed improved uptake of PMTCT services (see Table 1).

The m2m model was further associated with increased coping self-efficacy (86.6% vs. 64.5%, p<0.001); coping behaviour (69.4% vs. 56.9%, p<0.001); HIV disclosure and safer sex self-efficacy (71.7% vs. 50.7%, p<0.001); and reduction in the experience of depression (83.5% vs. 78.1%, p=0.028).

**Conclusions:**

m2m has developed and refined a simple, scalable, adaptable and sustainable model of peer education and psychosocial support that improves uptake of PMTCT services and addresses the challenges facing HIV-positive pregnant women and mothers. The evidence shows that m2m's psychosocial peer support helps HIV-positive pregnant women and new mothers and their families cope more effectively with HIV and enhances their psychosocial wellbeing. Integration of peer education and psychosocial support into clinical PMTCT standard-of-care is recommended.

**Abstract TUAD0201–Table 1:** Comparison of PMTCT outcomes

Outcome indicator	Average effects among matched exposed subjects in m2m sites (%)	Average effects among matched unexposed subjects in control sites (%)	PSM net effect (percentage points)	p
Receipt of ARVs/ART for PMTCT among HIV-positive pregnant women	91.8	95.1	−3.3	<0.001
ANC attendance at least four times during pregnancy among HIV-positive women	49.30	39.70	9.6	<0.001
Delivery by skilled health personnel in past 12 months among HIV-positive women	87.10	75.80	11.3	<0.001
Retention in care among HIV-positive women 12 months after ART initiation	90.90	63.60	27.3	<0.001
Receipt of Nevirapine suspension at birth by HIV-exposed babies (ART prophylaxis for PMTCT)	86.00	59.00	27	<0.001
Percentage of HIV-exposed children who were given a PCR test at six weeks after birth	71.50	45.80	25.8	<0.001
Percentage of HIV-exposed children who were given an HIV test six weeks after cessation of breast feeding	60.50	31.40	29.4	<0.001
Percentage of HIV-exposed children who were given an HIV test 18 months after delivery	60.20	18.10	42.1	<0.001
Linkage of HIV-positive babies to paediatric ART	60.90	27.80	33	<0.001

# Sub article: Effectiveness of conditional cash transfers to increase retention in care and adherence to PMTCT services: a randomized controlled trial

**DOI:** 10.7448/IAS.18.5.20389

**Published:** 2015-07-22

**Authors:** Marcel Yotebieng, Harsha Thirumurthy, Kathryn Elizabeth Moracco, Bienvenu Kawende, Jean Lambert Chalachala, Landry Kipula Wenzi, Noro Lantoniaina Rosa Ravelomanana, Andrew Edmonds, Deidre Thompson, Emile Okitolonda, Frieda Behets

**Affiliations:** 1 College of Public Health, Ohio State University, Columbus, United States; 2 Department of Epidemiology, University of North Carolina at Chapel Hill, Chapel Hill, United States; 3 Department of Health Policy and Management, University of North Carolina at Chapel Hill, Chapel Hill, United States; 4 Department of Health Behavior, University of North Carolina at Chapel Hill, Chapel Hill, United States; 5 School of Public Health, University of Kinshasa, Kinshasa, Democratic Republic of the Congo

## Abstract

**Introduction:**

Novel strategies are needed to increase retention in, and adherence to prevention of mother-to-child HIV transmission (PMTCT) services, and ultimately enhance PMTCT implementation effectiveness in sub-Saharan Africa.

**Objective:**

To determine whether small, increasing cash payments conditioned on attending scheduled clinic visits and receiving proposed services can increase the proportion of HIV-infected pregnant women who attend PMTCT visits and adhere to available PMTCT services through six weeks postpartum.

**Methods:**

Newly diagnosed HIV-infected women, ≤32 weeks pregnant, were recruited at antenatal care clinics in Kinshasa, Democratic Republic of Congo, and randomly assigned in a 1:1 ratio to an intervention group that received compensation on the condition they attend scheduled clinic visits and accept offered PMTCT services ($5 plus $1 increment at each subsequent visit) or to a control group that received usual care. Outcomes assessed included: retention in care measured by loss-to-follow-up (LTFU), and adherence to PMTCT services (attend all scheduled clinic visits and accept proposed services) through six weeks postpartum. Analysis was by intention to treat. The study is registered with clinicaltrials.gov: NCT01838005.

**Results:**

Between April 2013 and August 2014, 612 potential participants were identified, 545 were screened and 433 were enrolled and randomized (Figure 1). Participants in the two groups had similar characteristics at baseline. As of January 5, 2015, 407 had completed their six weeks postpartum visit or were no longer in care. Analysis of complete data showed that by six weeks postpartum, a lower proportion of participants in the intervention group (17.7%) than the control group (27.0%) were LTFU (unadjusted odds ratio (OR), 0.58; 95% confidence interval (CI), 0.36–0.94). Similarly, a higher proportion of participants in the intervention group (70.0%) than the control group (54.5%) attended all scheduled visits and accepted proposed services (OR=1.91; 95% CI, 1.21–2.87). Results were similar after adjusting for marital status, age and education (Table 1).

**Conclusions:**

Among newly diagnosed HIV-infected women, small, incremental cash incentives resulted in increased retention along the PMTCT cascade and adherence to available services. The overall effects of these incentives on HIV-free survival and cost-effectiveness warrant further investigation.

**Abstract TUAD0202–Figure 1:** Participants tree.

**Abstract TUAD0202–Table 1:** Effect of conditional cash compensation

	Study group			Odds ratio (95% CI)			
		
	Overall n (%)	Intervention n (%)	Control n (%)	Unadjusted	p	Adjusted	p
Loss to follow-up							
Yes	91 (22.36)	36 (17.73)	55 (26.96)	0.58 (0.36, 0.94)	0.0255	0.58 (0.36, 0.93)	0.0235
No	316 (77.64)	167 (82.27)	149 (73.04)				
Attendance of each clinic visit and received services							
Yes	254 (63.41)	142 (69.95)	112 (54.90)	1.91 (1.27, 2.87)	0.0017	1.97 (1.30, 2.97)	0.0013
No	153 (37.59)	61 (30.05)	92 (45.10)				

# Sub article: Using the critical path for rapid expansion and optimization of a PMTCT programme towards elimination of new HIV infections in children

**DOI:** 10.7448/IAS.18.5.20390

**Published:** 2015-07-22

**Authors:** Reuben Musarandega, Agnes Mahomva, Joanna Robinson, Esther Tumbare, Priti Dave Sen, Anna Hakobyan, Angela Mushavi

**Affiliations:** 1 Technical Department, Elizabeth Glaser Pediatric AIDS Foundation, Harare, Zimbabwe; 2 Elizabeth Glaser Pediatric AIDS Foundation, Global, Washington, DC, United States; 3 Health Portfolio, Children's Investment Fund Foundation, London, United Kingdom; 4 Performance Measurement, Children's Investment Fund Foundation, London, United Kingdom; 5 AIDS and TB Unit, Ministry of Health and Child Care, Harare, Zimbabwe

## Abstract

**Introduction:**

Elizabeth Glaser Pediatric AIDS Foundation (EGPAF) partnered with the Children's Investment Fund Foundation (CIFF) and Zimbabwe Ministry of Health and Child Care (MOHCC) to roll out the WHO 2010 and later 2013 prevention of mother-to-child HIV transmission (PMTCT) guidelines. EGPAF, MOHCC and CIFF developed a “critical path” with a prioritized set of performance indicators, with population-based targets, that are the main drivers of impact. The indicators are reviewed quarterly, as they largely draw on routine monitoring data. If performance is lagging in a particular indicator, a diagnosis is undertaken to identify the reason and corrective action explored. Critical path indicators and results for quarter 2, 2012 are in Figure 1. The EGPAF-CIFF goal was to reduce mother-to-child transmission (MTCT) of HIV from about 25% in 2009 to less ~9% by 2015.

**Methods:**

Health facilities (HFs) were supported to implement the guidelines through training and mentoring during site support visits, among other assistance. PMTCT data were collected quarterly from all supported HFs, and performance of each indicator compared with established targets during data-driven programme reviews held by EGPAF, partner programme officers and MOHCC district staff. Reasons for under-performance and improvement strategies were identified and implemented in subsequent quarters through mentoring and coaching of HF staff to improve service provision and patient follow-up.

**Results:**

By October 2014, EGPAF was supporting 1480 out of 1560 sites to provide WHO 2013 PMTCT guidelines (Option B+). Service uptake in all critical path indicators increased significantly (p<0.001) from 2009/10 to 2013/14 as follows: ANC bookings 68–100%, HIV testing 85–98%, AZT prophylaxis 32–91%, CD4 testing 41–67%, ART initiation for pregnant mothers 18–85%, EID 13–71%, mothers’ adherence on ARV prophylaxis 34–77%. The national MTCT rate fell to ~9.0% in 2013.

**Conclusions:**

Through use of the critical path cascade, EGPAF and CIFF supported the MOHCC to achieve a rapid scale-up of PMTCT services. There is a need to maintain coverage and quality PMTCT services and ensure that children needing ART are actively identified, started and maintained on treatment. EGPAF is intensifying support in these new areas.

**Abstract TUAD0203–Figure 1:** PMTCT critical path.

# Sub article: Attrition from antiretroviral treatment services among pregnant and non-pregnant patients following adoption of Option B+ in Haiti

**DOI:** 10.7448/IAS.18.5.20391

**Published:** 2015-07-22

**Authors:** Jean Wysler Domercant, Nancy Puttkammer, Lydia Lu, Kesner Francois, Dana Duncan, Reynold Grand'Pierre, David Lowrance, Michelle Adler

**Affiliations:** 1 Centers for Disease Control and Prevention, Port au Prince, Haiti; 2 International Training and Education Center for Health, Seattle, United States; 3 Eagle Applied Sciences, Atlanta, United States; 4 Ministry of Health of the Government of Haiti, Port au Prince, Haiti; 5 Division of Global HIV/AIDS, Centers for Disease Control and Prevention, Atlanta, United States

## Abstract

**Introduction:**

Attrition from antiretroviral treatment (ART) services is an important determinant of HIV treatment outcomes. This study assessed factors associated with attrition among pregnant and non-pregnant patients initiating ART following adoption of Option B+ (universal ART eligibility for HIV-infected pregnant women) in October 2012 in Haiti.

**Methods:**

Electronic medical records of adult patients initiated on ART from October 2012 to August 2014 at 73 health facilities (HF) from 8 of 10 Haitian administrative departments were analyzed. Within a survival analysis framework, attrition was defined as the first instance of failure to attend a HF visit for 90 days after a missed clinical or pharmacy-dispensing appointment, or an officially-recorded programme discontinuation, whichever came first. Known transfers to alternative HF were treated as censored observations, not attrition cases. ART initiations during or within 12 weeks after pregnancy were deemed Option B+ cases. The Kaplan-Meier method and Cox proportional hazards regression, stratified by HF, were used to determine attrition and associated factors.

**Results:**

Among 17,084 patients who initiated ART, 7719 (45.2%) were non-pregnant women, 5920 (34.7%) were men and 3445 (20.2%) were pregnant women. At six months, attrition was 15.6% (95% confidence interval (CI): 14.8–16.4) for non-pregnant women, 17.0% (16.1–18.0) for men and 30.1% (28.5–31.7) for pregnant women. At 12 months, attrition was 31.8% (95% CI: 30.6–33.0), 34.5% (33.2–35.9) and 50.8% (49.0–52.6) respectively. Adjusted for patient-level factors and HF, attrition risk was 63% higher among pregnant women and 16% higher among men, compared to non-pregnant women (p<0.001). Significant protective factors included: receiving psychosocial counselling (hazard ratio (HR): 0.84, p<0.001); cotrimoxazole prophylaxis (HR: 0.83, p<0.001); tuberculosis treatment (HR: 0.88, p<0.001) before ART initiation; having an HIV-positive household member (HR: 0.80, p<0.05); living in the same commune as the HF (HR: 0.94, p<0.05), and greater duration of pre-ART enrolment (HR: 0.99 for each 30-day increase, p<0.001).

**Conclusions:**

Following adoption of Option B+, ART attrition in Haiti was higher than that described in published reports from other resource-limited settings. Early, sustained and tailored interventions are urgently needed to reduce ART attrition in Haiti, particularly among pregnant women.

# Sub article: Evaluation of early experience implementing Option B+ in the northwest and southwest regions of Cameroon 2013–2014

**DOI:** 10.7448/IAS.18.5.20392

**Published:** 2015-07-22

**Authors:** Pius Muffih Tih, Edouard Katayi, Eveline Mboh, Esther Bonje, Edith Lem, Jacques Chirac Awa, Jennifer R Lim, Dana Duncan, Leah Petit, Flavia Bianchi, Thomas Welty, Mbamulu Nkemontoh Achu, Gladys Tayong, Gilbert Tene, Jembia J Mosoko, Omotayo Bolu

**Affiliations:** 1 Cameroon Baptist Convention Health Services, Bamenda, Cameroon; 2 Cameroon Baptist Convention Health Services, Mutengene, Cameroon; 3 Association of Schools and Programs of Public Health/U.S. Centers for Disease Control and Prevention, Allan Rosenfield Global Health Fellowship, Washington, DC, United States; 4 U.S. Centers for Disease Control and Prevention, Yaounde, Cameroon; 5 U.S. Centers for Disease Control and Prevention, Atlanta, United States; 6 Elizabeth Glazer Pediatric AIDS Foundation, Washington, DC, United States; 7 District Health Service, Department of Disease Control, Ministry of Public Health, Kumba, Cameroon; 8 District Health Service, Department of Disease Control, Ministry of Public Health, Bamenda, Cameroon; 9 QED Group, LLC, Washington, DC, United States

## Abstract

**Introduction:**

The Cameroon Ministry of Public Health began implementation of life-long antiretroviral treatment (ART) for HIV-positive pregnant and breastfeeding women (Option B+) in 2013. This evaluation assesses early ART acceptability, retention and Mother-to-child-transmission. Results will guide subsequent phases of the national rollout.

**Methods:**

From October, 2013, to June, 2014, we recruited participants from 22 purposefully selected health facilities in the Northwest and Southwest Regions for an observational cohort evaluation. HIV-positive pregnant and breastfeeding women, not currently on antiretrovirals (prophylaxis or treatment), were eligible to participate in the assessment. Option B+ was offered to all eligible participants, and a descriptive analysis was performed.

**Results:**

Of 1267 HIV-positive pregnant or breastfeeding women identified, 669 (53%) were eligible for the evaluation. Of those who were offered Option B+, 666 (99%) accepted life-long ART and 3 (<1%) accepted ART only during pregnancy and breastfeeding. As of October 2014, 569 (85%) women remained alive and on treatment; 8 (1.2%) died, 17 (3%) discontinued ART and 34 (5%) were lost to follow-up (LTFU). Fifty-six (8%) did not return for their first refill after ART initiation; this percentage varied from 2 to 8% between facilities. The six month retention for monthly cohorts of women initiating Option B+ was 77–91% (Figure 1). Of 409 infants born to the 669 women enrolled, 8 (2%) died, 3 (<1%) were LTFU. Four hundred and three (99%) received NVP prophylaxis within 72 hours of birth. By eight weeks post-partum, 342 (89%) were tested for HIV deoxyribonucleic-acid, 9 (3%) received a positive result. The remaining infants are not yet old enough for HIV status determination. All HIV-infected infants initiated ART.

**Conclusions:**

In Cameroon, Option B+ is highly accepted by HIV-positive pregnant and breastfeeding women and can achieve a high six month retention rate. Long-term retention, mortality and final mother-to-child-transmission after cessation of breastfeeding need further evaluation.

**Abstract TUAD0205–Figure 1:** Proportion of patients on ART Option B+ retained six months after treatment.

# Sub article: Improving early ANC attendance through community engagement and dialogue: project ACCLAIM in three African countries

**DOI:** 10.7448/IAS.18.5.20554

**Published:** 2015-07-22

**Authors:** Mary Pat Kieffer, Godfrey Woelk, Daphne Mpofu, Rebecca Cathcart, ACCLAIM Study Group

**Affiliations:** 1 Elizabeth Glaser Pediatric AIDS Foundation, Washington, DC, United States; 2 Department of Health Behavior and Health Education, Gillings School of Global Public Health, The University of North Carolina at Chapel Hill, Chapel Hill, United States; 3 Elizabeth Glaser Pediatric AIDS Foundation, Mbabane, Swaziland

## Abstract

**Introduction:**

Timing of first antenatal clinic (ANC) attendance in sub-Saharan Africa averages 24–25 weeks; however, to effectively prevent HIV transmission to infants, earlier ANC attendance and initiation of antiretroviral therapy are necessary.

Advancing Community Level Action for Improving Maternal and Child Health (MCH)/Prevention of Mother-to-Child HIV Transmission (PMTCT), known as ACCLAIM, a three-arm randomized trial in 45 clusters across Swaziland, Uganda and Zimbabwe, aims to improve access, uptake and retention in MCH/PMTCT services.

**Methods:**

The study randomized clusters and evaluated three interventions: 1) community leader engagement (participation in the *Community Leaders Institute*, mentoring to engage in community action); 2) Community Days and dialogues (community event with structured dialogues on MNCH/PMTCT, and provision of health services) and 3) male and female MCH classes (set of four structured sessions led by peer facilitators).

This sub-study analyzed early ACCLAIM results on earlier access to ANC services. Baseline gestational age (GA) data at first ANC visit were collected from health facilities before implementation and quarterly after implementation. We compared proportions of women attending ANC during first half of pregnancy (≤20 weeks’ gestation) at baseline and 6–12 months after interventions.

**Results:**

A total of 277 trained community leaders held >7000 community meetings and engaged>27,000 individuals in dialogues at Community Days, identifying and addressing barriers, misperceptions and harmful gender norms. The proportion of women attending ANC≤20 weeks’ gestation across the three countries increased by 36% from baseline; this trend was significant across the quarters observed (p<0.0001).

Attendance during the first trimester (≤12 weeks) also increased, from 11.7% (84/719) to 14.1% (102/721) in Swaziland (p=0.163), and from 3.4% (24/705) to 12.0% (97/809) (p<0.0001) in Zimbabwe (Uganda data not available). Community dialogues actively focused on the benefits of early ANC and addressed norms of waiting until the woman “shows” before seeking ANC.

**Conclusions:**

In our study, community based interventions have resulted in significant greater than one-third increase in ANC≤20 weeks’ gestation in three African countries. On-going data analysis will provide data on the full potential of open community dialogues by trained community leaders to change community norms and health-seeking behaviours such as early access to ANC and MCH/PMTCT services.

**Abstract TUAD0206LB–Table 1:** Change in gestational age at first antenatal care

Gestational age at first ANC	Baseline July–September 2013 (January–March 2014, Uganda) n=5071	6–12 months of implementation October–December, 2014 n=4799	p-value
≤20 weeks	1532 (30.2%)	1975 (41.2%)	p<0.0001
21+weeks	3539 (69.8%)	2824 (58.8%)	

# Sub article: Comparison of HIV-1 envelope specific IgA and IgG antiviral ability to prevent HIV-1 infection: additive, inhibitory and synergistic effects

**DOI:** 10.7448/IAS.18.5.20393

**Published:** 2015-07-22

**Authors:** Sandra Gerda Okala, Deborah F King, Robin J Shattock

**Affiliations:** 1 Medicine, Imperial College London, London, United Kingdom; 2 International AIDS Vaccine Initiative, London, United Kingdom

## Abstract

**Introduction:**

Despite the crucial role of IgA in mucosal immunity, very little is known about how IgG and IgA isotypes interact to prevent HIV-1 infection. This gap in the current knowledge was highlighted in the HIV-1 RV144 vaccine trial in which specific monomeric (m)IgA mitigated IgG effectors functions and correlated with increased risk of HIV-1 acquisition. Both IgG and dimeric (d)IgA are present in the female and male genital tracts, which are the main site of viral entry. However, the ratio of IgG to IgA varies between compartments. In this study, we compared the antiviral properties of IgG and IgA antibodies with the same epitope specificity at ratios found in genital secretions. Subsequently, we investigated whether the combination of antibody recognizing discrete epitopes but from the same isotype resulted in improved antiviral activities.

**Methods:**

CH31, b12, 2F5 and 7B2 mAbs binding to soluble HIV-1_BaL_ gp140 Env and kinetics parameters of these interactions were determined by competitive enzyme-linked immunosorbent assay and Bio-Layer Interferometry (BLI). HIV-1_BaL_ virus capture by the panel of mAbs was quantified by p24 ELISA, antibody mediated viral aggregation (AMVA) was determined using Nanoparticle Tracking Analysis (NTA) and neutralization activity by TZM-bl neutralization assay.

**Results:**

We demonstrated that IgGs captured significantly more virions than IgAs, and this was correlated with higher association rate constants whereas dIgA presented the ability to mediate viral aggregation. Strikingly, the combination of dIgA and IgG recognizing the same epitope did not elicit any additive effects. In contrast, IgG prevented dIgA binding to HIV-1_BaL_ gp140 Env and its ability to capture and aggregate HIV-1_BaL_ virions. However, mixtures of IgGs or dIgAs recognizing distinct epitopes but from the same isotype resulted in synergistic effects with higher proportions of captured viruses; antibody mediated viral aggregates and neutralization activities.

**Conclusions:**

This study compared the ability of IgG and dIgA to prevent HIV-1 infection with respect to the ratio IgG and dIgA found in genital secretions. Collectively, these results suggest that the combination of antibody targeting different epitopes provides enhanced general antiviral activities. Nonetheless, antibody binding to the same epitope but of different isotypes may lead to competition and inhibition of antiviral functions.

# Sub article: Anti-V3/glycan and anti-MPER neutralizing antibodies, but not anti-V2/glycan-site antibodies are strongly associated with higher anti-HIV-1 neutralization breadth and potency

**DOI:** 10.7448/IAS.18.5.20394

**Published:** 2015-07-22

**Authors:** Rajesh Abraham Jacob, Thandeka Moyo, Michael Schomaker, Fatima Abrahams, Berta Grau Pujol, Jeffrey Dorfman

**Affiliations:** 1 International Centre for Genetic Engineering and Biotechnology, Cape Town, South Africa; 2 Division of Immunology, University of Cape Town, Cape Town, South Africa; 3 Centre for Infectious Disease Epidemiology and Research, University of Cape Town, Cape Town, South Africa

## Abstract

**Introduction:**

Previous candidate HIV vaccines have failed to either induce wide-coverage neutralizing antibodies or substantially protecting vaccinees. Therefore, current efforts focus on novel approaches never before successfully used in vaccine design, including modelling epitopes. Candidate immunogen models identified by broadly neutralizing antibodies include the membrane proximal eternal region (MPER, recognized by 4E10, 2F5 and 10E8 monoclonal antibodies (mAbs)), V3/glycans (typified by PGT121-128 mAbs) and the V2/glycan site (initially defined by PG9 and PG16 mAbs). Anti-MPER and anti-V3/glycan antibodies are often autoreactive or polyreactive, and this is thought to pose both direct and indirect barriers to achieving neutralization breadth. Recent evidence shows that antibodies with moderate neutralization breadth are frequently attainable, with 50% of sera from chronically-infected individuals neutralizing ≥50% of a large, diverse set of viruses. Such moderately neutralizing antibodies may be more attainable in vaccinees. Despite these findings, there is little systematic information addressing which specificities are preferentially targeted among such commonly found, moderately broad neutralizing sera.

**Methods:**

We explored associations between neutralization breadth and potency and presence of neutralizing antibodies targeting MPER, V2/glycan site and V3/glycans in sera from 177 antiretroviral therapy-naive HIV-1-infected (>1 year) individuals recruited in Cape Town, South Africa.

**Results:**

Recognition of both MPER and V3/glycans was associated with increased breadth and potency. MPER-recognizing sera neutralized 4.62 more panel viruses than MPER-negative sera (95% prediction interval (PI) 4.41, 5.20), and V3/glycan-recognizing sera neutralized 3.24 more panel viruses than V3/glycan-negative sera (95% PI 3.15, 3.52). In contrast, V2/glycan site-recognizing sera neutralized only 0.38 more panel viruses (95% PI 0.20, 0.45) than V2/glycan site-negative sera and no association between V2/glycan site recognition and breadth or potency was observed.

**Conclusions:**

Despite autoreactivity of many neutralizing antibodies recognizing MPER and V3/glycans, antibodies to these sites are major contributors to neutralization breadth and potency in this cohort. This suggests that the autoreactivity effect is not critical and that the MPER and the V3/glycans should remain high priority vaccine candidates. The V2/glycan site result is surprising because broadly neutralizing antibodies to this site have been repeatedly observed. It may therefore be appropriate to focus on developing immunogens based upon the MPER and V3/glycans.

**Abstract WEAA0102–Table 1:** Broad/potent neutralization and target recognized

Category	Less potent (geo mean ID50<220)	Potently neutralizing (geo mean ID50>220)	Relative risk (95% CI)	p (X^2^)	Less broad (neutralizes<18/24 viruses)	Broadly neutralizing (neutralizes≥18/24 viruses)	Relative risk (95% CI)	p (X^2^)
Anti-MPER negative	124	20	1.00 (reference)		122	22	1.00 (reference)	
Anti-MPER positive	24	9	1.96 (0.99, 3.91)	0.061	23	10	1.98 (1.04, 3.78)	0.043
Anti-V2 glycan site negative	63	21	1.00 (reference)		62	22	1.00 (reference)	
Anti-V2 glycan site positive	29	5	0.59 (0.24, 1.43)	0.222	27	7	0.79 (0.37, 1.67)	0.522
Anti V3/glycans negative	75	17	1.00 (reference)		73	19	1.00 (reference)	
Anti-V3/glycans positive	12	9	2.32 (1.21, 4.46)	0.017	12	9	2.08 (1.10, 3.92)	0.033

# Sub article: Impact of HLA-B*35 alleles on HIV disease outcome in Mexico and Central America

**DOI:** 10.7448/IAS.18.5.20395

**Published:** 2015-07-22

**Authors:** Humberto Valenzuela-Ponce, Santiago Ávila-Ríos, Daniela Garrido-Rodríguez, Thalía García-Téllez, Maribel Soto-Nava, Tania Escamilla-Gómez, Claudia García-Morales, Jonathan Cortés-Álvarez, Ramón Hernández-Juan, Akio Murakami-Ogasawara, Carlos Mejía-Villatoro, Juan Pascale, Guillermo Porras, Carlos Quant, Ivette Lorenzana, Rita Meza, Elsa Palou, Marvin Manzanero, Gustavo Reyes-Terán, Mesoamerican HIV Project Group

**Affiliations:** 1 Center for Research on Infectious Diseases, National Institute of Respiratory Diseases, Mexico City, Mexico; 2 Hospital Roosevelt, Guatemala City, Guatemala; 3 Instituto Conmemorativo Gorgas de la Salud, Panama City, Panama; 4 Hospital Metropolitano Vivian Pellas, Managua, Nicaragua; 5 HIV National Program, Tegucigalpa, Honduras; 6 Ministry of Health, Belize, Belize

## Abstract

**Introduction:**

HLA-B*35 alleles have[SL1] been classified into two groups, PY and Px, based on residues 114/116 in the HLA peptide binding groove, defining the amino acid preference at position 9 of the peptides they present. B*35:02/35:03, part of the Px group, have been associated with rapid HIV disease progression in the context of HIV-1 B clade infection. As B*35 is the most prevalent HLA-B allelic group in Mexico and Central America (expressed in 41.4% of individuals), including a number of relatively unstudied B*35 alleles, we investigated HIV disease outcome in this cohort.

**Methods:**

HLA sequence-based typing was performed on 1971 chronically HIV-1 clade B infected, ART-naïve individuals from Mexico (n=1058), Guatemala (n=396), Nicaragua (n=218), Honduras (n=165), Panama (n=85) and Belize (n=49). Associations between HIV plasma viral load (pVL) and CD4 T cell count (CD4 count) with B*35 expression were evaluated using Mann–Whitney U-tests and Storey q values. Only HLA-B heterozygous individuals were compared in order to exclude confounding effects resulting from HLA homozygosity.

**Results:**

We observed 10 different B*35 alleles (n>5). Based on residues 114 and 116, B*35:01/08/14/16/17/20/43 were classified as PY, and B*35:02/03/12 as Px. Ranking HLA-B*35 alleles according to median pVL or CD4 count showed a wide spectrum of associated HIV disease outcomes. B*35:01 (PY) and B*35:12 (Px), which are not considered disease-susceptible alleles, were associated with higher pVL and lower CD4 count (p<0.05, q<0.05). B*35:12 detrimental effect was stronger in Guatemala and Nicaragua than in Mexico, and the magnitude of B*35:01 effect in each country was frequency-dependent. B*35:08 (PY) had a modest protective effect on disease outcome (although not statistically significant). No significant impact on median pVL or CD4 count was observed between HLA-B*35 PY (n=359) and Px (n=134) groups.

**Conclusions:**

These results challenge the B*35-PY/Px hypothesis, indicating that PY alleles can be disease-susceptible. Moreover, the previous observation that the negative effect of the B*35 group is due to all Px alleles is not supported by these data. Interestingly, differences in the detrimental effect of some B*35 alleles in different countries seemed to be frequency-associated, warranting further studies on HIV HLA-associated adaptation in previously uncharacterized populations.

# Sub article: Type-1 programmed dendritic cells induce primary CTL capable of effectively targeting the HIV-1 reservoir

**DOI:** 10.7448/IAS.18.5.20396

**Published:** 2015-07-22

**Authors:** Robbie B Mailiiard, Kellie N Smith, Paolo Piazza, James I Mullins, Charles R Rinaldo

**Affiliations:** 1 Infectious Diseases and Microbiology, University of Pittsburgh, Pittsburgh, United States; 2 Department of Microbiology, University of Washington, Seattle, United States; 3 Department of Medicine, University of Washington, Seattle, United States

## Abstract

**Introduction:**

The “kick and kill” strategy for the cure of chronic HIV-1 infection involves unmasking cells harbouring the latent viral reservoir followed by their immune elimination. We hypothesize that a broad priming of *de novo* rather than memory HIV-1 specific cytotoxic T-lymphocytes (CTL) will be required to effectively target the autologous HIV-1 reservoir, and that this “kill” can be best achieved using specifically programmed type-1 dendritic cells (DC1).

**Methods:**

Mature, IL-12p70 producing DC1 were generated using a combination of either TNFa, IL-1b, poly IC, IFNa and IFNg or CD40L and IFNg. Mature, IL-12 deficient DC were generated using either a combination of TNFa, IL-1b, IL-6 and PGE2 or CD40L alone. CD8^+^ T cells were purified from HIV-1 negative donors, and both naive (primary) and memory CD8^+^ T cells were isolated from HIV-1 infected Multicenter AIDS Cohort Study participants who were on virus-suppressive cART for several years. These cells were stimulated with autologous DC loaded with HIV-1 Gag peptides or autologous AT2-inactivated HIV-1. Resulting CTL activity was assessed by IFNg ELISPOT and antiviral cytotoxicity assays targeting autologous HIV-1 infected CD4^+^ T cells.

**Results:**

DC1 proved far superior to the IL-12-deficient DC for inducing primary CTL responses in both infected and uninfected donors. Importantly, DC1 required CD40L “help” at the onset of priming cultures for successful CTL induction and expansion. Both primary and memory CTL each responded to distinct autologous HIV-1 Gag peptides with robust IFNγ production. However, a broader targeting of known MHC class I-restricted epitopes was achieved by the primary CTL responders than the memory cells. Importantly, despite substantial IFNγ production by both T cell subsets, the primary CD8^+^ T cells were significantly superior to restimulated memory T cells in eradicated HIV-1 infected CD4^+^ T cells in the CTL assays.

**Conclusions:**

We demonstrate that naïve T cells from HIV-1 infected persons on cART have the repertoire and ability to be primed by high IL-12p70-producing DC1 to effectively target the HIV-1 reservoir, while memory CTL responses are suboptimal. These findings highlight the importance of directing HIV-1 curative strategies towards the induction of *de novo* rather than memory HIV-1-specific CTL responses.

# Sub article: Molecular determinants of HIV-1 permissiveness and persistence in gut-homing CD4+ T cells expressing the Th17 marker CCR6

**DOI:** 10.7448/IAS.18.5.20397

**Published:** 2015-07-22

**Authors:** Delphine Planas, Annie Gosselin, Patricia Monteiro, Jean-Philippe Goulet, Sandrine Da Fonseca, Aurelie Cléret-Buhot, Mohamed-Ali Jenabian, Jean-Pierre Routy, Petronela Ancuta

**Affiliations:** 1 CHUM-Research Center, Montreal, Canada; 2 Department of Microbiology, Infectiology and Immunology, University of Montreal, Montreal, Canada; 3 CATRaGENE, Montreal, Canada; 4 Department of Biological Sciences, Université du Québec à Montréal (UQAM), Montreal, Canada; 5 BioMed Research Centre, Université du Québec à Montréal (UQAM), Montreal, Canada; 6 Division of Hematology, McGill University Health Center, Montreal, Canada; 7 Immunodeficiency Service, McGill University Health Center, Montreal, Canada

## Abstract

**Introduction:**

HIV-infected CD4+ T-cells are enriched in gut-associated lymphoid tissues (GALT). The integrin a47 and CCR9 mediate imprinting for gut-homing, and their expression is induced by retinoic acid (RA), a vitamin A metabolite produced by GALT dendritic cells. We previously demonstrated that CD4+ T-cells expressing the Th17 marker CCR6 are permissive to HIV *in vitro*, harbour replication-competent HIV reservoirs in ART-treated subjects and that RA selectively increases HIV replication in these cells. To identify new molecular determinants of HIV permissiveness/persistence, we performed a genome-wide transcriptional analysis in RA-treated CCR6+ versus CCR6- T-cells.

**Methods:**

CD4+ T-cells were sorted from peripheral blood mononuclear cells by negative selection using magnetic beads (Miltenyi). Memory (CD45RA-) CCR6+ and CCR6- T-cells were sorted by flow cytometry (BDAriaII). Cells were stimulated *via* CD3/CD28 and cultivated in the presence or absence of RA (10 nM) for four days. Total RNA was extracted for microarrays analysis (HT 12v4 BeadChip, Illumina; >46,000 probe sets per chip). Validations of microarrays were performed by real-time PCR and/or flow cytometry. HIV-DNA integration was measured by nested real-time PCR. Functional validations were performed using RNA interference (Amaxa).

**Results:**

Among 15,303 “present calls,” 1538 and 1285 probe sets were modulated by RA in CCR6- and CCR6+ T-cells, respectively (p<0.05; fold change cut-off 1.3). Gene Set Variation Analysis (GSVA), Ingenuity Pathway Analysis (IPA) and *Gene Ontology* tools were used to identify pathways/individual transcripts specifically induced by RA in CCR6+ versus CCR6- T-cells. This signature included an increased expression of gut homing markers (a4β7, CCR9), HIV-1 coreceptors (CCR5, CXCR6) and also pathways linked to the regulation of T-cell activation (CD38, Lck, PTPN13 and MAP4K4), glucose metabolism (Glut1, Glut8), cell cycle (GADD45G), HIV replication *via* CCR5 expression (KLF2) and multidrug resistance (MDR1/ABCB1). In addition, the transcriptome of RA-treated CCR6+ T-cells showed decreased expression of known HIV-1 resistance factors (PPAR-g, CCL3 and CCL3L1).

**Conclusions:**

Our studies demonstrate that RA-mediated imprinting for gut-homing is associated with HIV permissiveness in CCR6+ but not CCR6- T-cells and reveal molecular mechanisms underlying these differences. These findings will orient the discovery of new therapeutic strategies aimed at limiting HIV permissiveness, and subsequently the size of HIV reservoirs, specifically in gut-homing Th17 cells.

# Sub article: The potential of attenuated *Mycobacterium tuberculosis* or BCG vaccines to enhance oral SIV acquisition in infant macaques

**DOI:** 10.7448/IAS.18.5.20555

**Published:** 2015-07-22

**Authors:** Kara Jensen, Myra Grace dela Pena-Ponce, Michael Piatak, William Jacobs, Glenn Fennelly, Katie Mollan, Michael Hudgens, Pamela Kozlowski, Angela Amedee, Jakob Estes, Jeffrey Lifson, Koen Van Rompay, Michelle Larsen, Kristina De Paris

**Affiliations:** 1 Microbiology & Immunology, University of North Carolina, Chapel Hill, NC, United States; 2 Frederick National Laboratory, Leidos Biomedical, Inc., Frederick, United States; 3 Albert Einstein Institute, New York, United States; 4 Rutgers New Jersey Medical School, Newark, United States; 5 CFAR, University of North Carolina, Chapel Hill, United States; 6 LSUHSC, Microbiology, Immunology, and Parasitology, New Orleans, United States; 7 CNPRC, UC Davis, Davis, United States

## Abstract

**Introduction:**

Infants bear a high burden of HIV-1 and tuberculosis (TB) infections, especially in sub-Saharan Africa. We previously demonstrated that the double auxotroph *Mycobacterium tuberculosis* (*Mtb*) strain mc^2^6435, engineered to co-express SIV Gag, was safe and immunogenic in neonatal macaques. Here, we tested the efficacy of an oral mc^2^6435 prime/intramuscular MVA-SIV boost regimen to protect against repeated low-dose oral SIVmac251 challenge in infant macaques.

**Methods:**

The study included 75 infant rhesus macaques. Mock-vaccinated infants (n=15) received saline. Vaccinated animals (n=60) received attenuated auxotroph *Mtb*-vaccines with or without SIV gag/env inserts (n=53) orally, or BCG (n=7) intradermally at birth at nine weeks, infants were exposed to a once-weekly low-dose oral SIVmac251 challenge regimen. Plasma viraemia was determined by real-time PCR. Cellular immune activation was determined by flow cytometric analysis in blood and tissues, soluble plasma markers were measured with a Procarta 37plex. Statistical analysis for risk-per-SIV exposure was determined by SAS and Kaplan-Meier plots; immune parameters were analyzed using Kruskal-Wallis with multiple Dunn's comparison.

**Results:**

A single administration of the mc^2^6435 vaccine at birth induced persistent immune activation that was associated with oral SIV acquisition after fewer challenges compared to mock-vaccinated infants. The human BCG vaccine resulted in similar enhanced acquisition of SIV, and BCG-vaccinated infants showed higher peak viraemia compared to mock- and *Mtb*-vaccinated infant macaques. The potential for enhanced oral SIV acquisition was independent of the mycobacterial vaccine strain, immunization route and boost regimen. Analysis of blood and tissue samples revealed that both *Mtb* and BCG vaccines induced immune activation of myeloid cell populations and CD4^+^ T cells, potential target cells of SIV. Immune activation was detected as early as three weeks post-vaccination and persisted for several months.

**Conclusions:**

Our results in the infant macaque model are consistent with BCG-induced immune activation of CD4^+^ T cells in human infants, reports of persistent monocyte activation in BCG-vaccinated human adults, and increased HIV-1 infection rates in human CD4^+^ T cells exposed to *Mtb* complex *in vitro*. Thus, in areas of high HIV-1 prevalence, TB vaccines need to be tested for their risk of enhancing HIV-1 susceptibility in human infants.

# Sub article: Long-term early antiretroviral therapy limits the HIV-1 reservoir size as compared to later treatment initiation but not to levels found in long-term non-progressors

**DOI:** 10.7448/IAS.18.5.20398

**Published:** 2015-07-22

**Authors:** Eva Malatinkova, Ward De Spiegelaere, Pawel Bonczkowski, Maja Kiselinova, Karen Vervisch, Wim Trypsteen, Margaret Johnson, Chris Verhofstede, Dany de Looze, Charles Murray, Sabine Kinloch-de Loes, Linos Vandekerckhove

**Affiliations:** 1 HIV Translational Research Unit, Department of Internal Medicine, Faculty of Medicine and Health Sciences, Ghent University and Ghent University Hospital, Ghent, Belgium; 2 Division of Infection and Immunity, Royal Free Hospital, London NW3 2QG, United Kingdom; 3 AIDS Reference Laboratory, Department of Clinical Chemistry, Microbiology and Immunology, Ghent University, Ghent, Belgium; 4 Department of Gastroenterology, Ghent University Hospital, Ghent, Belgium; 5 Department of Gastroenterology, Royal Free Hospital, London NW3 2QG, United Kingdom

## Abstract

**Abstract:**

**Introduction:**

Early initiation of long-term antiretroviral therapy (ART) may lead to viral control after treatment discontinuation. Recent evidence indicates that ART initiated within seroconversion limits the HIV-1 reservoir size. Insight into the reservoir in patients with different timings of ART as well as those who can control HIV-1 without therapy should further inform new treatment strategies.

**Methods:**

A cross-sectional study of HIV-1 reservoir size (total and integrated HIV-1 DNA) and dynamics (2-LTR circles and cell-associated HIV-1 unspliced RNA (usRNA)) was performed in peripheral blood mononuclear cells (PBMCs) in 84 HIV-1 infected patients from four cohorts in two clinical centres (London, UK and Ghent, BE): long-term treated patients with ART initiated during seroconversion (SRCV on ART; n=25) or chronic infection (Chronic ART; n=32), long-term non-progressors (LTNP; n=17) and ART-naïve recent seroconverters (Recent SRCV; n=10). Total HIV-1 DNA, 2-LTR and usRNA were measured by ddPCR and integrated HIV-1 DNA by *Alu*-HIV PCR. Clinical parameters including time on ART and aviremia, CD4 count and CD4/CD8 ratio were collected.

**Results:**

Median total HIV-1 DNA copies were: 92, 48, 137 and 1901 c/10^6^ PBMCs in SRCV on ART, LTNP, Chronic ART and Recent SRCV, respectively. Significantly lower levels of total (p=0.041) and integrated HIV-1 DNA (p=0.003) were detected in early as compared to chronically treated patients, however these were higher than those found in LTNP (Figure 1a and 1b). Interestingly, similar levels of integrated HIV-1 DNA were found in Recent SRCV compared to the Chronic ART cohort (p=0.104), confirming very fast seeding of the reservoir (Figure 1b). Levels of usRNA were significantly lower in early compared to chronically treated cohort (p=0.007), indicating a lower transcriptional activity in early treated patients and similar to LTNP (p=0.615). Furthermore, early treated patients exhibited a higher CD4/CD8 ratio as compared to chronically treated patients (p=0.009), suggesting lower levels of residual immune activation.

**Conclusions:**

Our data demonstrate that long-term early treated patients have smaller reservoir size as compared to patients treated during chronic infection, however not reaching levels found in LTNP. Interestingly, the reservoir dynamics in terms of 2-LTR and usRNA as well as the CD4/CD8 ratio in early treated patients are comparable to LTNP.

**Abstract WEAB0101–Figure 1:** Total HIV-1 DNA (a) and integrated HIV-1 DNA (b) levels in four patient cohorts. Data is shown as log10 copies/million (c/M) PBMC and significant p-values are indicated by *. Differences between the cohorts were determined by Wilcoxon Signed Rank test.

# Sub article: High rates of non-reactive HIV serology after antiretroviral treatment initiated in acute HIV infection

**DOI:** 10.7448/IAS.18.5.20399

**Published:** 2015-07-22

**Authors:** James L K Fletcher, Suteeraporn Pinyakorn, Mark de Souza, Siriwat Akapirat, Rapee Trichavaroj, Tippawan Pankam, Eugene Kroon, Donn Colby, Peeriya Prueksakaew, Duanghathai Suttichom, Jerome H Kim, Praphan Phanuphak, Nittaya Phanuphak, Jintanat Ananworanich, The SEARCH010/RV254 Study Group

**Affiliations:** 1 The Thai Red Cross AIDS Research Centre, SEARCH, Bangkok, Thailand; 2 U.S. Military HIV Research Program, Walter Reed Army Institute of Research, Silver Spring, United States; 3 Department of Retrovirology, Armed Forces Research Institute of Medical Sciences – United States Component, Bangkok, Thailand; 4 The Thai Red Cross Anonymous Clinic, Thai Red Cross AIDS Research Centre, Bangkok, Thailand; 5 Department of Medicine, Faculty of Medicine, Chulalongkorn University, Bangkok, Thailand; 6 Henry M. Jackson Foundation for the Advancement of Military Medicine, Bethesda, United States

## Abstract

**Introduction:**

Non-reactive HIV serology may be a marker of low HIV viral burden. We examined the evolution of HIV antibody in a cohort of individuals treated during acute HIV infection (AHI).

**Methods:**

Between April 2009 and December 2014, adults attending voluntary HIV testing in Bangkok, Thailand, were screened for AHI, by either pooled nucleic acid testing (NAT) of 4th generation immunoassay (4G IA) non-reactive samples or by 3rd (3G) or 2nd generation (2G) enzyme immunoassay (EIA) of 4G IA reactive samples. Immediate antiretroviral therapy (ART) was offered. Western blot and p24 quantification were performed for Fiebig staging. HIV serology at baseline, weeks 12 and 24 were performed.

**Results:**

Two hundred and thirty-three Thai adults were enrolled from 130,164 samples screened; three individuals did not initiate ART and were excluded from analysis. The median age of the volunteers was 27 years, and 95% were male. Median time from history of HIV exposure to enrolment was 18 days, and median time from enrolment to ART initiation was one day. Of 207 baseline 2G EIA non-reactive subjects, results were available for 150 at week 12 and 135 at week 24 (Table 1). At week 12, 34% were non-reactive by 2G, 3% by 3G and 20% by 4G IA; at week 24, 39% were non-reactive by 2G, 5% by 3G and 18% by 4G. Baseline HIV RNA<5 log_10_ copies/mL (p=0.02), CD4 count >350 cells/µL (p=0.01) and Fiebig stage 1 or 2 (p=0.03) were predictive of non-reactive 2G EIA at week 24. Lower AUC_0–24 week_ for HIV RNA was also associated with non-reactive 2G EIA at week 24 (p≤0.001, Figure 1). Seroreversion was uncommon. One of 23 individuals with reactive 2G EIA at baseline was non-reactive at week 24; 11 of 207 demonstrated transient 2G EIA reactivity at week 12.

**Conclusions:**

Approximately 40% of individuals who initiated treatment in AHI maintained non-reactivity to 2G EIA after 24 weeks of ART. Rapid ART initiation and HIV RNA decline as well as low HIV RNA and high CD4 at baseline predicted subsequent serological non-reactivity. HIV serologic non-reactivity is likely due to low viral burden, further supporting the benefits of early initiation of ART.

**Abstract WEAB0102–Figure 1:** Plasma viral load by 2G EIA reactivity.

**Abstract WEAB0102–Table 1:** Non-reactivity to enzyme immunoassay

Non-reactivity to HIV enzyme immunoassay, N (%)			

	Baseline (N=207)	Week 12 (N=150)	Week 24 (N=135)
2nd generation EIA	207 (100)	51 (34)^a^	53 (39)^a^
3rd generation EIA	99 (48)	5 (3)^a^	7 (5)^a^
4th generation IA	43 (21)	30 (20)	24 (18)

a McNemar's test, p<0.001, compared to baseline (Note: no significant difference between week 12 and 24).

# Sub article: Twenty-four weeks is too short to assess virological success in primary HIV infection treatment

**DOI:** 10.7448/IAS.18.5.20400

**Published:** 2015-07-22

**Authors:** Anne Vandendriessche, Thomas Mourez, Veronique Lemée, Yasmine Debab, Gilles Peytavin, Joel Ladner, François Caron, Jean-Christophe Plantier, Jeremie Leporrier

**Affiliations:** 1 Rouen University Hospital, Infectious Diseases, Rouen, France; 2 Rouen University Hospital, Virology, Rouen, France; 3 Université de Rouen, Institut de Recherche et d'Innovation Biomédicale (IRIB), Rouen, France; 4 APHP-Bichat Hospital, Pharmaco-Toxicology, Paris, France; 5 Université Paris 7, IAME Inserm 1137, Paris, France; 6 Rouen University Hospital, Epidemiology & Public Health, Rouen, France

## Abstract

**Introduction:**

The goal of HAART, in established HIV infection, is to obtain virological success (plasma HIV-RNA level (pVL)<40 copies/mL) associated with CD4 increase at 24 weeks of treatment (W24). Therefore, we analyzed whether such W24 end-point is also pertinent for patients treated for primary HIV infection (PHI).

**Methods:**

We conducted a 10-year retrospective analysis of the immuno-virological response in 55 adults receiving HAART within three months after diagnosis of PHI. Genotypic resistance tests were performed before HAART and at W24 for patients with virological failure (VF) as well as HAART plasma concentrations.

**Results:**

Patients were mostly men (n=48, 87%), White European (n=50, 91%), MSM (n=29, 52%) and mean age 35.9 years. At baseline, mean pVL was 2.6.10^6^ cp/mL (8.10^3^ to >10^7^) and mean CD4 count 479/mm^3^ (77–1003). Patients were mostly infected with subtype B HIV-1 (n=30, 54%). Due to the evolution of treatment recommendations over the 10-year study period, nine different combinations of HAART were used, including mostly TDF/FTC (n=38, 69%) and a protease inhibitor as third agent (n=49, 89%). At W24, 44/55 (80%) patients had pVL<40cp/mL, whereas 11/55 (20%) had low residual pVL (45–391 cp/mL; mean: 155). In these latter patients, we observed neither mutation associated with resistance nor inefficient drug concentration. VF was correlated in univariate analysis with a significantly higher mean baseline pVL (p=0.03) and a significantly lower mean baseline CD4 count (p=0.04) than patients with undetectable pVL at W24. There was no relationship between age, sex, ethnicity, source of contamination, HAART combination or VF at W24.

**Conclusions:**

Our results show that 24 weeks is too short to achieve virological success in patients with high pre-treatment pVL associated with low CD4 count. These data highlight that the usual W24 end-point to conclude virological success may not be appropriate in PHI.

# Sub article: HIV transmitted drug resistance declined from 2009 to 2014 among acutely infected MSM in Bangkok, Thailand

**DOI:** 10.7448/IAS.18.5.20401

**Published:** 2015-07-22

**Authors:** Donn Colby, Nittaya Phanuphak, Sunee Sirivichayakul, Peeriya Prueksakaew, Putthachard Saengtawan, Rapee Trichavaroj, Trevor Crowell, Jerome Kim, Jintanat Ananworanich, Praphan Phanuphak, RV254/SEARCH010 Study Group

**Affiliations:** 1 Thai Red Cross AIDS Research Center, SEARCH, Bangkok, Thailand; 2 Chulalongkorn University Hospital, Bangkok, Thailand; 3 Armed Forces Research Institute for Medical Sciences, Bangkok, Thailand; 4 U.S. Military HIV Research Program, Bethesada, United States; 5 Thai Red Cross AIDS Research Center, Bangkok, Thailand

## Abstract

**Introduction:**

Rates of transmitted drug resistance (TDR) have been reported to be 11–21% in the USA and Europe, where baseline genotype resistance testing prior to antiretroviral therapy (ART) is routine. In resource limited settings, baseline resistance testing is not the standard of care, but TDR data can ensure that first-line treatment regimens used in national HIV treatment programs remain effective.

**Methods:**

The RV254/SEARCH010 cohort has enrolled patients with acute HIV infection from the largest HIV testing and counselling centre in Thailand, since 2009. Patients have baseline genotype testing prior to initiating ART: TRUGENE HIV-1 (Siemens Healthcare Diagnostics, Australia) was used for the first 66 patients and a validated in-house method for the remainder. Mutations were categorized following the World Health Organization surveillance drug resistance mutation (SDRM) list. Prevalence of resistance was calculated by dividing the number of subjects with mutations by the number enrolled during each time period. Change in prevalence over time was assessed by chi-square test for trend. Time periods were combined into two-year blocks for analysis.

**Results:**

Genotype resistance test results were available from 184 of the first 186 subjects enrolled in the study; virus from two patients could not be amplified. Median age was 28 years, 95% were male and 92% were men who have sex with men (MSM). Median time (inter-quartile range, IQR) from HIV exposure to diagnosis was 18 (14–24) days. Median (IQR) HIV RNA was 5.7 (5.1–6.7) log_10_ copies/mL and was not significantly different between patients with and without resistance mutations. Median (IQR) CD4 was 352 (260–486) cells/mm^3^. Prevalence rates for resistance mutations are shown in the table. Overall TDR was 7.1%, declining from 12.5% in 2009–2011 to 4% in 2013–2014, although the change was not statistically significance (p=0.07). The mutations most commonly found were the M46I (n=3), K103N (n=2), Y181C (n=2) and M41L (n=2).

**Conclusions:**

TDR does not appear to be increasing among MSM in Thailand and may be declining. Routine genotype testing prior to initiating ART may not currently be necessary in this population, but surveillance for TDR should continue to monitor for any future changes.

**Abstract WEAB0104–Table 1:** Transmitted drug resistance among MSM in Bangkok

	Total	2009–2010	2011–2012	2013–2014	
					
	n (%)	n (%)	n (%)	n (%)	p
N enrolled	184	32	52	100	
Any resistance	13 (7.1)	4 (12.5)	5 (9.6)	4 (4.0)	0.07
N with RT genotype	183	32	51	100	
NRTI mutations	6 (3.3)	2 (6.3)	2 (3.9)	2 (2.0)	0.23
NNRTI mutations	4 (2.2)	3 (9.4)	1 (2.0)	0 (0)	0.03
N with PR genotype	180	32	50	98	
PI mutations	6 (3.3)	1 (3.1)	3 (6.0)	2 (2.0)	0.52

# Sub article: Social cohesion among sex workers has an independent effect on reduced client condom refusal in a Canadian setting

**DOI:** 10.7448/IAS.18.5.20402

**Published:** 2015-07-22

**Authors:** Elena Argento, Putu Duff, Brittany Bingham, Paul Nguyen, Steffanie Strathdee, Kate Shannon

**Affiliations:** 1 Gender & Sexual Health Initiative, BC Centre for Excellence in HIV/AIDS, Vancouver, Canada; 2 School of Population and Public Health, University of British Columbia, Vancouver, Canada; 3 Faculty of Health Sciences, Simon Fraser University, Burnaby, Canada; 4 Division of Global Health, Department of Medicine, University of California, San Diego, United States

## Abstract

**Introduction:**

Despite substantial evidence in low and middle-income settings that community-led empowerment and collectivization can be a powerful determinant of successful HIV prevention, there is limited understanding of the impact of connectedness among sex workers on HIV risk in the global north. This study longitudinally modelled the impact of social cohesion on client condom refusal among street and off-street sex workers in Vancouver, Canada.

**Methods:**

Longitudinal data were drawn from an open prospective cohort of female (trans*-inclusive) sex workers, AESHA (An Evaluation of Sex Workers Health Access), in Metro Vancouver (2010–2013). Participants were recruited through outreach to outdoor locations and hidden indoor and online venues and completed bi-annual interview questionnaires and HIV/STI testing by a project nurse. Lippman and colleagues’ *Social Cohesion Scale* measured community connectedness (i.e. perception of mutual aid, trust and support) among sex workers. Bivariable and multivariable logistic regression using generalized estimating equations (GEE) were used to examine the independent effect of social cohesion on client condom refusal over three-year follow-up.

**Results:**

Of 654 sex workers, one-third (n=221) reported client condom refusal over three-year follow-up. On average, a medium level of social cohesion was reported; median social cohesion scores were 24 (IQR 20–29, range=4–45). In the final multivariable confounder model, for every one point increase in the social cohesion score, the odds of client condom refusal decreased by 3%, (adjusted odds ratio=0.97; 95% CI: 0.95–0.99) after adjusting for age, injection drug use and place of solicitation.

**Conclusions:**

This is the first study to examine the independent effect of social cohesion on client condom refusal among sex workers in the global north. Findings suggest that community collectivization and sex worker-led empowerment efforts can have a direct protective effect on HIV risk reduction and shifting social norms among clients in the sex industry. Given public health and human rights concerns around new Canadian laws introduced this year to further criminalize sex workers’ ability to work together (C-36), these findings highlight the urgent need for legal reforms and a structural framework that better promotes sex workers’ ability to more formally collectivize, including sex worker-led efforts in the HIV response.

# Sub article: Understanding the financial lives of female sex workers: implications for economic strengthening interventions for HIV prevention

**DOI:** 10.7448/IAS.18.5.20403

**Published:** 2015-07-22

**Authors:** Emily Namey, Brian Perry, Jennifer Headley, Kouakou Albert Yao, Mariame Ouattara, Coulibaly Shighata, Michael Ferguson

**Affiliations:** 1 Social & Behavioral Health Sciences, FHI 360, Durham, United States; 2 Independent Consultant, Abidjan, Cote D'Ivoire; 3 Economic Development & Livelihoods, FHI 360, Washington, DC, United States

## Abstract

**Introduction:**

Many women's decisions about whether and how to participate in sex work are driven by financial considerations. Despite the importance of economic factors in structural interventions for HIV prevention, data on the financial practices of female sex workers (FSWs) on which to base economic strengthening programs for HIV risk reduction are limited.

**Methods:**

We collected qualitative data in Abidjan, Côte d'Ivoire, through structured participant observation activities conducted with 72 FSWs during non-working hours. Detailed notes were taken as FSWs discussed their expenditures, income-generation and saving and borrowing strategies. We also collected quantitative financial diary data from a sub-sample (n=33) of FSWs. Women who kept financial diaries did so for six weeks, meeting weekly with researchers to systematically discuss and record all financial transactions. Participant observation notes were coded and analyzed using qualitative thematic analysis. Data from financial diaries were analyzed using descriptive statistics.

**Results:**

All women in our sample reported sex work as their primary source of income; many supplemented their income with cash gifts and modest loans from clients, family or peer FSWs. Food, clothing and transportation accounted for the highest amounts of relatively-fixed spending. Around one-quarter of all expenses were related to costs of sex work (e.g. “work” clothing, beauty care, personal hygiene products, right to work payments, police pay-offs, etc.). Qualitatively, both income and expenditures were reported to fluctuate monthly (e.g. around pay day), seasonally (e.g. around holidays) and unexpectedly (e.g. illness or financial shocks). FSWs described saving money in their homes, through social tontines or through formal systems (mobile money or banks), to help manage expenditures. They also reported increasing their sex work activities (e.g. traveling to other areas, offering sex for goods) to bridge financial shortfalls.

**Conclusions:**

Economic strengthening interventions have, in theory, great potential to lower FSWs’ risks of HIV by lessening the financial drivers of sex work. Our findings offer a rare glimpse into the earning, spending, saving and borrowing practices of FSWs, providing evidence on which to base decisions about how best to design and implement economic strengthening elements of HIV prevention for FSWs.

# Sub article: High utilization of health services and low ART uptake among female sex workers (FSW) in three South African cities: results from the South Africa health monitoring study (SAHMS-FSW)

**DOI:** 10.7448/IAS.18.5.20404

**Published:** 2015-07-22

**Authors:** Tim Lane, Maria Sibanyoni, Albert Manyuchi, Thomas Osmand, Alexander Marr, Mike Grasso, Zach Isdahl, Nyaradzo Mutanha, Phakamile Makhubela, Mpho Silima, Nkosinathi Zuma, Helen Struthers, James McIntyre, Helen Rees, Francois Venter

**Affiliations:** 1 Center for AIDS Prevention Studies, University of California, San Francisco, United States; 2 Wits Reproductive Health & HIV Institute, Johannesburg, South Africa; 3 Anova Health Institute, Johannesburg, South Africa; 4 Center for AIDS Prevention Studies, University of California, San Francisco, United States

## Abstract

**Introduction:**

The 2012–2016 South Africa HIV National Strategic Plan calls for integrated behavioural and biological surveillance with female sex workers (FSW) to address critical HIV epidemiological and programmatic data gaps. In 2013–2014, we conducted the SAHMS-FSW in three metropolitan areas to estimate prevalence of HIV, syphilis and associated risk factors and assess current utilization of health and HIV services.

**Methods:**

We recruited 764 FSW in Johannesburg, 650 in Cape Town and 766 in Durban using respondent-driven sampling (RDS) to take behavioural surveys, access voluntary counselling and testing and provide blood samples for HIV and syphilis surveillance. Serological testing followed national standards. We used RDSAT (version 7.1) to estimate population-adjusted prevalence for HIV, syphilis, selected behavioural and programmatic indicators; and SPSS (version 18.0) for multivariate logistic regressions with selected RDS-adjusted behavioural and programmatic indicators to identify site-specific significant associations with HIV-infection. We report adjusted odds ratios (aOR) and 95% Confidence Intervals (95% CI) in Table 1: Predictors of HIV – South African Health Monitoring Study, 2014, and current ART utilization in Figure 1.

**Results:**

HIV prevalence was 71.8% (95% CI 56.5–81.2%), 39.7% (95% CI 30.1–49.8%) and 53.5% (95% CI 37.5–65.5%) in Johannesburg, Cape Town and Durban respectively. After controlling for age, consistent condom use and hazardous drinking, brothel-based FSW had significantly higher odds of HIV-infection in Cape Town (aOR 2.1, 95% CI 1.5–3.1) and Durban (aOR 2.3, 95% CI 1.4–3.9); those working both brothels and streets in Johannesburg were more likely to be HIV-positive (3.0, 95% CI 1.2–7.9). Those accessing healthcare in Johannesburg and Durban (aOR 1.4, 95% CI 1.2–1.6 and 1.3, 95% CI 1.1–1.4, respectively) and ANC services in Cape Town and Durban (aOR 1.6, 95% CI 1.1–2.4 and 1.8, 95% CI 1.1–3.0, respectively) were significantly more likely to be HIV-positive. However, uptake of ART remains low among FSW.

**Conclusions:**

Although FSW accessing healthcare services are more likely to be HIV-positive, current ART utilization demonstrates a substantial gap to be addressed as South Africa begins implementing universal treatment. Identification and expansion of effective outreach models are needed to increase utilization of ART, as well as effectively target prevention services for HIV-negative FSW. Health outreach strategies must account for behavioural and structural factors in specific sex-work environments.

**Abstract WEAC0103–Table 1:** Predictors of HIV – South African Health Monitoring Study, 2014, and current ART utilization

	Johannesburg		Cape Town		Durban	
			
	aOR	95% CI	aOR	95% CI	aOR	95% CI
Venue of sex work (street based is reference)						
Brothel based only	0.59	0.35–0.99	2.13	1.46–3.12	2.31	1.37–3.89
Street and brothel based only	3.01	1.15–7.89	0.01	0.0–149.77	0.18	0.02–1.69
Health care utilization	1.35	1.16–1.56	–	–	1.26	1.13–1.41
ANC utilization	0.28	0.17–0.47	1.63	1.10–2.43	1.8	1.07–3.03
Peer education exposure	3.1	1.88–5.12	0.31	0.19–0.51	–	–
UAI with non-paying partner	–	–	–	–	28.55	10.52–77.56
Age	1.13	1.08–1.18	–	–	1.19	1.15–1.24

**Abstract WEAC0103–Figure 1:** 

# Sub article: Closing the gap: integrating mobile HIV testing and point-of-care CD4 testing for timely identification of HIV-infected and ART-eligible venue-based female sex workers in Lilongwe, Malawi

**DOI:** 10.7448/IAS.18.5.20405

**Published:** 2015-07-22

**Authors:** Kathryn Lancaster, Thandi Lungu, Mina Hosseinipour, Katy Chadwick, Zoe Dibb, Vivian F Go, Brian W Pence, Kimberly A Powers, Irving F Hoffman, William C Miller

**Affiliations:** 1 University of North Carolina, Chapel Hill, NC, United States; 2 UNC Project, Lilongwe, Malawi; 3 Theatre for a Change, Lilongwe, Malawi

## Abstract

**Introduction:**

Female sex workers (FSW) are a hard-to-reach key population in sub-Saharan Africa with high HIV prevalence, infrequent access to HIV care services, and low uptake of antiretroviral therapy (ART). We describe HIV seroprevalence, HIV status awareness, and ART eligibility and use for venue-based FSW in Lilongwe, Malawi who received integrated mobile HIV and point-of-care (POC) CD4 testing.

**Methods:**

From July through August 2014, FSW were recruited using venue-based sampling. A total of 200 FSW, age ≥18 years, who reported exchanging money for sex in the past 12 months participated in a biological and behavioural survey to evaluate HIV testing, care, and treatment history. Seropositive FSW, identified using HIV rapid testing, received rapid Alere Pima CD4 counts. Eligibility for ART followed the Malawi national guidelines (CD4≤500 cells/mm^3^, currently pregnant or breastfeeding, or any pregnancy after July 2011 following Option B+ policy). Proportions were estimated for HIV seroprevalence, self-reported previous HIV diagnosis, ART-eligibility based on national guidelines, and self-reported ART use.

**Results:**

HIV seroprevalence was 69% (n=138); 20% (n=27) were newly diagnosed, and 80% (n=111) were previously diagnosed. Among those newly diagnosed, 63% (n=17) were identified as ART-eligible (median CD4: 305; IQR: 237–427). Among those who were previously diagnosed, 65% (n=72) were currently on ART, 22% (n=24) were currently ART-eligible but not on ART (median CD4: 391; IQR: 261–474) and 13% (n=15) were ART-ineligible and not on ART. The most commonly reported reason among previously diagnosed and ART-eligible FSW for not being on ART was a prior high CD4 count (17%; n=4).

**Conclusions:**

This study is one of the first to integrate mobile HIV and POC CD4 testing to identify HIV-infected and ART-eligible venue-based FSW in Malawi. The majority of newly diagnosed FSW were immediately identified as ART-eligible. A substantial proportion of previously diagnosed FSW were ART-eligible but not on ART, with many having a prior high CD4 count. Large-scale integration of frequent HIV and POC CD4 testing for timely identification of HIV-infected and ART-eligible FSW is urgently needed to improve health outcomes for FSW and decrease HIV transmission in sub-Saharan Africa.

# Sub article: Injection drug use among female sex workers in Iran: findings of the first national bio-behavioural study

**DOI:** 10.7448/IAS.18.5.20406

**Published:** 2015-07-22

**Authors:** Abbas Sedaghat, Mohammad Karamouzian, Hamid Sharifi, Ali Mirzazadeh, Mostafa Shokoohi, Aliakbar Haghdoost

**Affiliations:** 1 Ministry of Health and Medical Education, Center for Diseases Control, HIV/STIs Office, Tehran, Iran, Islamic Republic of Iran; 2 Regional Knowledge Hub and WHO Collaborating Center for HIV Surveillance, Institute for Future Studies in Health, Kerman University of Medical Sciences, Kerman, Iran, Islamic Republic of Iran

## Abstract

**Introduction:**

While the prevalence of HIV among female sex workers (FSW) in Iran is approximately 4.5%, FSW who have ever injected drugs are believed to have a significantly higher HIV prevalence. This study tries to assess the determinants of injection drug use among FSW through Iran's first and only national bio-behavioural surveillance survey.

**Methods:**

This survey was conducted in 2010, by recruiting 827 FSW through facility-based sampling from 21 sites in 14 cities in Iran. Data were collected through face-to-face interviews using a pilot-tested standardized risk assessment questionnaire. All analyses were weighted based on the response rate and adjusted for the clustering effect of the sampling sites. A predictive multivariable logistic regression model was constructed to investigate the determinants of injection drug use among FSW in Iran.

**Results:**

Mean age of participants was 32, 50% had primary school educations, 36% were married and most of them reported sex work as their primary source of income. Of all participants, 71.6% (95% CI: 68.5–74.6) had ever used drugs and 14.6% (95% CI: 12.2–16.9) had ever injected drugs. The most frequently injected drugs were methadone, crystal methamphetamine and crack. Among those who had ever injected drugs, 36.6% reported that they had a drug injection during the previous month and the prevalence of HIV was 11.2% (95% CI: 5.4–21.5). In the multivariable model, history of HIV testing (AOR=1.79, 95% CI: 1.19–2.69), duration of sex work (AOR=1.08, 95% CI: 1.04–1.12), drug use before sex in the past month (AOR=2.70, 95% CI: 1.79–4.10) and alcohol use before sex in the past month (AOR=2.07, 95% CI: 1.35–3.17) were significant predictors of injection drug use.

**Conclusions:**

The prevalence of injection drug use among FSWs in Iran is concerning which calls for special attention to be paid to FSWs who inject drugs. As selling sex to cover drug habit expenses is a likely practice among female drug users, a part of harm reduction programs for drug users should try to target this population in order to reduce their sex work practices.

# Sub article: Engagement in the HIV care cascade and predictors of uptake of antiretroviral therapy among female sex workers in Port Elizabeth, South Africa

**DOI:** 10.7448/IAS.18.5.20556

**Published:** 2015-07-22

**Authors:** Sheree Schwartz, Andrew Lambert, Nancy Phaswana-Mafuya, Claire Holland, Zamakayise Kose, Mfezi Mcingana, Stephanie Sweitzer, Stefan Baral

**Affiliations:** 1 Epidemiology, Center for Public Health and Human Rights, Johns Hopkins University, Baltimore, United States; 2 TB/HIV Care Association, Cape Town, South Africa; 3 HIV/AIDS, STIs and TB (HAST), Human Sciences Research Council, Port Elizabeth, South Africa; 4 Epidemiology, Center for Public Health and Human Rights, Johns Hopkins School of Public Health, Baltimore, United States; 5 TB/HIV Care Association, Port Elizabeth, South Africa

## Abstract

**Introduction:**

Female sex workers (FSW) are 13-times more likely to be living with HIV than other reproductive-aged women. Data on FSW engagement in the HIV care cascade are limited, but suggest high rates of drop-off prior to viral suppression, with substantial drop-offs at HIV diagnosis.

**Methods:**

FSW≥18 years were recruited through respondent driven sampling into a cross-sectional study in Port Elizabeth, South Africa. Socio-demographics, reproductive, behavioural and healthcare history were assessed through interview-administered questionnaires. All FSW were tested for HIV, and CD4 counts were assessed among women living with HIV. Engagement in the HIV care cascade is described, and predictors of self-reported antiretroviral therapy (ART) uptake among treatment-eligible, previously diagnosed FSW were estimated using robust Poisson regression. As ART eligibility thresholds changed from≤350 to≤500 cells/mm^3^ during the study period, eligibility was determined based on CD4 count and current guidelines at time of study participation.

**Results:**

Between October 2014 and April 2015, 410 FSW participated in study activities. Overall, 261/410 (63.7%) were living with HIV. Prior history of HIV testing and diagnosis were relatively high (>80%), however, self-reported ART coverage among HIV-positive FSW was just 39% (Figure 1).

After adjusting for time since HIV diagnosis, women who had intimate partners and had not disclosed their HIV status to them were over 50% less likely to be on ART than FSW not in relationships (Table 1). Mothers and women with fewer clients per month were also statistically significantly less likely to be on treatment than non-mothers or FSW with more clients in the adjusted analyses.

Among treatment eligible FSW not on ART, 16/61 (26.2%) had previously been initiated but were no longer taking ART.

**Conclusions:**

HIV testing was common among FSW in this setting, and awareness of HIV status was relatively high, however, efforts are needed to improve ART uptake and retention in this population. Though viral suppression data were not available, this likely represents additional fall-out from the care cascade. Disclosure to partners and family appear to be key barriers to treatment uptake. Building HIV disclosure skills and efficacy may help to improve health outcomes for FSW living with HIV and prevent onward transmission.

**Abstract WEAC0106LB–Figure 1:** Engagement of South African FSW in the HIV care cascade.

**Abstract WEAC0106LB–Table 1:** Predictors of ART use among ART-eligible FSW

		Prevalence ratio [95% CI]	p-value	Adjusted prevalence ratio [95% CI]†	p-value
Age (REF≥30 years)	18–29 years	0.78 [0.60–1.02]	0.069	0.87 [0.66–1.16]	0.346
Number of clients past 30 days	0–10	REF	0.074	REF	
	11 or more	1.24 [0.98–1.57]	0.074	1.29 [1.02–1.63]	0.032
Partnership and disclosure	No non-paying intimate partner	REF	–	REF	–
	Disclosed to some or all intimate partners	0.87 [0.69–1.09]	0.219	0.86 [0.69–1.08]	0.188
	Has not disclosed to intimate partners	0.41 [0.19–0.87]	0.021	0.47 [0.23–0.95]	0.036
Mother	No	REF		REF	
	Yes	0.82 [0.62–1.08]	0.156	0.76 [0.58–0.99]	0.047

† Univariate analyses also assessed age, race, education, mobility, violence and depression. The adjusted model includes variables statistically significant at p<0.20 in univariate analyses, including variables listed, age and time since HIV diagnosis.

# Sub article: Community-based adherence clubs improve outcomes for stable antiretroviral therapy patients: findings from Gugulethu, South Africa

**DOI:** 10.7448/IAS.18.5.20407

**Published:** 2015-07-22

**Authors:** Anna Grimsrud, Maia Lesosky, Cathy Kalombo, Linda-Gail Bekker, Landon Myer

**Affiliations:** 1 Division of Epidemiology and Biostatistics, University of Cape Town, Cape Town, South Africa; 2 Department of Medicine, University of Cape Town, Cape Town, South Africa; 3 Gugulethu Community Health Centre, Provincial Government of the Western Cape, Cape Town, South Africa; 4 The Desmond Tutu HIV Centre, University of Cape Town, Cape Town, South Africa

## Abstract

**Introduction:**

There are few data on patient outcomes from community-based models to deliver antiretroviral therapy (ART), with previous research focused on models for home-based delivery. We describe outcomes of ART patients decentralized to community-based adherence clubs (CACs) and compare outcomes with patients managed within a facility-based model.

**Methods:**

This analysis included 8150 adults initiating ART from 2002–2012 at a public sector clinic in Gugulethu, South Africa, followed until the end of 2013. From June 2012, stable patients (ART>12 months, suppressed viral load) were referred to CACs. Kaplan-Meier methods estimated time to outcomes among CACs stratified by gender and age (youth: 15–24 years of age and older patients: >25 years of age). Long-term follow-up (LTFU) was compared between CACs and facility-based care using proportional hazards models with time-varying covariates and inverse probability weights of CAC participation.

**Results:**

Of the 2113 patients (68.8% female, 7.4% youth) decentralized to a CAC, 94% were retained on ART after 12-months. After the first CAC visit, LTFU among CAC patients was 5.6 and 6.4% at 12-months (Figure 1a) and viral rebound 2.2 and 1.5% (Figure 1c), for men and women, respectively. LTFU was higher in CACs among youth compared to older patients (Figure 1b). Youth were twice as likely to be LTFU ((adjusted hazard ratio) aHR: 2.17, 95% CI 1.26–3.73) and experience viral rebound (aHR 2.24, 95% CI 1.00–5.04) in a CAC compared to older patients. Overall, CAC participation reduced LTFU by 67% (aHR: 0.33, 95% CI 0.27–0.40) compared to facility-based care, and this reduction persisted when stratified by patient demographic and clinic characteristics. Patients initiating ART most recently, in 2010 or 2011, had a 90% reduction in LTFU in a CAC compared to facility-based care (95% CI 0.05–0.21). Youth were the only sub-set of patients that did not have a significant decrease in risk of LTFU in CACs compared to the community health centre (CHC) (aHR 0.68, 95% CI 0.37–1.22).

**Conclusions:**

Community-based Adherence Clubs appear to be associated with a decreased risk of LTFU compared to facility-based care. More research is needed on how to expand the role of community-based ART services and what components of these delivery models support long-term retention.

**Abstract WEAD0101–Figure 1:** Kaplan-Meier plots over the first 18-months in a Community-based Adherance Club: (a) LTFU by gender, (b) LFTU by age, (c) Viral rebound by gender, (d) Viral rebound by age.

# Sub article: Sustained viral suppression in persons living with HIV/AIDS receiving HAART in Peru

**DOI:** 10.7448/IAS.18.5.20408

**Published:** 2015-07-22

**Authors:** Patricia Caballero, Cosme Marcelo Da Silva, Marly Da Cruz

**Affiliations:** 1 Department of Pathology, Universidad Nacional Mayor de San Marcos, Instituto de Medicina Tropical, Lima, Peru; 2 Instituto Nacional de Salud, Lima, Peru; 3 Infectious Diseases, Department of Epidemiology and Quantitative Methods, Fiocruz, ENSP Sergio Arouca, Rio de Janeiro, Brazil; 4 Department of Endemias Samuel Pessoa, Fiocruz, ENSP Sergio Arouca, Rio de Janeiro, Brazil

## Abstract

**Introduction:**

Successful treatment for HIV infection requires sustained viral suppression (SVS). Patients with undetectable HIV-RNA levels have a significantly lower risk of clinical disease progression. And at community level viral suppression is important to reduce HIV transmission and the emergence of resistant strains. The study aimed to analyze the frequency and duration of viral suppression (VS) in the first cohort of people living with HIV/AIDS (PLWHA) under treatment.

**Methods:**

We retrospectively evaluated data from all PLWHA uninsured adults who initiated HAART through the National Program during 2004–2006 and followed-up until 2012. Patients with complete records in the National Laboratory Reporting System Data Base were included. The duration of VS was analyzed using survival analysis (Kaplan-Meier) in PLWHA who achieved viral suppression. Survival time was measured between the first control with viral load ≤400 copies/ml until the presence of first interruption or failure of viral suppression (FSV) with viral load>400 copies/ml. Persons lost to follow up and those without FSV were censored. R Software 3.0.3.was used.

**Results:**

During the study period a total of 6289 PLWHA had access to health care settings for initial evaluation and only 5142 received HAART. Of these, 4530(88%) achieved VS for variable time (responders) and 612 never presented VS (non-responders). Cumulative survival rate was analyzed in responders: 91.1% maintained VS up to one year, 84.6% up to two years, 80.2% to three years, 77.1% to four years, 74.1% to five years and 70.1% to six years. According to survival analysis, Kaplan-Meier curves presented lower duration of VS in young adult patients, females, persons in prisons and those who did not increase their CD4 above baseline. No differences were observed with baseline CD4 and viral load (p<0.05).

**Conclusions:**

This findings suggest that SVS as a programme indicator is feasible and useful for monitoring health care settings and ranking them like a control quality measure. SVS could also be included as another parameter in cascade of treatment measures.

# Sub article: Entry into care following universal home-based HIV testing in rural KwaZulu-Natal, South Africa: the ANRS TasP 12249 cluster-randomized trial

**DOI:** 10.7448/IAS.18.5.20409

**Published:** 2015-07-22

**Authors:** Melanie Plazy, Kamal ElFarouki, Collins Iwuji, Nonhlanhla Okesola, Joanna Orne-Gliemann, Joseph Larmarange, Marie-Louise Newell, Deenan Pillay, François Dabis, Rosemary Dray-Spira, for the ANRS 12249 TasP Study Group

**Affiliations:** 1 INSERM U 897 – Centre Inserm Epidémiologie et Biostatistique, Bordeaux, France; 2 Université Bordeaux, Institut de Santé Publique, d'Epidémiologie et de Développement (ISPED), Bordeaux, France; 3 INSERM, UMR_S 1136, Pierre Louis Institute of Epidemiology and Public Health, Team of Research in Social Epidemiology, Paris, France; 4 Sorbonne Université, UPMC Univ Paris 06, UMR_S 1136, Pierre Louis Institute of Epidemiology and Public Health, Team of Research in Social Epidemiology, Paris, France; 5 Wellcome Trust Africa Centre for Health and Population Studies, Mtubatuba, South Africa; 6 Department of Infection and Population Health, University College London, London, United Kingdom; 7 Ceped (UMR 196 Paris Descartes IRD), IRD, Paris, France; 8 Faculty of Medicine, University of Southampton, Southampton, United Kingdom; 9 Division of Infection and Immunity, University College London, London, United Kingdom

## Abstract

**Introduction:**

In a Universal Test and Treat (UTT) strategy, entry into care soon after HIV diagnosis is crucial to achieve optimal population-antiretroviral treatment (ART) coverage. We evaluated the rate of, and factors associated with, entry into care following home-based HIV testing in a cluster-randomized trial of the effect of immediate ART on HIV incidence in rural KwaZulu-Natal, South Africa.

**Methods:**

From March 2012 to May 2014, individuals ≥16 years in ten (2×5) clusters were offered home-based HIV testing; those ascertained HIV-positive were referred to TasP trial clinics and were offered universal and immediate ART (intervention clusters) or according to national guidelines (control clusters). Entry into care was defined as attending a TasP clinic within three months of referral among adults not actively in HIV care (no visit to local HIV programme within past 13 months). Associated factors were identified separately by sex, using multivariable logistic regression.

**Results:**

Overall, 1205 adults (72.6% women) not actively in HIV care were referred to a TasP clinic. Of these, 405 (33.6%) attended a TasP clinic within three months (no difference between trial arms): 32.5% of women, 36.7% of men. Participants who ever visited the local HIV programme (n=360) were more likely to enter into care than those who didn't (women: adjusted odd-ratio (aOR) 1.76, 95% Confidence Interval (1.26–2.45); men: 2.07 (1.18–3.64)). In women (n=875), those less likely to attend a TasP clinic within three months had completed some secondary school (0.51 (0.33–0.79)) or at least secondary school (0.47 (0.29–0.76)) versus below primary school; were living 1–2 km from a TasP clinic (0.43 (0.30–0.62)) or 2–5 km (0.40 (0.27–0.61)) versus <1 km; didn't know anyone HIV+ within their family (0.60 (0.43–0.81)) and didn't agree that it is good to initiate ART as soon as possible if infected (0.47 (0.26–0.85)); among men (n=330), none of the factors examined was significantly associated with entry into care.

**Conclusions:**

Only one-third of HIV-positive adults referred after home-based HIV testing entered into care within three months in this rural South African community with a 30% HIV prevalence. Innovative interventions should be considered to ensure the success of a UTT strategy.

# Sub article: Assessing the HIV care continuum in The Caribbean, Central and South America network for HIV epidemiology (CCASAnet): progress in clinical retention, cART use and viral suppression

**DOI:** 10.7448/IAS.18.5.20410

**Published:** 2015-07-22

**Authors:** Peter F Rebeiro, Carina Cesar, Bryan E Shepherd, Raquel B De Boni, Claudia Cortés, Fernanda Rodriguez, Pablo Belaunzarán-Zamudio, Jean W Pape, Denis Padgett, Daniel Hoces, Catherine C McGowan, Pedro Cahn

**Affiliations:** 1 School of Medicine, Vanderbilt University, Nashville, United States; 2 Fundación Huésped, Buenos Aires, Argentina; 3 Instituto Nacional de Infectologia Evando ChagasFundação Oswaldo Cruz, Rio de Janeiro, Brazil; 4 Fundación Arriarán, Universidad de Chile, Santiago, Chile; 5 Instituto Nacional de Ciencias Médicas y Nutrición Salvador Zubirán, Mexico City, Mexico; 6 Le Groupe Haïtien d'Etude du Sarcome de Kaposi et des Infections Opportunistes in Port-au-Prince (GHESKIO), Port-au-Prince, Haiti; 7 Instituto Hondureño de Seguridad Social and Hospital Escuela, Tegucigalpa, Honduras; 8 Instituto de Medicina Tropical Alexander von Humboldt, Universidad Peruana Cayetano Heredia, Lima, Peru

## Abstract

**Introduction:**

Retention, combination antiretroviral therapy (cART) use and viral suppression are key stages in the HIV Care Continuum associated with delayed disease progression and reduced transmission. We assessed trends in these indicators within the large and diverse CCASAnet cohort over a decade.

**Methods:**

Adults from CCASAnet clinical cohorts in Argentina, Brazil, Chile, Haiti, Honduras, Mexico and Peru contributed data from first visit between 2003 and 2012 until final visit, death, or the end of 2012. Retention was ≥2 HIV care visits in a year, >90 days apart. cART use was prescription of a regimen of ≥3 active antiretroviral agents in a year. Viral suppression was HIV-1 RNA<200 copies/mL at last measurement in the year. cART use and viral suppression denominators were subjects with ≥1 visit in the year. Multivariable modified Poisson regression models were used to assess temporal trends and predict percentages meeting each indicator in each year, adjusting for age, sex, HIV transmission mode, cohort, calendar year and total time in care.

**Results:**

Among 18,799 individuals contributing to retention analyses, 14,380 to cART use analyses and 13,330 to viral suppression analyses, there were differences between those meeting indicator definitions versus not by most characteristics (Table 1).

There were significant improvements in the indicators from 2003 to 2012: from 63 to 80% retained, 74 to 91% using cART, and 53 to 82% virally suppressed (p<0.05, each). Predicted values from adjusted models revealed similar trends (Figure 1).

Female sex (risk ratio (RR)=0.96; 95% confidence interval (CI): 0.93, 0.99 vs. males) and injection drug use (IDU) as HIV transmission mode (RR=0.84; 95% CI: 0.74, 0.94 vs. male sexual contact with males (MSM)) were associated with lower retention, but unrelated with cART use or viral suppression. MSM transmission (RR=0.96; 95% CI: 0.92, 0.99) decreased probability of cART use versus heterosexual transmission.

**Conclusions:**

HIV Care Continuum outcomes have improved over time. However, efforts must be made to improve retention, particularly among females and IDUs, and cART use must be improved among MSM. Additional research is needed to sustain progress by identifying impediments to achieving positive Care Continuum outcomes, and their causes, in these settings.

**Abstract WEAD0104–Table 1:** Person-years contributed and characteristics

Characteristic	Not Retained^a^	Retained^a^	p*	Not on cART^b^	On cART^b^	p*	Not virally suppressed^c^	Virally suppressed^c^	p*
Total	22,386	67,171	<0.01	11,565	57,312	<0.01	19,369	41,271	<0.01
Age (years)	33.9 (28.2, 40.6)	36.4 (30.0, 43.9)	<0.01	32.5 (27.1, 39.3)	35.5 (29.6, 42.4)	<0.01	33.5 (27.7, 40.4)	36.0 (30.1, 42.9)	<0.01
Male sex	14,238 (25.1)	42,487 (74.9)	0.35	8119 (16.5)	40,982 (83.5)	<0.01	13,493 (31.0)	29,981 (69.0)	<0.01
Female sex	8148 (24.8)	24,684 (75.2)		3446 (17.4)	16,330 (82.6)		5876 (34.2)	11,290 (65.8)	
MSM HIV risk	7050 (27.6)	18,503 (72.4)	<0.01	5079 (18.6)	22,225 (81.4)	<0.01	7537 (31.4)	16,489 (68.6)	<0.01
IDU HIV risk	820 (52.7)	735 (47.3)		203 (15.1)	1141 (84.9)		349 (29.3)	842 (70.7)	
Hetero HIV risk	8443 (29.2)	20,495 (70.8)		4800 (16.1)	24,945 (83.9)		8921 (34.4)	17,044 (65.6)	
Other/unk. HIV risk	6073 (18.1)	27,438 (81.9)		1483 (14.2)	9001 (85.9)		2562 (27.1)	6896 (72.9)	
Individual years in care	7 (4, 9)	7 (4, 9)	<0.01	6 (3, 8)	8 (5, 10)	<0.01	6 (4, 9)	8 (5, 10)	<0.01

**Abstract WEAD0104–Figure 1:** Figure in HIV care continuum outcomes in CCASAnet.

# Sub article: Providing same day, observed ART to newly diagnosed HIV+ outpatients is associated with improved virologic suppression

**DOI:** 10.7448/IAS.18.5.20557

**Published:** 2015-07-22

**Authors:** Christopher Pilcher, Hiroyu Hatano, Aditi Dasgupta, Diane Jones, Sandra Torres, Fabiola Calderon, Clarissa Ospina-Norvell, Wendy Hartogensis, Erin Demicco, Elvin Geng, Monica Gandhi, Diane Havlir

**Affiliations:** 1 HIV/AIDS Division, Medicine, UCSF, San Francisco, United States; 2 Tulane University Medical School, New Orleans, United States

## Abstract

**Introduction:**

Despite known clinical and prevention benefits, ART is typically delayed by weeks-to-months after HIV diagnosis to allow linkage to care, HIV education, social stabilization and laboratory evaluation. The UCSF/San Francisco General Hospital (SFGH) RAPID programme aimed to eliminate this delay by providing same-day/observed ART even as HIV care was being established. We investigated consequences of the RAPID treatment initiation strategy.

**Methods:**

RAPID eligibility included new HIV diagnosis with acute/recent infection, active opportunistic infection or CD4 <200/mm^3^. At referral, all RAPID-eligible or -ineligible patients with new diagnosis received a standard package of multidisciplinary services for social support, education, risk and stigma reduction; labs were drawn; and regular provider follow-up was arranged. The RAPID intervention consisted of 1) same-day access to an on-call provider; 2) a five-day ART supply facilitated by and 3) an accelerated process for insurance benefits. Focusing on a July 2013–December 2014 programme period, survival analysis was used to compare time to achieving viral load (VL) <200 copies/mL between patients receiving and not receiving the RAPID intervention, and also between these patients and historical controls from two eras of ART provision at SFGH: pre-RAPID universal (2010–2013) and CD4-guided (2006–2009).

**Results:**

We studied 227 newly diagnosed outpatients receiving RAPID (n=39), universal (n=149) or CD4-guided (n=39) ART. No patients had private insurance and 27% were homeless; mean (range) CD4 was 381(2–1031)/mm^3^ and VL 4.6 (1.6–7.0) log_10_ cp/mL. Time to VL <200 cp/mL was significantly faster in RAPID patients versus both contemporaneous and historical controls (p<0.001; see Figure).

Median (IQR) time to VL <200 for RAPID ART was 56(40–87) days versus 119(58–201) days for universal and 283(128–777) days for CD4-guided ART. After three months of ART, 75% RAPID versus 38% non-RAPID patients achieved a VL <200 cp/mL; after six months, 95% RAPID versus 70% non-RAPID patients achieved VL <200 cp/mL. Among the first 39 patients receiving RAPID ART and followed for 5–18 months, only two (5%) had toxicity-related regimen changes, none discontinued ART and 35 (90%) remain engaged in care.

**Conclusions:**

Combined with patient education and psychosocial support, same day-observed initiation of ART at the time of HIV diagnosis was feasible and associated with substantially faster sustained viral suppression.

**Abstract WEAD0105LB–Figure 1:** Viral suppression over time by ART initiation strategy.

# Sub article: Targeted HIV testing in home or clinic for older children of HIV-infected adults in care increases paediatric HIV testing rates and reveals high prevalence of previously undiagnosed HIV infection

**DOI:** 10.7448/IAS.18.5.20411

**Published:** 2015-07-22

**Authors:** Anjuli Wagner, Irene Njuguna, Cyrus Mugo, Irene Inwani, Elizabeth Maleche-Obimbo, Kenneth Sherr, Dalton Wamalwa, Grace John-Stewart, Jennifer Slyker

**Affiliations:** 1 Department of Epidemiology, University of Washington, Seattle, United States; 2 Department of Pediatrics and Child Health, University of Nairobi, Nairobi, Kenya; 3 Kenyatta National Hospital, Nairobi, Kenya; 4 Department of Global Health, University of Washington, Seattle, United States; 5 Department of Global Health, University of Washington, Seattle, United States; 6 Department of Pediatrics, University of Washington, Seattle, United States; 7 Department of Medicine, University of Washington, Seattle, United States

## Abstract

**Introduction:**

Health systems offer infant HIV testing as part of prevention of mother-to-child HIV transmission (PMTCT) programs, but are not built to systematically diagnose HIV infection in older children before symptomatic illness. Offering HIV-infected adults attending HIV treatment programs targeted testing in home or clinic may increase early diagnosis of paediatric HIV.

**Methods:**

HIV-infected parents attending HIV care clinic at Kenyatta National Hospital (KNH) in Nairobi, Kenya were asked about their children's HIV status. Adults with untested children≤12 years old chose to test children either at home (HBT) or in a clinic (CBT). Multinomial relative risk regression was used to identify cofactors of testing acceptance.

**Results:**

During the 9-month period when targeted testing was routinely offered, approximately four times as many children were tested per month as in the previous 10-month period (13.6 vs 3.5 per month, RR: 3.9, 95% CI: 2.8–5.5).

Among 116 enrolled adults, 23 (20%) chose HBT and had 46 children tested, 48 (41%) chose CBT and had 58 children tested, and 45 (39%) did not complete testing. More adults chose CBT than HBT (p=0.003), but more children were tested per adult by HBT (2.0 vs. 1.2, p<0.001). HIV prevalence among 104 tested children was 8% overall; six infected children were identified by CBT and two by HBT (median age: 8 years (IQR: 2–11)).

Compared to adults who chose CBT, adults who chose HBT were more likely to have higher income, more education, be male, have a partner, have an unemployed partner and have a partner known to be HIV negative (p<0.05), while adults who did not test their children were more likely to have higher income and have a partner who was known to be HIV negative or of unknown HIV status (p<0.05). In multivariate analyses, income and partner status remained significantly associated with testing choice.

**Conclusions:**

Targeting HIV-infected parents in care increased the rate of paediatric testing and found high prevalence of paediatric HIV. CBT was preferred over HBT at this urban referral hospital. Efforts to increase paediatric HIV testing and to understand parental characteristics are important to provide timely diagnosis and linkage to care.

**Abstract WEAD0201–Figure 1:** Active referral increases paediatric HIV testing.

# Sub article: Moving towards targeted HIV testing in older children at risk of vertically transmitted HIV

**DOI:** 10.7448/IAS.18.5.20412

**Published:** 2015-07-22

**Authors:** Tsitsi Bandason, Grace Mchugh, Rashida Ferrand, Shungu Munyati, Katharina Kranzer, Prosper Chonzi

**Affiliations:** 1 Biomedical Research and Training Institute, ZENITH, Harare, Zimbabwe; 2 Biomedical Research and Training Institute, Harare, Zimbabwe; 3 London School of Hygiene and Tropical Medicine, London, United Kingdom; 4 Harare City Health, Harare, Zimbabwe

## Abstract

**Introduction:**

WHO recommends PITC to all in high-burden countries. Symptom screening algorithms have been used widely for other diseases like tuberculosis. Prompt identification of undiagnosed HIV infection remains a priority in Southern Africa. We previously proposed a simple algorithm where a child is asked to respond to any of the four questions, namely, whether child

1) has previously been admitted to hospital,

2) has had recurring skin problems,

3) is a single or double orphan

4) has experienced poor health in the past three months which can be asked by any cadre at primary care level for screening older children at risk of HIV infection and requiring an HIV test. The objective of this study was to validate the performance of this algorithm in a primary care setting.

**Methods:**

All previously untested children, aged 6–15 years attending seven selected Primary Health Care Clinics of Harare, Zimbabwe, with parental/guardian consent were tested for HIV infection and asked to respond to four algorithm questions. Each positive response was scored as one.

**Results:**

A total of 6102 (74%) children with median age 9 (IQR: 7 to 11) years, 3138 (51%) of them male, consented to an HIV test. HIV prevalence was 4.8% (95% CI: 4.2–5.3) and positivity increased successively as the score increased with those who scored zero, 55/3830 (1%); scored one, 110/1609 (7%); scored two, 80/489(16%); scored three, 26/96 (27%); scored four, 10/16(63%).

A child with a score of one or more had eight times odds (95% CI: 6–11) of testing HIV positive with a sensitivity of 80% (95% CI: 75–85), specificity of 66% (95% CI: 64–67). Sensitivity was higher in those aged 10 years or more (86% vs. 70%, p=0.001). Overall, we needed to test 11 children to identify one HIV positive.

**Conclusions:**

The algorithm maintained its integrity and demonstrated that it is a sensitive tool screening older children at risk of HIV infection. The algorithm can be used by lower cadre healthcare workers and can help prioritize limited resources.

# Sub article: Impact of implementing “Test and Treat” policy on paediatric ART enrolments and coverage in Uganda

**DOI:** 10.7448/IAS.18.5.20413

**Published:** 2015-07-22

**Authors:** Peter Elyanu, Eleanor Magongo, Barbara Asire, Ivan Lukabwe, Harriet Bitimwine, Cordelia Katureebe, Pamela Achii, Vivienne Mulema, Eric Dziuban, Nandita Sugandhi, Victor Musiime, Nora Namuwenge, Joshua Musinguzi

**Affiliations:** 1 STD/AIDS Control Program, Ministry of Health, Kampala, Uganda; 2 School of Public Health, University of Texas, Houston, United States; 3 Baylor College of Medicine Children's Foundation-Uganda, Kampala, Uganda; 4 Pharmacy Division, Ministry of Health/Management Sciences for Health, Kampala, Uganda; 5 Clinton Health Access Initiative (CHAI), Kampala, Uganda; 6 CDC, Atlanta, Uganda; 7 Clinton Health Access Initiative (CHAI), Boston, United States; 8 Makerere University College of Health Sciences, Pediatrics and Child Health, Kampala, Uganda

## Abstract

**Introduction:**

In 2013, it was estimated that 193,500 of children under 15 years were living with HIV in Uganda and 83% would be eligible for treatment according to WHO guidelines; recommending lifelong treatment for all children under five years and all older patient based on clinical or immunologic staging. However, despite efforts to scale up paediatric treatment, coverage remained low at 22% in 2013. Programmatic barriers to ART initiation in children include the perception that paediatric ART is complicated, unavailability of CD4 testing and difficulty in accurate clinical staging. In September 2013, Uganda adopted a “test and treat” antiretroviral therapy (ART) policy for all HIV infected children under 15 years of age to simplify recommendations and remove programmatic barriers to ART initiation in children.

**Methods:**

The MOH launched and disseminated these guidelines to all stakeholders though three day health facility based trainings and mentoring during the period January to December 2014. To evaluate the impact of this new policy a comparison was made between the number of children initiated between June and December 2013 and those initiated between January and June 2014.

**Results:**

By December 2014, 1340 (84%) of 1600 ART providing health facilities and 17,238 health workers were trained on the new guidelines. There was 1.4-fold increase in the number of HIV infected children newly initiated on ART from 5540 in June–Dec 2013 to 9145 in Jan–June 2014. The increase was greater among children aged 5–14 years and 2–4 years (2.4 and 1.4 fold, respectively); however, there was no change among the under two year old's ( see Figure 1). Pregnant adolescents constituted 2.5% (229/9145) of children less than 15 years of age enrolled on ART in Jan–June 2014. Paediatric ART coverage has increased from 22% (43,481/193,500) in December 2013 to 27% (51,305/193,500) in June 2014.

**Conclusions:**

Expanding eligibility criteria increases initiation of older children on ART but to enrol those who are at higher risk of disease progression/mortality, more work needs to be done to improve early infant diagnosis (EID) and early case detection.

**Abstract WEAD0203–Figure 1:** Children 0–14 years newly initiated on Antiretroviral therapy (July 2013 to June 2014).

# Sub article: Immunization practice and vaccine safety perception in centres caring for children with prenatally acquired HIV: results from the Pediatric European Network for Treatment of AIDS survey

**DOI:** 10.7448/IAS.18.5.20414

**Published:** 2015-07-22

**Authors:** Emma Concetta Manno, Alasdair Bamford, Pablo Rojo Conejo, Laura Marquez, Maria Jose Mellado, M Ángeles Muñoz-Fernández, Vana Spoulou, Nigel Klein, Jintanat Ananworanich, Marinella Della Negra, John Bernard Ziegler, Esse Meanson, Hermione Lyall, Delane Shingadia, Antonio Di Biagio, Vania Giacomet, Rosenfeldt Vibeke, Heloisa Helena de Sousa Marques, Eeva Salo, Alla Volokha, Henriette J Scherpbier, Tim Niehues, Jack Levy, Magdalena Marczynska, Mariana Mardarescu, Veronique Reliquet, Carlo Giaquinto, Stefania Bernardi, Paolo Palma

**Affiliations:** 1 Unit of Immune and Infectious Diseases, DPUO, University Department of Pediatrics, Children's Hospital “Bambino Gesu”, Rome, Italy; 2 Department of Paediatric Infectious Diseases and Immunology, Evelina London Children's Hospital, Guy's and St. Thomas’ NHS Foundation Trust, London, United Kingdom; 3 Hospital Universitario Doce de Octubre, Unidad de Inmunodeficiencias paediatrics, Servicio de Pediatría, Madrid, Spain; 4 Centro Hospitalar do Porto, Pediatric Infectious Diseases and Immunodeficiencies Unit, Porto, Portugal; 5 Hospital Universitario Infantil La Paz. Madrid, Servicio de Pediatría y Enfermedades Infecciosas y Tropicales, Madrid, Spain; 6 Hospital General Universitario Gregorio Marañón, Laboratorio InmunoBiología Molecular, Madrid, Spain; 7 Department of Infectious Diseases, ‘Aghia Sophia’ Children's Hospital, Athens, Greece; 8 Infectious Diseases and Microbiology Unit, Institute of Child Health, University College London, London, United Kingdom; 9 U.S. Military HIV Research Program, Walter Reed Army Institute of Research, Silver Spring, United States; 10 Henry M. Jackson Foundation for the Advancement of Military Medicine, Bethesda, United States; 11 Instituto de Infectologia Emílio Ribas, São Paulo, Brazil; 12 Department of Immunology and Infectious Diseases, Sydney Children's Hospital, Sydney, Australia; 13 Department of Paediatric Infectious Disease and Immunology, Evelina London Children's Hospital, Guy's and St. Thomas’ NHS Foundation Trust, London, United Kingdom; 14 Imperial College Healthcare NHS Trust, London, United Kingdom; 15 Great Ormond Street Hospital for Children NHS Foundation Trust, London, United Kingdom; 16 Infectious Diseases Unit, IRCCS San Martino University Hospital-IST, University of Genoa, Genoa, Italy; 17 Department of Paediatrics, L Sacco University Hospital, Milan, Italy; 18 Department of Paediatrics, Hvidovre University Hospital, Copenhagen, Denmark; 19 Instituto da Criança, Hospital das Clínicas, Faculdade de Medicina, Universidade de São Paulo, São Paulo, Brazil; 20 Children's Hospital, Helsinki University Central Hospital and University of Helsinki, Helsinki, Finland; 21 Children Center of Clinical Immunology, Kiev, Ukraine; 22 Department of Pediatric Haematology, Immunology and Infectious Diseases, Academic Medical Center, Emma Children's Hospital, Amsterdam, The Netherlands; 23 Department of Pediatrics, Helios Clinic Krefeld, Krefeld, Germany; 24 Saint-Pierre University Hospital, Brussels, Belgium; 25 Pediatric Department of Infectious Diseases, Medical University of Warsaw, Warszawa, Poland; 26 Department of Paediatric Immunodepression, Prof. Dr. Matei Bal, National Institute for Infectious Diseases, Bucharest, Romania; 27 Department of Infectious Diseases, CHU Nantes, Nantes, France; 28 Department of Paediatrics, University of Padova, Padova, Italy

## Abstract

**Introduction:**

Perinatally HIV-infected children are more susceptible to vaccine preventable infections and vaccine induced immunity is less robust than in healthy children because of precocious waning of protective immunity. For this high risk population it is important to design specific vaccine schedules to define correct dosing and to set accurate correlates of protection. This survey was performed to give an overview of current vaccinations practice among paediatricians looking after vertically HIV-infected children.

**Methods:**

An online questionnaire regarding vaccination practices in HIV-infected children was completed by investigators from the PENTA network. Data were collected between November 2013 and March 2014.

**Results:**

A total of 88 experts in the management of paediatric HIV-infection from 46 different units looking after 2465 patients completed the questionnaire. The majority of units (72%) did not perform routine childhood immunizations in HIV centres. Vaccination histories were incomplete for 40% of the studied population. Influenza, pneumococcal conjugate vaccine and human papilloma vaccine immunizations are widely administered (93, 89 and 83% of units, respectively). Varicella and Rotavirus vaccinations are less recommended (61 and 24% of the units, respectively). Monitoring of vaccine responses is employed in 72% of centres. Serology appears to be the most feasible assay among the different centres (90%), mostly performed with immune-enzymatic assays.

**Conclusions:**

Vaccination practices for perinatally HIV-infected children still vary widely between countries. A crucial issue is the incomplete adherence to varicella vaccine. Indeed only in few countries varicella vaccination is universally recommended for children at national. More efforts should be made to standardize mandatory and recommended vaccinations, as well as to guide timing of serological assays. The majority of units carry out immuno-enzymatic tests to evaluate specific antibody levels. However, methods vary with different cut-offs of protection and units of measurement employed. Moreover, especially in high risk groups (e.g. children who started late HAART or performed vaccinations before treatment), researches on the development of novel methods to assess protective immunity and accurate correlates of protection are needed. The ultimate goal will be to design individualized vaccine schedules, developed on therapeutic and immunological features of individual patients, optimizing the chances of them gaining robust long-term vaccine induced protection.

# Sub article: Lower ANC attendance and PMTCT uptake in adolescent versus adult pregnant women in Kenya

**DOI:** 10.7448/IAS.18.5.20558

**Published:** 2015-07-22

**Authors:** Keshet Ronen, Christine Mcgrath, Agnes Langat, John Kinuthia, Danvers Omolo, Benson Singa, Abraham Katana, Lucy Nganga, Grace John-Stewart

**Affiliations:** 1 Fred Hutchinson Cancer Research Center, Seattle, United States; 2 University of Washington, Seattle, United States; 3 University of Texas Medical Branch, Galveston, United States; 4 Centers for Disease Control and Prevention, Nairobi, Kenya; 5 Kenyatta National Hospital, Nairobi, Kenya; 6 Kenya Medical Research Institute, Nairobi, Kenya

## Abstract

**Introduction:**

Although rates of pregnancy and HIV infection are high among Kenyan adolescent women, their engagement in Prevention of Mother-to-Child HIV Transmission (PMTCT) services is poorly characterized. We hypothesized that adolescent women show lower engagement in the PMTCT cascade than adult women, from antenatal care (ANC) attendance to HIV testing and antiretroviral (ARV) uptake.

**Methods:**

We conducted a nationally representative cross-sectional survey of mothers attending 120 maternal child health clinics selected by probability-proportionate-to-size-sampling in Kenya in July–December 2013, with a secondary survey oversampling HIV-positive mothers in 30 clinics. Self-report questionnaires verified by clinic booklets recorded ANC attendance, HIV testing, ARV use and maternal characteristics. Data were compared between adolescent (age<20) and adult mothers. Differences in maternal characteristics were assessed by Chi-square test. Logistic regression was used to analyze ANC attendance and HIV testing among all women and ARV uptake among HIV-positive women.

**Results:**

Among 2521 mothers surveyed, 278 (12.8%) were adolescents. Adolescents were less likely than adults to have above primary education (25.0% vs. 42.9%, p<0.001), intended pregnancy (40.5% vs. 58.6%, p<0.001) and a current partner (73.1% vs. 90.9%, p< 0.001). Overall, 2471 (97.8%) reported attending ≥1 ANC visit. Among 1859 women with verified ANC visits, 898 (44.7%) attended ≥4 visits. Adolescents were less likely than adults to attend ≥4 ANC visits (35.2% vs. 45.6%, OR[95% CI]=0.65 [0.49–0.86]). This effect remained significant when adjusting for education, primigravida, pregnancy intention and HIV status (OR[95% CI]=0.59 [0.36–0.97]). Among 2359 women who attended ≥1 ANC visit and were not known to be HIV-positive prior to pregnancy, 2298 (96.1%) received HIV testing during pregnancy. Testing rates were not significantly different between adolescents and adults. Among 288 HIV-positive women who attended ≥1 ANC visit and were not on HAART prior to pregnancy, 20 (6.9%) were adolescents, and 243 (84.4%) used any ARVs for PMTCT. Adolescents were less likely to use ARVs than adults (65.0% vs. 85.8%, OR[95% CI]=0.31 [0.12–0.81]).

**Conclusions:**

Adolescent mothers showed poorer ANC attendance and lower uptake of ARVs for PMTCT. This calls for further study on barriers to ANC and PMTCT services among adolescent women and development of targeted interventions to improve uptake and retention of this vulnerable population through the PMTCT cascade.

# Sub article: Health resource use pattern analysis to inform targeted interventions alongside the HIV cascade of care and optimize the effect of treatment as prevention

**DOI:** 10.7448/IAS.18.5.20415

**Published:** 2015-07-22

**Authors:** Emanuel Krebs, Jeong Eun Min, Rolando Barrios, Julio Montaner, Bohdan Nosyk, STOP HIV/AIDS Study Group

**Affiliations:** 1 BC Centre for Excellence in HIV/AIDS, Vancouver, Canada; 2 Division of Aids, Faculty of Medicine, University of British Columbia, Vancouver, Canada; 3 Faculty of Health Sciences, Simon Fraser University, Burnaby, Canada

## Abstract

**Introduction:**

Identifying patterns of health resource utilization (HRU) of people living with HIV/AIDS (PLHIV) can allow for comparison of their effects on longer-term health outcomes and costs. Further, identification of patterns associated with greater risk of attrition between stages of the cascade of care can help in the development of targeted interventions to effectively increase patient retention.

**Methods:**

We conducted a population-level analysis of HRU for individuals having received a CD4 test after HIV diagnosis. All individuals 18 years or older in British-Columbia in the modern HAART-era (post-September 2006) were included. Using linked comprehensive administrative health databases in a probabilistic model-based clustering analysis with 14 HRU measures, we estimated parameters by maximum likelihood using the expectation maximization (EM) algorithm. Individuals with estimated parameters maximizing the probability of belonging to a similar HRU cluster were classified with each other, and the optimal number of clusters was estimated by the Bayesian Information Criterion. The analysis was conducted across CD4 count stratification (>200 cells/mm^3^; <200 cells/mm^3^).

**Results:**

Our study included 941 individuals with at least one year follow-up (median age 40, 21% female) and with a CD4 count obtained between September 1st, 2006 and March 31st, 2011. Individuals with CD4<200 clustered in two HRU patterns. The high cost cluster (N=68; mean $18,169(SD$21,432)), driven by lengthy HIV-related emergency hospitalization stays (76.5% with>7 days), had costs more than double the low cost cluster (N=147; $6811($13,592)). Individuals with CD4>200 were best classified in four clusters. The high cost cluster (N=74; $15,831($19,180)) was characterized by non-HIV ER hospitalizations (100%≥1 day, 55.4%>7 days) and high prevalence of mental health issues. The second highest cost cluster (N=60; $5058($5152)) was characterized by short-term non-HIV elective hospitalizations (48.3%=1 day). The two lower cost clusters both had no hospitalizations; the higher (N=425; $3378($6454)) with much more frequent physician visits and medication use than the lowest cost cluster (N=167; $1291($7969)).

**Conclusions:**

Even within relatively homogeneous cohorts in terms of disease progress at time of linkage to HIV care, individuals were found to have heterogeneous HRU patterns. Identifying classes of individuals according to HRU can help inform clinical response, as well as the design of public health interventions to optimize HIV care.

# Sub article: Optimizing HIV/AIDS resources in Armenia: increasing ART investment and examining seasonal labour migrant programs

**DOI:** 10.7448/IAS.18.5.20416

**Published:** 2015-07-22

**Authors:** Sherrie Kelly, Andrew Shattock, Cliff Kerr, Robyn Stuart, Arshak Papoyan, Trdat Grigoryan, Ruben Hovhannisyan, Samvel Grigoryan, David Wilson

**Affiliations:** 1 The Kirby Institute, UNSW, Sydney Australia; 2 School of Physics, University of Sydney, Sydney, Australia; 3 Department of Mathematical SciencesUniversity of Copenhagen, Copenhagen, Denmark; 4 National Center for AIDS Prevention, Yerevan, Armenia

## Abstract

**Background:**

HIV prevalence is declining in all key affected populations in Armenia (people who inject drugs, men who have sex with men, prisoners and female sex workers); however, there are increases among labour groups who seasonally migrate to countries of higher HIV prevalence. We conducted a modelling study to assess the impact of optimizing the national strategic plan to minimize HIV incidence and AIDS-related deaths by 2020. We determined optimal funding levels for all programs to best achieve the strategic plan and, in particular, examined the outcomes required for migrant programs to warrant increased funding.

**Methods:**

We used the Optima model to perform epidemiological and economic analyses. Demographic, epidemiological, behavioural and HIV programme cost data were obtained for Armenia from 2000 to 2014 and used to inform the model. Through internal and external consultations, assumptions were generated on what coverage levels among targeted populations could be attained for different investments, as well as their expected outcomes. A sensitivity analysis on migrant HIV testing and counselling programs was conducted around assumptions based on observed data.

**Results:**

According to Optima's optimization algorithm, shifts in funding allocations are required to minimize incidence and deaths by 2020. The largest emphasis should be on antiretroviral therapy (ART), as optimal allocations nearly doubled the investment in treatment from 17 to 24% of the total budget. This is projected to avert almost 25% of new infections and 50% of AIDS-related deaths by 2020 compared to levels if 2013 spending were maintained. We show that funding for seasonal migrant programs should be maintained through to 2020 at 5% of the total budget. Sensitivity analysis demonstrated that these programs are cost-effective to fund if the coverage threshold for HIV testing and counselling for seasonal migrants, as illustrated in Figure 1B, can be achieved.

**Conclusions:**

Optimization of HIV/AIDS investment in Armenia could significantly reduce HIV incidence and AIDS-related deaths by 2020, particularly by focusing more on antiretroviral therapy. We have also identified thresholds for programme performance, prior to their scale-up, which can be used to evaluate whether they should be scaled-up or down in the future.

Country of research: Armenia

Key Population: People living with HIV (PLHIV), Migrants/displaced persons/mobile populations

**Abstract WEAD0302–Figure 1:** (a) Optimized spending to minimize HIV incidence and AIDS-related deaths by 2020 in Armenia, (b) Sensitivity analysis of cost-coverage for seasonal migrant HIV testing and counselling program in Armenia.

# Sub article: Has performance-based financing accelerated progress towards controlling the HIV epidemic? An impact evaluation of Mozambique's HIV-focused PBF programme

**DOI:** 10.7448/IAS.18.5.20417

**Published:** 2015-07-22

**Authors:** Yogesh Rajkotia, Omer Zang, Pierre Nguimkeu, Iva Djurovic

**Affiliations:** ThinkWell, Maputo, Mozambique

## Abstract

**Introduction:**

As performance-based financing (PBF) gains global traction, evidence around its effectiveness to accelerate the elimination of HIV is needed. We evaluated the impact of a PBF programme implemented by Elizabeth Glaser Pediatric AIDS Foundation (EGPAF), on the provision of HIV, PMTCT and MCH services. We also examined the temporal effects of PBF to better understand its lifecycle both in terms of onset and duration of effect. Finally, we evaluated the impact of PBF on non-incentivized services.

**Methods:**

The impacts of PBF in Gaza (South) and Nampula (North) provinces were analyzed using a retrospective observational study design in which PBF provinces were matched with control provinces Eighteen indicators related to HIV, PMTCT and MCH services were reviewed. Due to regional heterogeneity, we evaluated the North and South as separate experiments. Beginning January 2011, up to eleven quarters of data from 134 PBF facilities after matching (84 North and 50 South) were used. Data sources include PBF programme data and health management information system data. Our econometric framework employed a multi-period, multi-group difference-in-difference model on data that was matched using propensity scoring. The regression design employed a generalized linear mixed model with both fixed and random effects, fitted using the seemingly-unrelated regression (SUR) technique.

**Results:**

PBF resulted in positive impacts on MCH, PMTCT, and paediatric HIV programme outcomes. The majority of the 18 indicators responded to PBF (77% North and 66% South), with at least half of the indicators demonstrating a statistically significant increase in average output of more than 50% relative to baseline. Most adult HIV (excluding pregnant women) initiation and retention indicators did not respond to PBF. On average, it took six quarters of implementation for PBF to take effect, and impact was generally sustained thereafter. Indicators were not sensitive to price, but rather inversely correlated to the level of effort associated with marginal output. No negative impacts on incentivized indicators nor spill-over effects on non-incentivized indicators were observed.

**Conclusions:**

The PBF programme in Mozambique has shown to produce large, sustained increases in the provision of PMTCT, paediatric HIV and MCH and should be considered as a powerful alternative to traditional input-based financing.

# Sub article: The estimated need of second-line antiretroviral therapy in sub-Saharan Africa 2015–2030: mathematical modelling study

**DOI:** 10.7448/IAS.18.5.20418

**Published:** 2015-07-22

**Authors:** Janne Estill, Nathan Ford, Luisa Salazar-Vizcaya, Andreas Haas, Nello Blaser, Matthias Egger, Vincent Habiyambere, Olivia Keiser, IeDEA Southern Africa

**Affiliations:** 1 Institut für Sozial- und Präventivmedizin (ISPM), University of Bern, Bern, Switzerland; 2 World Health Organisation, Geneva, Switzerland

## Abstract

**Introduction:**

At the end of 2013, about 300,000 patients were on second-line antiretroviral therapy (ART) in sub-Saharan Africa. The need for second-line ART may increase substantially with increasing duration of patients on ART and roll-out of viral load monitoring. We aimed to estimate the need of second-line ART in sub-Saharan Africa between 2015 and 2030 under various scenarios.

**Methods:**

We developed a mathematical simulation model of HIV progression on ART to project second-line needs up to 2030 for individual countries. The model allows the user to vary key input parameters, including annual numbers of patients starting ART, delay in switching after detection of treatment failure, possibility of treatment interruptions, background mortality and monitoring strategies. We applied the model to all countries in sub-Saharan Africa assuming 12 scenarios that combine different future ART scale-up scenarios (accelerated until universal coverage; stable; no future scale-up), monitoring (routine viral load monitoring in all or only selected countries), and retention and switching (including or excluding possibility of treatment drop-out and delayed switching). The input parameters were chosen to fit the numbers of patients on first- and second-line ART in 2005–2013 to observed estimates.

**Results:**

If the scale-up of ART is accelerated across the region, patients are retained in care, switching is immediate, and all countries implement routine viral load monitoring, the number of patients on second-line ART will increase to 4.1 million by 2030 (17% of all patients on ART). In a scenario with a stable scale-up and realistic drop-out and switching delay, the corresponding numbers were 2.8 million (15%) with universal routine viral load monitoring, and 2.2 million (12%) with routine viral load monitoring only in selected countries.

**Conclusions:**

We expect that by 2030, 2–3 million people will receive second-line ART in sub-Saharan Africa, but the number of patients in need may be over four million. Routine viral load monitoring, timely switching and minimizing treatment interruptions will further increase the number of patients on second-line ART.

**Abstract WEAD0304–Table 1:** Projected number of patients on first- and second-line ART in 2020 and 2030 under various scenarios

		2020				2030			
			
Future scale-up of ART initiation	Treatment interruptions and switching	Universal routine viral load monitoring		Targeted or routine viral load monitoring depending on country		Universal routine viral load monitoring		Targeted or routine viral load monitoring depending on country	
			
		1st-line	2nd-line	1st-line	2nd-line	1st-line	2nd-line	1st-line	2nd-line
Accelerated scale-up until universal	No interruptions, immediate switching	18,272,800	2,480,100	19,143,300	1,672,400	19,561,700	4,144,300	20,730,300	2,992,200
coverage reached	Interruptions included, delayed switching	18,334,300	1,771,300	18,869,600	1,221,200	20,161,000	3,561,500	21,143,100	2,539,400
Stable scale-up	No interruptions, immediate switching	13,306,000	1,899,200	13,807,300	1,387,100	15,717,400	3,239,100	16,397,400	2,555,600
	Interruptions included, delayed switching	12,598,600	1,445,300	12,970,000	1,056,600	15,892,600	2,758,100	16,462,900	2,166,900
No future scale-up	No interruptions, immediate switching	7,655,600	1,397,200	7,447,200	987,100	7,305,900	1,757,000	7,082,000	1,352,300
	Interruptions included, delayed switching	7,697,300	1,186,700	8,009,900	872,700	7,314,800	1,569,200	7,619,000	1,262,700

**Abstract WEAD0304–Figure 1:** Expected number of patients on first- and second-line ART in four selected, unnamed, sub-Saharan African countries. We assumed universal routine viral load monitoring, stable scale-up of ART initiation, and included treatment interruptions and delay in switching. Curves show the model projections and the points the observed numbers. Black/grey curves and points show the total number of patients, blue curves/points the patients on first-line ART and red/pink curves/points the patients on second-line ART.

# Sub article: Kenya private health sector HIV care services costing using the management accounting system for hospitals framework

**DOI:** 10.7448/IAS.18.5.20419

**Published:** 2015-07-22

**Authors:** Benson Chuma, Sayaka Koseki, Stephen Musau, Ben Johns, Agnes Gatome-Munyua

**Affiliations:** 1 Abt Associates, International Health, Nairobi, Kenya; 2 Abt Associates, Washington, United States; 3 Abt Associates, Washington, United States

## Abstract

**Introduction:**

The private sector is a key HIV service provider in Kenya, but few data on the cost of private service provision are available. The lack of cost data has inhibited the design of reimbursement mechanisms and health insurance packages, as well as policy decisions on private sector financing. This study estimated unit costs for private sector HIV services disaggregated by facility type and level, as a contribution to ongoing efforts to implement health insurance products covering HIV services.

**Methods:**

Cost and service volume data were collected from 149 private sector facilities in 2013 as part of a nationwide systematic sampling of public and private healthcare costing study supported by GIZ, the USAID-funded Strengthening Health Outcomes Through the Private Sector (SHOPS) Project Kenya, and the Ministry of Health. The MASH (Management Accounting System for Hospitals) tool was used to analyze data. Multiple facilities were eliminated due to lack of complete data with only 60 used.

**Results:**

Average unit costs per inpatient day and per outpatient visit were generated by sector and facility levels 2–4 (as defined by Kenya Norms and Standards 2006). HIV specific unit costs estimated included for HIV counselling and testing (HCT) services and provision of ART. The authors estimated operational costs, but were unable to estimate capital costs following lack of data. Average outpatient visits ranged from Ksh. 689 to 1036 in level 2 and level 4, respectively. ART visit costs ranged from Ksh. 1575 to 3660 across the facilities sampled. HCT visit services ranged from Ksh. 537 to 1151 across level 2 and 4 facilities, respectively.

**Conclusions:**

The study contributed to health financing policy discussions in the provision and financing of HIV services in Kenya. Data generated was presented to insurers and providers who expressed intentions of using it for decision making. Possible applications include design of HIV care inclusive insurance products and advising reimbursement decisions regarding the same. Providers offering HIV services can also use it to benchmark their efficiency. Due to poor record keeping in most facilities, only 60 of the 149 facilities had enough data for analysis. There's a need to support facilities to improve record keeping.

# Sub article: The PEPFAR COPs allocation database: a comprehensive database to monitor PEPFAR spending, increase data transparency, and improve civil society engagement in country operational plans

**DOI:** 10.7448/IAS.18.5.20559

**Published:** 2015-07-22

**Authors:** Brian Honermann, Gregorio Millett, Kali Lindsey, Sandhira Wijayaratne, Jennifer Sherwood, Jack MacAllister

**Affiliations:** Public Policy Office, amfAR, Washington, DC, United States

## Abstract

**Introduction:**

A total of $29.5 billion has been allocated by PEPFAR from 2007 to 2014 through the annual country operational plan (COP) process. COPs serve as a planning tool for activities of US government and in-country partners funded by PEPFAR. Historically, utilizing data from COPs has been difficult due to their inflexible PDF/RTF format hindering the ability to query and manipulate data, create graphical representations of the financial data or identify trends in PEPFAR allocations over time. As PEPFAR moves towards greater civil society engagement during COPs’ development, it is increasingly important that COPs’ data are readily accessible, categorizable and interpretable for civil society organizations (CSOs).

**Methods:**

Utilizing standard open source tools, amfAR – funded by MAC AIDS – created a navigable database and website of all allocation data contained in published COPs from 2007 through 2014. Data are categorized and can be graphically represented and disaggregated by year, primary partner, host country, strategic area, budget code and organizational type of recipient. Text narratives of individual budgetary mechanisms captured directly from the COPs are also included in the database and provide users with detailed information for specific allocations. In addition, epidemiological profiles and PEPFAR targets are available by country to provide context for the public health impact of investments.

**Results:**

From 2007 through 2014, $29.5 billion was allocated through the COPs process. By organizational type, the primary recipients of PEPFAR funds were NGOs ($8.3 billion), private contractors ($5.4 billion) and universities ($3.5 billion). Another $5.5 billion was not allocated to an identifiable partner or programme. Trends varied substantially by country. In Rwanda, resources shifted dramatically to Rwandan government agencies (from 7.6% of PEPFAR resources in 2011 to 34.2% of PEPFAR resources in 2013). Comparatively, PEPFAR 2013 host government funding was lower for Kenya (10.72%), Malawi (3.66%), Nigeria (2.64%) and South Africa (0.8%).

**Conclusions:**

amfAR's COPs database provides corresponding financial and graphical information about progress towards country ownership and gives the most granular view to date of PEPFAR budgets. The database will be an invaluable tool to help CSOs and others digest and utilize PEPFAR budgetary information.

The database is available at http://copsdata.amfar.org

**Abstract WEAD0306LB–Figure 1:** Host Country Government Agency Allocations.

# Sub article: Within-host evolution of X4 HIV-1 in a rare transmission pair revealed by phylogenetic reconstruction of deep-sequence data

**DOI:** 10.7448/IAS.18.5.20420

**Published:** 2015-07-22

**Authors:** Anh Le, Jeremy Taylor, Winnie Dong, Rosemary McCloskey, Conan Woods, Kanna Hayashi, M-J Milloy, P Richard Harrigan, Art FY Poon, Zabrina L Brumme

**Affiliations:** 1 Health Sciences, Simon Fraser University, Burnaby, Canada; 2 BC Centre for Excellence in HIV/AIDS, Vancouver, Canada

## Abstract

**Introduction:**

A putative case of transmission of an X4 HIV strain from a CCR5wt/wt donor to a homozygous CCR5Δ32/Δ32 recipient was retrospectively identified in the Vancouver Injection Drug Users Study. We collected longitudinal intrahost deep-sequence data and applied ancestral phylogenetic reconstruction methods to characterize HIV transmission and evolution in this rare event.

**Methods:**

Pairwise genetic distances separating donor and recipient bulk plasma HIV *gag, pol, nef* and *env-V3* sequences were the lowest in the cohort (e.g. 0.0027 substitutions/nuc site in Gag vs. cohort median 0.06), identifying them as a putative transmission pair. The estimated transmission date (ETD), calculated as the midpoint of the recipient's last HIV-negative and first positive dates, was Aug/01. Donor plasma/Peripheral blood mononuclear cells (PBMCs) were available at −13, −7, −1 and +35 months from ETD; recipient plasma/PBMCs were available +5, +6 and +12 months from ETD. *Env-V3* from plasma-RNA and PBMC-DNA were triplicate amplified, pooled equally and deep-sequenced (Roche 454). BEAST and HyPhy were used to reconstruct phylogenies, estimate multiplicity of infection and reconstruct transmitted/founder (T/F) viruses from plasma-derived deep sequences from donor and recipient.

**Results:**

Despite infection with the same X4 HIV strain, donor CD4 count was 20 cells/mm^3^ within 1.5 years of infection whereas the recipient's remained >270 cells/mm^3^. Donor/recipient plasma viral loads were comparable (~4.5 Log). All 10 ancestral reconstructions were consistent with transmission of a single X4 T/F virus between May and August 2001. The estimated T/F virus sequence was identical to the co-dominant variant (36%) observed in the recipient's first (+5 month) timepoint. This sequence was also observed in 0.09% of donor plasma and 33.5% of PBMC at month −1, suggesting minority variant transmission. In the donor, reversion of ~60% of the total plasma virus population to an R5 phenotype occurred by 50 months post-infection; in contrast, the recipient's dominant V3 sequence steadily diversified over time but remained consistently X4.

**Conclusions:**

Results highlight the utility of phylogenetic reconstruction applied to deep-sequence data to characterize T/F viruses and intra-host evolution in transmission pairs. Differential CD4 depletion and V3 evolution in these individuals, despite acquisition of a near-identical X4 strain, underscores the critical role of host genetics on HIV evolution/pathogenesis.

# Sub article: Genetic ancestry component proportions are correlated with HIV disease progression

**DOI:** 10.7448/IAS.18.5.20421

**Published:** 2015-07-22

**Authors:** Daniela Garrido-Rodriguez, Santiago Avila-Rios, Humberto Valenzuela-Ponce, Thalia García-Tellez, Veronica Quiroz-Morales, Claudia García-Morales, Maribel Soto-Nava, Akio Murakami-Ogasawara, Juan Carlos Fernandez-Lopez, Gustavo Reyes Terán

**Affiliations:** 1 Centre for Research in Infectious Diseases, National Institute of Respiratory Diseases, Mexico City, Mexico; 2 National Institute of Genomic Medicine, Mexico City, Mexico

## Abstract

**Introduction:**

Genetic stratification within specific populations may explain differences in HIV control. We explore the influence of genetic diversity on HIV disease progression in a cohort of mestizo individuals with different proportions of European (EUR), Amerindian (AMI) and African (AFR) genetic ancestry components.

**Methods:**

We estimated individual ancestry proportions in a cohort of 565 HIV clade-B infected, antiretroviral treatment-naïve Mexican individuals using a panel of 128 ancestry informative markers. HLA alleles and KIR genes were genotyped for each participant in order to control for already known associations with HIV control and to describe putative novel associations in different genetic context.

**Results:**

The mean ancestral component proportions in the study cohort were 0.594 AMI, 0.38 EUR and 0.026 AFR, as previously observed in Mexican mestizo populations. We observed a negative correlation between the proportion of AMI ancestry component (p=0.0014) and a positive correlation between the proportion of EUR ancestry component (p=0.0004) and CD4 T cell counts. To try to explain these observations, we evaluated differences in frequency and effects on CD4 T cell counts of specific HLA alleles, KIR genes or HLA-KIR combinations in EUR 60% vs. AMI 60% individuals. A*31:01, B*39:05, B*44:03 and C*07:02 showed protective effects in individuals with high EUR component, but risk effects in individuals with high AMI component (p<0.05). Most KIR genes were more protective for EUR individuals than for AMI individuals. KIR+HLA-Bw4 combinations were more frequent in individuals with EUR component (p<0.05) while KIR+HLA-C1 combinations were more frequent in AMI individuals (p<0.05). Interestingly, the previously observed protective associations KIR3DS1/3DL1+HLA-Bw4^80Ille^ were not evident, neither in the entire cohort, nor in EUR individuals. KIR2DS4 in combination with HLA-C1 seemed to be protective for individuals with higher EUR component.

**Conclusions:**

This is the first time that differences in HIV disease progression associated with genetic stratification are shown in a single population. Further studies involving fine stratification of genetically diverse populations, exploring expression of other genes involved in HIV control are warranted to understand differences observed in this study.

# Sub article: Dasatinib preserves SAMHD1 antiviral activity in CD4+ T cells treated with IL-7

**DOI:** 10.7448/IAS.18.5.20422

**Published:** 2015-07-22

**Authors:** Jose Alcami, Mercedes Bermejo, Benjamin Descours, Elena Mateos, Michael M Lederman, Monsef Benkirane, Mayte Coiras

**Affiliations:** 1 Microbiology, Instituto de Salud Carlos III, Majadahonda, Spain; 2 Institute of Human Genetics, Montpellier, France; 3 Molecular Biology and Microbiology, Case Western Reserve University School of Medicine, Cleveland, United States

## Abstract

**Introduction:**

HIV-1 post-integration latency in quiescent CD4+T cells is responsible for viral persistence despite antiretroviral treatment. It was proposed that the increase in proviral load in HIV-infected patients after IL-7 treatment was due to homeostatic proliferation of memory CD4+T cells. We determined previously that IL-7 increased HIV-1 infection through phosphorylation and subsequent inactivation of the restriction factor SAMHD1. Now we analyzed SAMHD1 phosphorylation in PBMC from patients enrolled in ACTG 5214 study (NTC00099671), in order to elucidate the role of IL-7 in HIV-1 proviral integration and persistence and whether this could be related to SAMHD1 inactivation. In addition, we determined that the tyrosine-kinase inhibitor Dasatinib preserved SAMHD1 antiviral activity, avoiding IL-7-mediated HIV-1 infection.

**Methods:**

PBMC samples obtained from 10 patients enrolled in ACTG 5214 study (NTC00099671), collected before (day 0) and 4 after administration of IL-7. PBMCs obtained from two patients diagnosed with chronic myeloid leukaemia (CML), on chronic treatment with Dasatinib. Resting CD4+T cells from healthy donors obtained by negative selection from PBMCs. Phosphorylation of SAMHD1 at T592 was determined by immunoblotting and flow cytometry. Proviral integration was analyzed by TaqMan qPCR. Dasatinib (BMS-354825, Sprycel) was provided by Bristol-Meyers Squibb.

**Results:**

1) IL-7 (1 nM) induced SAMHD1 phosphorylation, interfering with its antiviral activity. 2) IL-7-mediated SAMHD1 phosphorylation greatly increased HIV-1 infection in purified CD4+T cells, increasing early and late retrotranscription, as well as proviral integration. 3) A significant increase in pSAMHD1 was observed in central memory CD4+T cells from HIV-infected patients treated with IL-7 (ACTG 5214). 4) Dasatinib completely inhibited SAMHD1 phosphorylation at 75 nM, interfering with HIV-1 retrotranscription and consequently, with proviral integration. 5) CD4+T cells from patients with CML treated with Dasatinib showed lower expression of SAMHD1 phosphorylated.

**Conclusions:**

By inducing SAMHD1 phosphorylation, IL-7 increases susceptibility of resting CD4+T lymphocytes to infection, leading to HIV persistence. SAMHD1 regulation plays a central role in the establishment of HIV-1 reservoirs and represents a major target for therapeutic intervention. Dasatinib is the first compound currently used in clinic that has been described to preserve the antiviral function of an innate factor such as SAMHD1.

# Sub article: HIV-specific latency reversing therapies that exploit novel pathways for suboptimal Tat protein expression

**DOI:** 10.7448/IAS.18.5.20423

**Published:** 2015-07-22

**Authors:** Damian Purcell, Jonathan Jacobson, Leigh Harty, Kate Jarman, Kurt Lackovic, Georges Khoury, Talia Mota, Michelle Lee, Giovana Bernardi, Suha Saleh, Secondo Sonza, Sharon Lewin

**Affiliations:** 1 Microbiology and Immunology at the Peter Doherty Institute, University of Melbourne, Melbourne, Australia; 2 The Walter and Eliza Hall Institute of Medical Research, Parkville, Australia; 3 Peter Doherty Institute, University of Melbourne, Melbourne, Australia

## Abstract

**Introduction:**

We have identified a footprint of viral Tat expression in latent HIV infected cells. Suboptimal levels of Tat arise from an IRES-mediated translation of chimeric cell-HIV mRNAs that arise from alternative splicing of read-through mRNA transcripts from cellular promoters adjacent to latent integrated provirus. To simulate the role of RNA-processing pathways in HIV latency, we recapitulated the low level Tat-expression from cellular-provirus read-through transcripts present in HIV latency reporter cells that express low-level Tat using the native IRES that underlies the first coding tat exon and a second, different Click-Beetle-Luciferase, expressed from a CMV-IE promoter to test specificity. Novel compounds and drug combinations were screened to identify HIV-specific drugs that synergize with this latent-viral signature. HIV-specific activation was further examined in T-cell models.

**Methods:**

We screened 5600 compounds in a known drug library and a library comprising of 114,000 drug-like compounds using a 293. IRES HIV-specific reporter cell line that contained CMV-CBG/LTR-CBR luciferase reporter system. Hits were identified that activated the LTR-CBR while having a minimal effect on the CMV-CBG reporter. A rigorous selection verification included 11-point titration in the normal and counter-screen assay cell lines, in dsRED-expressing J-Lat cells, and activity in primary cell models of latent HIV.

**Results:**

From this screening cascade, two known BET bromodomain and four HDAC inhibitors were found to significantly and specifically activate LTR promoter whereas compounds such as Vorinostat exhibited non-specific activity and increased global transcription. Several drug combinations that target different mechanisms implicated in HIV-1 latency were found to synergistically reactivate the virus with high potency. Importantly, seven novel compound classes were identified in the 114,000 compound library screens. Analogues of these seven classes were obtained and examined in 11-point assay with CMV-CBG/LTR-CBR reporter cell lines and 106 compounds gave a clear indication of early structure-activity relationships.

**Conclusions:**

Seven novel classes of HIV-specific latency purging drugs were found that activate HIV provirus in synergy with a low intrinsic expression of HIV RNA and Tat. These novel small molecule leads warrant further development to iteratively enhance their HIV-1 specificity and potency. We also identified new drug combinations that synergistically activate expression from the latent HIV-1 LTR.

# Sub article: HIV rebound and meningoencephalitis following ART interruption after allogeneic hematopoietic stem cell transplant: an investigation of the source of HIV rebound

**DOI:** 10.7448/IAS.18.5.20424

**Published:** 2015-07-22

**Authors:** Adam Capoferri, Matthew Sievers, Andrew Redd, Ayla Cash, Daniel Xu, Steven F Porcella, Thomas Quinn, Robert F Siliciano, Mark Levis, Richard F Ambinder, Christine M Durand

**Affiliations:** 1 Howard Hughes Medical Institute, Baltimore, United States; 2 Department of Medicine, Johns Hopkins University, Baltimore, United States; 3 Division of Intramural Research, National Institutes of Health, National Institute of Allergy and Infectious Disease, Bethesda, United States; 4 Research Technologies Branch, Genomics Unit, Rocky Mountain Laboratories, Hamilton, United States; 5 Sidney Kimmel Cancer Center, Baltimore, United States

## Abstract

**Introduction:**

Allogeneic hematopoietic stem cell transplant (alloHSCT) with uninterrupted antiretroviral therapy (ART) is being investigated as a component of HIV eradication strategies. In the two “Boston patients,” alloHSCT resulted in the disappearance of HIV in peripheral blood. However, after analytical ART interruption, viral rebound occurred. Proposed sources of HIV rebound include the latent reservoir in resting CD4+T cells and tissue macrophages. We present the case of an HIV-infected patient, who received alloHSCT for leukaemia and experienced acute retroviral syndrome after self-discontinuing ART post-alloHSCT.

**Methods:**

Resting memory CD4+T-cells obtained 16 and 1 week prior to alloHSCT were used in a limiting-dilution viral outgrowth assay (VOA) in which each well that demonstrates viral growth contains a single replication-competent viral clone. The *pol* region of virus from positive VOA supernatants was sequenced. Rebound virus from blood and cerebrospinal fluid (CSF) was also analyzed using deep-sequencing (Roche 454) of *pol*. Sequences were aligned and maximum likelihood analysis was performed using the GTR+G model of evolution with 100 bootstrapping pseudoreplicates.

**Results:**

The patient had undetectable plasma HIV and achieved 100% donor chimerism at week 12 post-alloHSCT, but then became non-adherent with ART. At five months, the patient presented with fever and meningoencephalitis. Plasma and CSF HIV levels were 25,500 and 17,000 copies/mL, respectively. Before alloHSCT, 31 sequences were isolated from the VOA. At rebound, 14,645 and 5003 sequence reads were obtained from CSF and blood respectively and were combined into consensus sequences using a cut-off of>0.2% of total sequence reads. An identical sequence found at both pre-alloHSCT timepoints accounted for 9/31 (29%) of independent VOA sequences. This sequence grouped with the plasma and CSF viral rebound sequences in a monophyletic clade with high sequence homology.

**Conclusions:**

Despite 100% donor chimerism in peripheral blood, ART interruption led to HIV rebound in plasma and CSF. Rebound virus was identical to a pre-alloHSCT isolate which compromised nearly 1/3 of the latent CD4+T-cell reservoir sampled. This unique case suggests that recipient cells persist at early time-points after alloHSCT and that a single viral population latent in resting memory CD4+T cells can re-establish infection.

**Abstract MOPDA0105–Figure 1:** ML Tree.

# Sub article: Assay to measure the latent reservoir of replication-competent HIV-1 in suppressed patients based on ultra deep sequencing

**DOI:** 10.7448/IAS.18.5.20560

**Published:** 2015-07-22

**Authors:** Sook-Kyung Lee, Shuntai Zhou, Nancie Archin, David Margolis, Ronald Swanstrom

**Affiliations:** 1 Department of Biochemistry and Biophysics, UNC Center for AIDS Research, University of North Carolina, Chapel Hill, United States; 2 Department of Medicine, University of North Carolina, Chapel Hill, United States

## Abstract

**Introduction:**

Viral outgrowth assay (VOA) is a widely used culture assay to measure the latent HIV-1 reservoir harbouring replication-competent HIV-1 in resting CD4+ T cells in patients on HAART. However, the assay is costly, and both labour and resource intensive. To overcome some of these issues with the VOA, we designed an assay using ultra deep sequencing (UDS), which directly analyzes the number of different sequences of the induced viruses to score the number of latently HIV-infected resting CD4+ T cells. In this study, we tested the premise whether the viral sequences derived from two different proviruses are genetically distinct, since the assay involves a bulk culture.

**Methods:**

To analyze viruses derived from different VOA culture wells scored as p24 positive, the viral samples derived from different culture wells were assigned with a specific Barcode and subjected to sequence analysis of the V1–V3 region of *env* sequences using the Primer ID-based paired-end MiSeq platform. A total of nine patient samples, two acute and seven chronic, were analyzed by UDS. Phylogenetic trees were generated by using consensus sequences created from sequences with the identical Primer ID and were used to detect distinct viral lineages present in the individual culture supernatant. For chronic patient samples, IUPM values were determined by using distinct viral lineages detected and the adjusted number of patient-derived resting CD4+ T cells used for VOA.

**Results:**

Approximately 50% of the viral lineages derived from each chronic patient were distinct. In contrast, all viral lineages derived from each acute patient were homogeneous. When IUPM values determined by UDS analysis were compared to the IUPM values obtained from VOA, we observed approximately twofold higher IUPM values than the IUPM values determined by VOA. We also observed a significant positive correlation between the number of viral lineages observed per well and the number of resting T cells present per well.

**Conclusions:**

The results suggest that approximately 50% of the viral lineages induced from different cells derived from chronic patients were distinct. Thus, the UDS assay is applicable for samples derived from chronic patients. The multiplexing ability of the assay improves the efficiency for the throughput capacity.

# Sub article: Bacterial vaginosis, intravaginal practices and HIV genital shedding: implications for HIV transmission

**DOI:** 10.7448/IAS.18.5.20425

**Published:** 2015-07-22

**Authors:** Maria Alcaide, Maureen Chisembele, Emeria Malupande, Kristopher Arheart, Deborah Jones, Margaret Fischl

**Affiliations:** 1 Division of Infectious Diseases, Miller School of Medicine, University of Miami, Miami, United States; 2 Obstetrics and Gynecology, University of Zambia, Lusaka, Zambia

## Abstract

**Introduction:**

Bacterial vaginosis (BV) is associated with an increased risk of HIV transmission, and intravaginal practices (IVP), the practice of cleansing the vagina for hygienic, health or sexuality reasons, is the primary risk factor for developing BV. This study examines the relationship between BV, IVP and lower genital HIV shedding in HIV infected women in Zambia.

**Methods:**

Participants were HIV-1 infected women, older than 18 years and living in Lusaka, Zambia. Participants completed audio computer administered self-interviews questionnaires assessing demographic, sexual risk factors and IVP. BV was diagnosed by gram stain of vaginal secretions using Nugent criteria. HIV-1 plasma viremia and genital shedding was assessed by measuring HIV-1 RNA in plasma and cervico vaginal lavages using real time PCR.

**Results:**

One hundred and twenty-eight HIV-1 infected women were enrolled. Mean age was 37 years (range 24–60). Most had a stable male sex partner (126; 98%), and the majority of male partners had HIV infection (86; 67%). About one third (44; 34%) reported more than one partner in the prior year. All participants had engaged in IVP in the prior month, and over 90% used IVP daily. Ninety-eight participants (76%) had abnormal vaginal flora (Nugent score of 4–10); and 80 (62%) had BV (Nugent score 7–10). HIV-1 plasma viremia was detected in 26 participants (20%) (median=8.4 log copies/mL, range=3.9–14.5). HIV-1 genital shedding was detected in 18 participants (14%) (median=6.7 log copies/mL, range=3.6–12.7). In multivariate analysis, daily IVP were associated with BV (OR=7.9, CI=1.54–40.8, p<0.01) and plasma viremia was associated with HIV-1 genital shedding (OR=7.23, CI=2.43–21.37, p<0.01). Demographic, sexual risk factors, IVP or BV were not associated with HIV genital shedding.

**Conclusions:**

BV was common in this sample of women with HIV infection and occurred in women engaging in frequent IVP. Neither BV nor IVP increased HIV genital shedding in women on suppressive antiretrovirals. Effective antiretroviral therapy remains the main strategy to prevent HIV female genital shedding and risk of subsequent HIV transmission. Further research in women with detectable plasma viremia is needed to examine how IVP and BV affect the vaginal mucosa and increase HIV transmission.

Study was funded by NIH, K23HD074489.

# Sub article: IUD use in HIV-positive women

**DOI:** 10.7448/IAS.18.5.20426

**Published:** 2015-07-22

**Authors:** Laura Anca Vicol, Alicia Hornsberger, Neora Pick, Julie Van Schalkwyk, Dena Bloomenthal, Jan Christilaw, Deborah Money

**Affiliations:** 1 Oak Tree Clinic, BCWH, PHSA, Vancouver, Canada; 2 WHRI, BCWH, PHSA, Oak Tree Clinic, Vancouver, Canada

## Abstract

**Introduction:**

Eighty percent of HIV-positive (HIV+) women are of childbearing age, therefore access to effective, safe contraception is essential. Generally, intrauterine device (IUD) provide safe, effective contraception but historically, IUDs were contraindicated in HIV+ women due to concerns regarding infection. Data in HIV+ women are scarce. The goal was to assess rate of complications for IUS insertion in HIV+ women.

**Methods:**

IUDs insertions in HIV+ women were offered at Oak Tree Clinic (the provincial referral centre for HIV+ women and children) since 2009, following strict clinical evaluations for eligibility. Criteria used for insertion were: not planning a pregnancy for at least one year; requesting a reversible contraceptive, wanted/needed to avoid oestrogen-based methods and CD4>150. STD screening was done in all cases. Demographic information collected included: age, CD4, ARV at insertion and purpose of IUD (contraception vs. cycle control).

**Results:**

Data was reviewed from 44 sequential women given IUDs from 2009 to 2014 with ages 17– 48. CD4 count 160–1230 (median 590); 32/44 (73%) had viral loads <40 c/ml, 9 women had detectable VL between 89–126,908 c/ml. 7 were not on ARV therapy. 2 were on ARV but struggled with adherence and were detectable. 3/44 had a copper IUD. 40/44 had a hormonal IUD. 1 had a hormonal followed by a copper IUD. 3 IUDs were inserted for menorrhagia. 1 IUD was for combined therapeutic and contraceptive purposes. 5 requested the hormonal IUD removed not related to reproductive plans. 1 refused reinsertion when the expired hormonal IUD was removed. Complications included four IUD expulsions (9%) (three spontaneous; one partial within the cervix). The rate of expulsion in general population is 6%. One IUD was removed by hysteroscopy due to upward migration of strings and myometrial embedment. One IUD accidentally pulled out during intercourse and required emergent reinsertion of a new device. No IUD related infections or other serious complications occurred, regardless of CD4 count.

**Conclusions:**

In this small series of HIV+ women, IUDs were safe and well tolerated. This method of contraception should remain an option for HIV+ women if close follow up of short and long term complications can be followed.

# Sub article: Effectiveness of contraception for HIV-infected women using antiretroviral therapy: combined data from three longitudinal studies

**DOI:** 10.7448/IAS.18.5.20427

**Published:** 2015-07-22

**Authors:** Maria Pyra, Renee Heffron, Nelly R Mugo, Kavita Nanda, Katherine K Thomas, Connie Celum, Athena P Kourtis, Jared M Baeten, Partners in Prevention HSV/HIV Transmission Study and Partners PrEP Study Teams

**Affiliations:** 1 Department of Global Health, University of Washington, Seattle, United States; 2 Department of Epidemiology, University of Washington, Seattle, United States; 3 Department of Obstetrics and Gynaecology, University of Nairobi, Nairobi, Kenya; 4 Sexual, Reproductive, Adolescent and Child Health Research Program, Kenya Medical Research Institute, Nairobi, Kenya; 5 Integrated Health Sciences, FHI 360, Research Triangle Park, United States; 6 Department of Medicine, University of Washington, Seattle, United States; 7 Division of Reproductive Health, National Center for Chronic Disease Prevention and Health Promotion, Centers for Disease Control and Prevention, Atlanta, United States

## Abstract

**Introduction:**

Ensuring safe, effective contraception for women with HIV-1 is a public health imperative. Some data suggest that antiretroviral therapy (ART) may diminish the effectiveness of certain contraceptive methods, particularly implants.

**Methods:**

Combining data from 5282 HIV-infected women participating in three longitudinal studies (Partners in Prevention HSV/HIV Transmission Study, Couples Observation Study and Partners PrEP Study) from seven countries in Africa between 2004 and 2012, we calculated incident pregnancy rates among women using different contraceptive methods (implant, injectable and oral) and compared those to rates among women using no contraception. Multivariable Cox regression models controlled for confounding factors, and the interaction between each contraceptive method and ART use was tested to assess if ART diminished contraceptive effectiveness.

**Results:**

During follow-up (median 1.8 years, IQR 1.2–2.3), 9% of women ever used implant, 41% used injectables (primarily depot medroxyprogesterone acetate (DMPA)), 15% used oral pills and 47% never used hormonal contraception. Additionally, 31% of women ever used ART during follow-up, including 23% using nevirapine and 5% using efavirenz. Among women not using contraception, pregnancy rates were 13.2 and 22.5 per 100 women-years for those on and not on ART, respectively. Use of implants reduced the risk of pregnancy by more than 90%, both among women on ART (aHR 0.06, 95% CI 0.01–0.45) and not on ART (aHR 0.05, 95% CI 0.02–0.11). Likewise, injectables reduced pregnancy risk (aHR 0.18, 95% CI 0.09–0.35 on ART and aHR 0.20, 95% CI 0.16–0.24 not on ART), as did oral contraceptives by a lesser degree (aHR 0.37, 95% CI 0.15–0.91 on ART and aHR 0.36, 95% CI 0.28–0.47 not on ART). We found no statistical evidence that ART use diminished contraceptive effectiveness, including for nevirapine and efavirenz, although sample size was limited for assessing specific ART agents.

**Conclusions:**

In this large prospective evaluation of three studies, modern contraceptive methods were highly effective in reducing pregnancy risk in HIV-infected women, including those concurrently using ART. While limited evidence from other studies suggests that some ART agents could diminish the effectiveness of contraceptive implants, these data emphasize that implantable contraception is highly effective compared to no contraception and more so than shorter-acting methods such as injectables and oral pills.

**Abstract MOPDB0103–Table 1:** Contraceptive effectiveness, by ART status and type

Progestin use	ART Use	# Pregnancies	Person-years	Incidence rate (per 100 person-years)	aHR* (95% CI), reference no contraception	p-value for interaction term
No contraception	On ART	111	843.5	13.2	–	–
No contraception	No ART	1067	4733.6	22.5	–	–
Implant	On ART	1	94.1	1.1	0.06 (0.01, 0.45)	0.73
Implant	No ART	7	507.8	1.4	0.05 (0.02, 0.11)	
Injectable	On ART	11	332.8	3.3	0.18 (0.094, 0.35)	0.79
Injectable	No ART	111	2100.2	5.3	0.20 (0.16, 0.24)	
Oral pills	On ART	5	81.2	6.2	0.37 (0.15, 0.91)	0.97
Oral pills	No ART	63	573.1	11.0	0.36 (0.28, 0.47)	
Total		1376	9266.3	14.8	–	–

# Sub article: Importance of programmatic longitudinal surveillance for identification of congenital anomalies among infants exposed to HIV-1 and antiretrovirals: findings from the Mpepu Study, Botswana

**DOI:** 10.7448/IAS.18.5.20428

**Published:** 2015-07-22

**Authors:** Gbolahan Ajibola, Roger Shapiro, Rebecca Zash, Lewis Holmes, Oganne Batlang, Kerapetse Ramogothobeng, Florence Chilisa, Kara Bennett, Joseph Makhema, Shahin Lockman, Kathleen Powis

**Affiliations:** 1 Botswana Harvard AIDS Institute Partnership, Gaborone, Botswana; 2 Department of Immunology and Infectious Diseases, Harvard School of Public Health, Boston, United States; 3 Division of Infectious Diseases, Beth Israel Deaconess Medical Center, Boston, United States; 4 Department of Pediatrics, Massachusetts General Hospital, Boston, United States; 5 Bennett Statistical Consulting, Ballston Lake, United States; 6 Infectious Disease Unit, Brigham and Women's Hospital, Boston, United States; 7 Department of Internal Medicine, Massachusetts General Hospital, Boston, United States

## Abstract

**Introduction:**

A large and increasing number of HIV-infected women are conceiving while taking antiretrovirals (ARVs) globally. In resource-limited settings, surveillance systems, if present, often are limited to the initial birth exam.

**Methods:**

We used pre-randomization data from May 2011 to December 2014 from an ongoing clinical trial of infant cotrimoxazole prophylaxis in Botswana. Enrolments of live-born infants of HIV-infected women occurred after delivery, so long as the mother consented to infant participation and no infant life-threatening conditions were identified at birth. Infants were examined by study staff at delivery, and monthly in the first three months of life, and congenital anomalies were documented. We present a descriptive analysis of anomalies identified after the initial birth exam.

**Results:**

Of 2935 HIV-infected women enrolled in the Mpepu study who delivered live-born infants, newborn exams were documented on 2900 (99%) infants. ART from conception was documented for 1088 (38%) women; 1147 (40%) started ARVs during pregnancy; 442 (15%) women received AZT monotherapy; and 223 (7%) received no ARVs during pregnancy. A total of 28 congenital anomalies were identified, and 8 (29%) were first diagnosed at a visit after the initial birth exam (Table 1). No differences were identified in the number of infants with or without congenital abnormalities by ARV exposure group in pregnancy, but the study was underpowered to detect differences in rare outcomes. Identification of congenital anomalies after the birth exam occurred either because the anomaly was not readily apparent at birth (e.g. biliary atresia), or because an externally-identifiable anomaly was overlooked at birth but subsequent parental concern led to documentation and management of the anomaly.

**Conclusions:**

ARV use in pregnancy warrants ongoing surveillance monitoring for teratogenicity, particularly for regimens such as EFV/FTC/TDF with insufficient safety data in pregnancy. Nearly one third of birth anomalies detected in this cohort of well children were diagnosed after the initial birth exam. Our findings highlight the importance of incorporating, where possible, longitudinal assessment and reporting for detection of congenital anomalies that may not be identifiable at the birth exam.

**Abstract MOPDB0104–Table 1:** Congenital anomalies

Case #	Description of congenital anomaly	Presenting symptom	Timing of diagnosis from birth
1	Anovestibular fistula	Stool in urine	42 days
2	Biliary atresia	Jaundice at birth	48 days
3	Biliary atresia	Jaundice at birth	38 days
4	Congenital Lymphedema	Bilateral leg swelling	15 days
5	Jejunal atresia	Failure to pass stool with abdominal distension and vomiting	5 days
6	Macrocephaly	Widening of fontanelle and increasing head size	85 days
7	Pyloric stenosis	Projectile vomiting	25 days
8	Talipes equino valgus	Concern expressed by mother about position of foot	60 days

# Sub article: Communities can mobilize to test: findings from a community randomized trial of a theory-based community mobilization intervention in South Africa

**DOI:** 10.7448/IAS.18.5.20429

**Published:** 2015-07-22

**Authors:** Sheri A Lippman, Audrey Pettifor Pettifor, Torsten B Neilands, Catherine MacPhail, Dean Peacock, Suzanne Maman, Rhian Twine, Amanda Selin, Kathleen Kahn

**Affiliations:** 1 Department of Medicine, Center for AIDS Prevention Studies, University of California, San Francisco, United States; 2 Gillings School of Global Public Health, University of North Carolina at Chapel Hill, Chapel Hill, United States; 3 Wits Reproductive Health and HIV Institute, School of Clinical Medicine, Faculty of Health Sciences, University of the Witwatersrand, Johannesburg, South Africa; 4 School of Health, University of New England, Armidale, Australia; 5 Sonke Gender Justice, Cape Town, South Africa; 6 MRC/Wits Rural Public Health and Health Transitions Research Unit (Agincourt), School of Public Health, Faculty of Health Sciences, University of the Witwatersrand, Johannesburg, South Africa; 7 Centre for Global Health Research, Division of Epidemiology and Global Health, Umeå University, Umeå, Sweden

## Abstract

**Background:**

While community mobilization (CM) is a powerful tool to increase and sustain demand for HIV testing services, few rigorous trials of CM interventions have been conducted. We implemented a theory-driven CM intervention in order to improve HIV outcomes in 22 communities participating in a community randomized trial (CRT) in a rural area of Mpumalanga Province, South Africa. The mobilization activities were designed to improve community collaboration to address HIV and inequitable gender norms.

**Methods:**

Cross-sectional surveys were conducted with 50–55 residents ages 18–35 in each village prior to (n=1181; 2012) and following (n=1174; 2014) two years of intensive intervention activities in half of the villages. Intervention activities mapped onto six domains of CM: 1) shared concern around HIV, 2) community consciousness, 3) organizational structures, 4) leadership, 5) community cohesion and 6) collective action. Validated domain measures were included in the surveys and mean community CM scores were computed and used to predict HIV testing in the past year for each domain and for total CM scores. We used GEE logistic regression analysis to assess the effect of village level CM domain scores on individual-level testing outcomes and included interaction terms to assess intervention effects at follow-up.

**Results:**

The overall CM score as well as three of six CM domains, including consciousness, concerns, collective action, were significantly associated with HIV testing following the intervention and interacted with intervention assignment. For example, for every standard deviation increase in community consciousness, the odds of HIV testing increased for intervention village participants (OR: 1.36, p=<0.01) but not for control village participants. Similar findings for total CM score (OR: 1.51), shared concerns (OR: 1.62) and collective action (OR: 1.45) indicate that the intervention successfully improved HIV testing. Leadership, presence of organizations and community cohesion were not significantly associated with HIV testing at end line.

**Conclusions:**

To our knowledge this is the first CRT assessing a theory-based CM intervention including quantitative measures of CM domains over time. While not all of the six domains were associated with HIV testing uptake, we found clear evidence that communities can be mobilized and that CM measures are associated with improved engagement in HIV testing.

# Sub article: Reducing stigma and increasing HIV testing with a health information intervention, a cluster-randomized trial from Malawi

**DOI:** 10.7448/IAS.18.5.20430

**Published:** 2015-07-22

**Authors:** Laura Derksen, Adamson Muula, Joep van Oosterhout, Monique van Lettow, Alfred Matengeni, Sumeet Sodhi

**Affiliations:** 1 Department of Economics, London School of Economics, London, United Kingdom; 2 College of Medicine, University of Malawi, Blantyre, Malawi; 3 Dignitas International, Zomba, Malawi; 4 Toronto Western Hospital, University Health Network, Toronto, Canada

## Abstract

**Background:**

Despite widespread availability of antiretroviral therapy (ART), demand for HIV testing remains low across southern Africa. HIV testing may be viewed as a signal of HIV status. Those who seek an HIV test may be rejected by potential sexual partners who fear contracting HIV. This could discourage HIV testing and encourage travel far from home for HIV testing to avoid being seen. Such stigma may be exacerbated by unawareness of the public benefit of ART, that is, its capacity to reduce HIV transmission by 96%. We evaluated an information experiment designed to increase HIV testing rates by reducing stigma.

**Methods:**

We conducted a cluster-randomized controlled trial in Malawi. We held community health information meetings in all villages. In control villages (n=62), we provided information on the private benefits of ART, including its potential to prolong life and reverse AIDS symptoms. In intervention villages (n=60), the public benefit of ART was discussed in addition to the control message.

**Results:**

Among those aged 15–49, there was a significantly larger uptake of HIV testing in the intervention villages (intervention 2.6% vs. control 1.6%; p=0.0035), according to routinely collected data from 18 health facilities over a period of three months after the intervention. This effect was significant for men and women, and larger when corrected for spill-overs. The intervention led to a large shift in beliefs about ART, as measured by a survey five months after the intervention. Respondents in intervention villages were more likely to report accepting attitudes towards sexual partners on ART. High beliefs about the public benefit of ART were associated with significantly more tests at nearby clinics. HIV testing decisions were predicted by a respondent′s perception of his/her community's beliefs about ART. These observations strongly suggest that the effect of the intervention on HIV testing uptake is mediated by a reduction in stigma.

**Conclusions:**

The results demonstrate that stigma between sexual partners is a significant barrier to HIV testing, and that providing new information on the effect of ART on HIV transmission can increase testing uptake.

# Sub article: HIV self-testing increases HIV testing frequency among high-risk men who have sex with men: a randomized controlled trial

**DOI:** 10.7448/IAS.18.5.20431

**Published:** 2015-07-22

**Authors:** David Katz, Matthew Golden, James Hughes, Carey Farquhar, Joanne Stekler

**Affiliations:** 1 Department of Epidemiology, University of Washington, Seattle, United States; 2 Public Health – Seattle & King County, United States, Seattle

## Abstract

**Background:**

HIV self-testing has the potential to increase HIV testing and thereby decrease the time persons living with HIV are unaware of their status, but the absence of counselling may result in increased risk of HIV acquisition.

**Methods:**

In Seattle, Washington, we randomly assigned 230 HIV-negative men who have sex with men (MSM) at high risk for HIV acquisition in a 1:1 ratio to have access to HIV self-testing using the OraQuick ADVANCE Rapid HIV-1/2 Antibody Test on oral fluids or to testing as usual for 15 months. Men randomized to self-testing were trained to use the test and provided a self-test at baseline; they could contact the study for additional tests as needed up to once a month. All participants were advised to test quarterly, offered testing reminders and could test through any existing HIV testing source. The primary outcome was self-reported number of HIV tests during follow-up. To evaluate potential adverse effects of self-testing, we compared the following between the two arms: non-concordant condomless anal intercourse (CAI) and number of male CAI partners in the last three months (measured at 9 and 15 months) and diagnosis with a bacterial sexually transmitted infection (STI) at the final study visit (15 months).

**Results:**

Men randomized to self-testing reported significantly more HIV tests during follow-up (mean=5.3, 95% CI=4.7–6.0) than those in the control arm (3.6, 3.2–4.0; p<0.0001), representing an average increase of 1.7 tests per participant over 15 months. Men randomized to self-testing reported using an average of 3.9 self-tests during follow-up. Self-testing was non-inferior to clinic-based testing with respect to markers of HIV acquisition risk. At the final study visit, 5.4% of MSM randomized to self-testing were diagnosed with a bacterial STI compared with 12.2% of control participants (risk difference=−6.8%; 95% CI=−16 to +1.6%). There were no significant differences between the two arms in the proportion of men reporting non-concordant CAI or the reported number of male CAI partners in the last three months at 9 and 15 months.

**Conclusions:**

Access to free HIV self-testing increased testing frequency among high risk MSM and did not impact sexual risk behaviour or STI acquisition.

**Abstract MOPDC0103–Figure 1:** Distribution of self-reported HIV tests by arm.

# Sub article: Home HIV testing among transgender women in San Francisco: a pilot feasibility and acceptability study

**DOI:** 10.7448/IAS.18.5.20432

**Published:** 2015-07-22

**Authors:** Sheri A Lippman, Mary E Moran, Angel Ventura, Lesley S Castillo, Susan Buchbinder, Sarah Treves-Kagan, Jae M Sevelius

**Affiliations:** 1 Center for AIDS Prevention Studies, University of California, San Francisco, United States; 2 School of Public Health, University of California, Berkeley, United States; 3 San Francisco Department of Public Health, Bridge HIV, San Francisco, United States

## Abstract

**Background:**

Transgender women are the population most impacted by HIV in the United States, with prevalence approximately 40 times higher than the general population. The rates of HIV antibody testing in the transgender community are not commensurate with risk. Development of alternative testing strategies to ensure early detection, care and prevention of infection is critical.

**Methods:**

We conducted a pilot study to explore feasibility and acceptability of offering home-based, self-conducted HIV testing for transwomen. Fifty HIV-negative transwomen in San Francisco were provided with *OraQuick* oral HIV self-test kits and asked to utilize the tests once a month for three months. Survey data were collected at baseline, one month and three months. In-depth-interviews (IDIs) were conducted with 11 participants at their final visit to learn more about self-testing experiences, barriers to self-testing and how the self-test might fit into an expanded pool of testing options.

**Results:**

Self-testing was both feasible and acceptable: following the first test 94% reported the test easy to use; 93% said the results were easy to read; and 91% said they would recommend the self-test to others. Acceptability remained high at three months. Approximately 25% used the test kit with others present and 68% reported preference for self-tests versus clinic-based testing. IDIs revealed tension between a desire for the privacy afforded by self-testing and a desire for the social and resource support offered at health facilities. While most participants were comfortable accessing services and had been tested recently (88% in the past year), IDIs revealed apprehension about being seen at HIV-testing clinics. Qualitative data also indicated that partner testing was of interest and that the cost of the kits could discourage future utilization.

**Conclusions:**

The home-based, self-conducted HIV test provides a viable option for populations who prefer to avoid the clinic environment. To increase acceptability, enhanced linkage strategies to social and resource support should be considered. The current price point is inaccessible for populations that experience disproportionate economic marginalization. Interest in partner testing could represent an opportunity to package tests in pairs and an expanded opportunity for testing uptake. Additional research should focus on expanding delivery options and implementation strategies.

# Sub article: Supervised HIV self-testing to inform implementation and scale up of self-testing in Zimbabwe

**DOI:** 10.7448/IAS.18.5.20433

**Published:** 2015-07-22

**Authors:** Sue Napierala Mavedzenge, Euphemia Sibanda, Yvonne Mavengere, Karin Hatzold, Owen Mugurungi, Getrude Ncube, Frances Cowan

**Affiliations:** 1 RTI International, Women's Global Health Imperative, San Francisco, United States; 2 Centre for Sexual Health and HIV/AIDS Research, Harare, Zimbabwe; 3 Population Services International, Harare, Zimbabwe; 4 Zimbabwe Ministry of Health and Child Care, Harare, Zimbabwe; 5 Centre for Sexual Health and HIV Research, University College London, London, United Kingdom

## Abstract

**Background:**

HIV self-testing (HIVST) can potentially increase uptake of testing in a low-cost, confidential and non-stigmatizing manner. Rigorous evaluation of instructional materials for accurate self-testing has rarely been conducted. In preparation for implementation and scale-up of HIVST in Zimbabwe, we have adapted and iteratively refined instructional materials to support self-testing. Here we present results from our evaluation of these materials through supervised self-testing.

**Methods:**

Participants were recruited at an HIV testing clinic using convenience sampling. They were given the instructional materials and left alone to complete their self-test and record the result. Confirmatory rapid testing after HIVST, and pre- and post-test questionnaires to evaluate their experience were conducted. The testing process was video recorded and videos analyzed using checklists. Data were evaluated weekly and IEC materials iteratively refined accordingly to optimize accuracy.

**Results:**

We conducted 172 supervised self-tests among participants in urban Harare, with mean age of 30 (range 18–70), 53% female and 20% first-time testers. Overall 93% read their result accurately, in some cases despite failing to follow instructions as determined by video. Six percent were unable to determine their result. One percent got inaccurate results, including one HIV+ individual on antiretroviral therapy (ART) who followed instructions correctly as determined by video. While most (88%) reported the test was not hard to use, 23% said some instructions were unclear, resulting in modifications to the materials. Common sources of confusion were in interpreting results, the purpose of the test kit desiccant and unclear images/language. Low literacy was associated with unsure/invalid results, prompting revision of the materials for a rural, less literate setting. There, among 29 participants, 3% were unable to determine their results and 31% got an inaccurate result. Materials have been further revised making them almost entirely pictorial, and supervised self-testing is ongoing.

**Conclusions:**

Though there is little published research on optimizing HIVST materials, we found that thorough evaluation of materials through supervised self-testing has been critical to optimizing accuracy. Numerous revisions were required, and evaluation in different settings yielded differing results. Rigorous development and testing of HIVST supportive materials appropriate to country and setting is recommended prior to implementation of HIVST programs.

**Abstract MOPDC0105–Table 1:** HIV results among 172 participants in Harare

	Participant-read HIVST	Staff-read HIVST	Confirmatory test	
HIV negative	146	150 (146+3 unsure+1 transcription error*)	156 (149+7 invalid HIVST)	• 160/172=93% got an accurate HIVST result • 2/172=1% got an inaccurate HIVST result* • 10/172=6% unable to decipher their HIVST result. 7 (4%) of these had performed the test incorrectly, 3 (2%) could not interpret their result
HIV positive	16*	15	16	
HIV unsure	5	0	0	
HIV invalid	5	7	0	

* One was a participant transcription error – she was clear in her post-HIVST interview that she thought she was HIV negative. The second was someone on ART who tested negative via self-test and positive in confirmatory testing.

# Sub article: Integrating partner notification services into PMTCT (Option B+) services in the northwest and southwest regions of Cameroon

**DOI:** 10.7448/IAS.18.5.20434

**Published:** 2015-07-22

**Authors:** Pius Tih Muffih, Eveline Mboh, Ebua Fang, Winifred Wainfen, Honere Fon, Thomas Welty, Raymond Shields, Mathew Golden

**Affiliations:** 1 Cameroon Baptist Convention Health Services, Bamenda, Cameroon; 2 Cameroon Baptist Convention Health Board, AIDS Care and Prevention Program, McCall, Idaho, United States; 3 Cameroon Baptist Convention Health Services, Bellingham, United States; 4 Department of Epidemiology, University of Washington, Seattle, United States

## Abstract

**Background:**

Partner notification (PN) for control of sexually transmitted infections (STIs) is a public health strategy which notifies the partners of infected individuals of their possible exposure to disease. PN has rarely been used in sub-Saharan Africa as an HIV prevention intervention. In Cameroon, patients newly diagnosed with HIV do not usually receive assistance in notifying their sex partners leading to low partner disclosure and poor partner involvement in prevention of mother-to-child transmission (PMTCT). In 2012, the World Health Organization issued new guidelines in PMTCT including Option B+which recommends that all HIV positive pregnant women (PW) be placed on antiretroviral treatment for life irrespective of CD4 count. PN was integrated into PMTCT at 22 pilot Option B+sites as a strategy to increase male partner disclosure, notification, testing and linkage to care.

**Methods:**

Beginning in March 2013, Trained Health Advisors (HA) at the 22 B+sites interviewed consenting HIV-positive PW about their sexual partners in the last two years and facilitated disclosure or confidentially informed their partners that they had been exposed to HIV. The HAs pre-test counselled the partners and offered HIV testing in the clinic, their home or other location. They then educated both index cases and their partners on HIV prevention and risk reduction and linked all HIV positive partners to care and treatment.

**Results:**

During the 18 months, uptake was monitored monthly and 823 PW tested HIV positive at the 22 option B+sites (Figure 1). Of the 840 partners they identified, 693 (82.5%) were traced and notified of their exposure to HIV. Of the 693 notified, 421 (60.8%) did their HIV test and received results. A total of 139 (33.0%) of those tested were HIV positive and 138 (99.3%) were linked to appropriate C&T services. HIV negative partners (67.0% of those tested) were counselled on risk reduction. Male partner involvement increased greatly at seven of ten sites monitored.

**Conclusions:**

PN is a feasible HIV prevention strategy in resource-limited settings which can identify and test many partners of HIV positive PW. PN can be integrated into Option B+PMTCT programs to identify HIV positive partners who are placed on treatment alongside the HIV positive PW.

**Abstract MOPDC0106–Figure 1:** Uptake of PN services at 22 B + sites.

# Sub article: The effect of opiate substitution therapy on healthcare utilization and engagement among HIV-infected people who inject drugs in Ukraine

**DOI:** 10.7448/IAS.18.5.20435

**Published:** 2015-07-22

**Authors:** Chethan Bachireddy, Jacob Izenberg, Michael Soule, Sergey Dvoryak, Frederick Altice

**Affiliations:** 1 Department of Internal Medicine, Brigham and Women's Hospital, Harvard Medical School, Boston, United States; 2 Department of Psychiatry, University of California, San Francisco, United States; 3 Department of Psychiatry, Massachusetts General Hospital McLean, Harvard Medical School, Boston, United States; 4 Ukrainian Institute on Public Health Policy, Kyiv, Ukraine; 5 Department of Internal Medicine, Yale University School of Medicine, New Haven, United States

## Abstract

**Background:**

Eastern Europe and Central Asia face a rapidly escalating HIV epidemic driven by injection drug use (IDU). We evaluate the role of opioid substitution treatment (OST) in engaging HIV-infected people who inject drugs (PWID) in care and the effect of OST on utilization of medical services.

**Methods:**

Cross-sectional study of healthcare utilization in the past six months among 296 randomly sampled HIV-infected opioid-dependent PWID conducted in healthcare clinics in 2010 across Ukraine. Participants categorized as therapeutic on OST if on OST for at least three consecutive months prior to the past six months or as not taking OST if not on any OST in the past nine months. Based on this criterion, 24 individuals were excluded.

**Results:**

The 65% on OST (177/272) were less likely to be below the poverty line or live alone and more likely to be married or have gone to prison (p<0.05). The two groups did not differ significantly in terms of age, gender, or education. Those on OST had more years of opioid injection but were less likely to have injected in the past 30 days, to have engaged in poly-substance abuse, or to have ever overdosed on drugs (p<0.01). In the past six months, those on OST were less likely to seek emergency care (72% vs. 84%, p<0.05) and had fewer mean emergency care visits (2.77 vs. 4.57, p<0.02) with no significant differences in mean ambulatory visits (1.78 vs. 0.59, p=0.11) or hospitalizations (0.53 vs. 0.34, p=0.36). Those on OST were more likely to be engaged in HIV care, as evidenced by higher rates of antiretroviral therapy ART (37% vs. 26%, p=0.08), recent CD4 testing (82% vs. 60%, p<0.01), and recent TB testing (95% vs. 71%, p<0.01). Number of self-reported symptoms was higher in the non-OST group compared to those on OST (10.46 vs. 7.75, p<0.01). Limitations include cross-sectional design and potential for recall and social desirability biases.

**Conclusions:**

Despite higher rates of incarceration and more years of opioid injection, those therapeutic on OST were less likely to seek emergency care than those not on OST and more likely to be engaged in HIV care with fewer overall symptoms.

# Sub article: The effects of opioid substitution treatment and highly active antiretroviral therapy on the cause-specific risk of mortality among injection drug using people living with HIV/AIDS

**DOI:** 10.7448/IAS.18.5.20436

**Published:** 2015-07-22

**Authors:** Bohdan Nosyk, Jeong Eun Min, Elizabeth Evans, Libo Li, Lei Liu, Viviane Lima, Evan Wood, Julio Montaner

**Affiliations:** 1 BC Centre for Excellence in HIV/AIDS, Vancouver, Canada; 2 Faculty of Health Sciences, Simon Fraser University, Burnaby, Canada; 3 UCLA Integrated Substance Abuse Programs, University of California Los Angeles, Los Angeles, United States; 4 Northwestern University, Feinberg School of Medicine, Chicago, United States; 5 Division of Aids, Faculty of Medicine, University of British Columbia, Vancouver, Canada

## Abstract

**Background:**

Prior studies indicate that opioid substitution treatment (OST) reduces the risk of mortality and improves the odds of accessing highly active antiretroviral therapy (HAART), however the relative effects of these treatments for injection drug using people living with HIV/AIDS (PLHIV) are unclear. We aim to determine the independent and joint effects of OST and HAART on mortality, by cause, within a population of injection drug using PLHIV initiating HAART.

**Methods:**

We used a linked population-level administrative database for British Columbia, Canada (1996–2010) to form a cohort of injection drug using PLHIV. We selected all individuals identified as HIV-positive and either having a history of OST at initial HAART receipt, as indicated by methadone or buprenorphine dispensation records in the BC PharmaNet database or having an indication of injection drug use before HIV infection, as indicated in the HIV testing database. We employed time-to-event analytic methods, including competing risks models, proportional hazards models with time-varying covariates, and marginal structural models, to identify the independent and joint effects of OST and HAART on all-cause, as well as drug- and HIV-related mortality, controlling for covariates.

**Results:**

Among 1727 injection drug using PLHIV, 493 (28.5%) died during a median 5.1 years (interquartile range: 2.1–9.1) of follow-up: 18.7% due to drug-related causes, 55.8% due to HIV-related causes, and 25.6% due to other causes. Standardized mortality ratios were 12.2 (95% CI: 9.8, 15.0) during OST, and 30.0 (27.1, 33.1) during periods out of OST. Both OST (adjusted hazard 0.34 (95% CI: 0.23, 0.49)) and HAART (0.39 (0.31, 0.48)) decreased the hazard of all-cause mortality; however, individuals were at lowest risk of death when these medications were used jointly (0.16 (0.10, 0.26)). Both OST and HAART independently protected against not only HIV-related death, but also drug-related death and death due to other causes.

**Conclusions:**

While both OST and HAART are life-saving treatments, there is an urgency to ensure joint administration to protect against both drug and HIV-related mortality.

# Sub article: Assessing the HIV prevention potential of Mexico's “narcomenudeo” drug law reform: implementation challenges among people who inject drugs

**DOI:** 10.7448/IAS.18.5.20437

**Published:** 2015-07-22

**Authors:** Leo Beletsky, Patricia Gonzalez-Zuniga, Gudelia Rangel, Dan Werb, Jaime Arredondo, Steffanie A Strathdee

**Affiliations:** 1 School of Law, Northeastern University, Boston, United States; 2 Global Public Health, School of Medicine, University of California, San Diego, La Jolla, United States; 3 United States-Mexico Border Commission, Tijuana, Mexico

## Abstract

**Background:**

Mexico's innovative 2009 “narcomenudeo” law decriminalized small-scale drug possession, mandating drug treatment diversion in lieu of incarceration and reframing drug policy to facilitate HIV prevention. However, the US-Mexico Border region continues to experience elevated HIV risk related to syringe sharing, while evidence-based addiction treatment and other prevention services targeting people who inject drugs (PWID) remain critically under-resourced. We designed a longitudinal cohort study to assess the implementation of this structural intervention among at-risk PWID in Tijuana.

**Methods:**

This mixed-methods research programme integrated a structured questionnaire and laboratory testing with qualitative interviews assessing legal knowledge, police encounters, drug and sex risk behaviours, and infectious disease status. At baseline, 737 PWID were recruited in Tijuana; 32 participated in qualitative interviews.

**Results:**

Between 2010 and 2013, only 11% of PWID respondents reported being aware of drug decriminalization; virtually none experienced drug treatment diversion or the law's other operational components. Interviews underscored the law's irrelevance to PWID; 699 (98%) characterized police practices as typically inconsistent with formal law. Instead of diversion to addiction treatment, multivariate modelling suggested that police encounters are independently associated with increased HIV risk behaviours such as syringe sharing (OR=1.26; 95% CI=1.09–1.46) and poly-drug use (OR=2.11; 95% CI=1.38–3.22). Qualitative data underscored the dissonance between the formal legal standards for drug and syringe possession, treatment diversion and other public health-oriented legal provisions on the one hand, and the lived experience of drug users on the other. Interviews mapped out a number of pathways by which arbitrary police enforcement severely undermine drug users’ ability to engage in protective HIV behaviours. Mixed-methods findings reveal that, just as housing instability can aggravate HIV risk, the lack of predictability in one's legal environment—also known as a “weak rule of law”—can compound HIV risk.

**Conclusions:**

Formal drug policy reform may be necessary in many settings to reduce HIV risk among PWID, but appears insufficient as a stand-alone intervention. As policy interventions intended to facilitate HIV prevention gain global momentum, ancillary structural reforms such as police training to improve the rule of law are needed to unlock their public health potential. Operational partnerships with law enforcement are discussed.

# Sub article: Low threshold services for females who inject drugs: reducing gender inequities in methadone enrolment

**DOI:** 10.7448/IAS.18.5.20438

**Published:** 2015-07-22

**Authors:** Barrot H Lambdin, Cassian Nyandindi, Doug Bruce, Norman Sabuni, Ayoub Magimba, Eva Matiko

**Affiliations:** 1 Pangaea Global AIDS, Oakland, United States; 2 Department of Epidemiology and Biostatistics, University of California, San Francisco, United States; 3 Department of Global Health, University of Washington, Seattle, United States; 4 Ministry of Health and Social Welfare, Dar es Salaam, Tanzania, United Republic of Tanzania; 5 Department of Medicine, Yale University, New Haven, United States; 6 Centers for Disease Control and Prevention, Dar es Salaam, Tanzania, United Republic of Tanzania

## Abstract

**Background:**

In 2011, the government of Tanzania established methadone assisted therapy (MAT) to combat the dual epidemic of HIV and injection drug use. However, enrolment of females who inject drugs into MAT has lagged behind that of males. To address this inequity, the methadone clinic at Mwananyamala Regional Referral Hospital (MRRH) introduced low threshold services for females in January 2013, allowing women to bypass the historically required attendance at community-based organizations prior to enrolment. Furthermore, existing female clients were encouraged to recruit their peers and one-day of the week was set aside for enrolling female clients only.

**Methods:**

We conducted an interrupted time-series study to evaluate the impact of implementing low threshold services for females enrolling into MAT, using de-identified, routinely collected data from November 2012 to October 2014 at MRRH. Prais-winsten regression models were utilized to estimate the mean change in the proportion of clients that were female and the weekly number of females enrolling, adjusting for male enrolment and a period of MAT enrolment interruption form July–November 2013.

**Results:**

Overall, 759 clients enrolled into the methadone clinic during the study period. Of those enrolling, the mean age was 34 years. The mean number of people enrolling into methadone during the study period was 8 clients (95% CI: 7, 9) per week. After implementation of low threshold services, the proportion of female clients increased from 14% (95% CI: 13%, 15%) to 24% (95% CI: 23%, 25%; p=0.001), but after the enrolment interruption, the proportion of female methadone clients decreased slightly to 22% (21–22%).

Adjusting for male enrolment, the mean number of females enrolling per week was 2 (95% CI: 1–3; p=0.001) people per week higher as compared to before implementation. Following the enrolment stoppage, the average number of female enrollees was comparable to pre-intervention (mean change: 0; 95% CI:–1, 1; p=0.442).

**Conclusions:**

Implementation of low threshold services improved enrolment into the methadone programme among women, thereby increasing the proportion of female methadone clients. However, the gains in enrolment were attenuated after an enrolment interruption, highlighting the importance of programme stability with this group of clients.

**Abstract MOPDD0104–Figure 1:** Lowess smooth of the percentage of female methadone clients.

# Sub article: Increasing rates of earlier antiretroviral treatment associated with elevated levels of optimal virologic response among HIV-positive illicit drug users during a treatment-as-prevention-based initiative in a Canadian setting

**DOI:** 10.7448/IAS.18.5.20564

**Published:** 2015-07-22

**Authors:** M-J Milloy, Thomas Kerr, Robert Hogg, Silvia Guillemi, Julio Montaner, Evan Wood

**Affiliations:** British Columbia Centre for Excellence in HIV/AIDS, Vancouver, Canada

## Abstract

**Introduction:**

Among illicit drug users, renewed efforts to reduce high levels of HIV/AIDS-related morbidity and mortality and curb rates of viral transmission rely, in part, on earlier initiation of antiretroviral therapy (ART). However, there are concerns that starting treatment prior to immunosuppression for members of harder-to-treat groups could contribute to lower levels of treatment adherence and lead to impaired virologic response. Thus, we sought to evaluate trends in CD4 cell count at ART initiation over time and rates of subsequent virologic response among HIV-positive illicit drug users during a community-wide Treatment-as-Prevention campaign in Vancouver, Canada.

**Methods:**

We used data from the ACCESS study, an ongoing longitudinal cohort of HIV-positive illicit drug users linked to comprehensive HIV clinical monitoring and pharmacy dispensation records. In this retrospective study, we included all individuals who initiated ART from 2005 onwards. We used multivariable logistic regression to evaluate differences in mean CD4+ cell count at initiation by year of initiation. To estimate time to plasma HIV-1 RNA viral load <50 copies/mL by CD4 cell count at ART initiation, we used Kaplan–Meier and Cox proportional hazards methods.

**Results:**

Between 2005 and 2013, 357 individuals initiated ART. Median CD4 at initiation increased from 130 cells/mL (interquartile range: 60 – 205) in 2005 to 330 (205 – 430) in 2013. In a linear regression analysis adjusted for age, gender and ancestry, year of initiation was positively associated with CD4 cell count at initiation (b = 30.82 cells per year increase, p < 0.001). Among 357 initiates, 184 (52%) reached non-detectable plasma VL within 360 days. In an adjusted Cox proportional hazards model, CD4 cell count at initiation was positively associated with time to viral suppression (adjusted hazard ratio: 1.21 per 100 cell/mL increase; 95% confidence interval: 1.13–1.29).

**Conclusions:**

We observed substantial increases in CD4 cell count at initiation over time coincident with a community-wide TasP-based initiative. Individuals initiating ART earlier in the disease course exhibited higher rates of optimal virologic response. These findings support earlier initiation of ART among illicit drug users to reduce levels of HIV/AIDS-associated morbidity and mortality and rates of viral transmission.

# Sub article: Association between CSF and peripheral markers of immune-activation/inflammation and elevated intrathecal HIV-RNA levels in a cohort of HIV-infected antiretroviral naïve individuals

**DOI:** 10.7448/IAS.18.5.20439

**Published:** 2015-07-22

**Authors:** Esther Merlini, Francesca Iannuzzi, Francesca Bai, Mattia Trunfio, Stefano Bonora, Andrea Calcagno, Elvira Stefania Cannizzo, Matteo Basilissi, Teresa Bini, Antonella d'Arminio Monforte, Giulia Marchetti

**Affiliations:** 1 Clinic of Infectious Diseases and Tropical Medicine, University of Milan, Health Sciences, Milan, Italy; 2 Department of Infectious Diseases, University of Turin, Turin, Italy

## Abstract

**Introduction:**

Since the association between high HIV-RNA replication in central nervous system and immune activation/inflammation has not yet been established, we aimed to investigate the inflammatory *milieu* in CSF and peripheral blood of HIV+ antiretroviral-naïve subjects with high CSF viremia compared to those with low CSF viremia, in the attempt to identify biomarkers that might be used as diagnostic tools.

**Methods:**

A total of 150 HIV+ cART-naïve pts underwent to lumbar puncture for CSF HIV-RNA quantification and were tested for peripheral T-cell immune-phenotypes (CD38/CD45RA/CD45R0/CD127 on CD4/CD8; flow cytometry). In a subgroup of 64 patients CSF/plasma TNF-a, IL-6, sCD14, IFNg, MCP-1, IP-10, neopterin, S100beta (ELISA, Luminex) were measured. We defined: high CSF HIV-RNA ≥10,000 cp/mL (H-CSF), low CSF HIV-RNA <10,000 cp/mL (L-CSF), viral escape (VE) CSF/plasma HIV-RNA >1 log_10_ cp/mL. Statistical analyses: Chi-square, Mann–Whitney test and univariate/multivariate logistic regression.

**Results:**

48/150 pts (32%) resulted H-CSF. VE was found in 5/150 pts (3%). No differences in gender, risk exposure categories, viral hepatitis co-infections, HIV duration, age and CD4+ nadir were found between L-CSF and H-CSF. H-CSF pts displayed higher plasma HIV-RNA (p=0.002) and VE (p=0.019). The univariate logistic regression showed that H-CSF are characterized by lower central memory CD127+CD4% (p=0.026) and naïve CD8+CD45RA% (p=0.017) and higher activated CD8+CD38% (p=0.08) and memory activated CD8+CD38+CD45R0% (p=0.021). In multivariate analysis, lower proportion of CD8+CD45RA% was the only parameter independently associated with H-CSF (AOR 0.934, IC 95% 0.877–0.995, p=0.035), adjusting for plasma VL, CD4/CD8 ratio, CD127/CD4%, CD8/CD38%. Within the CSF, we found that H-CSF displayed significantly higher sCD14 (p<0.0001), neopterin (p=0.006), IL-6 (p=0.002) and IP-10 (0.035) and no differences in TNFa, MCP-1 and S100beta. Similarly, H-CSF showed higher circulating sCD14 (p<0.0001), but not TNFa, IL-6 and IFNg.

**Conclusions:**

The low percentage of naïve CD8+ T-cells, independently associated with higher CSF Viral Load, might be included in a panel of biomarkers useful to identify patients at major risk of high CSF replication, if confirmed by larger studies.

Besides, the finding of higher peripheral and CSF activation/inflammation in H-CSF group indicate a more complex scenario, where both districts cooperate in maintaining the inflammation within CNS, possibly affecting neuronal function, and therefore deserves further investigations.

# Sub article: Receptor mediated endocytosis directs subcellular trafficking and TLR signalling of HIV-1 in plasmacytoid dendritic cells

**DOI:** 10.7448/IAS.18.5.20440

**Published:** 2015-07-22

**Authors:** Meagan O'Brien, Olivier Manches, Craig Wilen, Vernon Wu, Nicole Sunseri, Nina Bhardwaj

**Affiliations:** 1 Department of Medicine, Icahn School of Medicine at Mount Sinai, New York, United States; 2 Department of Infectious Diseases, Icahn School of Medicine at Mount Sinai, New York, United States; 3 Laboratory and Genomic Medicine, Washington University School of Medicine, St. Louis, United States; 4 Department of Pediatrics, University of Chicago, Chicago, United States

## Abstract

**Introduction:**

Dysregulated type I interferon (IFN) responses contribute to immunopathology in chronic HIV infection, therefore it is critical to dissect the molecular mechanisms underlying HIV-stimulated IFN production. We examined the spatiotemporal regulation of IFN secretion by plasmacytoid dendritic cells (pDC), specialized cells that secrete high levels of IFN upon HIV recognition by Toll-like receptor (TLR) 7. We showed previously that intracellular trafficking of HIV to early endosomes is associated with potent IFN secretion but minimal NF-kB signalling, resulting in suboptimal pDC maturation; however, how HIV trafficking is determined and the causal link between HIV subcellular localization and differential TLR signalling are currently unknown.

**Methods:**

Human pDC were purified from peripheral blood and were stimulated with GFP labelled: HIV, HIV pseudotyped with influenza hemagglutinin envelope (HA-HIV), and PR8 influenza. TLR7 expressing HEK NF-kB reporter cells, stably transfected with CD4 mutants with cytoplasmic tails directing trafficking to early endosomes (EE) or lysosomes, were activated with HIV and controls. Analysis included ELISA, flow cytometry and florescent microscopy. Cells were imaged using the Advanced Precision imaging system and images were analyzed using ImageJ.

**Results:**

We compared the effects and spatiotemporal trafficking in pDC of HIV, influenza and HA-HIV. We demonstrate that HA-HIV strongly activates maturation pathways (NF-kB) in pDC and traffics rapidly to lysosomes, similarly to influenza but unlike HIV, suggesting that viral envelope directs trafficking and resultant phenotype of ssRNA virions in pDC. We studied HIV-CD4 interactions in a HEK reporter cell system expressing TLR7 with functional NF-kB signalling, which we co-transfected with CD4 mutants whose cytoplasmic tails either directed CD4 trafficking to EE or lysosomes. We show that wild type (WT) CD4 localizes to EE, whereas CD4 mutated with either DEC-205 or LAMP1 tail localizes to lysosomes. HIV traffics to EE in WT CD4 expressing TLR7 HEK cells and fails to stimulate NF-kB signalling, whereas HIV traffics to lysosomes in DEC-205/LAMP1 expressing TLR7 HEK cells and stimulates NF-kB signalling, suggesting that rerouting of HIV (via CD4) to lysosomal compartments triggers NF-kB rather than IFN pathways.

**Conclusions:**

CD4 receptor mediated endocytosis targeting early endosomes determines HIV intracellular localization and observed interferon-producing phenotype of HIV-activated pDCs.

# Sub article: HIV-1 Vpu exploits the crosstalk between BST2 and the ILT7 receptor to inhibit innate sensing of infected T cells by plasmacytoid dendritic cells

**DOI:** 10.7448/IAS.18.5.20441

**Published:** 2015-07-22

**Authors:** Mariana G Bego, Édouard A Côté, Nick Aschman, Johanne Mercier, Winfried Weissenhorn, Éric A Cohen

**Affiliations:** 1 Institut de Recherches Cliniques de Montréal, Montréal, Canada; 2 Unit of Virus Host Cell Interactions, Université Grenoble Alpes, Grenoble, France; 3 Department of Microbiology, Infectiology and Immunology, Université de Montréal, Montreal, Canada

## Abstract

**Introduction:**

Plasmacytoid dendritic cells (pDCs) constitute a major source of type-I interferon (IFN-I) production during acute HIV infection. Their activation results primarily from TLR7-mediated sensing of HIV-infected cells. BST2/Tetherin is a restriction factor that suppresses HIV release by cross-linking virions at the cell-surface. HIV-1 overcomes BST2 antiviral activity through Vpu, which partially downregulates BST2 cell-surface expression. Apart from its direct antiviral activity, BST2 was shown to bind the ILT7 pDC-specific inhibitory receptor and repress IFN-I production by activated pDCs. Here, we examined whether Vpu-mediated BST2 antagonism could modulate innate sensing of HIV-infected cells by pDCs.

**Methods:**

PBMCs or isolated pDCs were co-cultured with T cells infected with wild type or Vpu-defective HIV-1 and innate sensing was evaluated by monitoring IFN-I production. BST2-mediated activation of ILT7 signalling was analyzed using an ILT7-reporter cell system.

**Results:**

We show that Vpu attenuates the production of IFN-I during sensing of HIV-1 infected cells by pDCs. This control of innate sensing by Vpu could be prevented by: 1) depletion of BST2 from infected donor cells; 2) depletion of ILT7 in pDCs; or 3) blocking BST2-ILT7 interaction using anti-BST2 antibodies or soluble ILT7. Using a BST2 mutant that cannot cross-link budding virions but yet retains the capacity to repress IFN-I production by pDCs, we show that virion trapping on infected donor cells prevents BST2 from eliciting an inhibition of IFN-I production by pDCs. Interestingly, confocal microscopy analysis of virus producing cells reveals that in presence of Vpu there is a residual pool of surface BST2, which is excluded from viral budding sites and thus potentially accessible for interaction with ILT7 on pDCs. Lastly, using an ILT7 reporter cell system, we provide evidence that Vpu-mediated BST2 antagonism modulates the levels of available surface BST2 capable of engaging and activating ILT7 upon cell-to-cell contact.

**Conclusions:**

Overall, this study sheds light on a novel Vpu-BST2 interaction that allows HIV to control innate sensing of infected cells by pDCs via the negative signalling exerted by the ILT7-BST2 pair. This mechanism of innate immune evasion is likely to be critical for efficient viral dissemination and establishment of viral reservoirs during acute infection.

# Sub article: HIV-1 transcriptional silencing caused by TRIM22 inhibition of Sp1 binding to the promoter

**DOI:** 10.7448/IAS.18.5.20442

**Published:** 2015-07-22

**Authors:** Filippo Turrini, Anna Kajaste-Rudnitski, Sara S Marelli, Carine Van Lint, Atze T Das, Ben Berkout, Elisa Vicenzi

**Affiliations:** 1 Viral Pathogens and Biosafety Unit, Division of Immunology, Transplantation and Infectious Diseases, San Raffaele Scientific Institute, Milan, Italy; 2 Molecular Medicine PhD Program, Vita-Salute San Raffaele University, Milan, Italy; 3 Service of Molecular Virology, University of Bruxelles, Gosselies, Belgium; 4 Center for Infection and Immunity Amsterdam, Academic Medical Center, University of Amsterdam, Amsterdam, Netherlands

## Abstract

**Introduction:**

HIV-1 latency is a multifactorial process resulting by the interplay between cellular transcription factors and the viral regulatory protein Tat. We have previously described the interferon-inducible restriction factor TRIM22 as a suppressor of basal and phorbol ester-dependent LTR-mediated transcription independently of NF-kB and of Tat/TAR interaction. As basal HIV-1 transcription is mainly driven by the binding of the cellular transcription factor Sp1, we have investigated whether TRIM22 could interfere with such Sp1-driven transcriptional activation of HIV-1 LTR.

**Methods:**

293T cells, lacking of endogenous TRIM22, were co-transfected with a TRIM22-expressing plasmid together with reporters vectors driven by the HIV-1 promoter containing either wild-type or mutated Sp1 binding sites or lacking of either one or two sites; reporter expression was assessed 48 hours post-transfection. Endogenous TRIM22 was knocked-down (KD) in SupT1 cells that were subsequently infected with HIV-1 molecular clones engineered to be dependent on an incorporated Tet-On gene expression system for activation of transcription while being independent of Tat/TAR interaction. Virus replication was monitored up to 32 days post-infection. Cell extracts from TRIM22-transfected 293T was subjected to 1) immunoprecipitation, 2) Western blotting, 3), DNA pull-down and 4) chromatin immunoprecipitation (ChIP).

**Results:**

TRIM22 overexpression suppressed Sp1-driven transcription of HIV-1, as its inhibitory activity was lost in the absence of Sp1 binding sites. In contrast, TRIM22 KD increased the replication of infectious clones that were exclusively dependent upon Sp1 binding to the promoter. Furthermore, immunoprecipitation experiments showed that TRIM22 and Sp1 can interact physically although this interaction does not affect the level of expression of endogenous Sp1 or its phosphorylation state. TRIM22 did not directly bind to the HIV-1 LTR by either *in vitro* pull-down experiments or in ChIP experiments, however TRIM22 expression drastically prevented the binding of Sp1 to the HIV-1 LTR.

**Conclusions:**

TRIM22 inhibits Sp1-dependent transcription by interacting with Sp1 and preventing its binding to the HIV-1 LTR. Our findings bear relevance for the discovery of new pharmacological approaches aimed at targeting the reservoir of cells latently infected with replication-competent proviruses.

# Sub article: Polymorphisms in TRIM22 are associated with HIV-2 acquisition and disease progression

**DOI:** 10.7448/IAS.18.5.20443

**Published:** 2015-07-22

**Authors:** Shmona Simpson, Thushan deSilva, Louis-Marie Yindom, Alexandra Leligdowicz, Timothy Vincent, Sarah Rowland-Jones

**Affiliations:** 1 Nuffield Department of Medicine, University of Oxford, Oxford, United Kingdom; 2 Medical Research Council Unit, The Gambia, Serrekunda, Gambia

## Abstract

**Introduction:**

Tripartite motif-containing protein 22 (TRIM22) is an E3 ubiquitin ligase with activity against HIV-1: high levels of TRIM22 expression are associated with reduced viral set-point following acute HIV-1 infection. The TRIM22 gene has been greatly shaped by positive selection, and its expression is sensitive to retroviral infection, Type I and Type 2 interferon. The mechanism by which TRIM22 exerts its antiviral effect is poorly understood. Further, the impact of TRIM22 genetic variation in the context of HIV-2 disease is unknown.

**Methods:**

To test the hypotheses that TRIM22 expression antagonizes HIV-2 infection and that polymorphisms in TRIM22 significantly modulate this effect, we conducted three studies. Firstly, TRIM22 was genotyped in 60 HIV-2 patients, comparing viral controllers and rapid progressors, and a similar number of age and sex matched controls from the same community in rural Guinea-Bissau. Using regression modelling, polymorphisms were analysed alongside immunological and virological data. Secondly, a model of TRIM22 was constructed using computational methods and the polymorphisms observed *in vivo* were mapped and analysed. Finally, baseline cDNA and protein levels of TRIM22 from C8166 cells were measured using quantitative RT-PCR and flow cytometry respectively. The cells were subsequently infected with HIV-2, and measurements repeated to determine whether TRIM22 gene expression is sensitive to HIV-2 infection.

**Results:**

The data show that TRIM22 polymorphisms rs1063303 and rs7935564 are significantly associated with HIV-2 acquisition and disease progression. Further, polymorphisms observed *in vivo* cluster in functional regions that our modelling studies suggest may interact with the HIV-2 capsid. Finally, we show that TRIM22 gene expression is upregulated in the presence of HIV-2, in a lymphocyte cell line.

**Conclusions:**

Taken together, our data show that TRIM22 expression is sensitive to HIV-2 infection and that polymorphisms in TRIM22 genes are significantly associated with HIV-2 acquisition and disease progression. Further the study has computationally characterized positively selected polymorphisms observed *in vivo* and the data show that these polymorphisms have the potential to significantly alter protein structure and function. These data provide the first analysis of TRIM genetic variation in the context of HIV-2 infection.

# Sub article: The negative checkpoint receptor TIGIT marks exhausted T cells during SIV infection and correlates with SIV disease progression

**DOI:** 10.7448/IAS.18.5.20561

**Published:** 2015-07-22

**Authors:** Gabriela M Webb, Glen M Chew, Tsuyoshi Fujita, Benjamin J Burwitz, Helen L Wu, Jason S Reed, Katherine B Hammond, Scott G Hansen, Mark Maurer, Alan J Korman, Lishomwa C Ndhlovu, Jonah B Sacha

**Affiliations:** 1 Vaccine & Gene Therapy Institute, Oregon National Primate Research Center, Oregon Health & Science University, Beaverton, United States; 2 Department of Tropical Medicine, Hawaii Center for HIV/AIDS, John A. Burns School of Medicine, University of Hawaii, Honolulu, United States; 3 Department of Microbiology and Immunology, Tohoku University Graduate School of Medicine, Sendai, Japan; 4 Biologics Discovery California, Bristol-Myers Squibb, Redwood City, United States

## Abstract

**Introduction:**

During chronic viral infections, high antigenic load continually stimulates T cells resulting in T-cell exhaustion. Exhausted T cells increase the expression of negative checkpoint inhibitors such as PD-1, which raise the threshold for activation and contribute to suppressed immune responses. Another recently discovered immune checkpoint receptor, TIGIT, is upregulated on T cells in neoplasms and chronic LCMV infection. We hypothesize that TIGIT functions as a negative checkpoint receptor marking dysfunctional T cells during SIV infection and that modulation of TIGIT would restore anti-SIV-specific T-cell responses.

**Methods:**

Spleen, lymph node (LN) and PBMCs from SIV-naïve and SIV-infected rhesus macaques (RMs) were examined for surface expression of TIGIT. *In vitro* cytokine production was assessed via intracellular cytokine staining. Proliferative capacity was determined through CFSE dilution assays in the presence of antibodies blocking TIGIT and PD-1 pathways (anti-TIGIT mAb and anti-PD-L1 mAb).

**Results:**

TIGIT expression was significantly upregulated on CD8^+^ T cells derived from the spleen and LN but not on PBMC in SIV-infected animals. The frequency of TIGIT^+^ CD8^+^ T cells in the LN significantly correlated with SIV viral load, and TIGIT expression was driven primarily by g-chain cytokines such as IL-2. TIGIT was expressed on approximately 40% of SIV-specific CD8^+^ T cells, even in animals with full cART suppression of viral replication. While Ki-67 expression did not differ between TIGIT^+^ and TIGIT^−^ CD8^+^ T cells, TIGIT^-^ CD8^+^ T cells produced significantly more IFN-g compared to TIGIT^+^ CD8^+^ T cells. Single and dual blockade of TIGIT and/or PD-1 signalling pathways restored proliferative capacity of SIV-specific T cells *in vitro*.

**Conclusions:**

TIGIT is a negative checkpoint receptor that marks a novel population of functionally exhausted SIV-specific CD8^+^ T cells and is associated with SIV disease progression. The enhancement of virus-specific T-cell proliferative responses in the presence of single or dual blockade of TIGIT and/or PD-1 suggests that targeting the TIGIT pathway is a viable therapeutic approach to reverse T-cell dysfunction. Given the high sequence homology of rhesus and human TIGIT, this provides a platform to further investigate TIGIT, along with other checkpoint inhibitors, as potential targets for mediating a functional cure for HIV.

# Sub article: Prolongation of QTc interval in HIV-infected individuals compared to the general population is not caused by antiretroviral therapy

**DOI:** 10.7448/IAS.18.5.20444

**Published:** 2015-07-22

**Authors:** Stefan Esser, Marie Henrike M Geisel, Marina Arendt, Christina Schulze, Volker Holzendorf, Anna Warnke, Norbert H Brockmeyer, Martin Hower, Dirk Schadendorf, Till Neumann, Lewin Eisele, Raimund Erbel, Susanne Moebus, Karl-Heinz Jöckel, Nico Reinsch, HIV HEART Study Group and Heinz Nixdorf Recall Investigative Group

**Affiliations:** 1 Department of Dermatology and Venerology, University of Duisburg Essen, Essen, Germany; 2 Institute for Medical Informatics, Biometry and Epidemiology (IMIBE), University of Duisburg Essen, Essen, Germany; 3 Department of Cardiology, West German Heart Centre, University of Diusburg Essen, Essen, Germany; 4 Clinical Trial Centre Leipzig, University Leipzig, Coordination Centre for Clinical Trials (ZKS Leipzig – KKS), Leipzig, Germany; 5 Department of Sexual Health and Medicine, Clinic of Dermatology, Venerology and Allergology, Ruhr University Bochum, Bochum, Germany; 6 Department of Internal Medicine, City Hospital Dortmund, Dortmund, Germany

## Abstract

**Introduction:**

Prolongation of the QTc interval (QTc) increases the risk of cardiovascular events (CVE). The incidence of CVE is higher in HIV-infected (HIV+) patients compared with the general population. The impact of different antiretroviral therapies (ART), co-medication and HIV-infection on the electrical activity of the heart is rarely investigated in large HIV+ cohorts.

**Methods:**

We compare QTc of HIV+ outpatients of the HIV HEART study (HIVH) and of controls of the population-based Heinz Nixdorf Recall study (HNR), both recruited from the German Ruhr area since 2000. HIVH cases were age- and sex-matched with HNR controls in a 1:2 ratio. QTc was measured and corrected using the Bazett's formula. We used crude and adjusted linear mixed models to account for the matched design and adjusted for QTc interval prolonging medication (QTc-PM, no ART). Differences in QTc between HIV specific factors and ART were evaluated using ANOVA in the HIVH subpopulation. All analyses were stratified by sex.

**Results:**

496 HIVH participants (83.3% male, aged 54.5±6.7) were matched with 992 HNR controls. We observed a longer QTc in HIVH subjects compared with HNR controls: 424±23 ms versus 411±15 ms for male and 435±20 ms versus 416±17 ms for female subjects (p<0.0001 for both sexes). HIVH males used QTc-PM more often (22.3% vs. 17.6% for HNR) than HIVH females (13.3% vs. 24.7% for HNR). However, adjusting for QTc-PM the mean differences in QTc remained significant with 13 (95% CI: 11, 15) ms for male and 19 (95% CI: 14, 24) ms for female subjects. Prolongation of QTc (male >440 ms, female >460 ms) was pathologic in 22.8% versus 3.9% of HIVH and HNR males and in 12.1% versus 1.8% of the females. No differences in the QTc were observed within the HIVH population for different ART medications, for the clinical and immunological HIV status and for the route of HIV infection in both sexes.

**Conclusions:**

HIV+ patients have longer mean QTc and more often pathological prolonged QTc compared with age- and sex-matched controls from the general population even after adjustment for intake of non-antiretroviral QTc-PM. ART, HIV stage and HIV transmission route are not associated with QTc prolongation.

# Sub article: Favourable effect on vitamin D and bone after switching from Atripla to darunavir/ritonavir: a randomised controlled clinical trial

**DOI:** 10.7448/IAS.18.5.20445

**Published:** 2015-07-22

**Authors:** Lisa Hamzah, Juan Manuel Tiraboschi, Martina Toby, Helen Iveson, Christine Mant, Cason John, Keith Burling, Ranjababu Kulasegaram, Alasdair Teague, Frank A Post, Julie Fox, MIDAS Study Group

**Affiliations:** 1 Renal Sciences, King's College London, London, United Kingdom; 2 Department of Nuclear Medicine, Guys and St Thomas’ NHS Foundation Trust, London, United Kingdom; 3 Core Biochemical Assay Laboratory, Cambridge University NHS Foundation Trust, Cambridge, United Kingdom; 4 King's Centre for Global Health, King's College Hospital NHS Foundation Trust, London, United Kingdom

## Abstract

**Introduction:**

Efavirenz has been associated with reductions in vitamin D (25[OH]D) and Tenofovir with increased bone turnover, reductions in bone mineral density (BMD) and renal tubular dysfunction (RTD). We hypothesized that switching from Atripla to Darunavir/Ritonavir monotherapy (DRV/r) might increase 25[OH]D, and improve BMD and RTD.

**Methods:**

Patients with HIV RNA <50 copies/mL on Atripla for ≥ six months were randomized 1:1 to receive ongoing Atripla or DRV/r (800/100 mg once daily) for 48 weeks. Primary endpoint was change from baseline in 25[OH]D at week 48. Secondary endpoints included changes in BMD, bone turnover markers and RTD. Linear regression estimated the mean difference in 25[OH]D in patients on Atripla versus DRV/r. Secondary endpoints were expressed as the mean (95% CI) observed between-arm difference from baseline.

**Results:**

70 subjects (86% male, 66% white, mean (SD) CD4 cell count 537.3 (191.5) per mm^3^) were randomized, of whom 26 (DRV/r) and 31 (Atripla) completed the 48 week study on the allocated treatment. The mean (SD) difference between baseline and week 48 25[OH]D was 5.0 (5.9) ng/mmol for DRV/r and 1.2 (6.0) for Atripla. After adjustment for baseline 25[OH]D and demographics, at week 48 DRV/r monotherapy was associated with a +3.5 (95% CI: 0.5, 6.4) ng/mmol increase in 25[OH]D compared to Atripla (p=0.02). Subjects in the DRV/r arm experienced increases in BMD (mean between-arm difference (0.02 (0.003, 0.04) g/cm^2^ at the lumbar spine, p=0.03, and 0.03 (0.006, 0.06) g/cm^2^ at the neck of femur, p=0.02), and reductions in parathyroid hormone (PTH) (−20.4 (−38.8, −2.0) ng/l, p=0.03), bone-specific alkaline phosphatase (−7.1 (−9.7, −4.5) IU/L, p<0.0001) and serum type 1 pro-collagen (−16.9 (−26.5, −7.4) ug/L, p=0.0008), as compared with subjects on Atripla. No significant difference in RTD (urine retinol-binding protein/creatinine ratio and phosphate reabsorption) was observed. Reasons for discontinuation in the DRV/r arm included side effects (n=4) and virus load rebound (n=2), all of which resolved with DRV/r discontinuation or regimen intensification.

**Conclusions:**

A switch from Atripla to DRV/r resulted in significant improvements in 25[OH]D and PTH, and a 2–3% increase in BMD. DRV/r monotherapy provides a bone-friendly treatment option to patients with osteoporosis or increased fracture risk.

# Sub article: Long-term bone mineral density changes in antiretroviral-treated HIV-infected individuals

**DOI:** 10.7448/IAS.18.5.20446

**Published:** 2015-07-22

**Authors:** Philip Grant, Doug Kitch, Grace McComsey, Ann Collier, Sue Koletar, Kristine Erlandson, Michael Yin, Benedetta Bartali, Belinda Ha, Kathy Melbourne, Todd Brown

**Affiliations:** 1 Infectious Diseases, Stanford University, Stanford, United States; 2 Center for Biostatistics in AIDS Research, Harvard School of Public Health, Harvard School of Public Health, Boston, United States; 3 Department for Pediatrics and Medicine, Case Western Reserve University, Cleveland, United States; 4 Division of Allergy and Infectious Diseases, University of Washington, Seattle, United States; 5 Division of Internal Medicine, Ohio State University, Colombus, United States; 6 Division of Infectious Diseases, University of Colorado, Denver, United States; 7 Division of Infectious Diseases, Columbia University, New York, United States; 8 New England Research Institute, Watertown, United States; 9 Viiv, Research Triangle Park, United States; 10 Medical Sciences, Gilead Sciences, Foster City, United States; 11 The Welch Center for Prevention, Epidemiology, and Clinical Research, Johns Hopkins University, Baltimore, United States

## Abstract

**Introduction:**

Accelerated bone mineral density (BMD) loss occurs during the first two years of ART. Few studies have evaluated subsequent BMD changes, especially compared to uninfected controls.

**Methods:**

ACTG A5318 performed one follow-up site-specific dual-energy x-ray absorptiometry (DXA) in HIV-infected individuals who had received baseline and follow-up DXAs during the randomized treatment trial A5202/A5224s. As controls, we obtained DXA results from uninfected participants enrolled in BACH/Bone and WIHS cohorts. Repeated measures analyses compared BMD change rate between HIV-infected and uninfected, adjusting for age, sex, race and body mass index (BMI). In the HIV-infected group, we performed multivariable analyses evaluating association of HIV-specific (baseline and time-updated CD4 and viral load), HIV treatment-related (randomized ART regimen, cumulative tenofovir (TDF) exposure) and non-HIV related factors (age, sex, race, relevant concomitant medication use, BMI, total lean body mass) on BMD change rate.

**Results:**

Baseline characteristics between HIV infected (n=97) and HIV-uninfected (n=630) participants were generally similar: median age, 40 versus 46; % female, 14 versus 14; % black, 34 versus 35; median BMI, 24 versus 29; and median years between first and last DXA, 7.5 versus 6.9. Seventy-one percent of HIV-infected participants were on TDF at last DXA. Compared to controls, HIV-infected individuals had significantly greater adjusted BMD decline rate at lumbar spine (LS) and total hip (TH) during the first 96 weeks of ART (both p<0.001). Subsequently, on follow-up DXA, HIV infection remained significantly associated with greater adjusted BMD decline rate at LS (−0.29%/year; 95% CI: −0.49, −0.09; p=0.005) but not at TH (p=0.63). In the HIV group, the rate of BMD decline slowed after the first 96 weeks of ART (0–96 weeks vs. Late Change: LS: −0.75%/year vs. −0.19%/year, p=0.04; TH: −1.29%/year vs. −0.30%/year, p<0.001). During the late period, no HIV-related characteristic was associated with BMD loss, but lower total lean body mass (and not BMI) was associated with greater BMD loss at LS and TH (both p<0.001).

**Conclusions:**

Although the rate of BMD decline slowed after the first 96 weeks after ART initiation in HIV-infected persons, the rate of bone loss at the lumbar spine was still significantly greater than HIV-uninfected controls.

# Sub article: Prevalence of non-alcoholic fatty liver disease and liver fibrosis among perinatally HIV-infected Asian adolescents with history of transaminitis

**DOI:** 10.7448/IAS.18.5.20447

**Published:** 2015-07-22

**Authors:** Tavitiya Sudjaritruk, Torsak Bunupuradah, Linda Aurpibul, Pope Kosalaraksa, Nia Kurniati, Thanyawee Puthanakit

**Affiliations:** 1 Department of Pediatrics, Faculty of Medicine, Chiang Mai University, Chiang Mai, Thailand; 2 Research Institute for Health Sciences, Chiang Mai University, Chiang Mai, Thailand; 3 HIV-NAT, Thai Red Cross – AIDS Research Centre, Bangkok, Thailand; 4 Srinagarind Hospital, Khon Kaen University, Khon Kaen, Thailand; 5 Department of Child Health, Cipto Mangunkusumo General Hospital, Jakarta, Indonesia; 6 Department of Pediatrics, Faculty of Medicine, Chulalongkorn University, Bangkok, Thailand

## Abstract

**Introduction:**

Liver disease is an important non-AIDS related morbidity in HIV-infected adults. Non-alcoholic fatty liver disease (NAFLD) is a clinical-pathological syndrome which may progress toward liver fibrosis and cirrhosis. The study objective was to determine the prevalence of NAFLD and liver fibrosis among perinatally HIV-infected adolescents with a history of transaminitis.

**Methods:**

A cross-sectional study was conducted at 4 paediatric HIV centres in Thailand (Bangkok, Chiang Mai, Khon Kaen) and Indonesia (Jakarta). HIV-infected adolescents aged 10 to 25 years with virologic suppression and had transaminitis (ALT >30 U/L or AST >50 U/L) within past 12 months were enrolled. Adolescents with history of hepatitis B/C co-infection or significant alcohol consumption were excluded. The assessments included liver ultrasonography (USG-evaluation of fatty liver); transient elastography (TE-evaluation of liver stiffness), serum liver function test. Aspartate aminotransferase-to-platelet ratio index (APRI-biomarker of liver fibrosis) was calculated. Liver stiffness was defined as any liver fibrosis.

(TE ≥5.1 kPa) and significant liver fibrosis (TE ≥7.4 kPa). APRI >0.5 and >1.5 were defined as mild/moderate fibrosis and advanced fibrosis, respectively. Correlation of APRI and TE result was assessed.

**Results:**

From August to December 2014, 39 adolescents were enrolled. Median (IQR) age was 17.2 (14.6–19.4) years; 47% were male. Median (IQR) duration of ART was 7.8 (4.4–11.2) years, of which 54% currently received non-nucleoside reverse transcriptase (NNRTI)-based regimen. Median (IQR) current CD4 cells count was 691 (535–979) cells/mm^3^. Fatty liver was observed in 6 (15%) adolescents, of which 2 (5%) had severe fatty liver (Table1). Seventeen (46%) adolescents had any liver fibrosis and 6 (15%) had significant liver fibrosis (Table 1). Median (IQR) of ALT and AST were 30 (21–39) and 25 (20–31) U/L, respectively. Four (11%) had mild/moderate fibrosis by APRI. The APRI was moderately positively correlated with liver stiffness evaluated by TE (Pearson's correlation coefficient=0.51; p=0.001).

**Conclusions:**

About one-third of perinatally HIV-infected adolescents with a history of transaminitis met criteria of fatty liver or liver fibrosis. Longitudinal follow-up to monitor for progression and provide appropriate interventions in a timely manner is needed.

**Remark:**

This study is funded by CIPHER Grants (2014), International AIDS Society.

**Abstract TUPDB0104–Table 1:** Characteristics of perinatally HIV-infected adolescents with non-alcoholic fatty liver disease or liver fibrosis

Sex	Age (yrs)	BMI (kg/m^2^)	ALT (U/L)	AST (U/L)	Fatty Liver by USG	TE (kPa)	APRI
**M**	23	**36.2**	**160**	**87**	**Severe**	**14.0**	**0.63**
F	17	21.3	**36**	24	**Severe**	**5.7**	0.21
M	15	17.6	**36**	42	**Mild**	**5.9**	0.39
F	12	15.4	**36**	31	**Mild**	**5.7**	0.42
F	20	17.8	**46**	35	**Mild**	4.3	0.47
F	20	20.5	**71**	33	**Mild**	3.3	0.33
M	17	**25.8**	**50**	45	Normal	**8.6**	**0.60**
F	18	19.4	23	25	Normal	**8.0**	0.34
M	14	17.8	23	32	Normal	**7.9**	0.27
M	18	18.5	29	22	Normal	**7.8**	0.17
F	23	18.0	19	18	Normal	**7.7**	0.34

M=male; F=female; BMI=body mass index; ALT=alanine aminotransferase; AST=aspartate aminotransferase; USG=ultrasonography; TE=transient elastography; APRI=aspartate aminotransferase-to-platelet ratio index; The bold text indicates abnormal values for each test.

# Sub article: Fixed dose combination EVG/COBI/TDF/FTC does not affect insulin resistance: the STRIBILD-IR study

**DOI:** 10.7448/IAS.18.5.20448

**Published:** 2015-07-22

**Authors:** Christoph D Spinner, Kristina E Kern, Sebastian Noe, Alexander von Werder, Christiane Schwerdtfeger, Roland M Schmid, Alexander Zink, Eva Wolf, Roman Iakoubov

**Affiliations:** 1 Department of Medicine II, University Hospital Klinikum rechts der Isar, Munich, Germany; 2 Department of Dermatology and Allergology, University Hospital Klinikum rechts der Isar, Munich, Germany; 3 Clinical Research, Muc Research GmbH, Munich, Germany

## Abstract

**Introduction:**

The incidence of insulin resistance (IR) and diabetes mellitus in HIV-patients, both contributing to cardiovascular morbidity and mortality, has been associated with antiretroviral therapy (ART). Only limited data exists on metabolic effects of regimens including newer drugs such as fixed dose combination drugs, particularly concerning IR.

**Methods:**

In this prospective, open-label, randomized phase-I-study we investigated the effects of the recently available fixed dose combination of tenofovir disaproxil fumarate, emtricitabine, elvitegravir and cobicistat (TDF/FTC/EVG/cobi, group I) on IR, in comparison to established ART with TDF/FTC+lopinavir/ritonavir (LPV/r, group II) and TDF/FTC+darunavir/ritonavir (DRV/r, group III). N=30 healthy, male volunteers were randomly assigned into one of the 3 study arms. IR was measured using golden standard method of hyperinsulinemic eugylcemic clamp before and 14 days after initiation of study medication. Briefly, a constant insulin infusion (2 mIU/(kg*min)) was infused over two hours, glucose infusion was adjusted as necessary to achieve stable glucose levels (target 90±5 mg/dl). All volunteers took the study medication, as verified by pill counting. IR was evaluated using the mean glucose disposal rate normalized to body weight (M_BW_, (mg glucose/min*kg)), as calculated during the clamp. To test for statistical significance of global and pairwise differences in IR_,_ analyses of variances and the Student's t-test was used. To test for significant changes in IR within study arms, the paired t-test was used.

**Results:**

The enrolled volunteers were young, non-obese, healthy males; no significant differences were detected concerning baseline characteristics (Table 1). Mean IR did not differ between the groups before treatment (I vs. II vs. III: 11.2±3.2 (SD, standard deviation); n=10 vs. 12.5±3.3; n=9 vs. 11.6±2.5; n=9). The medication was well tolerated; 2 patients were excluded from analysis due to medical (hypothyroidism) and technical (insulin pump error) reasons. TDF/FTC+LPV/r significantly affected IR after 14 day of treatment as compared to baseline (9.2±1.8 vs. 12.5±3.3; p=0.037), but neither TDF/FTC/EVG/cobi (11.3±2.5 vs. 11.2±3.2; p=n.s.) nor TDF/FTC+DRV/r (11.3±2.4 vs. 11.6±2.5; p=n.s.) did.

**Conclusions:**

Our study shows for the first time that neither treatment with the fixed dose combination TDF/FTC/EVG/cobi nor with TDF/FDC+DRV/r affects IR as compared to the established regimen TDF/FTC+LPV/r.

**Abstract TUPDB0105–Table 1:** Baseline characteristics

Group/parameter (Mean±SD)	I: TDF/FTC/EVG/cobi	II: TDF/FTC + LPV/r	III: TDF/FTC + DRV/r
Age (years)	26.3 (±4.8)	27.3 (±4.8)	27.2 (±2.3)
Weight (kg)	75.3 (±4.8)	70.2 (±8.3)	72.3 (±7.6)
Body height (cm)	183.5 (±4.2)	178.9 (±5.7)	180.0 (±5.5)
BMI (kg/m^2^)	22.4 (±1.1)	21.9 (±2.2)	22.3 (±1.5)
Fasting blood glucose (mg/dl)	82.0 (±5.1)	82.3 (±6.7)	83.3 (±6.0)

# Sub article: Molecular investigation for HIV-1 cross-group transmissions during the outbreak period (2011–2014) in Athens metropolitan area: introduction of subtype A from Eastern Europe

**DOI:** 10.7448/IAS.18.5.20449

**Published:** 2015-07-22

**Authors:** Dimitrios Paraskevis, Georgios Nikolopoulos, Vana Sypsa, Mina Psichogiou, Meni Malliori, Samuel R Friedman, Angelos Hatzakis

**Affiliations:** 1 Department of Hygiene Epidemiology and Medical Statistics, Medical School, University of Athens, Athens, Greece; 2 Hellenic Center for Diseases Control and Prevention, Athens, Greece; 3 Department of Propedeutic Medicine, Laikon General Hospital, Athens, Greece; 4 Medical School, University of Athens, Athens, Greece; 5 National Development and Research Institutes, (NDRI), New York, United States

## Abstract

**Introduction:**

New diagnoses of HIV-1 infections among people who inject drugs (PWID) increased in Athens metropolitan area, Greece during 2011. Our aim was to identify potential cross-group transmissions between PWID and other risk groups using molecular methods.

**Methods:**

HIV-1 subtypes were determined for 711 HIV-1(+) PWID sampled during 2011–2014. Cross-group transmissions among the PWID were those that originated from other groups as estimated by phylogenetic trees. Specifically cross-group transmissions corresponded to viral lineages from PWID that didn't fall into the outbreak transmission networks or the PWID recombinants. Further phylogenetic analyses were conducted for the sequences from cross-group transmissions.

**Results:**

Among the 711 HIV-1(+) PWID, 630 (88.6%) sequences fell within four IDU transmission networks belonging to CRF14_BG (n=356, 50.1%), CRF35_AD (n=123, 17.3%), subtype B (n=106, 14.9%) and A (n=45, 6.3%); 48 (6.8%) were recombinants consisting of partial regions originating from the PWID-specific clades. On the other hand, sequences from 33 (4.6%) PWID didn't belong either to the PWID transmission networks or the recombinants, suggesting that they are evidence of potential cross-group transmissions. Phylogenetic analyses (n=28) for subtypes A and B detected most frequently among the cross-group transmissions suggested that most of these infections originated from non-PWID transmission networks in Greece and the former Soviet Union countries (A_FSU_). Specifically we found that nine (75.0%) of the subtype B infections originated from Greece, whereas eight (50.0%) and seven (43.8%) of subtype A strains were of A_FSU_ and Greek origin, respectively (Figure 6). The gender distribution didn't differ significantly between those infected within PWID networks (F: n=99; M: n=579) or the cross-group transmissions (F: n=7; M: n=26).

**Conclusions:**

During the four year period of the HIV-1 outbreak among the PWID in Athens metropolitan area, we estimated that 33 (4.6%) of the infections in this group are due to cross-group infections. Notably, half of these cross-group infections due to subtype A originate from the large IDU epidemic in Eastern Europe (A_FSU_). For subtype B, however, the majority of cross-group infections originated from Greece.

**Abstract TUPDC0101–Figure 1:** Phylogenetic tree of subtype A sequences from PWIDs with evidence for cross-group transmissions plus sequences from the Greek epidemic sampled during 1999–2013 and a randomly selected global sample.

# Sub article: Clusters of HIV transmission among high-risk populations in Pakistan

**DOI:** 10.7448/IAS.18.5.20450

**Published:** 2015-07-22

**Authors:** Laura H Thompson, Joel O Wertheim, Tahira Reza, John L Wylie, Faran Emmanuel, James Brooks, James F Blanchard, Paul Sandstrom

**Affiliations:** 1 Community Health Sciences, University of Manitoba, Winnipeg, Canada; 2 Department of Medicine, University of California at San Diego, San Diego, United States; 3 Centre for Global Public Health – Pakistan, Islamabad, Pakistan; 4 Department of Medical Microbiology, University of Manitoba, Winnipeg, Canada; 5 National HIV and Retrovirology Laboratory, JC Wilt Infectious Disease Research Centre, Public Health Agency of Canada, Winnipeg, Canada; 6 Centre for Global Public Health, University of Manitoba, Winnipeg, Canada

## Abstract

**Introduction:**

In Pakistan, people who inject drugs (PWID) have a high HIV prevalence (~27%), and the prevalence among sex workers (SW) has recently increased. There is considerable geographic heterogeneity of HIV prevalence, which may reflect multiple subepidemics with unique trajectories, characterized by specific risk contexts, behaviours and sexual or syringe-sharing networks. This study uses genetic clustering to identify and characterize these HIV subepidemics of ongoing transmission.

**Methods:**

Mapping and integrated behavioural and biological surveillance took place among 16,756 PWID and male (MSW), *hijra* (HSW) and female (FSW) SW across Pakistan in 2011. Of the 1637 persons who tested HIV positive (9.8%), we were able to analyze gp41 sequences from 1153. These sequences were aligned to a reference sequence: HXB2. We identified sequences that were highly similar (≤1% pairwise Tamura Nei 93 genetic distance) and deemed these persons potential transmission partners. Transmission clusters were constructed by connecting persons who share potential transmission partners. Clusters were characterized in terms of high risk population group membership and city. Logistic regression was used for tests of statistical significance.

**Results:**

The prevalence of HIV was determined to be 27.3%, 5.2%, 1.6% and 0.6% among PWID, HSW, MSW and FSW, respectively. Of the 1153 sequences, 652 were clustered (56.5%) in 87 unique clusters ranging in size from 2 to 96 sequences. Average cluster size was 7.5 (s.d.=15), although clusters of two predominated. Compared with MSW, PWID were more likely to be clustered (odds ratio=1.6, p=0.01). Larger clusters were more likely to span multiple cities and include SW, with an average mixed PWID/SW cluster size of 23.6, compared with cluster sizes of five or two for clusters composed entirely of PWID or SW, respectively. Most PWID who were in clusters were in large clusters of nine or more individuals, whereas HSW and MSW tended to be in clusters of diverse sizes.

**Conclusions:**

A comprehensive understanding of HIV transmission in Pakistan will be critical to design strategically targeted HIV prevention programs. Clusters may be indicators of ongoing transmission, and thus an effective strategy for prevention programs could be to target the cities and population groups with high clustering.

# Sub article: Transmission networks of HIV-1 among men who have sex with men in East and Southeast Asia

**DOI:** 10.7448/IAS.18.5.20451

**Published:** 2015-07-22

**Authors:** Kok Keng Tee, Rami Kantor, Somnuek Sungkanuparph, Yutaka Takebe, Patrick Li, Rossana Ditangco, Praphan Phanuphak, Thira Sirisanthana, Benedict Sim, Winai Ratanasuwan, Pacharee Kantipong, Mahiran Mustafa, Tuti Parwati Merati, Awachana Jiamsakul, Thida Singtoroj, Adeeba Kamarulzaman, TREAT Asia Studies to Evaluate Resistance - Monitoring (TASER-M)

**Affiliations:** 1 Department of Medicine, University of Malaya, Kuala Lumpur, Malaysia; 2 Department of Medicine, Alpert Medical School of Brown University, Providence, United States; 3 Department of Medicine, Faculty of Medicine, Ramathibodi Hospital, Mahidol University, Bangkok, Thailand; 4 AIDS Research Center, National Institute of Infectious Diseases, Tokyo, Japan; 5 Queen Elizabeth Hospital, Hong Kong, China; 6 Research Institute for Tropical Medicine, Manila, Philippines; 7 HIV-NAT/Thai Red Cross AIDS Research Centre, Bangkok, Thailand; 8 Research Institute for Health Sciences, Chiang Mai University, Chiang Mai, Thailand; 9 Hospital Sungai Buloh, Kuala Lumpur, Malaysia; 10 Faculty of Medicine Siriraj Hospital, Mahidol University, Bangkok, Thailand; 11 Chiangrai Prachanukroh Hospital, Chiang Rai, Thailand; 12 Hospital Raja Perempuan Zainab II, Kota Bharu, Malaysia; 13 Faculty of Medicine, Udayana University and Sanglah Hospital, Bali, Indonesia; 14 The Kirby Institute, UNSW Australia, Sydney, Australia; 15 TREAT Asia, amfAR, Bangkok, Thailand

## Abstract

**Introduction:**

The HIV epidemic among men who have sex with men (MSM) is expanding at an alarming rate in Asia. Understanding the dynamics of HIV-1 transmission among MSM through viral sequence analyses may provide essential information on the origin of viral lineages and the characteristics of disease spread.

**Methods:**

We determined transmission networks of HIV-1 among MSM across countries in East and Southeast Asia. A total of 1856 HIV-1 polymerase gene sequences were obtained from TREAT Asia Studies to Evaluate Resistance-Monitoring (TASER-M) sites in Hong Kong, Thailand, Malaysia and the Philippines between 2006 and 2011. Time-stamped sequence datasets of HIV-1 subtype B (n=144) and CRF01_AE (n=186) from antiretroviral-naive MSM were identified and subjected to spatiotemporal analysis using Bayesian phylodynamic methods. A transmission network was defined as a phylogenetic cluster (≥2 isolates) supported by >90% bootstrap values and Bayesian posterior probability value of 1 at the tree node.

**Results:**

Phylogenetic reconstructions showed that 68% of HIV-1 subtype B and 46% of CRF01_AE sequences were grouped in 50 transmission networks of various sizes (mean size=5.6, range=2–32 sequences), with subtype B sequences having a higher tendency to form a network (p<0.0001). With additional representative sequences from China, Mongolia and Myanmar from the Los Alamos National Laboratory HIV Sequence Database, 34 networks involving 154 subtype B-infected individuals and 16 networks involving 125 CRF01_AE-infected individuals were observed. Location mapping showed that the MSM networks in East and Southeast Asia were mostly localized (78%) in their respective countries, with 22% spanned beyond a single country. Genealogy-based analysis to estimate the divergence time for each transmission network indicated the continued emergence of new networks over the past three decades. The uninterrupted growth of sub-epidemics of various cluster sizes suggests the role of transmission networks as a continuous driving force of the epidemic among MSM in Asia.

**Conclusions:**

Despite expanded access to antiretroviral therapy in Asia, our analysis showed continued regional emergence of recent HIV-1 subtype B and CRF01_AE networks among MSM. Strategies such as early diagnosis and treatment as prevention to reduce transmission risks among sero-discordant partners need to be expanded across the region.

# Sub article: Estimating the size of men who have sex with men (MSM) using modified capture-recapture method based on network sampling in the capital city of Georgia in 2014

**DOI:** 10.7448/IAS.18.5.20452

**Published:** 2015-07-22

**Authors:** Lela Sulaberidze, Ali Mirzazadeh, Ivdity Chikovani, Natia Shengelia, Nino Tsereteli, George Gotsadze

**Affiliations:** 1 Curatio International Foundation, Tbilisi, Georgia; 2 Global Health Sciences, University of California San Francisco, San Francisco, United States; 3 Regional Knowledge Hub and WHO Collaborating Centre for HIV Surveillance, Kerman, Islamic Republic of Iran; 4 Center for Information and Counseling on Reproductive Health – Tanadgoma, Tbilisi, Georgia

## Abstract

**Introduction:**

Estimates of the number of people at high risk for HIV infection are crucial for prevention, treatment and care planning. Taking into consideration that Georgia is the country, where HIV prevalence is concentrated among men who have sex with men (MSM) and information on the size of this key population was lacking, we conducted the study using seven different population size estimation methods in Tbilisi, Georgia. We want to focus on a new method proposed by Dombrowski among methamphetamine users in 2012. This represents capture-recapture using network sampling technique. Among MSM, we first time applied this method with few modifications.

**Methods:**

Modified capture-recapture requires singe sample, which for our study was 210 MSM 18 years and older recruited through Respondent Driven Sampling. The study participants were asked about their personal characteristics (approximate height, weight, hair colour and ethnicity) and so called “telefunken codes” derived from the last four digits of their own mobile number. In difference to the original method that used six personal identifiers, we dropped eye colour (based on piloting results) and gender. This represented the capture. Afterwards, the study participants were asked to provide the similar characteristics appealing to their five MSM contacts randomly selected from mobile phone directories. This represented the recapture. Some respondents (2.38%) did not have mobile phones with them and some did not have five MSM contacts in their mobile phone directory. To get to the final estimates Lincoln-Peterson method was used.

**Results:**

Using the four-identifier categorical variables and the “telefunken code,” we identified 36 matches between the two captures (205 captured and 770 recaptured). This led to the population size of 1.2% (95% CI, 0.9%–1.6%) of the adult male population. The results were comparable to those from other methods used in our study (see Table 1).

**Conclusions:**

Despite the study limitations – difficulty to get the “telefunken codes” for the recapture phase – modified capture-recapture method provides reasonable population size estimates for MSM when compared to the median estimates and their boundaries of other more established methods. Estimating size of MSM through modified capture-recapture method appeared to be feasible, simple, cost-saving and effective method that is valuable for future application.

**Abstract TUPDC0104–Table 1:** Different MSM population size estimates from various methods implemented in Tbilisi, 2014

PSE method	Point estimate (18–59y)	Lower bound (18–59y)	Upper bound (18–59y)
Modified capture-Recapture	4385	3115	5654
MSM size – median of all seven estimates*	5100	3243	9088
MSM prevalence in adult population (%)	1.42	0.90	2.53

* Estimates derived from the following methods: Network Scale-Up, Web- and mob-App Multipliers, Service Multiplier, Unique Object Multipliers, RDS-based Handcock, Wisdom of Crowd, Modified Capture-Recapture.

# Sub article: Network-level factors associated with IPV perpetration among young urban Tanzanian men

**DOI:** 10.7448/IAS.18.5.20453

**Published:** 2015-07-22

**Authors:** Marta Mulawa, HLM Reyes, V Foshee, CT Halpern, L Kajula, S Maman

**Affiliations:** 1 Health Behavior, University of North Carolina at Chapel Hill, Durham, United States; 2 Maternal and Child Health, University of North Carolina at Chapel Hill, Durham, United States; 3 Department of Psychiatry and Mental Health, Muhimbili University of Health and Allied Sciences, Dar es Salaam, United Republic of Tanzania

## Abstract

**Introduction:**

Research suggests that characteristics of an individual's social network may influence intimate partner violence (IPV) perpetration among men in sub-Saharan Africa. For example, studies indicate that network-level measures of gender norms or IPV acceptance may be associated with IPV perpetration. However, to date, no studies have identified network-level factors associated with IPV among East African youth. We used data from our on-going HIV prevention trial in Dar es Salaam, Tanzania with 1268 men, ages 15–59 years (mean=26), nested within 60 networks of randomly selected social clubs called “camps.” The purpose of this study was to assess the degree to which variance in men's IPV perpetration was attributed to camp membership and to determine the effect of camp-level norms (gender norms and IPV attitudes) on IPV perpetration.

**Methods:**

We used 2-level hierarchical linear models to model the relationship between individual and camp-level characteristics and past-year physical IPV perpetration, assessed using an adapted version of the World Health Organization violence against women instrument. Camp-level gender norms were computed by averaging responses among all camp members to an adapted version of the Gender Equitable Men Scale. All individual-level variables were group-mean centred to facilitate decomposition of between and within-camp effects. We estimated an unconditional random effects model to determine the proportion of IPV variance attributable to camp membership. Subsequent models sequentially introduced individual-level demographic/control variables, camp-level norms and individual-level norms.

**Results:**

A significant proportion of variance in IPV perpetration (3.1%) was due to between-camp differences (τ_00_=0.0054, p=0.01). Increasing levels of camp equitable gender norms were significantly associated with decreasing IPV perpetration (γ=−0.167, p=0.04), and this association remained after controlling for individual-level gender norms. Camp-level norms regarding IPV acceptance were not associated with IPV perpetration.

**Conclusions:**

Studies have found a strong association between IPV and HIV. We found that membership in social groups with equitable gender norms reduced men's risk of perpetrating IPV, even after adjusting for their own views about gender norms and the acceptability of violence. This finding highlights the importance of multilevel HIV and IPV interventions that simultaneously address individual risk factors while making gender norms more equitable within social networks.

# Sub article: Gender differences in HIV testing behaviours by community-level and individual-level stigma in rural South Africa

**DOI:** 10.7448/IAS.18.5.20454

**Published:** 2015-07-22

**Authors:** Sarah Treves-Kagan, Alison El Ayadi, Audrey Pettifor, Catherine MacPhail, Rhian Twine, Suzanne Maman, Kathleen Kahn, Sheri A Lippman

**Affiliations:** 1 Center for AIDS Prevention Studies, University of California, San Francisco, United States; 2 Department of Obstetrics, Gynecology and Reproductive Sciences, Bixby Center for Global Reproductive Health, University of California, San Francisco, United States; 3 Gillings School of Global Public Health, University of North Carolina at Chapel Hill, Chapel Hill, United States; 4 Faculty of Health Sciences, School of Clinical Medicine, Wits Reproductive Health and HIV Institute (WRHI), University of the Witwatersrand, Johannesburg, South Africa; 5 Faculty of Health Sciences, School of Public Health, Medical Research Council/Wits Rural Public Health and Health Transitions Research Unit (Agincourt), University of the Witwatersrand, Johannesburg, South Africa; 6 Collaborative Research Network, University of New England, Armidale, Australia

## Abstract

**Introduction:**

Despite national testing campaigns and increased access to HIV treatment, stigma remains a significant barrier to testing in South Africa. A nuanced understanding of stigma and testing is instrumental in refining intervention programming. Stigma can be examined at either the individual or community level and may operate differentially by gender. Further, estimating HIV testing uptake achievable through stigma reduction interventions is critical for understanding potential impact.

**Methods:**

We examined the relationship between anticipated HIV stigma at individual and community levels on recent HIV testing, stratified by gender, using data from a population-based sample of 1126 adults aged 18–35 residing in 22 villages in Mpumalanga, South Africa. Anticipated HIV stigma, or expectations of discrimination should one become HIV positive, was measured using a 9-item scale and dichotomized as *any* versus *no* stigma. Community-level stigma was defined as the proportion of individuals within each village reporting any anticipated stigma. We assessed associations of community and individual stigma and HIV testing for men and women. We then used multi-level regression models to estimate the potential effect of changing community-level stigma to improve testing uptake using the g-computation algorithm. Analyses were weighted to account for the survey design.

**Results:**

Men tested less frequently (OR 0.22, 95% CI 0.14–0.33) and reported more individual anticipated stigma (OR 5.1, 95% CI 2.6–10.1) than women. Men reporting no individual-level stigma (vs. some) were 48% more likely to have tested (p=0.08). For women, testing behaviour was not associated with individual anticipated stigma but for each percentage point reduction in community-level stigma the likelihood of testing increased by 3% (p=0.03). We modelled gains in HIV testing at different levels of community stigma (Figure 1). For example, results indicate a potential 15% intervention gain in HIV testing among women if community-level stigma decreased by 5%. Changing community-level stigma did not result in significant gains for men.

**Conclusions:**

Our data indicates that HIV-related stigma influences HIV testing for men and women through different pathways. Stigma reduction programs may need to consider gender differences and tailor activities to the target population. Longitudinal research is needed to confirm projections and direction of effect.

**Abstract TUPDD0101–Figure 1:** Testing under various stigma scenarios.

# Sub article: Men “missing” from population-based HIV testing: insights from qualitative research

**DOI:** 10.7448/IAS.18.5.20455

**Published:** 2015-07-22

**Authors:** Carol S Camlin, Emmanuel Ssemmondo, Gabriel Chamie, Dalsone Kwarisiima, Norton Sang, Elizabeth A Bukusi, Craig R Cohen, Moses R Kamya, Diane Havlir

**Affiliations:** 1 Obstetrics, Gynecology & Reproductive Sciences, University of California – San Francisco (UCSF), San Francisco, United States; 2 Infectious Disease Research Collaboration (IDRC), Kampala, Uganda; 3 Medicine, University of California – San Francisco (UCSF), San Francisco, United States; 4 Kenya Medical Research Institute (KEMRI), Kisumu, Kenya

## Abstract

**Introduction:**

Men's uptake of HIV testing will be critical to the success of test and treat strategies in generalized epidemics. We used qualitative research methods to identify cultural factors and community level processes that influence HIV testing uptake in the context of an ongoing test and treat trial of 334,479 persons in East Africa (SEARCH, NCT# 01864603).

**Methods:**

In-depth interviews, participant observation and focus group discussions were used to evaluate contextual factors in communities that influenced uptake of baseline HIV testing. The study used a hybrid model of mobile HIV testing including community health campaigns (CHC) followed by home-based testing (HBT) for non-CHC attendees. Data were collected in eight rural communities in Uganda and Kenya and interpreted using Atlas.ti software. Analytical codes were defined and applied by an 8-person research team on the basis of theory and the empirical data, and iteratively refined during the analysis process.

**Results:**

Structural barriers to male participation in community health campaigns led to reduced participation in CHCs and HBT: informal sector labour opportunities for men often require extended absences from rural households. Participants reported for example that during planting season, men needed to guard fields from monkeys from dawn until nightfall; in lakeshore communities, fishermen travel long distances and off-load fish at multiple beaches, using multiple residences and temporary lodgings. Community leaders were critical in outreach to promote CHC attendance, but power differentials between elder and younger men may have contributed to heterogeneous mobilization. Cultural factors including male gender norms counter to health-seeking behaviours, and valorizing risk-taking, also served as barriers to HIV testing. Men often tested “by proxy,” inferring their HIV status from the test results of wives. Yet debates about HIV risks were vigorous, with many men questioning traditional masculine gender norms; moreover, the promise of antiretroviral therapy (ART) to prolong health appeared to motivate many men to participate in testing.

**Conclusions:**

Mobile testing reduces but does not eliminate barriers to men's participation; however, the promise of ART may be enabling changes in male gender norms related to testing. Findings may be useful for developing novel strategies to improve male engagement in test and treat efforts.

**Abstract TUPDD0102–Table 1:** Illustrative Quotes

***Entrenched gender norms***
• “Men are generally lazy … ‘I am already infected and still want to show my male ego without considering my family’ … many men as well are not ready to take up HIV test and would push their partners to go first and rely on their results.” – Male youth Focus Group Discussion (FGD) participant, Sena
• “As men we have a lot of fear … Men also like giving excuses, that they are ever busy in the name of searching for the family, even if they have gotten this food that they are ever looking for [laughter].” – Male adult FGD participant, Sena
• “Many men believe that medical issues are women's affairs.” – Male adult FGD participant, Ongo
• “Men are people with hardened hearts. They will hardly rush for any programme. They can release their wives and children first to go, and for him, he assesses before going.” – Female adult FGD participant, Kameke
***Signs of changing gender norms***
• Interviewer: “You have mentioned that most people do not test as couples; please tell me more about this?”
• “A good percentage of men are not faithful. It is men who would even end up enrolling for HIV care at a very far facility. Men should change and be free to test as couples so as to build trust. They should stop frustrating their women as well.” [Female adult participant]
• “Gender based violence is real and rampant in this community. This is so because there is no family dialogue to discuss family issues. I do dialogue in my house but when I introduced the HIV topics, many started avoiding the dialogue.” [Male adult participant, FGD Tom Mboya]

# Sub article: Examining the relationship between paediatric PMTCT outcomes and knowledge of partner status

**DOI:** 10.7448/IAS.18.5.20456

**Published:** 2015-07-22

**Authors:** Anne Schley, Ewa Skowronska, Emeka Okonji, Esca Scheepers

**Affiliations:** Department of Programs and Technical Support, mothers2mothers, Cape Town, South Africa

## Abstract

**Introduction:**

The mothers2mothers’ (m2m) Mentor Mother programme empowers pregnant women and new mothers to make informed decisions about their maternal and reproductive health as well as their infants’ health, through provision of peer education and psychosocial support. The m2m's 2013 annual evaluation showed that discordancy was negatively associated with the uptake of paediatric prevention of mother-to-child transmission (PMTCT) services. HIV-positive mothers who knew their male partners were HIV-negative were less likely to bring their infants for PCR testing at 6–8 weeks (OR=0.60, p=0.005), or for a follow-up test at 18 months (OR=0.75, p=0.017), compared to mothers who knew their partners were HIV-positive. The aim of this study is to further investigate the role that knowledge of one's partner's HIV status plays in the uptake of paediatric PMTCT services.

**Methods:**

Secondary analysis of m2m's 2013 internal programme evaluation data was conducted. Data comprised of a representative random sample of 5592 HIV-positive clients’ longitudinal records (routinely maintained by Mentor Mothers), enrolled from March through May 2012 in six African countries. The relationship between knowledge of partner status and uptake of paediatric PMTCT services was investigated through bivariate analysis (chi-square) and binary logistic regression analysis using STATA 12.

**Results:**

Knowledge of partner HIV status was significantly associated with uptake of paediatric PMTCT services. Mothers who knew their partner's HIV status were more likely to take up paediatric PMTCT services compared to those who did not know their partner's status. The likelihood of improved uptake of PMTCT services was the highest among mothers who knew they were in a concordant relationship. There was no significant relationship between knowledge of partner status and uptake of infant ART.

**Conclusions:**

Additional primary research on the effects of concordancy and discordancy on PMTCT outcomes is recommended. Our secondary analysis suggests that uptake of paediatric PMTCT services is more likely to occur amongst clients who know that they are in a concordant relationship. This evidence supports m2m's inclusion of a tailored serodiscordant couples education and support intervention to facilitate mutual disclosure of HIV status in partners, especially in the context of Option B+, thus improving outcomes in the postnatal care cascade.

**Abstract TUPDD0103–Table 1:** Paediatric PMTCT and knowledge of partner status

	Unknown partner HIV status	Partner known HIV positive (known concordant relationship) OR (p-value)	Partner known HIV negative (known discordant relationship) OR (p-value)
Infant PCR test	1	1.96 (0.000)	1.20 (0.261)
Infant PCR test result	1	2.12 (0.000)	1.41 (0.022)
Infant 18 months test	1	1.89 (0.000)	1.41 (0.012)
Infant 18 months test result	1	1.91 (0.000)	1.44 (0.008)
Infant on ART	1	0.84 (0.505)	0.89 (0.751)

# Sub article: Who benefits from partner services in Mozambique? Results from a pilot programme in a public, urban clinic

**DOI:** 10.7448/IAS.18.5.20457

**Published:** 2015-07-22

**Authors:** Caryl Feldacker, Serene Myers, Freide Cesar, Zulmira Parades, Catarina Ferrao, Sinesia Citao Citao, Florindo Mudender, Matthew Golden

**Affiliations:** 1 Global Health, International Training and Education Center for Health, University of Washington, Seattle, United States; 2 International Training and Education Center for Health, Maputo, Mozambique; 3 Ariel Glaser Foundation, Maputo, Mozambique; 4 Provincial Department of Health, Maputo, Mozambique; 5 Division of Allergy and Infectious Diseases, University of Washington Harborview Medical Center, Seattle, United States

## Abstract

**Introduction:**

Notifying partners of persons newly diagnosed with HIV can help identify undiagnosed infections and link people to care. Assisted partner services (APS) offers persons with newly diagnosed HIV infection help notifying and getting sex partners tested. APS is not widely available in sub-Saharan Africa, including Mozambique. We explore who benefits from APS as compared to passive services through a pilot programme in an urban, public clinic in Maputo, Mozambique.

**Methods:**

Between June and September 2014, four community health workers (CHWs) offered APS to 223 index patients (IPs) with recently diagnosed HIV: 220 accepted and 206 (94%) were retained at eight weeks. CHWs used structured interviews to collect data at baseline, four and eight weeks. At baseline, CHWs counselled IPs to notify partners and encourage their HIV testing, but did not offer to notify partners directly. At four weeks, with consent, CHWs notified partners to encourage testing. We used logistic regression, adjusted for clustering, to define the odds that APS increased HIV testing uptake and identified new HIV infections, setting significance at p≤0.05.

**Results:**

Of 206 IPs, 79% were female, 73% were married and 31% named >1 sex partner. IPs named 283 partners, 278 had complete date: 59% are spouses. Of 192 people tested, 103 (53.6%) tested after APS at four weeks. Of 103 HIV positive diagnoses, 55 (53.4) were reported at eight, but not four, weeks, suggesting APS-assisted identification of new HIV infections. APS appeared to increase both partner HIV testing and identification of HIV-infected partners across a range of subgroups (Table 1). The magnitude of impact varied. In multivariate analysis, APS appeared more effective among persons in ongoing sexual relationships and less effective among persons with multiple sex partners, a group in whom partner testing and HIV identification remained relatively low.

**Conclusions:**

APS significantly improves HIV testing uptake and case-finding among current sex partners: those in monogamous pairs benefit most. These findings suggest that the model of APS piloted in Mozambique might be most profitably focused on persons in ongoing partnerships and highlights the need for better interventions for persons with multiple sex partners.

**Abstract TUPDD0104–Table 1:** Factors associated with uptake of assisted partner services for HIV testing and HIV diagnosis

N=278	Tested Prior to APS # (%)	Total Tested at 8 weeks # (%)	OR testing Post v Pre APS* (Univariate)	OR testing Post v Pre APS** (Multivariate)	HIV+ Prior to APS # (%)	Total HIV+ at 8 weeks # (%)	OR HIV+ Post v Pre APS* (Univariate)	OR HIV+ Post v Pre APS** (Multivariate)
Male partner (ref: female)	59 (28.8)	142 (69.3)	1.61 (0.91–2.84)		33 (15.8)	78 (38.1)	1.51 (0.76–2.99)	
Live together	66 (43.4)	124 (81.6)	1.07 (0.67–1.70)		36 (23.7)	71 (46.7)	1.67 (0.92–3.03)	
IP has >1 sex partner	37 (27.4)	73 (54.1)	0.43 (0.26–0.72)	0.52 (0.31–0.89)	19 (13.6)	33 (24.4)	0.28 (0.15–0.52)	0.39 (0.20–0.77)
Has continuing sexual relations	78 (35.3)	172 (77.8)	2.78 (1.44–5.33)	2.09 (1.04–4.17)	43 (19.3)	94 (42.5)	3.36 (1.27–8.93)	1.92 (0.70–5.53)
IP reason for HIV testing: symptoms	20 (23.8)	52 (61.9)	0.99 (0.58–1.70)		9 (10.6)	24 (28.6)	0.74 (0.39–1.39)	
IP reason for HIV testing: prenatal	44 (37.0)	96 (80.7)	1.61 (0.97–2.67)		21 (17.2)	54 (45.4)	2.34 (1.30–4.21)	1.66 (0.91–3.03)
Total	89	192			48	103		

Results from logistic regression models using the (cluster) option in STATA. 95% CI presented in parentheses.

* Results from univariate models.

** Results from multivariate models.

# Sub article: Male partner acceptance of home-based syphilis and HIV testing offered to couples during pregnancy

**DOI:** 10.7448/IAS.18.5.20458

**Published:** 2015-07-22

**Authors:** Jennifer Mark, John Kinuthia, Alfred Osoti, Molly Gone, Victor Asila, Saloni Parikh, Daisy Krakowiak, Bourke Betz, Barbra Richardson, Alison Roxby, Carey Farquhar

**Affiliations:** 1 Epidemiology, University of Washington, Seattle, United States; 2 Kenyatta National Hospital, Nairobi, Kenya; 3 Department of Obstetrics and Gynecology, University of Nairobi, Nairobi, Kenya; 4 School of Public Health and Community Medicine, University of Washington, Seattle, United States

## Abstract

**Introduction:**

Testing partners for HIV in the antenatal period is an effective way to bring HIV services to couples. Leveraging antenatal HIV testing with point of care (POC) diagnostics for other sexually transmitted infections (STI) may improve male partner treatment services among couples.

**Methods:**

We conducted a prospective study among male partners of pregnant women who received home-based couple HIV testing and education (HOPE) following a first antenatal visit in Kisumu, Kenya. From April to July 2014, rapid POC syphilis testing (SD Bioline Syphilis 3.0) was added to the package of services for men and those with positive results were referred to the clinic for treatment. We assessed men's acceptance of testing and intention to seek clinic-based treatment and calculated an odds ratio to examine correlation between uptake of syphilis and HIV testing.

**Results:**

Data were available for 73 (83%) couples receiving a HOPE visit. Men were on average 26 years of age (IQR: 22, 29). At study entry, most men reported having previously tested for HIV (93%, n=68), of whom 7% reported being of known HIV positive status (n=5) and 80% reported knowing their female partner's HIV status (n=59). Of 73 men, 67 accepted syphilis testing (92%) among whom 64 intended to attend clinic STI treatment if they received a positive syphilis result (95%). HIV prevalence among the men was14.7% and one man (<1%) was syphilis positive. In this group, 61 (83%) accepted both syphilis and HIV tests. Three men (4%) refused both tests and three men (4%) accepted HIV alone. Six men (8%) accepted syphilis alone, of whom two reported having been previously tested as HIV-positive. If a man accepted HIV testing, he was 10-fold as likely to accept syphilis testing, compared to a man who refused HIV testing (OR: 10.2; 95% CI: 1.05–89.3; p=0.02).

**Conclusions:**

In a high HIV and low syphilis setting, home-based education and POC syphilis testing of male partners during pregnancy is highly acceptable when coupled with HIV testing and may encourage men to seek clinic-based STI services. Integration with HIV testing appears feasible, and syphilis test uptake is highly correlated with HIV test uptake.

# Sub article: Antiretroviral treatment uptake and correlates of adherence among men who have sex with men and transgender women in Mumbai, India

**DOI:** 10.7448/IAS.18.5.20459

**Published:** 2015-07-22

**Authors:** Christopher Pina, Alpana Dange, Shruta Neytra, Harish Kambli, Santosh Karambe, Rosy Chhabra, Viraj Patel

**Affiliations:** 1 Albert Einstein College of Medicine, Bronx, United States; 2 Humsafar Trust, Mumbai, India; 3 Montefiore Medical Center, Bronx, United States

## Abstract

**Introduction:**

Understanding factors influencing ART adherence is needed to optimize treatment responses for HIV infected men who have sex with men (MSM) and Hijra/transgender women (TGW) in India. The objective of this formative study was to determine rates of ART uptake and adherence and explore potential factors associated with adherence in Indian MSM and TGW.

**Methods:**

We conducted a cross-sectional survey in Hindi among all HIV positive MSM and TGW on ART accessing support services at a LGBT community based organization in Mumbai between July and September 2014. Non-adherence was measured by self-report and defined as missing any doses (i.e. <100% adherent) in the past one month and three months. Potential correlates of adherence assessed were sociodemographics, medication side-effects, depression (CESD-10), self-efficacy (GSE), internalized homophobia/stigma and medication beliefs using chi-square or t-tests.

**Results:**

Of the 300 individuals registered in the organization's HIV support programme, 28.3% (85/300) were eligible for ART by current country standards (e.g. CD4=350 or having an OI) with 22% (65/300) currently on ART. Of those on ART, 83% (54/65) were MSM and 17% (11/65) TGW; 40% (25/65) were married to women, and most (97%) received free ART through government clinics. Overall, 32% (21/65) were non-adherent in the past one month and 45% (29/65) in past three months. Correlates (p<0.05) of non-adherence were similar for one month and three months and were associated with younger age, non-Kothi identity (MSM subgroup), alcohol use, having sex with women, feeling healthy and negative medication beliefs but was not directly associated with depression, internalized homophobia, or medication self-efficacy.

**Conclusions:**

In one of the first studies of adherence among MSM and TGW in India, ART treatment uptake and adherence were suboptimal. Modifiable factors associated with adherence may serve as targets for interventions to support adherence. Further work is however needed to verify self-report measures with biological outcomes and confirm findings in other samples of Indian MSM and TGW.

# Sub article: Evolution of neutralizing antibodies in HIV-1 subtype C infection

**DOI:** 10.7448/IAS.18.5.20460

**Published:** 2015-07-22

**Authors:** Bongiwe Ndlovu, Tandile Hermanus, Nancy Tumba, Penny Moore, Manjeeta Jaggernath, Bruce D Walker, Lynn Morris, Thumbi Ndung'u

**Affiliations:** 1 HIV Pathogenesis Programme, University of KwaZulu-Natal, Durban, South Africa; 2 AIDS Virus Research Unit, National Institute for Communicable Diseases, Johannesburg, South Africa; 3 Ragon Institute of MGH, University of Cambridge, MIT & Harvard, Boston, United States

## Abstract

**Introduction:**

The development of a preventative HIV-1 vaccine will most likely require induction of broadly neutralizing antibodies (BCN). Neutralizing antibodies develop in almost all HIV-1 infected individuals, however they develop months following HIV-1 infection and they are strain-specific. The development of BCN antibodies occurs only in 20–30% of HIV-1 infected individuals. However, the mechanism that leads to the development of BCN is unknown and not all epitopes have been identified. The aim of the study was to evaluate pathways and mechanisms that lead to the development of broadly neutralizing antibodies.

**Methods:**

Twenty individuals with acute HIV-1 infection were identified and followed longitudinally for three years in Durban, KwaZulu-Natal. A panel of 18 viruses (6 subtype A, 6B and 6C) was used to screen the patients for neutralizing antibodies at 2–3 years post-infection using the TZM-bl neutralization assay. The patients that developed broadly neutralizing antibodies were followed up longitudinally at 8, 10, 14, 16, 18, 71, 88, 100, 124, 150, 200 weeks to determine the timing of emergence of the BCNs. Specificity of BCNs was determined using single point mutagenesis at 3 years post-infection.

**Results:**

Three out of 20 individuals (AS3-268, AS2-1037, AS2-358) developed broadly neutralizing antibodies. AS3-268 developed potent BCN activity peaking at three years post-infection and it targets N276A glycan on the CD4 binding site of gp120. AS2-1037 developed potent broadly neutralizing activity peaking at two years post-infection and it targets N332A glycan on the V3 loop of gp120. AS2-358 developed BCNs peaking at two years post-infection and it did not map to any known specific epitope.

**Conclusions:**

Broadly neutralizing antibodies could be detected at approximately one year post-infection and they targeted different epitopes on the viral envelope. Work is currently in progress to assess the maturation of breadth and to assess antibody-virus co-evolution.

# Sub article: HLA-B*58:02-specific benefit of MRKAd5 Gag/Pol/Nef vaccine in an African population

**DOI:** 10.7448/IAS.18.5.20461

**Published:** 2015-07-22

**Authors:** Ellen Michelle Leitman, Jacob Hurst, Masahiko Mori, Philippa C Matthews, Nicole Frahm, James Kublin, Glenda E Gray, Philip JR Goulder

**Affiliations:** 1 Department of Paediatrics, University of Oxford, Oxford, United Kingdom; 2 Department of Medicine, University of Oxford, Nuffield Oxford, United Kingdom; 3 Vaccine and Infectious Disease Division, Fred Hutchinson Cancer Research Center, HIV Vaccine Trials Network, Seattle, United States; 4 South African Medical Research Council, Cape Town, South Africa; 5 HIV Pathogenesis Programme, Doris Duke Medical Research Institute, University of KwaZulu-Natal, Durban, South Africa

## Abstract

**Introduction:**

The MRKAd5 Gag/Pol/Nef vaccine increased the risk of HIV acquisition. However, the Step study suggested an HLA-specific benefit in viral setpoint to vaccinees who subsequently became infected. The Phambili trial, using the same MRKAd5 vaccine, presented an opportunity to investigate the existence of an HLA-specific effect in a genetically distinct South African population. Gag-specific CD8 T-cell responses restricted by protective South African HLA alleles such as HLA-B*57 are associated with successful control of infection, while disease-susceptible alleles such as HLA-B*58:02 present non-Gag epitopes and are associated with rapid progression. We hypothesized that the MRKAd5 Gag/Pol/Nef vaccine might redirect responses towards Gag in HLA-B*58:02+ Phambili subjects who would not target it naturally.

**Methods:**

Viral loads (VL), CD4 T-cell counts, HLA types and ELISpot anti-HIV CD8 T-cell responses were analyzed in subjects blinded to vaccine/placebo assignment. All data analyzed were from ART-naïve subjects.

**Results:**

HLA-B*58:02 was the most prevalent allele (population frequency 23%). HLA-B*58:02+ vaccinees (n=7) had lower viral setpoints than placebo-recipients (n=7) (25,670 vs. 215,500, p=0.03), a 0.8log lower VL calculated using all longitudinal pre-ART data via a mixed effects model (p<0.001, Figure 1a), reached CD4<350 cells/µl slower (p=0.002, Figure 1b) and showed an increase in Gag breadth in ELISpot assays (p=0.04) compared to HLA-B*58:02+ placebo-recipients.

**Conclusions:**

In addition to the known increased risk of HIV acquisition resulting from the MRKAd5 Gag/Pol/Nef vaccine, these current data suggest a therapeutic effect of the same vaccine in subjects expressing HLA-B*58:02, an African HLA allele strongly associated with rapid progression in natural HIV infection. HLA-B*58:02+ vaccinees showed a lower viral setpoint and slower time to CD4<350 cells/µl, associated with increased Gag-specific ELISpot responses. Caveats to the study include limitation of ELISpot assays to only 60 of 100 study subjects, selected based on cell availability; and of HLA typing to 79 subjects, based on material availability. These factors potentially introduced unintended selection bias effects. Nonetheless, these data on ART-naïve subjects are consistent with Step studies, indicating a beneficial therapeutic MRKAd5 HLA-specific effect that in South Africa includes the most prevalent HLA-B allele, HLA-B*58:02.

**Abstract WEPDA0102–Figure 1:** (a) Graphical presentations of patient longitudinal pre-ART data and regression lines obtained from a variable intercept linear mixed effects model showing longitudinal VL differences in infected B*58:02+vaccinees (blue, n=7) and placebo-recipients (red, n=7); ANOVA. (b) Kaplan-Meier curves showing time to CD4<350 cells/µl in infected HLA-B*58:02+vaccinees (blue, n=7) and placebo-recipients (red, n=7); log rank test.

# Sub article: HIV-1 subtype C is significantly more infectious than other subtypes

**DOI:** 10.7448/IAS.18.5.20462

**Published:** 2015-07-22

**Authors:** Todd Demarco, Wes Rountree, Bhavna Hora, Yue Chen, Sarah Keinonen, Laura Racz, Lily Daniell, Raul Louzao, Ana Sanchez, Michael Busch, Thomas Denny, Feng Gao

**Affiliations:** 1 Departments of Medicine, Duke Human Vaccine Institute, Duke University Medical Center, Durham, United States; 2 Blood Systems Research Institute, San Francisco, United States

## Abstract

**Introduction:**

HIV-1 subtype C accounts for about 50% of the global HIV-1 infections. It is the predominant subtype in India, Ethiopia and countries in southern Africa. However, virological attributes to this unique epidemiological pattern have not yet been fully defined.

**Methods:**

A total of 207 HIV-1 positive plasma or established strains were cultured and expanded to higher titre stocks by culturing in PBMCs from HIV-1 negative donors. Near full-length genome (NFLG) sequences were obtained by amplifying two overlapping half genomes. The newly obtained sequences were aligned to the HIV-1 whole genome reference sequences for subtyping. Viral genome copy numbers, tissue culture infection doses (TCID) and p24 concentrations were determined for virus stocks and compared via linear regression among major subtypes. Mann-Whitney U tests were used for the infectivity comparisons at the alpha 0.05 level.

**Results:**

Analysis of NFLG sequences showed that these viruses belonged to subtype A1 (16), subtype B (48), subtype C (53), subtype D (10), CRF01_AE (12), other subtypes and CRFs (F1, F2 G, CRF02, and CRF022; each with ≤8 sequences) and URFs (45). Only subtypes with ≥10 NFLG sequences were subjected to further analysis. No biologically relevant differences (a 0.5 log_10_ difference) among all compared subtypes were observed for three measurements: viral genome copy numbers, TCID, and p24 concentrations. The only exception was that the TCID of subtype C was 0.51 log higher than that of CRF01 (p=0.04). The infectivity per viral genome (TCID/RNA copy) was the highest for subtype C (0.00452 TCID/RNA copy) and was significantly higher than those of all four compared subtypes (A1, B, D and CRF01_AE; p=0.0286, p=0.0004, p<0.001 and p=0.0205, respectively). The p24/RNA copy ratios of subtypes C and B (0.13 and 0.12 pg/RNA copy, respectively) were the highest and were significantly higher than those of subtypes A1 and D (p<0.05), but similar to that of CRF01_AE.

**Conclusions:**

The high infectivity of HIV-1 subtype C may give it more replication advantages and allow it to disseminate faster in HIV-1 infected populations in some geographic areas compared to other subtypes. High infectivity may play a critical role in the global epidemic of subtype C.

# Sub article: Functional differences in the viral accessory protein Nef between major HIV-1 subtypes

**DOI:** 10.7448/IAS.18.5.20463

**Published:** 2015-07-22

**Authors:** Aaron Johnson, Rajesh Jacob, Mansour Haeryfar, Jimmy Dikeakos

**Affiliations:** Department of Microbiology and Immunology, University of Western Ontario, London, Canada

## Abstract

**Introduction:**

The HIV-1 accessory protein Nef is essential for HIV-1 pathogenesis and progression to AIDS. By hijacking the cellular trafficking machinery Nef is able to alter T cell activation, increase viral replication and permit viral immune evasion via downregulation of the cell-surface receptors CD28, CD4 and MHC I, respectively. However, only recently have these functions been studied outside of laboratory-adapted strains of HIV-1. This proposal aims to investigate how the high degree of HIV-1 genetic diversity impacts Nef function.

**Methods:**

An HIV-1 based lentiviral expression system was used to express Nef proteins from 10 group M subtypes (A1, A2, B, C, F1, F2, G, H, J and K) in the context of an HIV-1 infection. T cell lines were infected with pseudoviruses encoding Nef proteins and analyzed for surface levels of CD28 and MHC-I using fluorescent antibody staining and flow cytometry. Alternatively, CD4 cell surface levels were measured by transfecting CD4^+^ HeLa cells with expression plasmids encoding Nef-GFP fusion proteins followed by fluorescent antibody staining and flow cytometry. Nef expression was determined by a combination of western blot analysis and flow cytometry to measure fluorophore fused Nef proteins.

**Results:**

Our results demonstrate that MHC I, CD28 and CD4 are differentially downregulated between HIV-1 subtypes. Notably, subtype C Nef, the most common subtype globally, was significantly less efficient at downregulating MHC I and CD28 when compared to the laboratory strain NL4.3. Subtype G Nef, found predominantly in Central and West Africa, was significantly less efficient at downregulating all three cell surface receptors. Differences in downregulation efficiency for all three receptors were attributed to variations in Nef protein expression.

**Conclusions:**

This study represents a comprehensive analysis of Nef function among 10 HIV-1 subtypes and adds to the growing evidence that HIV-1 genetic diversity impacts viral protein function. Due to the pathogenic role Nef plays in an HIV-1 infection, these results may help explain recent studies that show differences in disease progression in individuals infected with different HIV-1 subtypes. Finally, these findings support further study of all major HIV-1 subtypes and emphasize the need to consider subtype differences when developing alternative treatment options.

# Sub article: Characterization of HIV-1C gp120 in recently and chronically infected individuals in Botswana

**DOI:** 10.7448/IAS.18.5.20464

**Published:** 2015-07-22

**Authors:** Terence Mohammed, Simani Gaseitsiwe, Keikantse Matlhagela, Maitshwarelo Matsheka, Sikhulile Moyo, Rosemary Musonda

**Affiliations:** 1 Research Laboratory, Botswana Harvard AIDS Institute Partnership, Gaborone, Botswana; 2 School of Medicine, University of Botswana, Gaborone, Botswana; 3 Biological Sciences, University of Botswana, Gaborone, Botswana

## Abstract

**Introduction:**

Viral diversity provides a major challenge in the development of a vaccine against HIV-1. A potential target for HIV-1 vaccines is gp120 envelope protein, which is involved in viral entry and is a target of the host immune system. It has been shown that Envelope characteristics have a role to play in disease progression. However some studies have demonstrated conflicting results. In this study, we aim to analyze HIV-1gp120 characteristics, specifically: potential N- glycosylation sites, amino acid sequence length and net electric charge in cell associated and cell free RNA derived from recently and chronically infected individuals in Botswana.

**Methods:**

This was a retrospective study using stored samples collected from treatment naïve HIV-1C infected cohorts at Botswana Harvard AIDS Institute Partnership, representing recently infected and long term infection as determined by serological assays for recency and longitudinal follow up. A 1200 base pairs fragment of V1 to V5 region of gp120 was amplified by nested PCR and sequenced on both strands using Big Dye Technology in proviral DNA and cell free RNA. Potential N-glycosylation sites were determined using Los Alamos HIV sequence database while subtype was assigned using REGA HIV subtyping tool.

**Results:**

There was a significant increase in amino acid sequence length of V2 (p=0.027) and V4 (p=0.0099) in proviral DNA in the chronic stage as compared to the recent stage of infection. Similar changes were also observed in cell free RNA in V4 (p=0.0074). In addition, the number of potential N-linked glycosylation sites in proviral DNA was significantly increased in chronic infection in V4 (p=0.0253). No significant changes in net electric charges were observed. There was an association between viral load and V4 region (p<0.001). All samples were classified as subtype C.

**Conclusions:**

The increase in amino acid sequence length and potential N-Glycosylation sites in the V2 and V4 region may be essential in disease progression. The changes observed in V2 and V4 warrant further investigation. A clear understanding of envelope characteristics is important for development and design of new vaccine and therapeutics.

# Sub article: Early loss of splenic Tfh cells in SIV-infected rhesus macaques

**DOI:** 10.7448/IAS.18.5.20562

**Published:** 2015-07-22

**Authors:** Félicien Moukambi, Henintsoa Rabezanahary, Vasco Rodrigues, Jérôme Estaquier

**Affiliations:** 1 Centre de Recherche du CHU de Quebec, Maladies Infectieuses, Quebec, Canada; 2 Centre de Recherche du CHU de Quebec, Quebec, Canada; 3 CNRS FR 3636 Paris Descartes, Paris, France

## Abstract

**Introduction:**

Follicular T helper cells (Tfh), a subset of CD4 T lymphocytes, are essential for B-cell activation and provide help to B cells in the production of antigen-specific antibodies. Although several studies have analyzed the dynamics of Tfh cells in the context of AIDS by analyzing peripheral blood and LNs of HIV-infected patients, paradoxically, none of these studies in HIV/SIV infection have addressed the role of Tfh cells in the primary organ of B-cell activation, the spleen.

**Methods:**

To address these questions, we have infected rhesus macaques with SIVmac251 (20 AID50). Animals were killed at different time points post-infection and lymphoid organs were recovered. Tfh cells (PD-1^high^CXCR5^+^) and CD4+ T cell subsets were monitored by flow cytometry. Concomitantly, B-cell subsets were also analyzed. CD4 T-cell subsets were sorted and SIV DNA was quantified by RT-PCR.

**Results:**

Herein, we demonstrated for the first time that the percentages and numbers of splenic Tfh cells decrease early during the acute phase in macaques infected with SIV. This profound loss and abnormal differentiation of Tfh is also associated with the loss of memory B-cell subsets. Moreover, SIV DNA is detected in splenic Tfh cells early after infection. Finally, our results showed that the frequency of splenic Tfh and memory B cells are higher in slow-progressor compared to rapid progressor RMs at the chronic phase.

**Conclusions:**

Altogether, our results demonstrate the drastic depletion of splenic memory B cells, which might be related to the loss of fully maturated Tfh cells.

# Sub article: Reliability of rapid HIV-1/HIV-2 INSTI^®^ on plasma and capillary blood for diagnosis of non B subtypes and circulating recombinant forms of HIV-1 circulating in Gabon

**DOI:** 10.7448/IAS.18.5.20465

**Published:** 2015-07-22

**Authors:** Angélique Ndjoyi-Mbiguino, Guy Francis Nzengui Nzengui, Hervé M'Boyis Kamdem, Laurent Bélec

**Affiliations:** 1 Microbiologie, Université des Sciences de la Santé, Libreville, Gabon; 2 Laboratoire National de Référence des Maladies Sexuellement Transmissibles et du SIDA, Département de Microbiologie, Faculté de Médecine de Libreville, Université des Sciences de la Santé, Libreville, Gabon; 3 Virology, Université Paris Descartes, Paris, France

## Abstract

**Introduction:**

*Point-of-care* or “rapid” serologic assays for HIV are widely used in resources-limited setting. Their evaluation in the field carried out independently of the fabricant is crucial to assess their capability to accurately detect non-B subtypes or circulating recombinant form (CRF) of HIV-1.

Our objective was to evaluate the HIV-1/HIV-2 INSTI^®^ test (distributed by Nephrotec, Rungis, France) for the diagnosis of non-B subtypes and CRF of HIV-1 circulating in Gabon, a country of wide genetic diversity.

**Methods:**

A panel of 250 HIV-positive and 250 HIV-negative plasmas was prospectively collected after informed consent in adult patients attending the Laboratoire National de Référence des MST et du SIDA, Libreville, as recommended by the WHO (Service delivery approaches to HIV testing and counselling: A strategic policy framework; 2012). The reference HIV serology consisted of ImmunoComb II HIV1&2 BiSpot (Inverness Medical Innovations, Yavne, Israel) as screening test followed by confirmatory Western blot (New Lav Blot I, Bio-Rad, Marnes-la-Coquette, France). All HIV-positive plasma were furthermore subjected to HIV genotyping by *pol* nested PCR, amplicons sequencing, and analysis of resulting FASTA sequences by Genotyping software from NCBI. A subgroup of 1 out of 10 patients was also tested in parallel with finger-stick whole blood INSTI^®^ test.

**Results:**

All HIV-1 belong to HIV-1 group M with broad HIV-1 genetic diversity as assessed using *pol* sequences (CRF02_AG (53%), CRF14 (18%), CRF15 (12%), CRF01_AE (8%), A1 (4%), G (2%), K (2%), B (1%)). Among 250 HIV-infected and 250 HIV-negative plasmas, 250 and 249, respectively, were positive or negative by INSTI^®^. Thus, INSTI^®^ test sensitivity and specificity were 100% and 99.6%, respectively; positive and negative predictive values in Gabon were 91.5% and 100%, respectively. For the major subtype CRF_02AG, sensitivity and specificity were 100%. Finally, all 50 patients tested in parallel using plasma and capillary blood and were identified similarly.

**Conclusions:**

HIV-1/HIV-2 INSTI^®^ test is highly reliable for the detection of various non-B HIV-1 antibodies, both in plasma and capillary blood; and it fulfils the WHO criteria for HIV test prequalification. The rapid INSTI^®^ test could be useful for HIV screening in Gabon, as well as in other sub-Saharan African countries.

# Sub article: Evaluation of the Roche COBAS Ampliprep/COBAS TaqMan HIV-1 Qualitative version 2 assay on whole blood using specimens with unknown ARV exposure

**DOI:** 10.7448/IAS.18.5.20466

**Published:** 2015-07-22

**Authors:** Raquel Viana, Thabisile Xaba, Teja Stojiljkovic, Nico Gunther, Carole Wallis

**Affiliations:** 1 Department of Molecular Pathology, Lancet Laboratories and BARC-SA, Johannesburg, South Africa; 2 Diagnostics Division, Roche Products (PTY) LTD, Johannesburg, South Africa

## Abstract

**Introduction:**

Roche COBAS Ampliprep/COBAS TaqMan HIV-1 Qualitative version 2 (TaqMan v2 qual) has recently been released for testing of dried blood spot (DBS) for infants and plasma for adults that are antiretroviral (ARV) naïve; however, ARV status of patients is often unknown. This study evaluated the use of whole blood (WB) for HIV-1 detection using TaqMan v2 qual.

**Methods:**

133 samples (125 EDTA, 8 Virology Quality Assurance (VQA) WB) were used with known HIV-1 status (positive, n=75; negative, n=58) as per Roche Amplicor HIV-1 DNA PCR assay v1.5 (Roche v1.5). EDTA samples were split: 1 mL plasma, 100 µL WB and 70 µL DBS. Samples were processed using TaqMan v2 qual according to manufacturer's instructions and results compared to Roche v1.5. Sensitivity and specificity were determined for each sample type and compared to EDTA plasma viral load. Seven WB samples (HIV-1 positive, n=4; HIV-1 negative, n=3) were evaluated for reproducibility and precision using the TaqMan v2 qual.

**Results:**

Of the 69 Roche v1.5 HIV-1 positive samples, 68 were detected using TaqMan v2 qual DBS or WB; whereas only 60 were detected using TaqMan v2 qual plasma. HIV-1 positive samples missed had either a viral load of not detected or <20 RNA copies/mL. The TaqMan v2 qual plasma samples missed 13% of HIV-1 positive samples. No false positives were observed across the three different matrixes evaluated. Of the 8 VQA WB samples tested on TaqMan v2 qual and Roche v1.5, 100% concordance was observed (n=6, HIV-1 positive; n=2, HIV-1 negative). Diagnostic sensitivity and specificity are detailed in the table below. Reproducibility and precision was 100% for all samples tested.

**Conclusions:**

TaqMan v2 qual using DBS or WB had the highest sensitivity when compared to Roche v1.5 (98.5%). Plasma samples on TaqMan v2 qual missed 13% of HIV-1 positive samples. With the increase of microbicide use, pre-exposure prophylaxis and reported poor disclosure of prior ARV use, this study indicates that plasma samples are not the ideal sample matrix for testing adults when ARV exposure is unknown. The high percentage of adult samples that were missed would have serious implications for decreasing HIV-1 transmission rates.

**Abstract WEPDB0102–Table 1:** Diagnostic Sensitivity and Specificity of the Roche COBAS Ampliprep/COBAS TaqMan HIV-1 Qualitative version 2 compared to the Roche Amplicor HIV-1 DNA PCR assay v1.5

Roche COBAS Ampliprep/COBAS TaqMan HIV-1 Qualitative version 2 sample type	Sensitivity	Specificity
Whole blood	98.5%	100%
Plasma	87.0%	100%
Dried blood spot	98.5%	100%

# Sub article: CD4 count at antiretroviral therapy initiation and the risk of loss to follow-up: results from a multicentre cohort study

**DOI:** 10.7448/IAS.18.5.20467

**Published:** 2015-07-22

**Authors:** Anna Grimsrud, Morna Cornell, Michael Schomaker, Matthew P Fox, Catherine Orrell, Hans Prozesky, Kathryn Stinson, Frank Tanser, Matthias Egger, Landon Myer, International Epidemiologic Databases to Evaluate AIDS Southern Africa Collaboration (IeDEA-SA)

**Affiliations:** 1 Division of Epidemiology & Biostatistics, University of Cape Town, Cape Town, South Africa; 2 Centre for Infectious Disease Epidemiology & Research, University of Cape Town, Cape Town, South Africa; 3 Center for Global Health & Development, Boston University, Boston, United States; 4 Health Economics and Epidemiology Research Office, University of the Witwatersrand, Johannesburg, South Africa; 5 Department of Epidemiology, Boston University, Boston, United States; 6 Desmond Tutu HIV Centre, University of Cape Town, Cape Town, South Africa; 7 Division of Infectious Diseases, Department of Medicine, University of Stellenbosch, Stellenbosch, South Africa; 8 Tygerberg Academic Hospital, Cape Town, South Africa; 9 Médecins Sans Frontières, Cape Town, South Africa; 10 Africa Centre for Health and Population Studies, University of KwaZulu-Natal, Mtubatuba, South Africa; 11 Division of International and Environmental Health, Institute of Social and Preventive Medicine, University of Bern, Bern, Switzerland; 12 Division of Epidemiology and Biostatistics, University of Cape Town, Cape Town, South Africa

## Abstract

**Introduction:**

Over the past decade of antiretroviral therapy (ART) scale-up, median CD4 counts at ART initiation have increased and ART initiation is recommended at progressively higher CD4 thresholds. However data on the relationship between CD4 count at ART initiation and loss to follow-up (LTFU) are limited and conflicting. We investigated the association between higher CD4 counts at ART initiation and LTFU in South Africa (SA).

**Methods:**

All adults initiating ART between 2008 and 2012 at 3 public sector sites in SA were included. LTFU was defined as no clinic visit in the six months before database closure. The Kaplan-Meier estimator and Cox's models examined the relationship between CD4 count at ART initiation and 24-month LTFU. Estimates of corrected LTFU were generated adjusting observed LTFU for unascertained deaths through linkage via identification numbers (IDs) with the SA National Population Register. Final models were adjusted for patient demographics, year of ART initiation, and programme expansion.

**Results:**

Among 17,038 patients, the median CD4 at initiation increased from 119 (interquartile range (IQR): 54–180) in 2008 to 257 (IQR: 175–318) in 2012. In unadjusted models, observed LTFU was associated with both CD4 counts<100 cells/mL and CD4 counts≥300 cells/mL compared to those with a CD4 count 150–199 cells/mL. After adjustment, patients with CD4 counts ≥300 cells/mL were 1.35 (95% CI: 1.12–1.63) times as likely to be LTFU after 24 months compared to those with a CD4 count 150–199 cells/mL. Correction for unascertained deaths attenuated the association between CD4 counts <100 cells/mL and LTFU while the association between CD4 counts ≥300 cells/mL and LTFU persisted (Figure 1). Increases in LTFU observed in patients with CD4 counts ≥300 cells/mL was greatest in the first three months on treatment. In sensitivity analyses imputing missing CD4 values at ART initiation and using inverse probability weighting to account for missing IDs, the association between higher CD4 counts and increased LTFU persisted.

**Conclusions:**

Patients initiating ART at higher CD4 counts may be at increased risk for LTFU, particularly early after ART initiation. With programmes initiating patients at progressively higher CD4 counts models of ART delivery need to be reoriented to support long-term retention.

**Abstract WEPDB0103–Figure 1:** Adjusted 12-month log hazard ratios of observed and corrected LTFU from Cox's proportional hazards models by CD4 count at ART initiation.

# Sub article: Clinical decision and outcomes of patients suspected of treatment failure and tested for HIV-viral load at the Infectious Diseases Institute (IDI), Kampala, Uganda

**DOI:** 10.7448/IAS.18.5.20468

**Published:** 2015-07-22

**Authors:** Mark Steven Nsumba, Castelnuovo Barbara, Rachel Zimaze Musomba, Arvind Kaimal, Kalule Ivan, Lwanga Isaac, John Laurence, Parkes-Ratanshi Rosalind, Kambugu Andrew

**Affiliations:** 1 Research Department, Infectious Disease Institute, Kampala, Uganda; 2 Makerere University, Kampala, Uganda; 3 Infectious Disease Institute, Kampala, Uganda; 4 PCT, Infectious Disease Institute, Kampala, Uganda

## Abstract

**Introduction:**

WHO now recommends routine viral load (VL) monitoring, and the scale up of this has started in sub-Saharan Africa. Recent publications from the region suggest that often patients with detectable VL delay switching to second line ART or are not switched.

**Objective:**

To evaluate the outcome of patients with a VL>1000 copies/mL accessing care at a large urban HIV Centre in Kampala, Uganda.

**Methods:**

At IDI VL tests have been available since 2005. Until December 2014 these were reserved for patients with documented immunological or clinical failure. Those patients with detectable VL are managed through a treatment failure path-way consisting of: 1) review of the results by the clinician, 2) case discussion in the weekly multidisciplinary “switch-meeting” 3) follow up by a clinician and counsellor based on the decision reached during the “switch-meeting.” We performed a retrospective audit of a sample of patients on first line ART with VL >1000 in 2014; data was extracted from 95 randomly sampled clinic files and the clinic database.

**Results:**

1093 patients on first line ART were tested for VL in 2014, of which 365 (33.4%) had a detectable VL; of these 95 (26%) clinical files were sampled. Median log_10_ VL was 4.9 (IQR: 4.7–5.3).

The diagram summarizes the action taken for the 95 sampled patients stratified by referral to the treatment failure path-way. 60/95 (63.1%) were switched to 2^nd^-line after a median time of 49 days (IQR: 14–84). Of note an action was taken for all patients referred to the treatment failure path-way.

**Conclusions:**

The majority (65%) of patients with a detectable VL were switched to 2^nd^-line, and an additional 28% had an action taken. This is a favourable outcome compared to outcomes in other treatment centres around SSA, and we believe that the “switch meeting” model has helped to ensure that action is taken. We advocate that this additional step be considered in WHO and national guidelines to ensure adherence strengthening and prompt switch to second line in patients failing ART.

**Abstract WEPDB0104–Figure 1:** Action taken for the sampled patients with viral failure stratified by referral to the treatment failure path-way.

# Sub article: Classification of HIV virological failure using whole blood versus plasma viral load

**DOI:** 10.7448/IAS.18.5.20469

**Published:** 2015-07-22

**Authors:** Aabida Khan, Lucia Hans, Luis Gonzales, Sergio Carmona, Nei-Yuan Hsiao

**Affiliations:** 1 Division of Medical Virology, University of Cape Town and National Health Laboratory Service, Cape Town, South Africa; 2 Department of Haematology and Molecular Medicine, University of the Witwatersrand and National Health Laboratory Service, Johannesburg, South Africa; 3 Alere Health Care, Johannesburg, South Africa

## Abstract

**Introduction:**

In resource limited settings, timely plasma separation and transportation to centralized laboratories is a major challenge to the scale-up of viral load (VL) testing. Whole blood (WB) collection and testing through either dried blood spots (DBS) or point-of-care VL assays are potential solutions. However, there is limited evidence on the performance of WB-based VL assays.

**Methods:**

We evaluated three WB VL testing platforms, Alere q HIV-1/2, DBS Abbott RealTime HIV-1 and Roche CAP/CTM HIV-1 (DBS, free virus elution protocol) using routine clinical samples across a wide viral load spectrum chosen from South African public sector patients on combination antiretroviral therapy. Abbott RealTime HIV-1 was used as gold standard and virological failure (VF) was defined for plasma at 1000 copies/mL.

**Results:**

Of the 299 samples selected, 153 (51%) had plasma VL>1000 copies/mL. Abbott DBS VL had the best overall VL correlation with its plasma counterpart (r^2^=0.76), followed by the Roche DBS VL (r^2^=0.62) and Alere q HIV-1/2 (r^2^=0.46). Among samples with VF, Alere q HIV-1/2 and Abbott DBS assays were highly sensitive, correctly classified 100% and 98% of the samples, respectively. Roche DBS assay was only able to identify 53% of the VF samples correctly. For samples with plasma VL<1000 copies/mL there were upward misclassification due to further VL>1000 copies/mL identified by WB VL on both Alere q HIV-1/2 (81%) and Abbott DBS VL (21%) when compared to the plasma reference, while Roche DBS VL showed 99% agreement in this category. Receiver operating characteristic analysis revealed that the threshold of log_10_ 4.12, 3.43 and 2.60 copies/mL provided the best overall VF classification for Alere q HIV-1/2 (85%), Abbott DBS VL (94%) and Roche DBS VL (82%), respectively.

**Conclusions:**

Variability was noted between the different WB VL assays with difficulties assigning a uniform threshold across all platforms, reflecting the differences in sample treatment/processing (DBS versus fresh blood samples) and sample input volume. The performance at 1000 copies/mL of DBS protocols and point-of-care devices remains significantly varied and further development is required to ensure minimal VF misclassification.

**Abstract WEPDB0105–Table 1:** Percent of correct classification of VF by WB HIV VL

Abbott RealTime HIV-1 Plasma VL	LDL (lower than detectable limit)	Not LDL <1000 copies/mL	1000–10,000 copies/mL	>10,000 copies/mL
N	94	52	52	101
Alere q HIV-1/2 WB% correct classification	22%	14%	100%	100%
DBS Abbott RealTime HIV-1% correct classification	87%	63%	94%	100%
DBS Roche CAP/CTM HIV-1% correct classification	100%	98%	0%	80%

# Sub article: Geographic origin trends among HIV+ mothers and children in Canada and impact on vertical HIV transmission rates

**DOI:** 10.7448/IAS.18.5.20470

**Published:** 2015-07-22

**Authors:** Jason Brophy, Terry Lee, Laura Sauve, Ari Bitnun, Joel Singer, Fatima Kakkar, Normand Lapointe, Ariane Alimenti, Deborah Money, Wendy Vaudry, Lindy Samson, for the Canadian Pediatric and Perinatal AIDS Research Group

**Affiliations:** 1 Children's Hospital of Eastern Ontario, Ottawa, Canada; 2 Department of Pediatrics, University of Ottawa, Ottawa, Canada; 3 CIHR – Canadian HIV Trials Network, Vancouver, Canada; 4 BC Women's Hospital and Health Centre, Vancouver, Canada; 5 Department of Pediatrics, University of British Columbia, Vancouver, Canada; 6 The Hospital for Sick Children, Toronto, Canada; 7 Department of Pediatrics, University of Toronto, Toronto, Canada; 8 CHU Ste-Justine, Montréal, Canada; 9 Department of Pediatrics, Université de Montréal, Montréal, Canada; 10 Department of Obstetrics, University of British Columbia, Vancouver, Canada; 11 Women's Health Research Institute, Vancouver, Canada; 12 Stollery Children's Hospital, Edmonton, Canada; 13 Department of Pediatrics, University of Alberta, Edmonton, Canada

## Abstract

**Introduction:**

Migration contributes significantly to new HIVcases in Canada. This study describes geographic origin trends among HIV+ mothers and perinatally infected children and the impact of geographic origin on vertical HIV transmission (VT) rates among HIV+ mother-infant pairs (MIP) in Canada from 1990 to 2013.

**Methods:**

The Canadian Perinatal HIV Surveillance Program collects data at 22 centres. The primary focus is on MIP with an infant born in Canada and identified prior to/within three months of birth; MIP with Canadian-born infants identified after three months and HIV+ children born abroad are also tracked. Data reviewed for this study included: maternal country of origin, clinical characteristics, antiretroviral usage and infant outcome. Logistic regression determined VT rate differences for foreign-born (FBM) versus Canadian-born mothers (CBM).

**Results:**

Among 3877 MIP, 2089 (53.9%) mothers were FBM. Of 1481 (70.9%) African mothers, 30.7%, 20.1%, 17.7% and 16.7% came from East, Central, Horn and West Africa, respectively. CBM accounted for 66.7% (971/1456) in Western/Central Canada, whereas FBM predominated in Ontario (945/1357, 69.6%; greatest proportion East African, 25.0%) and Quebec (713/1020, 69.9%; greatest proportion Caribbean, 36.2%). The largest numbers of FBM originated from Haiti (12.5%), Ethiopia (8.7%), Congo (7.0%), Zimbabwe (5.4%) and Nigeria (4.6%). In the pre-cART era (1990–1996), Haiti contributed 29.9% (90/301) of FBM, decreasing to 13.0% (119/918) in 1997–2007 and 6.6% (52/782) in 2008–2013. Since 2008, Ethiopia (80/782, 10.2%), Congo (64/782, 8.2%) and Nigeria (62/782, 7.9%) predominated.

VT rate among Canadian-born children from 1990 to 2013 was 3.8% (3.0% among FBM) and 1.2% from 2008 to 2013 (0.7% among FBM). African mothers had lower risk of VT (1990–2013: OR = 0.45, 95% CI 0.29–0.71; 2008–2013: OR=0.35, 95% CI 0.12–1.08) compared to CBM; no differences were seen for other regions.

Of 353 HIV+ children (born in Canada or abroad) with FBM, the greatest numbers came from Haiti (48, 13.6%), Ethiopia (33, 9.3%), Burundi (30, 8.5%) and Congo (15, 4.2%).

**Conclusions:**

Geographic origins of HIV+ FBM in Canada have changed over time, shifting from predominantly Haitian in the pre-cART era to predominantly African more recently. African mothers have lower VT rates than CBM. Understanding country-specific cultural and obstetrical/paediatric health issues is imperative to providing optimal care.

# Sub article: The Canadian perinatal HIV surveillance programme (CPHSP): programme description and trends in demographics, treatment and transmission

**DOI:** 10.7448/IAS.18.5.20471

**Published:** 2015-07-22

**Authors:** Joel Singer, Ari Bitnun, Terry Lee, Lindy Samson, Jason Brophy, Deborah Money, Ariane Alimenti, Wendy Vaudry, Fatima Kakkar, Normand Lapointe, Laura Sauve, Canadian Pediatric & Perinatal AIDS Research Group

**Affiliations:** 1 Methodology and Statistics, School of Population and Public Health, University of British Columbia, Vancouver, Canada; 2 CIHR Canadian HIV Trials Network, Vancouver, Canada; 3 Hospital for Sick Children, University of Toronto, Toronto, Canada; 4 CIHR Canadian HIV Clinical Trials Network, Vancouver, Canada; 5 Children's Hospital of Eastern Ontario, University of Ottawa, Ottawa, Canada; 6 BC Women's Hospital and Health Centre, University of British Columbia, Vancouver, Canada; 7 Stollery Children's Hospital, University of Alberta, Edmonton, Canada; 8 Hopital Ste-Justine, University of Montreal, Montreal, Canada

## Abstract

**Introduction:**

The Canadian Perinatal HIV Surveillance Program (CPHSP) is an active surveillance programme generating national data HIV+ women and their infants in Canada since 1990. We describe the CPHSP’s evolving methodology and analyze mother-infant pair (MIP) demographics, antiretroviral treatment and vertical transmission (VT) rates in Canada from 1990 to 2013.

**Methods:**

MIPs are identified at 22 centres following obstetric or paediatric referral for care. Data is entered via a secure web-based Oracle database, which is managed and analyzed by the CIHR-Canadian HIV Trials Network. A nationally representative steering committee provides direction and oversight. Data collected include maternal characteristics, antiretroviral therapy (ART) and infant outcome. VT rates are based on data of MIP delivered in Canada and identified within three months after birth; infants identified beyond three months of birth are tracked separately.

**Results:**

Among 2914 MIP from the combination ART (cART) era (1997–2013), the overall VT rate was 2.1% but only 0.7% in MIP receiving cART and 0.1% in women receiving >4 weeks of cART. Of 200 identified HIV+ women giving birth in Canada in 2013, 76% acquired HIV heterosexually, 17% through injection drug use (IDU) and 2% perinatally; 53% of mothers were Black and 23% Aboriginal. The proportion untreated steadily decreased from 20.3% in 1997 to 3.0% in 2013. Aboriginal women (7%) continued to represent the largest proportion of untreated women (7%) in 2013, though this decreased from a peak of over 20% during the period 2005–2009. A similar improvement was seen among IDU, with only 3% untreated in 2013. In 2013, seven (3.5%) women had no antenatal cART or suboptimal treatment, the lowest annual number and percentage in the cART era, resulting in two children becoming infected.

**Conclusions:**

The CPHSP allows for comprehensive identification of perinatal HIV exposure and outcome trends in Canada. Ongoing challenges include ensuring all MIPS are captured given Canada’s geographically and demographically diverse population and low HIV prevalence. Despite continued improvement in treatment access for pregnant HIV+ women, VT continues to occur with Aboriginal women being at greater risk of inadequate treatment and VT.

# Sub article: HIV acquisition after arrival in France among sub-Saharan African migrants living with HIV in Paris area. Estimations from the ANRS PARCOURS study

**DOI:** 10.7448/IAS.18.5.20472

**Published:** 2015-07-22

**Authors:** Annabel Desgrees du Lou, Julie Pannetier, Andrainolo Ravalihasy, Anne Gosselin, Virginie Supervie, Nathalie Bajos, France Lert, Nathalie Lydie, Rosemary Dray-Spira

**Affiliations:** 1 IRD, CEPED, Paris, France; 2 INSERM, IPLESP, Paris, France; 3 INSERM, CESP, Le Kremin Bicêtre, France; 4 INSERM, CESP, Villejuif, France; 5 INPES, Saint Denis, France

## Abstract

**Introduction:**

HIV acquisition among sub-Saharan migrants living in Europe has long been considered to predominantly occur before migration because of generalized HIV epidemics in sub-Saharan African countries. Recent evidence suggests that a substantial proportion have acquired HIV while they were living in Europe. In the UK, this proportion was recently estimated at 31% using a CD4-based modelling approach. Such an estimate is not currently available for France.

**Methods:**

We estimated the proportion of sub-Saharan migrants who acquired HIV infection after their arrival in France using life-event and clinical information on a random sample of HIV-infected hospital outpatients born in sub-Saharan Africa in Paris region. We assumed that HIV infection had probably been acquired in France if at least one of the following life-event criterion was fulfilled: 1) HIV diagnosis >10 years after arrival in France, 2) ≥1 negative HIV test in France, and 3) sexual debut after arrival in France. If none of these criteria was fulfilled, we estimated the duration from HIV infection based on first CD4 count measurement using statistical modelling. Infection was assigned in France if, out of 500 durations estimated for each individual, >50% (median scenario) or >95% (conservative scenario) fell within the period while individuals were living in France.

**Results:**

Of the 898 HIV-infected adults born in sub-Saharan Africa included in the analysis, we estimated that 49% (95% confidence interval: 45–53) in the median scenario and 35% (31–39) in the conservative scenario acquired HIV while living in France. This proportion was lower for women than men (30% (25–35) vs. 44% (37–51) in the conservative scenario) and increased with duration in France.

**Conclusions:**

The proportion of sub-Saharan African migrants having acquired HIV infection while living in France is high, highlighting the need for improved focused HIV prevention. This requires a better understanding of the determinants of HIV infection in France in this population.

**Abstract WEPDC0103–Figure 1:** Flow chart – assignment of HIV acquisition.

**Abstract WEPDC0103–Table 1:** France HIV acquisition – conservative scenario

		Men				Women			
			
		N	Weighted %	95% CI	p value	N	Weighted %	95% CI	p value
Overall		348	43.9	37.4–50.6		550	30.0	25.1–35.4	
Age at arrival in France	<25 yr	84	78.1	65.5–87.1	<0.001	171	54.1	46.5–61.5	<0.001
	25–34 yr	139	44.3	35.9–53.2		251	24.5	17.7–32.8	
	35 yr and more	125	19.8	13.0–28.8		128	8.4	4.4–15.5	
Number of years in France	0–2	137	10.3	4.9–20.6	<0.001	254	5.4	3.2–8.9	<0.001
prior to diagnosis	3–5	45	19.3	7.0–43.0		93	23.4	18.6–29.0	
	6–9	39	54.0	36.2–70.9		67	52.5	36.3–68.3	
	10 or more	106	93.5	85.4–97.3		95	86.0	77.0–91.9	

# Sub article: Evidence of local HIV transmission in the African community of King County, Washington

**DOI:** 10.7448/IAS.18.5.20473

**Published:** 2015-07-22

**Authors:** Roxanne Kerani, Joshua Herbeck, Susan Buskin, Julia Dombrowski, Amy Bennett, Elizabeth Barash, Lindley Barbee, Matthew Golden

**Affiliations:** 1 Medicine, University of Washington, Seattle, United States; 2 HIV/STD Program, Public Health – Seattle and King County, Seattle, United States; 3 Global Health, University of Washington, Seattle, United States; 4 Communicable Disease Epidemiology, Public Health – Seattle and King County, Seattle, United States

## Abstract

**Introduction:**

In many parts of the U.S., immigrants from sub-Saharan Africa comprise a large proportion of heterosexual HIV cases. However, little is known about the frequency of ongoing HIV transmission within these communities.

**Methods:**

Public Health-Seattle and King County staff routinely interview patients newly reported with HIV infection, and attempt to contact sex partners to ensure notification and HIV testing. We describe the characteristics, testing history and partner outcomes for African-born persons newly reported with HIV infection in King County (KC), WA from 1/1/2010 to 12/31/2013. Additionally, we reconstructed an HIV-1 pol phylogeny for 1430 cases diagnosed in KC 2008–2014, with 100 sequences each from Kenya and Ethiopia added for African references.

**Results:**

During the study period, 1148 adults were reported with HIV in KC, including 101 (8.8%) born in Africa. Of 63 cases in African-born individuals with new HIV diagnoses, 49 (77.8%) were interviewed for partner services. Seven reported being diagnosed with HIV-infection before U.S. arrival and were excluded from further analysis, leaving 42 individuals. Median time from U.S. arrival to HIV diagnosis was 7.0 years (range: 8 days–26.7 years). Most were born in East African countries (N=34, 81.0%). Twenty-seven (64.3%) were women; mean age was 42.6 years (range: 24.9–62.2). Sixteen (38.1%) cases reported at least one negative test prior to HIV diagnosis, and 11 (31.4%) reported >1 negative HIV test after U.S. arrival. Pol genotypes were available for seven of these 11 cases; for six of these seven, a local case was the nearest phylogenetic neighbour, and two were infected with subtype B virus. This suggests local transmission sources for these six cases. The 42 newly diagnosed individuals identified 47 partners; six (12.8%) partners had been diagnosed with HIV infection prior to the investigation. Thirteen partners were newly HIV tested as a result of index patients’ HIV diagnoses; five (38.5%) were HIV-infected. Of the 11 partners who were previously positive (6) or newly diagnosed (5), seven were interviewed and six were African-born.

**Conclusions:**

We found substantial evidence of ongoing HIV transmission in the African community of KC. Additional efforts are needed to increase HIV testing and prevention among African immigrants in the U.S.

# Sub article: Heterogeneity of the HIV epidemic in rural Africa: findings from a geospatially informed study of HIV epidemiology in fishing, trading, and agrarian communities in Rakai, Uganda

**DOI:** 10.7448/IAS.18.5.20474

**Published:** 2015-07-22

**Authors:** Larry Chang, Mary K Grabowski, Robert Ssekubugu, Fred Nalugoda, Steven Reynolds, Justin Lessler, Sean Moore, Thomas Quinn, Ronald Gray, David Serwadda, Maria Wawer

**Affiliations:** 1 Division of Infectious Diseases, Johns Hopkins School of Medicine, Baltimore, United States; 2 Department of Epidemiology, Infectious Disease Epidemiology, Johns Hopkins Bloomberg School of Public Health, Baltimore, United States; 3 Rakai Health Sciences Program, Kalisizo, Uganda; 4 Laboratory of Immunoregulation, Division of Intramural Research, National Institute for Allergy and Infectious Diseases, National Institutes of Health, Bethesda, United States

## Abstract

**Introduction:**

National and district level HIV prevalence rates may obscure substantial variation of HIV disease burden at the community level. Understanding the extent to which HIV differs across communities and the drivers of disparities and similarities within individual districts may offer opportunities for a more effective, targeted HIV response.

**Methods:**

HIV prevalence and risk behaviours were assessed among 17,109 individuals (53.8% female vs. 46.2% male) in 40 communities in Rakai District, Uganda between August 2011 and October 2013 through the population-based Rakai Community Cohort Study. Communities were classified as lakeside fish landing sites (n=4), agrarian (n=27) or trading communities (n=9) based upon occupation analysis. HIV prevalence was geospatially mapped using Bayesian methods and variability across and within community classifications was characterized. Differences in risk behaviours between communities were assessed using modified Poisson regression models.

**Results:**

There was large variation in HIV prevalence, ranging from 9 to 43%, across communities (see Figure below). Fish landing sites had a mean HIV prevalence of 41% (range: 37–43%). Mean HIV prevalence in trading communities was 17% with substantial variability (range: 11–22%) and 14% in agrarian communities, also with substantial variability (range: 9–26%). Agrarian and trading communities in close proximity (<18 km) to fishing landing sites had HIV prevalence ranging from 11 to 26%. Overall, HIV prevalence was higher among women than men (p=0.01), and the disparity was greatest in the fish landing sites (49% vs. 34%). The proportion of males and females reporting =4 sex partners in the last year was 6.4 (95% CI: 4.1–11.0) and 3.2 (95% CI: 2.7–3.8) times higher in fishing communities than in the agrarian/trading population, respectively. Levels of consistent condom use with non-marital partners were significantly lower in the fish landing sites (RR=0.80, 95% CI: 0.69–0.94).

**Conclusions:**

Large variations in HIV prevalence and risk factors across communities in rural Rakai underscores the need for a granular approach to HIV prevention and response based on local assessment of HIV burden and risks and locally tailored interventions that may include targeting of high risk groups such as those in fish landing sites.

**Abstract WEPDC0105–Figure 1:** HIV prevalence in each of the 40 RCCS communities.

# Sub article: Cost-effectiveness of implementing CRAG-LFA screening for cryptococcal meningitis among people living with HIV in Uganda

**DOI:** 10.7448/IAS.18.5.20475

**Published:** 2015-07-22

**Authors:** Anu Ramachandran, Yukari Manabe, Radha Rajasingham, Maunank Shah

**Affiliations:** 1 Johns Hopkins University School of Medicine, Baltimore, United States; 2 Infectious Disease, Johns Hopkins University School of Medicine, Baltimore, United States; 3 Minneapolis, University of Minnesota, United States

## Abstract

**Introduction:**

Cryptococcal meningitis (CM) constitutes a significant source of morbidity and mortality in resource-limited regions. One million cases occur annually, representing 10–30% of HIV-related death in prevalent regions. Optimal interventions for CM prevention remain unclear. The recently developed serum cryptococcal antigen lateral-flow assay (CRAG-LFA) is highly sensitive and specific, and may allow early detection of subclinical cryptococcemia in those at risk of developing CM. We sought to determine the cost-effectiveness of implementing CRAG-LFA screening for people living with HIV in Uganda compared to other interventions for CM prevention.

**Methods:**

A decision-tree model was constructed to compare three strategies for cryptococcal prevention among people living with HIV (PLWH) with CD4<100: Standard of care (SOC, i.e. no cryptococcal screening), CRAG-LFA screening followed by evaluation and treatment of cryptococcemia or universal primary prophylaxis (UPP) with fluconazole for all patients and no CRAG-LFA screening. Primary outcomes were expected costs, DALY’s and incremental cost-effectiveness ratios (ICERs). In sensitivity analysis, we analyzed the impact of costs, prevalence and alternative clinical algorithms on the cost-effectiveness of CRAG-LFA screening.

**Results:**

CRAG-LFA screening was associated with an ICER of $5.88 per DALY averted compared to SOC, and was highly cost-effective at current willingness to pay thresholds for Uganda. CRAG-LFA screening dominated the UPP intervention (i.e. both cheaper and more effective). Overall, implementation of CRAG-LFA screening was projected to cost $1.46 more per person than SOC, and could reduce the relative risk of cryptococcal-associated mortality by over 40%. When including the cost of lifetime ART, the ICER for CRAG-LFA screening was $557 compared to SOC and still considered cost-effective. In sensitivity analysis, prevalence of baseline CM and cost of the CRAG-LFA influenced cost-effectiveness. In probabilistic sensitivity analysis, the CRAG-LFA screening intervention was cost-effective in 100% of simulations, and cost-saving in 30% of simulations.

**Conclusions:**

CRAG-LFA screening is extremely cost-effective with the potential to prevent significant morbidity and mortality from CM in vulnerable populations, and represents excellent value for money as a screening intervention for HIV programs in Uganda.

**Abstract WEPDD0101–Table 1:** Cost-effectiveness projection results

Intervention	Total Cost	Incremental Cost	Incremental Cost (including lifetime ART)	DALYs Accumulated	Incremental Effectiveness (DALYs averted)	Incremental Cost-Effectiveness Ratio (ICER)	Incremental Cost-Effectiveness Ratio (ICER) including lifetime ART
Standard of Care (SOC)	9.12	REFERENCE	REFERENCE	8.55	REFERENCE	REFERENCE	REFERENCE
CRAG-LFA Screening	10.58	1.46	139.48	8.30	0.25	5.88	557.60
Universal Primary Prophylaxis (UPP)	236.23	227.10	332.19	8.35	0.20	1141.96	1660.95

**Abstract WEPDD0101–Figure 1:** Decision-analysis model schematic. Abbreviations: SOC-Standard of care, UPP-Universal fluconazole primary prophylaxis, CRAG-LFA–cryptococal antigen lateral flow assay, CM-cryptococcal meningitis, WTP-WHO pre-emptive therapy. Decision-analytic model schematic. We modeled progression or relapse of CM over a 5 year time-horizon for a cohort of PLWH with CD4 < 100. In all model arms symptomatic patients at baseline receive evaluation for CM assumed to include a lumbar puncture (LP), and treatment if diagnosed with CM. We assumed ART initiation in all arms. The model explores three interventions for prevention of cryptococcal morbidity for those without a baseline diagnosis of CM: 1) SOC, in which patients receive no CM screening or prophylaxis 2) UPP, in which all asymptomatic patients (and symptomatic patients without CM diagnosis * as noted in the model) receive primary prophylaxis with 200 mg of fluconazole. 3) CRAG-LFA, in which all patients receive serum CRAG-LFA screening. Individuals with positive CRAG were assumed to receive the WHO preemptive treatment for cryptococcemia with fluconazole 800 mg for two weeks, followed by fluconazole 400 mg for eight weeks. CRAG-negative individuals receive no further antifungal therapy.

# Sub article: Lost opportunities to identify and treat HIV-infected patients: results from a comprehensive study of provider-initiated HIV testing and counselling (PITC) in Malawi

**DOI:** 10.7448/IAS.18.5.20476

**Published:** 2015-07-22

**Authors:** Monica Schwarz, Robbie Flick, Mwelura Harawa, Katie Simon, Maria Kim, Jeff Robison, Saeed Ahmed

**Affiliations:** 1 Department of Pathology, University of Utah School of Medicine, Salt Lake City, United States; 2 Baylor College of Medicine – Abbott Fund Children's Clinical Center of Excellence, Lilongwe, Malawi; 3 Infectious Diseases, University of Colorado School of Medicine, Denver, United States; 4 Baylor International Pediatric AIDS Initiative at Texas Children's Hospital, Baylor College of Medicine, Houston, United States; 5 Department of Pediatrics, University of Utah, Salt Lake City, United States

## Abstract

**Introduction:**

Early diagnosis and treatment of HIV improves patient outcomes and minimizes risk of transmission. Provider-initiated testing and counselling (PITC) is an effective case-finding strategy, but implementation models vary. Malawi Ministry of Health (MOH) guidelines recommend routine opt-out PITC, in line with WHO recommendations for countries with generalized epidemics, but little is known about its implementation. Our objective was to assess PITC implementation in Malawi.

**Methods:**

We conducted a cross-sectional study of PITC implementation at 118 clinics and wards within 12 MOH facilities in central Malawi during June–July 2014. Qualitative data detailing PITC practices was collected through structured interviews with 71 providers who conduct HIV testing at their facility, and characterized using standardized definitions (Figure 1). Quantitative data describing patient visits and HIV tests recorded during 2013 was abstracted from MOH HIV testing reports.

**Results:**

Variable models of PITC were reported across facilities and departments (Table 1). Overall, symptom-based PITC was most commonly reported. Only antenatal and maternity (20/24) departments reported implementing routine opt-out testing. Use of a PITC register varied significantly according to department type.

Only 7.7% (86,657/1,102,802) of patient visits in 2013 included an HIV test. Subgroup analysis of TB and antenatal clinics with available data demonstrated that HIV status was ascertained in 94.3% (5293/5615) and 86.8% (26,831/30,961) of patients, respectively.

Providers most commonly cited test kit shortages (71/71 providers), inadequate physical space (58/71) and inadequate number of HIV counsellors (32/71) as challenges in PITC implementation. Providers from inpatient units cited the inability to test on weekends (8/16).

**Conclusions:**

Various models of PITC concurrently exist at MOH facilities in Malawi. Only antenatal and maternity clinics demonstrated high rates of routine opt-out PITC. The low ratio of facility visits that included an HIV test suggest missed opportunities for HIV testing. However, the high proportion of patients at TB and antenatal clinics with known HIV status suggest routine testing is feasible. These results underscore the need to develop clear, standardized PITC protocols and tools, and to address obstacles of limited health commodities, infrastructure and human resources.

**Abstract WEPDD0102–Figure 1:** Definitions of PITC models.

**Abstract WEPDD0102–Table 1:** Reported PITC model and use of PITC register

	*Types of PITC reported, n (%)*			
		
Department type	Routine opt-out	Routine opt-in	Symptom-based	PITC register in use, n (%)
TB Clinic	5/12 (42)	7/12 (58)	0/12 (0)	12/12 (100)
Antenatal clinic and maternity ward	20/24 (83)	4/24 (17)	0/24 (0)	24/24 (100)
Family planning clinic	1/11 (9)	7/11 (64)	3/11 (27)	8/11 (73)
STI clinic	3/6 (50)	3/6 (50)	0/6 (0)	6/6 (100)
Outpatient Department, Under-5 clinic, and immunization clinic	4/36 (11)	4/36 (11)	28/36 (78)	9/36 (25)
Malnutrition clinic	7/10 (7)	2/10 (20)	1/10 (10)	5/10 (50)
Adult and paediatric inpatient wards	1/19 (5)	3/19 (16)	15/19 (79)	5/19 (26)
Totals	41/118 (35)	30/118 (25)	47/118 (40)	69/118 (59)

# Sub article: Evaluation of HIV PIMA™CD4 point-of-care test operation by trained non-health workers in rural health centres in Chiradzulu District, Malawi

**DOI:** 10.7448/IAS.18.5.20477

**Published:** 2015-07-22

**Authors:** Birgit Schramm, Aliaa Tayea, Liselotte Wolters, Sarala Nicholas, Charlie Willy Masiku, Dawie Baxter Zolowere, Eustice Mhango, James Raphael Kandulu, Jean-Francois Etard, Isabel Amaros, Elisabeth Szumilin, Monique Gueguen

**Affiliations:** 1 Epicentre, Paris, France; 2 Medecins sans Frontieres, Chiradzulu, Malawi; 3 Medecins sans Frontieres, Lilongwe, Malawi; 4 Ministry of Health, Chiradzulu, Malawi; 5 Ministry of Health, Lilongwe, Malawi; 6 UMI 233 Institut de Recherche pour le Développement, Université de Montpellier, Montpellier, France; 7 Medecins sans Frontieres, Paris, France

## Abstract

**Introduction:**

CD4 count is essential to identify antiretroviral treatment (ART) eligibility. For over a decade, Médecins Sans Frontières and the Ministry of Health provides ART in 10 rural health centres (HCs) in Chiradzulu District, Malawi. From June 2013, Alere’s PIMA^TM^ CD4 point-of-care (POC) test is being implemented in the HCs. Shortage of health care- and laboratory staff is an issue in this setting. We assessed task-shifting of PIMA CD4 test operation to non-health workers living in the community around the HCs.

**Methods:**

Four non-health workers received a one-week structured training on PIMA CD4 POC operation. Between June 2014 and January 2015, 331 venous blood samples of pre-ART and ART-patients attending routine CD4-testing in two rural HCs were included. Each sample was assessed on site with PIMA by a lab technician (LT) and a trained community worker (TCW), and measured with PartecCyflow^®^ counter at district hospital. Kappa-coefficient and percent agreement for CD4-classification below and above relevant thresholds were obtained. Bias and limits of agreement (LOA) were assessed for absolute CD4 counts. PIMA error-rates and failed runs (2 consecutive errors) were recorded and TCW-operator acceptability assessed.

**Results:**

Three-hundred-twenty-eight venous blood samples (85% ART-patients, 68.5% female) were included. Median CD4 count (LT PIMA) was 425 cells/µL (IQR: 323, 570). Error rates were low (LT: 1.2% vs. TCW: 2.4%, p=0.34) and no failed runs occurred. Good agreement was achieved for PIMA results by LTs versus TCWs for CD4 threshold 350 cells/µL (91.7% (95% CI: 88.2–94.5); kappa=0.80) and 500 cells/µL (91.1% (95% CI: 87.5–93.9); kappa=0.80). The mean bias (TCW-PIMA minus LT-PIMA) was low (−2.2 cells/µL (LOA: 137.4, −141.9)). Bias and LOA comparing PIMA results by LTs or TCW versus Partec was similar (LT-PIMA minus Partec: −46.4 cells/µL (95.9, −188.8)); (TCW-PIMA minus Partec: −46.5 cells/µL (118.0 −211.0)). TCWs rated PIMA operation as very easy.

**Conclusions:**

Adequately trained community workers delivered CD4 results equivalent to lab technicians with PIMA POC in health centre laboratories. Task shifting of simplified CD4 POC-technologies to trained non-health care staff can serve as a key strategy to ensure sustainable provision of CD4-testing in support of ART-initiation in rural facilities.

# Sub article: Improving dried blood spot transport logistics for early infant diagnosis in Nigeria: *the SPEEiD model*

**DOI:** 10.7448/IAS.18.5.20478

**Published:** 2015-07-22

**Authors:** Nwokedi Ndulue, Andrew Etsetowaghan, Chima Gabriel, Jimoh Ibrahim

**Affiliations:** 1 Clinical Unit, Management Sciences for Health, Abuja, Nigeria; 2 Laboratory System Strengthening, Management Sciences for Health, Ilorin, Nigeria; 3 Nigerian Postal Service, Expedited Mail Service, Ilorin, Nigeria

## Abstract

**Introduction:**

WHO recommends that all children exposed to HIV be tested within four to six weeks of birth to ensure that all infected infants are identified and initiated on treatment early. One major challenge with early infant diagnosis (EID) of HIV in Nigeria remains the absence of standardized logistic sample transfer systems, resulting in long turnaround times between date of sample collection and date of return of result to the mother. To address this challenge, the USAID-funded ProACT project implemented by MSH, pioneered the Strengthening the Process and Efficiencies for Early infant Diagnosis (*SPEEiD*) model, which involves the transportation of dried blood spot (DBS) samples from remote HIV clinics to regional PCR labs using the Nigerian Postal Service (NIPOST) Express Mail Service (EMS) platform, which has a network of over 3900 post offices and agencies spread across the country, ensuring penetrance to remote HIV clinics. The objective of this study was to review the effect of utilizing an innovative DBS transport model in improving DBS transportation.

**Methods:**

We carried out a retrospective analysis of logistic data from 177 samples transferred from 28 PMTCT sites using the *SPEEiD* model over a 12 month period from March 2013 to February 2014 in Kwara state, North Central Nigeria.

**Results:**

A review of the data showed a reduction in turnaround time for return of results from 3–6 months to 3–4 weeks utilizing the *SPEEiD* Model. Results were received for 97% of samples (171/177) transported with this model, compared to 51% previously. The average cost of sample transfer was estimated at between $20 and $40 per batch and remains comparatively less expensive to other models by at least 30%.

**Conclusions:**

The MSH *SPEEiD* model remains an indigenous, cost effective, sustainable and time-sensitive sample transfer model which ensures that exposed infants are able to receive their EID test results quickly. This approach may be easily replicated by other partners working in similar resource limited settings, as it provides a practical solution for DBS sample transfer, which remains one of the major challenges affecting EID of HIV in Nigeria.

# Sub article: Trends in early infant HIV diagnosis and treatment services in rural southwest Uganda (2011–2014)

**DOI:** 10.7448/IAS.18.5.20565

**Published:** 2015-07-22

**Authors:** Juan Seclen-Palacin, Eliab Natumanya, Linda Nabitaka, Edward Bitarakwate, Shabbir Ismail

**Affiliations:** 1 Elizabeth Glaser Pediatrics AIDS Foundation, Washington, United States; 2 Elizabeth Glaser Pediatrics AIDS Foundation Uganda, Mbarara, Uganda; 3 Ministry of Health of Uganda, AIDS Control Program, Kampala, Uganda

## Abstract

**Introduction:**

In Uganda, 39% of HIV-exposed infants (HEI) were HIV tested within two months and <30% of children accessed to ARVs (UNAIDS, 2014), which reveal challenges to reach to early infant HIV diagnosis and treatment services (EIDT) services. Through implementation of Strengthening TB and HIV/AIDS response in Uganda Southwestern Region (STAR-SW) project, Elizabeth Glaser Pediatrics AIDS Foundation (EGPAF) provides support to districts and sites to strengthen and increase access to EIDT services. This includes training and mentoring site-based healthcare workers (nurses, clinicians) on proper utilization of EID guidelines (counselling and testing manuals, treatment protocols), optimizing patient care flow, expanding points of care, strengthening laboratory capacity, utilization of EIDT clinical registers and reporting and conducting regular data reviews for continuous improvement. This report describes trends of accessing EIDT services under this project.

**Methods:**

Using HIV programme data from the Uganda Health System for January 2011 to June 2014, we conducted an EIDT analysis covering all 192 supported sites. Indicators analyzed were number of HIV-positive pregnant women identified during antenatal care, HIV-positive mothers delivered at health institutions, HEI received ARV at birth, exposed infants tested for HIV within two months after birth. ARV uptake and HIV testing coverage were estimated by dividing number of HEI-received ARVs at birth and who were tested within two months between HIV-positive pregnant women in antenatal care (ANC), respectively. Descriptive and trends analysis were conducted.

**Results:**

By January 2011, HEI testing coverage was 17.8%, which increased to 47.5% in December 2012. With the rollout of the Option B+ in early 2013, HEI testing continued to increase and reached around 63% in mid-2014 (trend R^2^=0.8174). Simultaneously, HEI receiving ARVs at maternity progressively increased over time from 17% (312/1799) in January 2011 to 32% at the end of 2012, peaking at 48% (966/2026) in June 2014.

**Conclusions:**

HIV testing and ARV uptake for HEI have progressively improved in STAR-SW catchment area. The various site level of support provided by EGPAF seems to have contributed to these results. EGPAF will continue supporting the national and district health systems in further expansion of EIDT services as well as on further analysis of disaggregated data, informing quality improvement interventions and planning additional operational research studies.

**Abstract WEPDD0105–Figure 1:** Trends of EID for HIV in southwestern Uganda: 2011–2014.

# Sub article: Sensitivity of a rapid point of care assay for early HIV antibody detection is enhanced by its affinity for HIV gp41 IgM antibodies

**DOI:** 10.7448/IAS.18.5.20563

**Published:** 2015-07-22

**Authors:** Noushin Moshgabadi, Rick Galli, Amelia Daly, Sze Mun Shirely Ko, Morgan Zhang, Tayla Westgard, Ashley Bulpitt, Christopher R Shackleton

**Affiliations:** Biolytical Laboratories, Richmond, Canada

## Abstract

**Introduction:**

HIV-IgM antibody is detectable within two weeks following infection and is therefore an important immunoassay target for early HIV antibody detection. The objective of this study is to determine if the proven early HIV antibody sensitivity of the 60-second INSTI HIV-1/HIV-2 antibody test is due to its ability to detect HIV-IgM antibodies.

**Methods:**

The INSTI HIV-1 gp41 recombinant antigen was applied to a HIV-IgM ELISA to demonstrate its ability to capture HIV gp41 IgM antibody. This HIV-IgM ELISA assay was run on six commercial early seroconversion samples, known to be HIV-IgM positive, and five long-term HIV-positive serum samples. A separate experiment to demonstrate that the dye-labelled recombinant Protein A-based colour developer (CD) used in the INSTI assay has affinity to human IgM was conducted. A quantity of 0.5 µg of purified human immunoglobulins (IgM, IgD, IgA, IgE and IgG) were blotted onto nitrocellulose (NC) and probed with the CD to observe for spot development. Finally, to determine if INSTI performance is affected by IgM removal, IgM was removed by human anti-IgM MicroBeads on 21 early seroconversion samples with known or undetermined levels of HIV-IgM and with five samples from long-term HIV-positive samples. INSTI results were observed for reduced test spot intensity following IgM removal.

**Results:**

The gp41-based HIV-IgM ELISA was positive for the six early seroconversion samples that were known INSTI and HIV-IgM positive, and negative for the five long-term HIV-positive samples indicating the assay signal was due to HIV-IgM capture by the immobilized gp41 antigen. The dye-labelled recombinant Protein-A used in the INSTI colour developer produced distinct spots for purified IgM, IgA and IgG blotted on the NC membrane. Following IgM removal from 21 seroconversion samples with known or undetermined HIV-IgM levels, 10/21 samples became INSTI HIV negative from INSTI HIV positive. In 10/21 samples test, spot intensity was reduced by >50% and 1/21 samples slightly &lt;50%, while the five long-term HIV-positive samples showed no reduction (Table 1).

**Conclusions:**

The INSTI HIV-1/HIV-2 Antibody Test is shown to detect HIV gp41-specific IgM antibodies in early HIV infection, which enhances its utility in early HIV infection.

**Abstract WEPDD0106–Table 1:** Summary of INSTI^™^ test results on Commercial HIV-1 Seroconversion Samples before and after IgM removal

	Before IgM removal	After IgM removal			
Number of seroconversion (SC) samples					
	INSTI positive	INSTI test dot became negative	INSTI test dot intensity diminished by >50%	INSTI test dot intensity diminished by <50%	No change in INSTI test dot intensity
15 early SC, IgM positives	15	8	7	–	–
6 early SC, IgM not determined	6	2	3	1	–
5 late SC, IgG	5	–	–	–	5
Total=26	26	10	10	1	5

# Sub article: AUTHOR INDEX

**Published:** 2015-07-22

## A

**Table:** 

Abdel-Mohsen, M	TUAA0204LB
Abdool Karim, S	TUAC0101LB
Abimiku, A	MOAA0205
Abrahams, F	WEAA0102
Abram, M	TUAB0103
Abrams, EJ	MOAB0107LB
Achii, P	WEAD0203
Achu, MN	TUAD0205
Adams, R	MOAA0101
Adler, M	TUAD0204
Agot, K	MOAC0302LB, TUAC0102
Agyei, Y	TUAC0106LB
Ahlers, J	MOAA0104
Ahmed, S	WEPDD0102
Ajibola, G	MOPDB0104*
Akapirat, S	WEAB0102
Akello, I	MOAC0302LB
Akelo, V	MOAC0106LB
Alave, J	MOAB0205LB
Alcaide, M	MOPDB0101*
Alcami, J	MOPDA0103*
Alimenti, A	WEPDC0101, WEPDC0102
Allegre, T	TUAB0207LB
Allison, S	TUAC0106LB
Altice, F	MOPDD0101
Amaros, I	WEPDD0103
Ambinder, RF	MOPDA0105
Amedee, A	WEAA0106LB
Amico, KR	MOAC0305LB, MOAC0306LB
Amzal, B	MOAC0204
Ananworanich, J	WEAB0102, WEAB0104, WEAD0204, MOAB0105
Anastos, K	MOAA0203
Anato, S	TUAC0304
Ancuta, P	WEAA0105
Anderson, P	TUAC0202
Anderson, PL	MOAC0306LB
Andrade, J	TUAB0102
Andrew, K	WEPDB0104
Andrew, P	TUAC0106LB
Angin, M	MOAA0105LB
Ansari, A	MOAA0202
Anstett, K	TUAA0104
Antonini-Michelle, T	TUAB0207LB
Apornpong, T	TUAB0101
Apps, R	TUAA0106LB
Arasteh, K	TUAB0104
Archin, N	MOPDA0106LB
Arendt, M	TUPDB0101
Argento, E	WEAC0101*
Arheart, K	MOPDB0101
Arredondo, J	MOPDD0103
Arribas, J	TUAB0103
Arthos, J	MOAA0202
Arts, E	TUAA0102
Asamoah-Odei, E	MOAD0101
Ásbjörnsdóttir, KH	MOAB0103*
Aschman, N	TUPDA0103
Asila, V	TUPDD0105
Asire, B	WEAD0203
Asmuth, D	MOAA0204
Aumaitre, H	TUAB0207LB
Aurpibul, L	TUPDB0104
Auvert, B	MOAC0102*
Avettand-Fenoel, V	MOAA0105LB
Avihingsanon, A	TUAB0101
Avila-Rios, S	MOPDA0102
Ávila-Ríos, S	WEAA0103
Awa, JC	TUAD0205

## B

**Table:** 

Bacchetti, P	MOAA0106LB
Bachireddy, C	MOPDD0101*
Bacon, O	TUAC0202
Badal-Faesen, S	MOAB0205LB, MOAC0101LB
Baddeley, A	MOAB0204*
Baeten, J	MOAC0105LB
Baeten, JM	MOPDB0103
Bai, F	TUPDA0101
Bailer, R	TUAA0106LB
Bajos, N	WEPDC0103
Bakir Timimi, H	MOAB0204
Bamford, A	WEAD0204
Banchongkit, S	TUAB0101
Bandason, T	WEAD0202*
Bangsberg, DR	TUAA0103
Bañon, S	TUAB0204
Bao, J	MOAB0205LB
Baral, S	WEAC0106LB, TUAC0304
Barash, E	WEPDC0104
Barbara, C	WEPDB0104
Barbee, L	WEPDC0104
Barnabas, R	MOAC0105LB*
Bärnighausen, T	TUAC0105
Barrios, R	WEAD0301, MOAC0103
Bartali, B	TUPDB0103
Basilissi, M	TUPDA0101
Batisse, D	TUAB0207LB
Batlang, O	MOPDB0104
Bautista-Arredondo, S	TUAC0104, MOAC0205LB
Bavinton, BR	TUAC0306*
Baxter, C	TUAC0101LB
Bayus, B	TUAD0104
Bego, MG	TUPDA0103
Behets, F	TUAD0202
Bekker, L-G	WEAD0101, MOAB0104
Belaunzarán-Zamudio, P	WEAD0104
Bélec, L	WEPDB0101
Beletsky, L	MOPDD0103*
Bellaton, E	MOAA0105LB
Benki-Nugent, S	MOAB0103
Benkirane, M	MOPDA0103
Bennai, Y	TUAB0207LB
Benne, C	MOAA0102
Bennett, A	TUAC0303, WEPDC0104
Bennett, K	MOPDB0104
Bennett, S	MOAB0104
Benson, P	TUAB0103
Berhanu, R	MOAB0202
Berkout, B	TUPDA0104
Bermejo, M	MOPDA0103
Bernardi, G	MOPDA0104
Bernardi, S	WEAD0204
Bershteyn, A	TUAD0101*
Best, J	TUAD0104
Betz, B	TUPDD0105
Beusenberg, M	MOAD0101
Bhakeecheep, S	TUAB0101
Bhardwaj, N	TUPDA0102
Bhatti, L	TUAB0203
Bianchi, F	TUAD0205
Billaud, E	TUAB0207LB
Bingham, B	WEAC0101
Bini, T	TUPDA0101
Bisson, G	MOAB0205LB
Bitarakwate, E	WEPDD0105
Bitimwine, H	WEAD0203
Bitnun, A	WEPDC0101, WEPDC0102
Blanchard, JF	TUPDC0102
Blankson, J	MOAA0101
Blaser, N	WEAD0304
Blick, G	TUAB0203
Bloch, M	TUAB0103, TUAB0206LB
Bloomenthal, D	MOPDB0102
Blosser, SJ	MOAC0304LB
Bock, N	MOAC0301LB
Boffito, M	TUAB0106LB
Bolu, O	TUAD0205
Bonczkowski, P	WEAB0101
Bonje, E	TUAD0205
Bonora, S	TUPDA0101
Booth, R	TUAC0402*
Boothe, M	TUAC0401
Borkowf, C	TUAC0203
Botha, G	MOAA0205
Boulle, A	TUAD0102
Bouscaillou, J	MOAC0102
Boven, K	MOAB0106*
Bowen, N	TUAD0103
Bowonwatanuwong, C	TUAB0101
Brantley, J	MOAC0105LB
Brewster, J	TUAC0402
Brockmeyer, NH	TUPDB0101
Brodie, E	MOAA0205
Brooks, J	TUPDC0102
Brophy, J	WEPDC0101*, WEPDC0102
Brothers, J	TUAC0204LB
Brown, T	TUPDB0103
Bruce, D	MOPDD0104
Brum, T	MOAC0101LB
Brumme, C	TUAA0101
Brumme, ZL	MOPDA0101, TUAA0103
Bruneau, J	TUAC0406LB
Brunet, L	TUAB0201*
Brunetta, J	TUAB0102
Bruno, D	MOAC0106LB
Bruyand, M	MOAB0203
Buchbinder, S	MOPDC0104, TUAA0103, TUAC0202
Budgell, E	MOAB0202*
Bui, DD	TUAC0404
Bukusi, EA	TUPDD0102
Bullock, B	MOAA0101
Bulpitt, A	WEPDD0106
Bunupuradah, T	MOAB0106, TUAB0101*, TUPDB0104
Burbelo, P	MOAA0103
Burger, H	MOAA0203
Burling, K	TUPDB0102
Burns, D	MOAC0101LB
Burwitz, BJ	TUPDA0106LB
Busch, M	WEPDA0103
Buskin, S	TUAC0303, WEPDC0104
Buskin, SE	TUAD0105LB
Bustorff, F	MOAC0106LB
Butcher, J	MOAA0205
Buzdugan, R	MOAC0203, MOAC0205LB
Byrareddy, S	MOAA0202*

## C

**Table:** 

Caballero, P	WEAD0102*
Cahn, P	WEAD0104
Caillat-Zucman, S	MOAA0105LB
Calcagno, A	TUPDA0101
Calderon, F	WEAD0105LB
Calmy, A	MOAC0104
Cameron, B	MOAA0205
Cameron, M	MOAA0104
Cameron, P	MOAA0106LB
Camlin, CS	TUPDD0102*
Cannizzo, ES	TUPDA0101
Capoferri, A	MOPDA0105*
Carlson, JM	TUAA0103
Carmona, S	WEPDB0105
Caron, F	WEAB0103
Carrington, M	TUAA0103
Casado, JL	TUAB0204
Cash, A	MOPDA0105
Casillas, P	TUAC0203
Castillo, LS	MOPDC0104
Cathcart, R	TUAD0206LB
Cavassini, M	TUAB0102
Caylà, JA	MOAB0203
Ceesay, N	TUAC0304
Celentano, D	MOAC0101LB
Celum, C	MOAC0105LB, MOPDB0103
Cesar, C	WEAD0104
Cesar, F	TUPDD0104
Chadwick, EG	MOAB0102
Chadwick, K	WEAC0104
Chaisson, R	MOAB0201
Chaivooth, S	TUAB0101
Chalachala, JL	TUAD0202
Chamie, G	TUPDD0102
Chan, G	TUAC0303*
Chang, C	MOAA0106LB*
Chang, L	WEPDC0105
Changalucha, J	MOAC0301LB
Chapman, EL	MOAC0304LB
Chariyalertsak, S	MOAC0101LB, TUAC0201
Chas, J	TUAB0207LB
Chauma, A	MOAC0202
Chege, W	TUAC0202
Chen, X	TUAC0301
Chen, Y	MOAC0101LB
Chen, Y	WEPDA0103
Chen, YQ	MOAC0106LB
Cheng, A	TUAB0103
Chetchotisakd, P	TUAB0101
Chew, G	TUAA0204LB*
Chew, GM	TUPDA0106LB
Chhabra, R	TUPDD0106
Chikovani, I	TUPDC0104
Chilisa, F	MOPDB0104
Chimbwandira, F	MOAC0201
Chioma, S	MOAA0101
Chirwa, L	TUAC0203
Chisembele, M	MOPDB0101
Chitwarakorn, A	MOAC0306LB
Chitwarakorn, A	TUAC0302
Chohan, BH	MOAB0103
Chokephaibulkit, K	MOAB0104, MOAB0105
Chomont, N	MOAA0102, MOAA0103
Chonzi, P	WEAD0202
Chotirosniramit, N	MOAC0106LB
Christilaw, J	MOPDB0102
Chuma, B	WEAD0305*
Cicala, C	MOAA0202
Citao, SC	TUPDD0104
Clayton, K	TUAA0204LB
Clements, J	MOAA0101
Cléret-Buhot, A	WEAA0105
Co, M	TUAB0203
Cohen ÉA	TUPDA0103*
Cohen, C	TUAB0102
Cohen, CR	TUPDD0102
Cohen, M	MOAC0101LB*
Cohen, MS	MOAC0106LB
Cohen, S	TUAC0202
Coiras, M	MOPDA0103
Colby, D	WEAB0102, WEAB0104*
Colley, G	TUAA0101
Collier, A	TUPDB0103
Conrad, C	MOAC0303LB, MOAC0304LB
Cooper, C	TUAB0201, TUAB0202*
Cooper, DA	TUAB0101
Cornell, M	WEPDB0103
Cortés, C	WEAD0104
Cortes, CP	MOAD0103
Cortés-Álvarez, J	WEAA0103
Côté, ÉA	TUPDA0103
Cottle, L	MOAC0101LB
Cotton, LA	TUAA0103
Cousien, A	TUAC0406LB*
Cowan, F	MOPDC0105
Cowan, FM	MOAC0203*, MOAC0205LB*
Cox, J	TUAC0406LB
Cragg, C	MOAD0105LB
Crauwels, H	MOAB0106
Cressey, T	MOAC0204
Crowell, CS	MOAB0102*
Crowell, T	WEAB0104
Cryer, C	MOAA0101
Curlin, ME	MOAC0306LB
Custodio, J	TUAB0103
Cutler, E	MOAC0102
Czaicki, N	TUAC0104*

## D

**Table:** 

Da Cruz, M	WEAD0102
Da Fonseca, S	WEAA0105
Da Silva, CM	WEAD0102
Dabis, F	WEAD0103, MOAC0104
Dabis, F	TUAB0207LB
Dai, J	TUAC0206LB
Daly, A	WEPDD0106
Dange, A	TUPDD0106
Daniell, L	WEPDA0103
Dantanarayana, A	MOAA0106LB
d'Arminio Monforte, A	TUPDA0101
Das, AT	TUPDA0104
Das, B	TUAA0205LB
Dasgupta, A	WEAD0105LB
Dave Sen, P	TUAD0203
Davis, J	TUAC0402
De Boni, RB	WEAD0104, TUAC0205LB
De Cock, K	TUAD0103
De Ledinghen, V	TUAB0207LB
de Looze, D	WEAB0101
De Maeyer, M	TUAA0105
De Paris, K	WEAA0106LB*
De Rijck, J	TUAA0105
De Rosa, S	MOAB0103
de Sousa Marques, HH	WEAD0204
de Souza, M	WEAB0102
De Spiegelaere, W	WEAB0101*
De Truchis, P	TUAB0207LB
Debab, Y	WEAB0103
Debyser, Z	TUAA0105
Decamp, A	TUAA0106LB
Deeks, S	MOAA0103, MOAA0104, TUAA0204LB, TUAA0205LB
Deeks, S	MOAA0106LB
DeJesus, E	TUAB0102
dela Pena-Ponce, MG	WEAA0106LB
Della Negra, M	WEAD0204
Demarco, T	WEPDA0103*
Demeulemeester, J	TUAA0105
Demicco, E	WEAD0105LB
Denny, T	WEPDA0103
Derksen, L	MOPDC0102*
Deschamps, D	MOAA0105LB
Descours, B	MOPDA0103
Desgrees du Lou, A	WEPDC0103
deSilva, T	TUPDA0105
Dezutti, C	TUAC0206LB
Dhiver, C	TUAB0207LB
Di Biagio, A	WEAD0204
Dibb, Z	WEAC0104
Dicker, I	TUAB0106LB
Dieterich, D	TUAB0202
Dikeakos, J	WEPDA0104
Dinh, TTT	TUAC0404
Diouf, D	TUAC0304
DiPerri, G	TUAB0102
Dirawo, J	MOAC0203
Ditangco, R	TUPDC0103
Djurovic, I	WEAD0303
Do, TN	TUAC0404
Do, VC	MOAB0105
Doblecki-Lewis, S	TUAC0202
Dobrer, S	TUAC0103
Dobrowolski, C	TUAA0205LB
Doherty, M	MOAB0204, MOAD0101*
Dombrowski, J	TUAC0303, WEPDC0104
Dombrowski, JC	TUAC0305, TUAD0105LB*
Domercant, JW	TUAD0204*
Dominguez, S	TUAB0207LB
Dong, W	MOPDA0101
Donoghoe, M	MOAD0101
Dorfman, J	WEAA0102*
Dray-Spira, R	WEAD0103, MOAC0104, WEPDC0103
Duff, P	WEAC0101
Dukhovlinova, E	MOAC0106LB
Duncan, D	TUAD0204, TUAD0205
Dunne, E	TUAC0302
Dunning, L	MOAB0101
Durand, CM	MOPDA0105
Duvivier, C	TUAB0207LB
Duwve, J	MOAC0303LB*
Duwve, JM	MOAC0304LB
Dvoryak, S	MOPDD0101
Dvoryak, S	TUAC0402
Dye, BJ	MOAC0305LB, MOAC0306LB
Dziuban, E	WEAD0203

## E

**Table:** 

Eckhoff, P	TUAD0101
Edmonds, A	TUAD0202
Egger, M	WEAD0304, MOAC0201, WEPDB0103
Ehrenberg, P	TUAA0106LB
Eisele, L	TUPDB0101
El Ayadi, A	TUPDD0101
El Farouki, K	WEAD0103
Elharrar, V	MOAC0101LB, MOAC0305LB, MOAC0306LB
Elion, R	TUAB0102, TUAC0202
Elliott, J	MOAA0106LB, TUAD0102
Elston, B	TUAC0103
Elyanu, P	WEAD0203
Emery, S	TUAB0101
Emmanuel, F	TUPDC0102
Engelsmann, B	MOAC0203
Engle, E	MOAA0101
Erbel, R	TUPDB0101
Erlandson, K	TUPDB0103
Eron, J	MOAC0101LB
Eron, JJ	TUAB0203
Escamilla-Gómez, T	WEAA0103
Eshleman, S	MOAC0101LB, TUAC0106LB
Eshleman, SH	MOAC0106LB*, MOAC0306LB
Esser, S	TUPDB0101*
Essex, M	MOAC0101LB
Estaquier, J	WEPDA0106LB
Estes, J	WEAA0106LB
Estill, J	WEAD0304
Etard, J-F	WEPDD0103
Etsetowaghan, A	WEPDD0104
Evans, DH	MOAB0202
Evans, E	MOPDD0102
Evans, T	MOAA0206LB

## F

**Table:** 

Fang, E	MOPDC0106
Farquhar, C	MOPDC0103, TUPDD0105
Fauci, A	MOAA0202
Faye, A	MOAA0105LB
Fedchuk, L	TUAB0207LB
Feldacker, C	TUPDD0104
Fennelly, G	WEAA0106LB
Fenton, K	TUAD0104
Ferguson, M	WEAC0102
Fernandes, NM	TUAC0205LB
Fernandez-Lopez, JC	MOPDA0102
Ferrand, R	WEAD0202
Ferrao, C	TUPDD0104
Ferrari, G	TUAA0106LB
Filipovics, A	TUAB0207LB
Fischl, M	MOPDB0101
Fisher, H	TUAC0401
Fleming, M	TUAD0105LB
Fleming, T	MOAC0101LB
Fletcher, JLK	WEAB0102*
Flick, R	WEPDD0102
Flynn, P	MOAB0106
Fofana, DB	MOAB0102
Fon, H	MOPDC0106
Fong, Y	TUAA0106LB
Fontaine, H	TUAB0207LB
Foote, J	MOAA0101
Ford, N	WEAD0304, MOAD0102
Fordyce, M	TUAB0103
Foshee, V	TUPDC0105
Fourati, S	MOAA0104
Fourie, J	MOAB0104
Fox, J	TUPDB0102
Fox, MP	WEPDB0103
Frahm, N	WEPDA0102
Francois, K	TUAD0204
Frange, P	MOAA0105LB
Franks, J	MOAC0305LB
Friedman, RK	TUAC0306
Friedman, SR	TUPDC0101
Frohlich, J	TUAC0101LB
Fromentin, R	MOAA0102
Fromentin, R	MOAA0103
Fu, G	TUAC0301
Fu, J	TUAC0301
Fuchs, J	TUAA0103
Fujita, T	TUAA0204LB, TUPDA0106LB
Furrer, H	MOAB0203

## G

**Table:** 

Gabriel, C	WEPDD0104
Gadabu, O	MOAC0201
Galang, R	MOAC0303LB
Galang, RR	MOAC0304LB*
Galaska, B	TUAC0206LB
Gallant, J	MOAC0101LB
Galler, S	TUAD0104
Galli, R	WEPDD0106
Gama, L	MOAA0101
Gamble, T	MOAC0101LB, MOAC0106LB
Gandhi, M	WEAD0105LB, TUAA0205LB
Ganova-Raeva, L	MOAC0304LB
Gao, F	WEPDA0103
Gao, Y	TUAA0102
García-Morales, C	MOPDA0102, WEAA0103
García-Tellez, T	MOPDA0102
García-Téllez, T	WEAA0103
Garrido-Rodriguez, D	MOPDA0102*
Garrido-Rodríguez, D	WEAA0103
Gaseitsiwe, S	WEPDA0105
Gatell, J	TUAB0104*
Gathe, J	TUAB0103
Gatome-Munyua, A	WEAD0305
Gaur, A	MOAB0104*
Geisel, MHM	TUPDB0101
Geng, E	WEAD0105LB
Geng, W	TUAC0301
Gentry, J	MOAC0304LB
George, M	MOAA0203*
Geraghty, D	TUAA0106LB
Getahun, H	MOAB0204
Ghannoum, M	MOAA0104
Ghidinelli, M	MOAD0101
Ghneim, K	MOAA0104*
Giacomet, V	WEAD0204
Giaquinto, C	WEAD0204
Gibbons, K	TUAB0203
Giganti, M	MOAD0103
Gijsbers, R	TUAA0105*
Girardi, E	MOAB0203
Go, VF	WEAC0104
Godbole, S	MOAC0101LB
Golden, M	MOPDC0103, MOPDC0106, TUAC0303, TUPDD0104*, WEPDC0104
Golden, MR	TUAC0305, TUAD0105LB
Golub, J	MOAB0201
Gomez-Olivé, X	TUAC0106LB
Gone, M	TUPDD0105
Gonzales, L	WEPDB0105
Gonzalez-Zuniga, P	MOPDD0103
Gosselin, A	WEAA0105
Gosselin, A	WEPDC0103
Gotsadze, G	TUPDC0104
Goulder, PJR	WEPDA0102
Goulet, J-P	WEAA0105
Gouveia, ML	TUAC0401
Grabowski, MK	WEPDC0105*
Grand'Pierre, R	TUAD0204
Grant, P	TUPDB0103*
Grant, RM	MOAC0305LB, MOAC0306LB, TUAC0201*
Grasela, D	TUAB0106LB
Grasso, M	WEAC0103
Grau Pujol, B	WEAA0102
Gray, C	MOAA0205
Gray, GE	WEPDA0102
Gray, R	WEPDC0105
Gregory, P	TUAA0202
Gress, J	TUAB0206LB
Grigoryan, S	WEAD0302
Grigoryan, T	WEAD0302
Grimsrud, A	MOAD0105LB, WEAD0101*, WEPDB0103*
Grinsztejn, B	MOAC0101LB, MOAC0106LB, TUAC0205LB*, TUAC0306
Grosso, A	TUAC0304*
Grulich, AE	TUAC0306
Grund, J	MOAC0301LB
Guanira, JV	TUAC0201
Gueguen, M	WEPDD0103
Guillemi, S	MOPDD0105, TUAC0405
Gunther, N	WEPDB0102
Gupta, A	MOAB0205LB
Gupta, S	TUAB0103*

## H

**Table:** 

Ha, B	TUPDB0103
Haas, A	WEAD0304
Haas, AD	MOAC0201*
Haber, N	TUAC0105*
Habiyambere, V	WEAD0304
Haeryfar, M	WEPDA0104
Hageman, K	TUAC0203
Haghdoost, A	WEAC0105
Hagins, D	TUAB0104
Hakim, J	MOAB0205LB, MOAC0101LB
Hakobyan, A	MOAC0203, TUAD0203
Halpern, CT	TUPDC0105
Hamilton, E	TUAC0106LB
Hammond, KB	TUPDA0106LB
Hamzah, L	TUPDB0102*
Han, L	TUAD0104
Han, X	TUAC0301
Han, Y	TUAA0104
Hance, RJ	TUAC0201
Hanrahan, C	MOAB0201*
Hans, L	WEPDB0105
Hansen, SG	TUPDA0106LB
Harawa, M	WEPDD0102
Harent, S	TUAB0207LB
Harley, B	MOAD0105LB
Harrigan, PR	MOPDA0101, TUAA0101, TUAA0103
Hartogensis, W	WEAD0105LB, MOAA0106LB
Harty, L	MOPDA0104
Hassounah, S	TUAA0104
Hatano, H	WEAD0105LB
Hatzakis, A	TUPDC0101
Hatzold, K	MOPDC0105
Havlir, D	WEAD0105LB, MOAC0101LB, TUPDD0102
Hayashi, K	MOPDA0101
Hayes, R	MOAC0301LB
Headley, J	WEAC0102
Hecht, F	MOAA0106LB, TUAA0204LB
Heffron, R	MOPDB0103
Henderson, F	TUAC0203*
Hendrix, C	MOAC0305LB, MOAC0306LB, TUAC0206LB
Heneine, W	MOAC0304LB
Henostroza, G	MOAB0205LB
Herbeck, J	WEPDC0104
Herbst, K	TUAC0105
Hermanus, T	WEPDA0101
Hernández-Juan, R	WEAA0103
Hill, A	MOAD0102
Hillman, D	MOAC0303LB, MOAC0304LB
Hinkle, J	MOAA0204
Hirnschall, G	MOAD0101
Hirsch-Moverman, Y	MOAC0305LB
Hislop, M	TUAD0102
Hoagland, B	TUAC0205LB
Hoces, D	WEAD0104
Hoffman, I	MOAC0101LB, MOAC0202
Hoffman, IF	WEAC0104
Hoffmann, C	TUAB0104
Hogg, B	TUAC0405
Hogg, E	MOAB0205LB
Hogg, R	MOPDD0105, TUAA0101
Hoh, R	MOAA0106LB
Holland, C	WEAC0106LB
Holmes, L	MOPDB0104
Holmes, M	TUAA0202
Holtz, T	TUAC0302*
Holtz, TH	MOAC0306LB*
Holzendorf, V	TUPDB0101
Honermann, B	WEAD0306LB*
Hoogstoel, A	MOAB0106
Hoots, B	MOAC0303LB
Hoover, K	MOAC0303LB
Hora, B	WEPDA0103
Hornsberger, A	MOPDB0102
Horth, R	TUAC0401
Hosek, S	TUAC0204LB*
Hosseinipour, M	WEAC0104, MOAB0205LB*, MOAC0101LB
Hosseinipour, MC	MOAC0202
Hovhannisyan, R	WEAD0302
Hower, M	TUPDB0101
Hsiao, M	MOAB0101
Hsiao, N-Y	WEPDB0105
Hu, Q	TUAC0301
Hu, S-L	TUAA0202
Hu, W	TUAA0203
Hu, YB	TUAB0203
Huang, S	TUAD0104
Hudelson, SE	MOAC0106LB
Hudgens, M	TUAD0104, WEAA0106LB
Hughes, J	MOAC0105LB, MOAC0305LB, MOAC0306LB, MOPDC0103, TUAC0106LB
Hughes, JP	MOAB0103, MOAC0106LB, TUAD0105LB
Hull, M	TUAB0201
Humphries, H	TUAC0101LB
Hunt, P	MOAA0204
Hurst, J	WEPDA0102
Hwang, C	TUAB0106LB*

## I

**Table:** 

Iakoubov, R	TUPDB0105
Iannuzzi, F	TUPDA0101
Ibrahim, J	WEPDD0104
Inwani, I	WEAD0201
Isaac, L	WEPDB0104
Isdahl, Z	WEAC0103
Ishii, N	TUAA0204LB
Ismail, S	WEPDD0105
Ivan, K	WEPDB0104
Iveson, H	TUPDB0102
Iwuji, C	WEAD0103, MOAC0104*
Izenberg, J	MOPDD0101

## J

**Table:** 

Jacob, R	WEPDA0104
Jacob, RA	WEAA0102
Jacobs, S	MOAD0105LB
Jacobs, W	WEAA0106LB
Jacobson, J	MOPDA0104
Jaggernath, M	WEPDA0101
Jahn, A	MOAC0201
Jao, J	MOAB0107LB*
Jarman, K	MOPDA0104
Jarupanich, T	MOAC0204
Jaspan, H	MOAA0205*
Jean, K	MOAC0102
Jenabian, M-A	WEAA0105
Jenkins, L	MOAC0103*
Jennings, K	MOAD0105LB
Jensen, K	WEAA0106LB
Jere, E	MOAC0202
Jia, H	MOAC0304LB
Jiamsakul, A	TUPDC0103
Jiang, Y	TUAC0301
Jin, F	TUAC0306
Jirajariyavej, S	TUAB0101
Jöckel, K-H	TUPDB0101
John, C	TUPDB0102
Johns, B	WEAD0305
Johnson, A	WEPDA0104*
Johnson, M	WEAB0101
John-Stewart, G	WEAD0201, WEAD0205LB
John-Stewart, GC	MOAB0103
Johnston, R	TUAA0205LB
Jones, D	WEAD0105LB
Jones, D	MOPDB0101
Jones, L	MOAB0205LB
Joseph, B	TUAC0403*
Joseph, P	MOAC0105LB
Jourdain, G	MOAC0204
Joy, J	TUAA0101*
Julien, A	TUAC0106LB
Justman, JE	TUAC0206LB*

## K

**Table:** 

Kahn, K	MOPDC0101, TUAC0106LB, TUPDD0101
Kaimal, A	WEPDB0104
Kajaste-Rudnitski, A	TUPDA0104
Kajula, L	TUPDC0105
Kakkar, F	TUAB0105*, WEPDC0101, WEPDC0102
Kallas, EG	TUAC0205LB
Kalombo, C	WEAD0101
Kamarulzaman, A	TUPDC0103
Kambli, H	TUPDD0106
Kaminski, R	TUAA0203
Kamya, MR	TUPDD0102
Kanchar, A	MOAB0204
Kandulu, JR	WEPDD0103
Kang Dufour, M-S	MOAC0203
Kanjanavikai, P	MOAC0204
Kantipong, P	TUAB0101, TUPDC0103
Kantor, R	TUPDC0103
Kapiga, S	MOAC0301LB
Kapogiannis, B	TUAC0204LB
Karambe, S	TUPDD0106
Karamouzian, M	WEAC0105
Karaoz, U	MOAA0205
Karim, QA	TUAC0101LB*
Karn, J	TUAA0203, TUAA0205LB*
Kasonde, M	TUAC0203
Katana, A	WEAD0205LB
Katayi, E	TUAD0205
Katlama, C	TUAB0206LB
Kato, M	TUAC0404*
Katureebe, C	WEAD0203
Katz, D	MOPDC0103*, TUAD0105LB
Katz, DA	TUAC0305
Kawende, B	TUAD0202
Kawooya, V	TUAD0201
Keinonen, S	WEPDA0103
Keiser, O	WEAD0304*, MOAC0201
Kelly, S	WEAD0302*
Kerani, R	WEPDC0104*
Kern, KE	TUPDB0105
Kerr, C	WEAD0302
Kerr, SJ	MOAB0105, TUAB0101
Kerr, T	MOPDD0105, TUAC0103, TUAC0405
Khalili, K	TUAA0203*
Khan, A	WEPDB0105*
Kharsany, A	TUAC0101LB
Khosropour, CM	TUAC0305*
Khoury, G	MOPDA0104
Khudyakov, Y	MOAC0304LB
Kidzeru, E	MOAA0205
Kieffer, MP	TUAD0206LB*
Kiem, H-P	TUAA0202
Kiertiburanakul, S	TUAB0101
Kijak, G	TUAA0106LB
Kim, A	TUAD0103*
Kim, J	WEAB0104, TUAA0106LB
Kim, J	TUAD0104
Kim, JH	WEAB0102
Kim, M	WEPDD0102
King, DF	WEAA0101
Kinloch, NN	TUAA0103*
Kinloch-deLoes, S	WEAB0101*
Kinuthia, J	WEAD0205LB, TUPDD0105
Kirk, O	MOAB0203
Kirui, FK	MOAB0205LB
Kiselinova, M	WEAB0101
Kitch, D	TUPDB0103
Kizito, H	MOAB0104
Klein, D	TUAD0101
Klein, G	MOAA0204
Klein, K	TUAA0102*
Klein, MB	TUAB0201, TUAB0205
Klein, N	WEAD0204
Klinbuayaem, V	TUAB0101
Klopfer, S	TUAB0206LB
Koblin, B	TUAA0103
Koenig, E	TUAB0102
Koetsawang, S	MOAC0204
Kohler, P	MOAD0102
Kolber, M	TUAC0202
Koletar, S	TUPDB0103
Koné, N	MOAB0102
Korman, A	TUAA0204LB
Korman, AJ	TUPDA0106LB
Kosalaraksa, P	MOAB0105
Kosalaraksa, P	TUPDB0104
Kose, Z	WEAC0106LB
Koseki, S	WEAD0305
Koup, R	TUAA0106LB
Kourtis, AP	MOPDB0103
Kozlowski, P	WEAA0106LB
Krakowiak, D	TUPDD0105
Kranzer, K	WEAD0202
Krebs, E	WEAD0301*
Kriel, E	MOAD0105LB
Kroon, E	WEAB0102
Kroon, M	MOAB0101*
Krows, M	MOAC0105LB
Krystal, M	TUAB0106LB
Kublin, J	WEPDA0102
Kulasegaram, R	TUPDB0102
Kumarasamy, N	MOAB0205LB, MOAC0101LB, MOAC0106LB
Kumwenda, J	MOAB0205LB, MOAC0106LB
Kumwenda, K	MOAC0202
Kumwenda, N	MOAC0101LB
Kuringe, E	MOAC0301LB
Kurniati, N	MOAB0105
Kurniati, N	TUPDB0104
Kwarisiima, D	TUPDD0102
Ky-Zerbo, O	TUAC0304

## L

**Table:** 

Lackovic, K	MOPDA0104
Lacombe, K	TUAB0207LB
Ladner, J	WEAB0103
Lafeuillade, A	TUAB0207LB
Lagat, D	MOAB0205LB
Lalezari, J	TUAB0203, TUAB0206LB
Lallemant, M	MOAC0204*
Lama, JR	MOAB0205LB
Lamarre, V	TUAB0105
Lambdin, BH	MOPDD0104*
Lambert, A	WEAC0106LB
Lancaster, K	WEAC0104*
Landovitz, R	TUAC0204LB
Lane, T	TUAC0401, WEAC0103*
Langat, A	WEAD0205LB
Lapointe, N	TUAB0105, WEPDC0101, WEPDC0102
Larke, N	MOAC0301LB
Larmarange, J	WEAD0103, MOAC0104
Larsen, M	WEAA0106LB
Lataillade, M	TUAB0106LB
Latkin, C	TUAC0402
Laurence, J	WEPDB0104
Le, A	MOPDA0101*
Le, AKA	TUAC0404
Le, AQ	TUAA0103
Le, MG	TUAC0404
Le Chenadec, J	MOAA0105LB
Le Cœur, S	MOAC0204
Leask, K	TUAC0101LB
Leclerc, P	TUAC0406LB
Lederman, M	MOAA0104
Lederman, MM	MOPDA0103
Lee, M	MOPDA0104
Lee, S	MOAA0103*
Lee, S-K	MOPDA0106LB*
Lee, T	WEPDC0101, WEPDC0102
Leerattanapetch, N	TUAB0101
Leger, PD	MOAB0205LB
Leisegang, R	TUAD0102*
Leitman, EM	WEPDA0102*
Leligdowicz, A	TUPDA0105
Lem, E	TUAD0205
Lemée, V	WEAB0103
Leporrier, J	WEAB0103
Leroy, V	TUAB0207LB
Lert, F	WEPDC0103
Lesosky, M	WEAD0101
Lessler, J	WEPDC0105
Levi, J	MOAD0102*
Levis, M	MOPDA0105
Levy, J	WEAD0204
Levy, L	TUAC0206LB
Lewin, S	MOAA0106LB, MOPDA0104
Lewy, T	MOAA0203
Li, H	MOAA0206LB
Li, L	MOPDD0102
Li, M	MOAA0101
Li, P	TUPDC0103
Li, XD	MOAA0204
Liang, J	TUAA0104
Liang, R	TUAA0101
Liegler, T	TUAA0204LB
Lifson, J	WEAA0106LB, MOAA0102, MOAA0106LB
Lim, JR	TUAD0205
Lima, V	MOPDD0102
Lindsey, K	WEAD0306LB
Link-Barnes, G	MOAB0201
Lippman, SA	MOPDC0101*, MOPDC0104*, TUPDD0101
Lissouba, P	MOAC0102
Liu, A	TUAC0202*
Liu, AY	TUAC0201, TUAC0205LB
Liu, F	TUAD0104
Liu, L	MOAC0304LB
Liu, L	MOPDD0102
Liu, N	TUAC0204LB
Liu, Y	TUAB0102
Lo, Y-R	MOAD0101, TUAC0404
Lobritz, M	TUAA0102
Lockman, S	MOPDB0104
Lombaard, J	MOAB0106
Long, L	MOAB0202
Loquere, A	MOAC0305LB
Lorenzana, I	WEAA0103
Losso, MH	MOAB0203
Louzao, R	WEPDA0103
Lovchik, J	MOAC0304LB
Lowrance, D	TUAD0204
Lu, J	TUAC0301
Lu, L	TUAC0301
Lu, L	TUAD0204
Luechai, P	TUAC0302
Lukabwe, I	WEAD0203
Lule, F	MOAD0101
Lundgren, JD	MOAB0203
Lungu, T	WEAC0104
Luo, W	MOAC0304LB
Lyall, H	WEAD0204
Lydie, N	WEPDC0103
Lyons, C	MOAA0101

## M

**Table:** 

Ma, B	TUAD0104
Ma, ZM	MOAA0204
Maartens, G	TUAD0102
Mabuza, W	TUAC0106LB
Mabuza, X	TUAC0304
MacAllister, J	WEAD0306LB
Macmillan, DR	TUAA0103
MacPhail, C	MOPDC0101, TUAC0106LB, TUPDD0101
Madlala, P	TUAA0105
Madruga, JV	TUAC0205LB
Magimba, A	MOPDD0104
Magongo, E	WEAD0203*
Mahapatra, T	TUAC0301
Mahler, H	MOAC0301LB
Mahomva, A	MOAC0203, TUAD0203
Maiga, AI	MOAB0102
Mailiiard, RB	WEAA0104*
Makhema, J	MOAC0101LB, MOPDB0104
Makhubela, P	WEAC0103
Malatinkova, E	WEAB0101*
Maleche-Obimbo, E	WEAD0201
Maliwichi, M	MOAC0106LB
Malliori, M	TUPDC0101
Mallolas, J	TUAB0206LB
Malupande, E	MOPDB0101
Maman, S	MOAC0302LB, MOPDC0101, TUPDC0105, TUPDD0101
Manabe, Y	WEPDD0101
Manches, O	TUPDA0102
Mangenah, C	MOAC0205LB
Mankowski, J	MOAA0101
Mannheimer, S	MOAC0305LB*
Manno, EC	WEAD0204*
Mant, C	TUPDB0102
Manyuchi, A	WEAC0103
Manzanero, M	WEAA0103
Marcelin, A-G	MOAB0102
Marchetti, G	TUPDA0101
Marcus, C	TUAC0106LB
Marczynska, M	WEAD0204
Mardarescu, M	WEAD0204
Marelli, SS	TUPDA0104
Margolis, D	MOPDA0106LB
Mark, J	TUPDD0105*
Markowitz, M	TUAA0103
Marquez, L	WEAD0204
Marr, A	WEAC0103
Marshall, BDL	TUAC0103*
Martens, CA	MOAC0106LB
Martinson, N	MOAB0201
Marzinke, M	MOAC0305LB, TUAC0206LB
Masciotra, S	MOAC0304LB
Masiku, CW	WEPDD0103
Masters, S	MOAC0302LB, TUAC0102
Matengeni, A	MOPDC0102
Mateos, E	MOPDA0103
Matiko, E	MOPDD0104
Matlhaba, O	TUAC0203
Matlhagela, K	WEPDA0105
Matsheka, M	WEPDA0105
Matteelli, A	MOAB0204
Matthews, G	TUAB0206LB
Matthews, PC	WEPDA0102
Maurer, M	TUAA0204LB, TUPDA0106LB
Mave, V	MOAB0205LB
Mavengere, Y	MOPDC0105
Mayer, K	MOAC0101LB, TUAA0103
Mayer, KH	MOAC0106LB, TUAC0201
Mboh, E	MOPDC0106, TUAD0205
M'Boyis Kamdem, H	WEPDB0101
Mc Gowan, C	MOAD0103
McCallister, S	TUAB0102, TUAB0103
McCauley, M	MOAC0101LB, MOAC0106LB
McCloskey, R	MOPDA0101, TUAA0101
Mccloskey, R	TUAA0103
McComsey, G	TUPDB0103
McCoy, S	TUAC0104
McCoy, SI	MOAC0203
Mccoy, SI	MOAC0205LB
McElrath, J	TUAA0106LB
Mcfarland, W	TUAC0401
McGowan, CC	WEAD0104
McGowan, I	TUAC0206LB
Mcgrath, C	WEAD0205LB
Mchugh, G	WEAD0202
McHutchison, JG	TUAB0202
Mcingana, M	WEAC0106LB
McIntyre, J	WEAC0103
McKinstry, L	TUAC0106LB
McMahan, V	TUAC0201
McMahon, J	MOAA0106LB
Mcmahon, J	TUAD0102
Meanson, E	WEAD0204
Mejía-Villatoro, C	WEAA0103
Mekviwattanawan, S	TUAB0101
Melbourne, K	TUPDB0103
Mellado, MJ	WEAD0204
Merati, TP	TUPDC0103
Mercier, J	TUPDA0103
Merlini, E	TUPDA0101*
Mesplède, T	TUAA0104*
Metcalf Pate, K	MOAA0101*
Metivier, S	TUAB0207LB
Metras, M-E	TUAB0105
Meza, R	WEAA0103
Mhango, E	MOAC0201, WEPDD0103
Miailhes, P	TUAB0207LB
Micci, L	MOAA0102
Michael, N	TUAA0106LB
Midiani, D	MOAC0201
Miller, C	MOAA0204
Miller, R	MOAB0203
Miller, WC	WEAC0104, MOAC0202
Millett, G	WEAD0306LB
Milloy, M-J	MOPDA0101, MOPDD0105*, TUAC0103, TUAC0405
Milloy, MJ	TUAA0103, TUAC0403
Mills, L	MOAC0101LB
Mills, T	TUAB0102*
Min, JE	MOPDD0102, WEAD0301
Miro, JM	MOAB0203
Mirzazadeh, A	WEAC0105, TUPDC0104
Miyahara, S	MOAB0205LB
Mocroft, A	MOAB0203
Moebus, S	TUPDB0101
Mofolo, I	MOAC0202
Mohammed, T	WEPDA0105*
Mohapi, L	MOAB0205LB
Mokoena, I	TUAC0106LB
Mollan, K	TUAD0104, WEAA0106LB
Money, D	MOPDB0102, WEPDC0101, WEPDC0102
Montaner, J	WEAD0301, MOPDD0102, MOPDD0105, TUAA0101, TUAC0103, TUAC0405
Montefiori, D	TUAA0106LB
Monteiro, P	WEAA0105
Moodie, EEM	TUAB0201
Moore, D	MOAD0104
Moore, P	WEPDA0101
Moore, S	WEPDC0105
Moracco, KE	TUAD0202
Morales-Ramirez, J	TUAB0104
Moran, ME	MOPDC0104
Moreira, RI	TUAC0205LB
Moreno, A	TUAB0204
Moreno, S	TUAB0204*
Mori, M	WEPDA0102
Morissette, C	TUAC0406LB
Morris, L	WEPDA0101
Moses, A	MOAB0205LB
Moshgabadi, N	WEPDD0106*
Mosoko, JJ	TUAD0205
Mota, T	MOPDA0104
Mothopeng, T	TUAC0304
Moukambi, F	WEPDA0106LB*
Mourez, T	WEAB0103
Moyo, S	MOAC0106LB, WEPDA0105
Moyo, T	WEAA0102
Mpofu, D	TUAD0206LB
Msandiwa, R	MOAB0201
Mshana, G	MOAC0301LB
Msukwa, MT	MOAC0201
Mtande, T	MOAC0202
Mudender, F	TUPDD0104
Muffih, PT	MOPDC0106*
Mugo, C	WEAD0201
Mugo, NR	MOPDB0103
Mugurungi, O	MOAC0203, MOPDC0105
Mugyenyi, P	MOAB0205LB
Mujuru, HA	MOAC0203
Mukerjee, P	MOAA0104
Mukui, I	TUAD0103
Mulawa, M	TUPDC0105*
Mulder, N	MOAA0205
Mulema, V	WEAD0203
Mullins, JI	WEAA0104
Muñoz-Fernández, MÁ	WEAD0204
Munsakul, W	TUAB0101
Munyati, S	WEAD0202
Murakami-Ogasawara, A	MOPDA0102, WEAA0103
Murphy, RL	MOAB0102
Murray, C	WEAB0101
Murray, K	MOAC0302LB, TUAC0102
Musarandega, R	TUAD0203*
Musau, S	WEAD0305
Mushavi, A	MOAC0203, TUAD0203
Musiime, V	WEAD0203
Musinguzi, J	WEAD0203
Musomba, RZ	WEPDB0104
Musonda, R	WEPDA0105
Mustafa, M	TUPDC0103
Mutanha, N	WEAC0103
Mutanhaurwa, R	TUAC0203
Muula, A	MOPDC0102
Mwale, M	MOAC0202
Mwangomba, L	MOAC0202
Mwanyumba, S	TUAD0103
Myer, L	WEAD0101, MOAB0101, MOAB0107LB, WEPDB0103
Myers, S	TUPDD0104

## N

**Table:** 

Nabitaka, L	WEPDD0105
Naggie, S	TUAB0202
Naidoo, K	MOAB0205LB
Nair, G	TUAC0206LB
Najarro, K	MOAA0101
Nalugoda, F	WEPDC0105
Namey, E	WEAC0102*
Namuwenge, N	WEAD0203
Nanda, K	MOPDB0103
Nankya, I	TUAA0102
Napierala Mavedzenge, S	MOPDC0105*
Naranbhai, V	MOAA0106LB
Natumanya, E	WEPDD0105
Ncube, G	MOPDC0105
Ndhlovu, L	TUAA0204LB
Ndhlovu, LC	TUPDA0106LB
Ndjoyi-Mbiguino, A	WEPDB0101*
Ndlovu, B	WEPDA0101*
Ndulue, N	WEPDD0104*
Ndung'u, T	TUAA0105, WEPDA0101
Neilands, TB	MOPDC0101
Nelson, M	TUAB0206LB
Nelson, W	TUAA0106LB
Neumann, T	TUPDB0101
Newell, M-L	WEAD0103
Neytra, S	TUPDD0106
Nganga, L	WEAD0205LB, TUAD0103
Ngo-Giang-Huong, N	MOAC0204
Nguimkeu, P	WEAD0303
Nguyen, BC	TUAC0404
Nguyen, HH	TUAC0404
Nguyen, HL	TUAC0404
Nguyen, LH	TUAC0404
Nguyen, LV	MOAB0105
Nguyen, MS	TUAC0404
Nguyen, P	WEAC0101
Nguyen, T	TUAA0101
Nguyen, TTV	TUAC0404
Nhando, N	MOAC0106LB
Nicholas, S	WEPDD0103
Nickel, G	TUAA0102
Niehues, T	WEAD0204
Nielsen, K	MOAC0101LB
Nikolopoulos, G	TUPDC0101*
Nimitvilai, S	TUAB0101
Nitayaphan, S	TUAA0106LB
Njau, P	TUAC0104
Njuguna, I	WEAD0201
Noe, S	TUPDB0105
Nordio, J	MOAC0103
Nosyk, B	WEAD0301, MOPDD0102*
Nsumba, MS	WEPDB0104*
Ntombela, F	TUAC0101LB
Nunez, A	MOAC0103
Nunez, A	TUAD0105LB
Nyandindi, C	MOPDD0104
Nyirenda, M	MOAB0205LB
Nzengui Nzengui, GF	WEPDB0101

## O

**Table:** 

O'Connell, R	TUAA0106LB
Obel, N	MOAB0203
O'Brien, M	TUPDA0102*
Ochoa-Moreno, I	MOAC0205LB
O'Doherty, U	MOAA0103
Oishi, K	TUAD0101
Okala, SG	WEAA0101*
Okesola, N	WEAD0103
Okitolonda, E	TUAD0202
Okonji, E	TUAD0201, TUPDD0103
Omanga, E	MOAC0302LB, TUAC0102
Omolo, D	WEAD0205LB
Orkin, C	TUAB0206LB
Orne-Gliemann, J	WEAD0103, MOAC0104
Orrell, C	WEPDB0103
Osman, N	TUAA0104
Osmand, T	WEAC0103
Osoti, A	TUPDD0105
Ospina-Norvell, C	WEAD0105LB
Ostrowski, M	TUAA0204LB
Ou, SS	MOAC0106LB
Ou, S-S	MOAC0101LB, MOAC0305LB, MOAC0306LB
Ouattara, M	WEAC0102
Ouedraogo, G	TUAC0304
Owen, SM	MOAC0304LB

## P

**Table:** 

Padgett, D	WEAD0104
Padian, N	TUAC0104
Padian, NS	MOAC0203, MOAC0205LB
Pageaux, G-P	TUAB0207LB
Paiardini, M	MOAA0102*
Palma, P	WEAD0204
Palmer, S	MOAA0103
Palou, E	WEAA0103
Pan, J	TUAC0206LB
Panchia, R	MOAC0101LB, MOAC0106LB
Pang, PS	TUAB0202
Pankam, T	WEAB0102
Pannetier, J	WEPDC0103*
Panteleev, A	MOAB0203
Pape, JW	WEAD0104
Papoyan, A	WEAD0302
Parades, Z	TUPDD0104
Paraskevis, D	TUPDC0101
Parikh, S	TUPDD0105
Parslow, T	MOAA0202
Pascale, J	WEAA0103
Patel, M	MOAC0303LB
Patel, V	TUPDD0106
Pattanasin, S	TUAC0302
Peacock, D	MOPDC0101
Peeling, R	TUAD0104
Pence, BW	WEAC0104
Pendse, R	MOAD0101
Perelson, A	MOAA0106LB
Perez Elías, MJ	TUAB0204
Perez, A	MOAC0304LB
Perry, B	WEAC0102
Peters, P	MOAC0303LB
Peters, PJ	MOAC0304LB
Petersen, M	MOAC0203
Peterson, C	TUAA0202*
Petit, L	TUAD0205
Peton, N	MOAD0105LB
Petro, G	MOAB0107LB
Pettifor, A	TUAC0106LB*, TUPDD0101
Pettifor, AP	MOPDC0101
Peyrani, P	MOAC0304LB
Peytavin, G	WEAB0103, MOAA0105LB, MOAC0102
Pham, HT	TUAC0404
Phanuphak, N	WEAB0102, TUAC0306, WEAB0104
Phanuphak, P	WEAB0102, WEAB0104, TUAB0101, TUPDC0103
Phaswana-Mafuya, N	WEAC0106LB
Phillips, T	MOAB0107LB
Piatak, M	WEAA0106LB
Piazza, P	WEAA0104
Pick, N	MOPDB0102
Pilcher, C	WEAD0105LB*
Pillay, D	WEAD0103, MOAC0104, TUAC0105
Pillay, S	MOAB0205LB
Pina, C	TUPDD0106*
Ping, L-H	MOAC0106LB
Pinyakorn, S	WEAB0102
Piper, J	TUAC0206LB
Pitche, V	TUAC0304
Pitisuttithum, P	TUAA0106LB
Piwowar-Manning, E	MOAC0101LB, MOAC0106LB, MOAC0305LB, TUAC0106LB
Planas, D	WEAA0105*
Plantier, J-C	WEAB0103
Platt, HL	TUAB0206LB
Plazy, M	WEAD0103*
Plettenberg, A	TUAB0104
Plotkin, M	MOAC0301LB
Plummer, A	TUAB0102
Podlekareva, DN	MOAB0203
Pohlmeyer, C	MOAA0101
Polacino, P	TUAA0202
Pontones, P	MOAC0303LB, MOAC0304LB
Poon, A	TUAA0101
Poon, AFY	MOPDA0101, TUAA0103
Porcella, SF	MOAC0106LB, MOPDA0105
Porras, G	WEAA0103
Porter, K	MOAC0104
Portilla, J	TUAB0104
Post, F	TUAB0103
Post, FA	MOAB0203, TUPDB0102
Powers, KA	WEAC0104
Powis, K	MOPDB0104
Pozniak, A	MOAD0102, TUAB0103
Prasithsirikul, W	TUAB0101
Prasitsuebsai, W	MOAB0104, MOAB0105
Prentice, H	TUAA0106LB
Prestage, G	TUAC0306
Promda, N	TUAC0302
Prozesky, H	WEPDB0103
Prueksakaew, P	WEAB0102, WEAB0104
Psichogiou, M	TUPDC0101
Purcell, D	MOPDA0104*
Puren, A	MOAC0102
Puthanakit, T	TUPDB0104
Puttkammer, N	TUAD0204
Pyra, M	MOPDB0103*

## Q

**Table:** 

Quant, C	WEAA0103
Queen, S	MOAA0101
Quereda, C	TUAB0204
Quinn, T	MOPDA0105, WEPDC0105
Quinn, TC	MOAC0106LB
Quirk, E	MOAB0104
Quiroz-Morales, V	MOPDA0102

## R

**Table:** 

Rabezanahary, H	WEPDA0106LB
Racz, L	WEPDA0103
Raffi, F	TUAB0104
Rain-Taljaard, R	MOAC0102
Rajasingham, R	WEPDD0101
Rajkotia, Y	WEAD0303*
Rakhmanina, N	MOAB0104
Ramachandran, A	WEPDD0101*
Ramachandran, S	MOAC0304LB
Ramapuram, J	MOAB0106
Ramogothobeng, K	MOPDB0104
Rangel, G	MOPDD0103
Rao, S	TUAC0102
Ratanasuwan, W	TUPDC0103
Ratcliff, A	TUAA0102
Ravalihasy, A	WEPDC0103
Ravelomanana, NLR	TUAD0202
Ravindran, P	TUAB0106LB
Rawangban, B	MOAC0204
Rawat, A	MOAD0104*
Ray, N	TUAB0106LB
Raymond, A	MOAD0102
Rebeiro, PF	WEAD0104*
Redd, A	MOPDA0105
Redd, AD	MOAC0106LB
Reed, JS	TUPDA0106LB
Rees, H	WEAC0103
Reeves, RK	MOAA0206LB*
Reidner, G	MOAD0101
Reimann, K	MOAA0202
Reinsch, N	TUPDB0101
Reliquet, V	WEAD0204
Rerks-Ngarm, S	TUAA0106LB
Reyes Terán, G	MOPDA0102
Reyes, HLM	TUPDC0105
Reyes-Terán, G	WEAA0103
Reynolds, S	WEPDC0105
Reza, T	TUPDC0102
Richardson, B	TUPDD0105
Richardson, L	TUAC0405*
Richman, D	MOAA0103
Rinaldo, CR	WEAA0104
Riviere, C	MOAB0205LB
Robb, M	TUAA0106LB
Robertson, M	TUAB0206LB
Robinson, J	TUAD0203
Robison, J	WEPDD0102
Rocha, MM	MOAC0106LB
Roche, M	MOAA0106LB
Rockstroh, JK	TUAB0206LB*
Rodrigues, V	WEPDA0106LB
Rodriguez, B	MOAA0104
Rodriguez, F	WEAD0104
Rojo Conejo, P	WEAD0204
Rolland, M	TUAA0106LB
Rollet-Kurhajec, K	TUAB0205
Ronen, K	WEAD0205LB*
Rooney, J	TUAC0204LB
Rosalind, P-R	WEPDB0104
Roseberry, J	MOAC0303LB
Roseberry, JC	MOAC0304LB
Rosen, S	MOAB0202
Rosenberg, N	MOAC0202*
Rosenthal, E	TUAB0207LB
Rosenthal, K	MOAA0205
Rountree, W	WEPDA0103
Routy, J-P	WEAA0105
Rouzioux, C	MOAA0105LB
Rowhani-Rahbar, A	MOAB0103
Rowland-Jones, S	TUPDA0105
Roxby, A	TUPDD0105
Roy, É	TUAC0406LB
Ruane, PJ	TUAB0203
Rudy, B	TUAC0204LB
Rutherford, A	MOAC0103
Rutledge, B	TUAC0204LB
Ruxrungtham, K	TUAB0101
Ryan, E	MOAA0102

## S

**Table:** 

Saag, M	TUAB0202
Saag, MS	TUAB0206LB
Sabsanong, P	MOAC0204
Sabuni, N	MOPDD0104
Sacha, JB	TUPDA0106LB
Saeed, S	TUAB0205*
Saengtawan, P	WEAB0104
Saez-Cirion, A	MOAA0105LB*
Saidi, F	MOAC0202
Salazar-Vizcaya, L	WEAD0304
Saleh, S	MOPDA0104
Salgado, M	MOAA0101
Salmon, D	TUAB0207LB*
Salo, E	WEAD0204
Samson, L	WEPDC0101, WEPDC0102
Samsunder, N	TUAC0101LB
Sanchez, A	WEPDA0103
Sandoval, M	MOAC0304LB
Sandstrom, P	TUPDC0102
Sang, N	TUPDD0102
Santangelo, P	MOAA0202
Santos, BR	MOAC0101LB
Sathane, I	TUAC0401*
Sato, P	TUAC0106LB
Satumay, K	TUAC0302
Sauve, L	WEPDC0101, WEPDC0102
Schadendorf, D	TUPDB0101
Schafer, J	MOAA0206LB
Schechter, M	TUAC0201
Schechter, MT	TUAA0103
Scheepers, E	TUAD0201, TUPDD0103
Scherpbier, HJ	WEAD0204
Schley, A	TUPDD0103*
Schmid, RM	TUPDB0105
Schmitz, K	TUAD0201*
Schnippel, K	MOAB0202
Schomaker, M	WEAA0102, WEPDB0103
Schramm, B	WEPDD0103*
Schrijvers, R	TUAA0105
Schultze, A	MOAB0203*
Schulze, C	TUPDB0101
Schürmann, D	TUAB0106LB
Schwartz, S	WEAC0106LB*
Schwarz, M	WEPDD0102*
Schwerdtfeger, C	TUPDB0105
Scully, E	TUAA0205LB
Seclen-Palacin, J	WEPDD0105*
Sedaghat, A	WEAC0105*
Sekaly, R	MOAA0104
Selin, A	MOPDC0101, TUAC0106LB
Sema Baltazar, C	TUAC0401
Serwadda, D	WEPDC0105
Sevelius, JM	MOPDC0104
Sevinsky, H	TUAB0106LB
Shackleton, CR	WEPDD0106
Shah, M	WEPDD0101
Shamblaw, D	TUAB0102
Shang, H	TUAC0301
Shankar, A	MOAC0304LB
Shannon, K	WEAC0101
Shao, Y	MOAB0104
Shapiro, R	MOPDB0104
Sharifi, H	WEAC0105
Shattock, A	WEAD0302
Shattock, R	TUAA0102
Shattock, RJ	WEAA0101
Shaughnessy, M	TUAB0206LB
Shaw, A	MOAA0204*
Shearer, K	MOAB0202
Shen, X	TUAA0106LB
Shengelia, N	TUPDC0104
Shepherd, BE	WEAD0104, MOAD0103
Sherr, K	WEAD0201
Sherwood, J	WEAD0306LB
Shields, J	MOAC0303LB
Shields, R	MOPDC0106
Shighata, C	WEAC0102
Shikuma, C	TUAA0204LB
Shingadia, D	WEAD0204
Shirely Ko, SM	WEPDD0106
Shirk, E	MOAA0101
Shive, C	MOAA0104
Shokoohi, M	WEAC0105
Shwartz, J	TUAC0206LB
Sibanda, E	MOPDC0105
Sibanyoni, M	WEAC0103
Siberry, G	TUAC0204LB
Sievers, M	MOAC0106LB, MOPDA0105
Sikaris, K	MOAA0106LB
Silicano, J	MOAA0103
Silicano, R	MOAA0103
Siliciano, RF	MOPDA0105
Silima, M	WEAC0103
Sim, B	TUPDC0103
Simba, O	TUAC0104
Simon, K	WEPDD0102
Simoni, JM	TUAD0105LB
Simpson, S	TUPDA0105*
Singa, B	WEAD0205LB
Singer, J	WEPDC0101, WEPDC0102*
Singh, B	MOAC0102
Singtoroj, T	MOAB0105, TUPDC0103
Sirisanthana, T	TUPDC0103
Sirivichayakul, S	WEAB0104
Siriwachirachai, T	MOAC0204
Sithole, B	TUAC0304
Skowronska, E	TUPDD0103
Skrahin, A	MOAB0203
Slyker, J	WEAD0201
Slyker, JA	MOAB0103
Smith, D	TUAB0104
Smith, KN	WEAA0104
Sobotha, C	TUAB0106LB
Sodhi, S	MOPDC0102
Sogni, P	TUAB0207LB
Sohn, AH	MOAB0105*
Solomon, A	MOAA0106LB
Solomon, MM	TUAC0201
Solomon, S	MOAD0105LB
Somsouk, M	MOAA0204
Sonza, S	MOPDA0104
Soto-Nava, M	MOPDA0102, WEAA0103
Soule, M	MOPDD0101
Spinner, CD	TUPDB0105*
Spörri, A	MOAC0201
Spoulou, V	WEAD0204
Sripan, P	MOAC0204
Sriporn, A	TUAC0302
Ssali, F	MOAB0106
Ssekubugu, R	WEPDC0105
Ssemmondo, E	TUPDD0102
Stahlman, S	TUAC0304
Stamm, LM	TUAB0202
Stanley, C	MOAC0202
Stekler, J	MOPDC0103
Stevens, M	MOAB0106
Stinson, K	TUAD0102, WEPDB0103
Stintzi, A	MOAA0205
Stirratt, M	MOAC0305LB, MOAC0306LB
Stojiljkovic, T	WEPDB0102
Strathdee, S	WEAC0101, TUAC0402
Strathdee, SA	MOPDD0103
Strumpf, EC	TUAB0205
Struthers, H	WEAC0103
Stuart, R	WEAD0302
Sudjaritruk, T	MOAB0105, TUPDB0104*
Sugandhi, N	WEAD0203
Sulaberidze, L	TUPDC0104*
Sulkowski, M	TUAB0206LB
Sulkowski, MS	TUAB0202, TUAB0203
Sun, X	MOAB0205LB
Sungkanuparph, S	TUPDC0103
Sunseri, N	TUPDA0102
Supervie, V	WEPDC0103
Suthar, A	TUAC0404
Suttichom, D	WEAB0102
Swanstrom, R	MOAC0106LB, MOPDA0106LB
Sweitzer, S	WEAC0106LB
Swindells, S	MOAC0101LB
Switzer, WM	MOAC0304LB
Sylla, M	MOAB0102
Sypsa, V	TUPDC0101
Szumilin, E	WEPDD0103

## T

**Table:** 

Taha, T	MOAC0101LB
Taiwo, B	MOAB0102
Takebe, Y	TUPDC0103
Tal, K	MOAC0201
Taljjard, D	MOAC0102
Tang, W	TUAC0301, TUAD0104
Tanser, F	TUAC0105, WEPDB0103
Tapia, K	MOAB0103
Taruberekera, N	TUAC0304
Tayea, A	WEPDD0103
Taylor, A	TUAC0203
Taylor, J	MOPDA0101
Tayong, G	TUAD0205
Teague, A	TUPDB0102
Techapornroong, M	TUAB0101
Tee, KK	TUPDC0103*
Teeraananchai, S	MOAB0105
Tenakoon, S	MOAA0106LB
Tene, G	TUAD0205
Tenthani, L	MOAC0201
Teodoro, E	TUAC0401
Teppler, H	TUAB0104
Terris-Prestholt, F	TUAD0104
Thai, H	MOAC0304LB
Thirumurthy, H	MOAC0302LB*, TUAC0102*, TUAD0202
Thomas, KK	MOPDB0103
Thomas, R	TUAA0106LB*
Thompson, D	TUAD0202
Thompson, LH	TUPDC0102*
Thompson, M	TUAB0104
Tih, PM	TUAD0205*
Tippanonth, N	TUAC0302
Tiraboschi, JM	TUPDB0102
Toby, M	TUPDB0102
Toibaro, J	MOAB0203
Tollman, S	TUAC0106LB
Tomaras, G	TUAA0106LB
Torres, S	WEAD0105LB
Townley, E	TUAC0106LB
Trahan, M-J	TUAB0105
Tran, HT	TUAC0404
Traore, B	MOAB0102
Trapence, G	TUAC0304
Treves-Kagan, S	MOPDC0104, TUPDD0101*
Trichavaroj, R	WEAB0102, WEAB0104
Trinh, R	TUAB0203
Trunfio, M	TUPDA0101
Truong, KH	MOAB0105
Trypsteen, W	WEAB0101
Tsereteli, N	TUPDC0104
Tucker, J	TUAD0104*
Tumba, N	WEPDA0101
Tumbare, E	TUAD0203
Tumwesigye, E	MOAC0105LB
Turrini, F	TUPDA0104*
Turyamureeba, B	MOAC0105LB
Twine, R	MOPDC0101, TUAC0106LB, TUPDD0101

## U

**Table:** 

Uebel, KE	MOAD0104
Umar, E	TUAC0304
Urien, S	MOAC0204

## V

**Table:** 

Valantin, MA	TUAB0207LB
Valdez Madruga, J	TUAB0102
Valenzuela-Ponce, H	MOPDA0102, WEAA0103*
van der Elst, L	TUAC0101LB
Van Eygen, V	MOAB0106
van Heerden, A	MOAC0105LB
van Lettow, M	MOPDC0102
Van Lint, C	TUPDA0104
Van Lunzen, J	TUAB0102
van Oosterhout, J	MOPDC0102
van Oosterhout, JJ	MOAC0201
Van Rompay, K	WEAA0106LB
van Rooyen, H	MOAC0105LB
Van Schalkwyk, J	MOPDB0102
Vandekerckhove, L	WEAB0101*
Vandendriessche, A	WEAB0103*
Vasarhelyi, K	MOAC0103
Vaudry, W	WEPDC0101, WEPDC0102
Veloso, VG	MOAB0205LB, TUAC0205LB
Venter, F	WEAC0103
Ventura, A	MOPDC0104
Verhofstede, C	WEAB0101
Vermillion, M	MOAA0101
Vernazza, P	MOAD0102
Vervisch, K	WEAB0101
Vets, S	TUAA0105
Viana, R	WEPDB0102
Vibeke, R	WEAD0204
Vicenzi, E	TUPDA0104
Vicol, LA	MOPDB0102*
Viljoen, K	MOAA0205
Villinger, F	MOAA0202
Vincent, T	TUPDA0105
Vitoria, M	MOAD0101
Vittinghoff, E	TUAC0202
Volberding, P	TUAD0104
Volokha, A	WEAD0204
von Werder, A	TUPDB0105
Vu, QD	TUAC0404

## W

**Table:** 

Wagner, A	WEAD0201*
Wagner, R	TUAC0106LB
Wagner, T	TUAA0103
Wainberg, M	TUAA0104
Wainfen, W	MOPDC0106
Walker, BD	TUAA0103, WEPDA0101
Walker-Sperling, V	MOAA0101
Wallis, C	WEPDB0102*
Walmsley, S	TUAB0201
Wamalwa, D	WEAD0201, MOAB0103
Wambura, M	MOAC0301LB*
Wamicwe, J	TUAD0103
Wanasiri, P	MOAC0204
Wang, C	TUAB0203
Wang, J	TUAA0202
Wang, J	TUAC0106LB
Wang, Z	TUAC0301
Warnke, A	TUPDB0101
Warszawski, J	MOAA0105LB
Watadzaushe, C	MOAC0203
Waterhouse, D	MOAC0303LB
Wawer, M	WEPDC0105
Weaver, E	MOAA0204
Webb, GM	TUPDA0106LB*
Wei, C	TUAD0104
Wei, X	TUAB0103
Weidle, P	MOAC0303LB
Weiser, B	MOAA0203
Weiss, H	MOAC0301LB
Weissenhorn, W	TUPDA0103
Welty, T	MOPDC0106
Welty, T	TUAD0205
Wendoh, J	MOAA0205
Wenzi, LK	TUAD0202
Werb, D	MOPDD0103
Werlinrud Efsen, AM	MOAB0203
Wertheim, JO	TUPDC0102
Westgard, T	WEPDD0106
Wiesner, L	TUAD0103
Wijayaratne, S	WEAD0306LB
Wilen, C	TUPDA0102
Wilkinson, L	MOAD0105LB*
Williams, T	TUAC0203
Wilson, CM	TUAC0204LB
Wilson, D	WEAD0302
Wimonsate, W	TUAC0302
Wittkop, L	TUAB0207LB
Woelk, G	TUAD0206LB
Wolf, E	TUPDB0105
Wolff, M	MOAD0103*
Wolters, L	WEPDD0103
Wood, E	MOPDD0102, MOPDD0105, TUAC0103, TUAC0405
Woods, C	MOPDA0101
Wu, HL	TUPDA0106LB
Wu, V	TUPDA0102
Wyles, D	TUAB0203*
Wylie, JL	TUPDC0102

## X

**Table:** 

Xaba, T	WEPDB0102
Xia, G	MOAC0304LB
Xiao, H	TUAB0106LB
Xing, Y	TUAC0106LB
Xu, D	MOPDA0105
Xu, J	TUAC0301*
Xu, X	TUAB0104

## Y

**Table:** 

Yang, B	TUAD0104
Yang, JC	TUAB0202
Yang, L	TUAD0104
Yao, KA	WEAC0102
Yassi, A	MOAD0104
Yates, N	TUAA0106LB
Yazdanpanah, Y	TUAC0406LB
Yin, M	TUPDB0103
Yindom, L-M	TUPDA0105
Yotebieng, M	TUAD0202*
Young, J	TUAB0201
Young, P	TUAC0401
Youngleson, M	MOAD0105LB
Yu, Y	TUAC0301

## Z

**Table:** 

Zablotska, I	TUAC0306
Zamor, P	TUAB0206LB
Zang, O	WEAD0303
Zash, R	MOPDB0104
Zerbe, A	MOAB0107LB
Zhang, M	WEPDD0106
Zhang, Y	TUAA0203
Zhang, Y	TUAD0104
Zhou, S	MOPDA0106LB
Zhuang, M	TUAC0301
Ziegler, JB	WEAD0204
Zink, A	TUPDB0105
Zink, C	MOAA0101
Zolla-Pazner, S	TUAA0106LB
Zolopa, A	MOAB0205LB
Zolowere, DB	WEPDD0103
Zou, H	TUAC0301
Zuma, N	WEAC0103
